# Directed nickel-catalyzed regio- and diastereoselective arylamination of unactivated alkenes

**DOI:** 10.1038/s41467-021-26527-x

**Published:** 2021-11-01

**Authors:** Leipeng Xie, Shenghao Wang, Lanlan Zhang, Lei Zhao, Chun Luo, Linping Mu, Xiuguang Wang, Chao Wang

**Affiliations:** Tianjin Key Laboratory of Structure and Performance for Functional Molecules; College of Chemistry, Tianjin Normal University, Tianjin, 300387 People’s Republic of China

**Keywords:** Homogeneous catalysis, Synthetic chemistry methodology

## Abstract

Few methods have been reported for intermolecular arylamination of alkenes, which could provide direct access to important arylethylamine scaffolds. Herein, we report an intermolecular *syn*-1,2-arylamination of unactivated alkenes with arylboronic acids and *O*-benzoylhydroxylamine electrophiles with Ni(II) catalyst. The cleavable bidentate picolinamide directing group facilitates formation of stabilized 4-, 5- or 6-membered nickelacycles and enables the difunctionalization of diverse alkenyl amines with high levels of regio-, chemo- and diastereocontrol. This general and practical protocol is compatible with broad substrate scope and high functional group tolerance. The utility of this method is further demonstrated by the site-selective modification of pharmaceutical agents.

## Abstract (web summary)

Few methods exist to add a carbon and nitrogen atom across an unfunctionalized alkene. Here the authors show a method to regio- and diastereoselectively add aryl and amino groups to terminal and internal alkenes, via nickel catalysis and a removable directing group.

## Introduction

C–C and C–N bonds are two of the most omnipresent bonds in nature, and the arylamination of olefins represents a powerful and attractive synthetic tool for the simultaneous introduction of vital aryl and amino groups across alkenes to enable the rapid increase in molecular complexity from abundant and readily available materials^1–4^. Intramolecular arylamination with an alkene acceptor tethered to either the aryl halide or active nitrogen functionality using transition-metal catalysts has been developed for the synthesis of nitrogen-containing cycles^5–14^. In comparison, intermolecular arylamination providing access to arylethylamine-based acyclic molecules is particularly difficult and remain rare, owing to their high entropic cost and the problems of controlling the chemoselectivity of multicomponent reactions^15–20^.

**Fig. 1:**
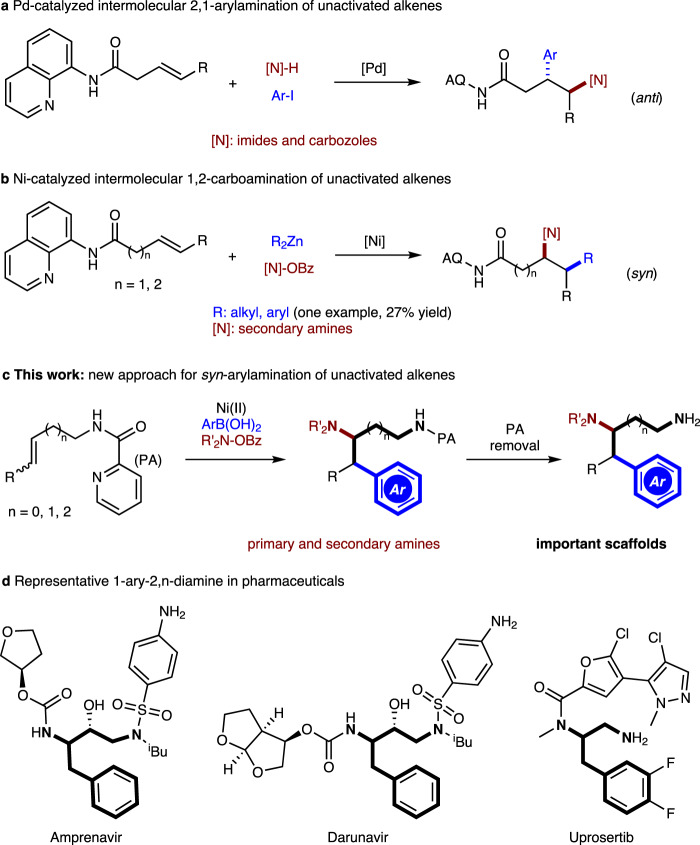
Background and synopsis of current work. **a** Pd-catalyzed intermolecular 2,1-arylamination of unactivated alkenes. **b** Ni-catalyzed intermolecular 1,2-carboamination of unactivated alkenes. **c** This work: an approach for *syn*-arylamination of unactivated alkenes. **d** Representative 1-ary-2,n-diamine in pharmaceuticals.

The arylethylamine scaffold exists in many bioactive molecules and pharmaceuticals, such as dopamine agonists^21,22^. Only a few methods have been reported for the synthesis of this important motif via 1,2/2,1-arylamination of alkenes. Liu and coworkers disclosed an enantioselective Cu-catalyzed 2,1-arylamination of terminal styrenes using the N-fluoro-N-alkylsulfonamide as the amine reagent via a radical process^15^. In 2018, Stephenson^16^ demonstrated a two-component photocatalytic 2,1-arylamination of vinyl arenes with arylsulfonylacetamides as the bifunctional reagents. For the more challenging arylamination of unactivated alkenes with low reactivity, reduced polarization, and high tendency toward *β*-hydride elimination, the directing group strategy has been utilized to address the issues. Recently, Engle achieved an *anti*-2,1-arylamination of 8-aminoquinoline tethered alkenyl carbonyls with aryl iodides and nitrogen nucleophiles via aminopalladation (Fig. 1a)^18^. After the pioneering work, this group reported a Ni-catalyzed 1,2-carboamination of alkenes with organozinc nucleophiles and *O*-benzoylydroxylamine (N–O) electrophiles in a syn-addition (Fig. 1b)^19^. However, the arylzinc reagent underwent arylamination with low efficiency. In this context, it is highly desirable to develop a protocol to enable regio- and stereoselective 1,2-arylamination of unactivated alkenes in terms of efficiency, alkene, and amine substrate scope, functional group tolerance.

Recently, functionality-tolerant and operationally simple arylboron has been used in the catalytic intermolecular dicarbofunctionalization of alkenes, such as diarylation^23–26^ and alkylarylation^27,28^. We, therefore, hypothesized that arylboron may be used with the N–O electrophiles to realize Ni-catalyzed arylamination of unactivated alkenes. However, to the best of our knowledge, intermolecular olefin arylamination with these two reagents remain scarce^17^. The development of this protocol for arylamination of unactivated alkenes was limited by three fundamental issues: (1) undesired competitive cross-couping between arylboron reagents and *O*-benzoylhydroxylamines^29,30^, (2) the low binding affinity of unactivated alkenes to metal centers, especially for internal alkenes, and (3) the difficulty to control regioselectivity, such as arylamination vs aminoarylation, and 1,2-arylamination vs 1,n-arylamination via chain-walking isomerization^31–33^.

Inspired by the flexibility of the coordinating directing group, we anticipated that the Ni-Ar species could be generated by transmetalation with arylboron reagents with a Ni(II) catalyst under the assistance of a bidentate directing group. Subsequent 1,2-migratory insertion followed by a sequential electrophilic amination reaction will yield the arylamination product^34–39^. Critical to the success of this process, the reaction must be initiated by a transmetalation step to form the Ni-Ar species, which is rarely reported and challenging because of the fact that most of the three-component difunctionalization reactions utilizing arylboron reagents were initiated by oxidative addition with electrophiles to Ni(0) catalyst, thus furnishing products that incorporate electrophile at the terminal position and aryl at the internal position.

Herein, we report an approach for the 1,2-arylamination of unactivated alkenes under practical and easily handled Ni(II) catalytic system with commercially available arylboronic acids and readily prepared *O*-benzoylhydroxylamines with excellent regio- and diastereocontrol (Fig. 1c). Under the assistance of picolinamide (PA)-directing group^40–42^, diverse alkenyl amines could be converted into valuable 1-aryl-2,n-diamines, a common structure found in a number of natural products and pharmaceuticals (Fig. 1d). This protocol is compatible with terminal and internal alkenyl amines of different chain lengths, a broad range of primary and secondary amine sources, and exhibits excellent functional group tolerance.

## Results

### Evaluation of reaction conditions

**Fig. 2:**
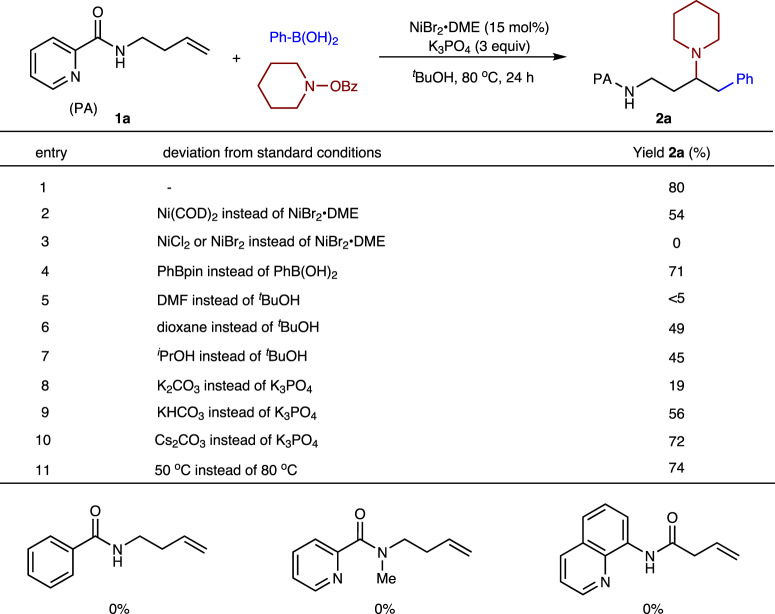
Variation of reaction parameters. Reactions conditions: **1a** (0.2 mmol), PhB(OH)_2_ (3 eq), piperidino benzoate (2 eq), ^t^BuOH (2 mL). Yields were for isolated and purified products.

To check the feasibility of the above scenario, we started this investigation of Ni-catalyzed arylamination of homoallylic amine substrate **1a** containing a PA-directing group with phenylboronic acid and piperidino benzoate as coupling partners (Fig. 2). After extensive optimization, we were pleased to find that the reaction in the presence of NiBr_2_·DME as the catalyst and K_3_PO_4_ in *tert*-butanol at 80 °C gave the desired 1,2-arylamination product **2a** as a single regioisomer in 80% isolated yield, accompanying with ≈5% unreacted alkene and trace amounts of hydroarylation byproduct (entry 1). Ni(COD)_2_ also catalyzed the reaction in moderate yield (54%) (entry 2). NiCl_2_ or NiBr_2_ catalyst did not give any desired product, probably due to the lack of stabilizing spectator ligand to promote ligand exchange/transmetalation (entry 3). Replacement of nucleophile PhB(OH)_2_ by PhBpin led to a slightly lower yield (entry 4). The use of DMF solvent led to a trace amount of product (entry 5), while the use of dioxane or ^*i*^PrOH solvent provided a lower yield (entries 6–7). Inferior results were obtained when using other bases instead of K_3_PO_4_ (entries 8–10) or conducting the reaction at 50 °C (entry 11). Benzoyl protected homoallylic amine and N-methylated PA were subjected to the standard conditions, and both reactions did not occur, indicating that the pyridine N(sp^2^) and N-H moiety were both indispensable in this transformation. It merits to mention that, the putative 8-aminoquinoline-masked 3-butenoic acid^43–47^, which was widely used in alkene difunctionalization, did not afford any product under the optimized conditions.

### Substrate scope

**Fig. 3:**
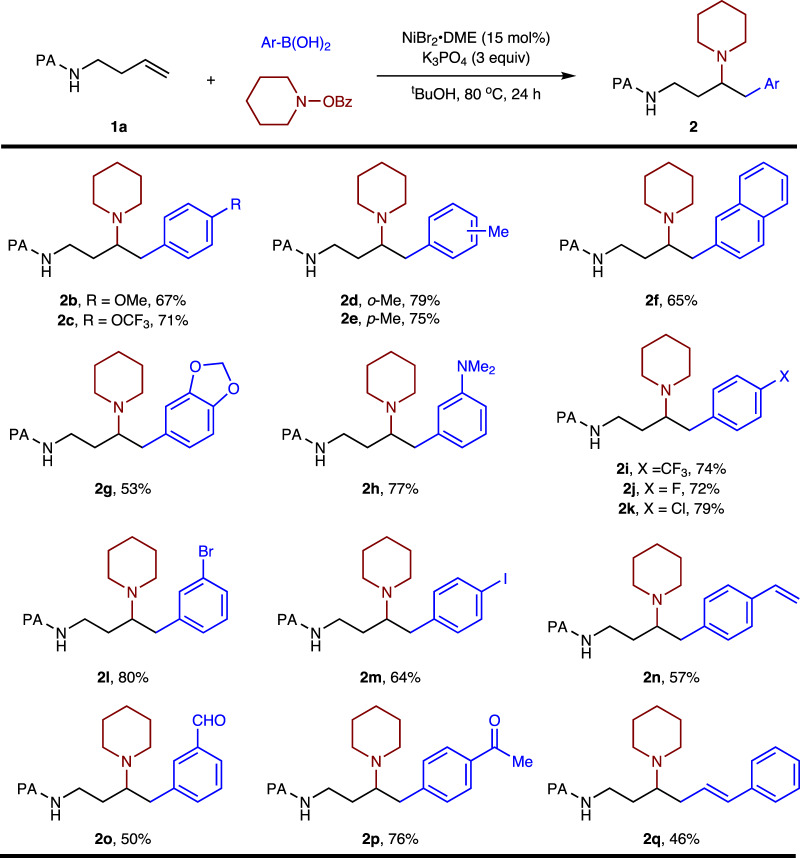
Scope of arylboronic acids. Reactions conditions: **1a** (0.2 mmol), ArB(OH)_2_ (3 eq), piperidino benzoate (2 eq), ^t^BuOH (2 mL).

With the identified conditions in hand, we first sought to define the scope of the arylboronic acid partner (Fig. 3). In general, arylboronic acids bearing a wide range of electronically varied substituents reacted to produce the desired products in good yields. A variety of functional groups were accommodated well, including ethers (**2b-c**), dimethylamine (**2h**), trifluoromethyl (**2i**), fluoro (**2j**), chloro (**2k**) and bromo (**2l**). Notably, sterically hindered ortho-substituted arylboronic acid showed slightly increased reactivity, reacted to afford the desired product in very good yield (**2d**). In addition, arylboronic acids containing iodide, alkene, aldehyde and ketone (**2m**-**p**), which can be further derivatized, were also amenable to this reaction. Gratifyingly, alkenylboronic acid worked well to give the desired product (**2q**) with moderate yield. On the other hand, alkyl- and alkynylboronic acid coupling partners were ineffective under the reaction conditions.

**Fig. 4:**
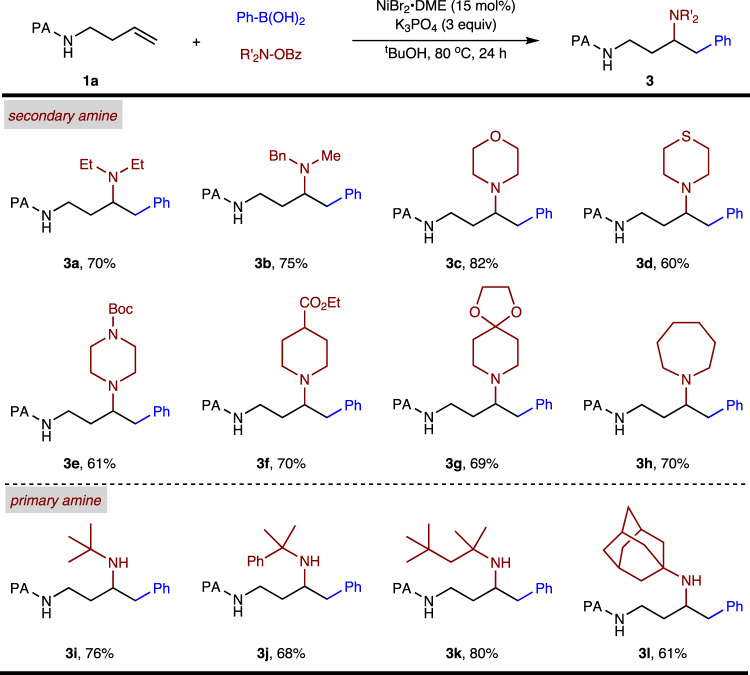
Scope of amine electrophiles. Reactions conditions: **1a** (0.2 mmol), PhB(OH)_2_ (3 eq), N–O reagent (2 eq), ^t^BuOH (2 mL).

Subsequently, we examined the N–O electrophile scope of the reaction using PhB(OH)_2_ as the nucleophilic component (Fig. 4). The reactivities of disubstituted amine sources were first investigated in the arylamination, diethylamine, and *N*-methylbenzylamine could be introduced under the optimized conditions (**3a-b**). In addition to acyclic amines, cyclic amines containing a series of heterocyclic scaffolds, including morpholine (**3c**), thiomorpholine (**3d)**, piperazine (**3e)**, ester group (**3f)**, and acetal group (**3g**), which are prevalent in biologically active molecules, were competent substrates. Seven-membered hexamethyleneimine was also tolerated, affording the desired product **3h**. The scope of monosubstituted amine transfer agents for the synthesis of secondary amines was explored, which usually led to a dramatic drop in reaction efficiency^48,49^. To our delight, sterically hindered secondary alkylamines are accessible with *O*-benzoylhydroxylamines derived with tertiary alkyl group (**3i-l**). However, less sterically hindered primary amine transfer agents gave no desired arylamination product, affording hydroarylation product in moderate yield.

**Fig. 5:**
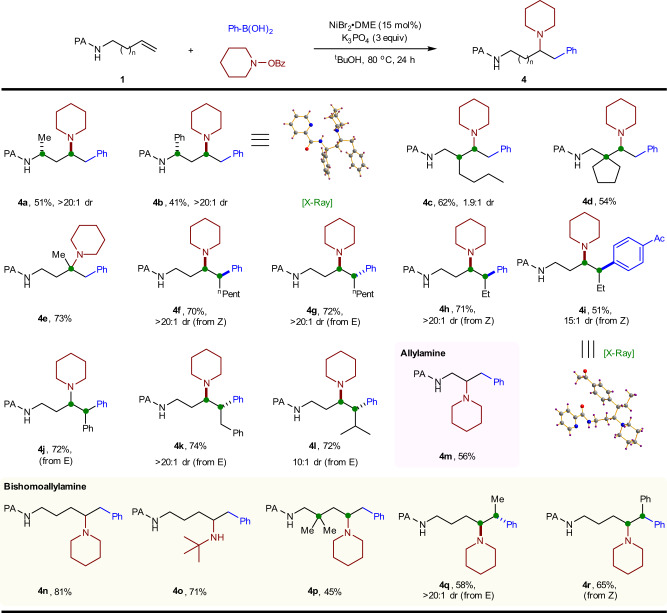
Scope of alkenes. Reactions conditions: **1** (0.2 mmol), ArB(OH)_2_ (3 eq), N–O reagent (2 eq), ^t^BuOH (2 mL); dr was determined by NMR or GC-MS analysis of the crude products.

After evaluation of electrophile and nucleophile scope, we turned our attention to the scope of alkene substrates (Fig. 5). The α-substituted homoallylamine was first tested under the standard conditions, the terminal alkene bearing alkyl- or aryl-substitution at the α-position proceeded smoothly to afford the arylamination products (**4a-b**) in moderate yields with high diastereoselectivity. The relative stereochemistry of the diastereomer of **4b** was established by X-ray crystallographic analysis, with the *trans* orientation of α and γ substituents. This result established the relative stereochemistry of two stereocenters remote from one another, which has been shown to be difficult. The *trans* orientation of the skipped stereocenters generated from arylamination of *α-*substituted terminal alkenes presumably arose from the formation of the more stable *trans*-nickelacycle upon C–N bond reductive elimination (see models in the Supplementary Information). *n-*Butyl-substituted homoallylamine at the β-position could also be tolerated in the reaction but with lower diastereoselectivity (1.9:1, **4c**), and a more sterically bulky group led to a lower yield (**4d**). 1,1-Disubstitued alkene was also tolerated and successfully afforded desired product containing a quaternary center (**4e**). We then explored alkene substrates that are typically challenging in alkene carboamination. Gratifyingly, Z- or E- internal substrates could be efficiently converted into the *syn*-diastereomer under the optimized conditions (**4f-l**), which was consistent with our proposed mechanism. Both diastereoisomers were accessible based on the *cis*/*trans* configuration of the alkene substrate (**4f** and **4g**), suggesting the alkene does not undergo isomerization in the Ni-catalyzed process. In addition, phenyl-, benzyl-, and isopropyl-substituents were well tolerated in the reaction.

In light of the success in developing a regioselective arylamination of homoallylamines, we continued our survey by applying the protocol to PA-protected allylic and bishomoallylic amines. To our delight, the reaction of allylamine with phenylboronic acid and piperidino benzoate under the optimized conditions furnished the arylamination product in moderated yield, with only a single regioisomer was detected by GC-MS analysis. Moreover, a variety of terminal and internal bishomoallylamines underwent arylamination to regioselectively provide δ-amino benzenepentanamine products. Likewise, both *trans*- and *cis*-internal alkenes were effective in this reaction, delivering the desired product with excellent diastereoselectivity (>20:1, **4q**). We assumed that four and six-membered nickelacycles were formed and can be stabilized in the catalytic system.

### Synthetic potential

**Fig. 6:**
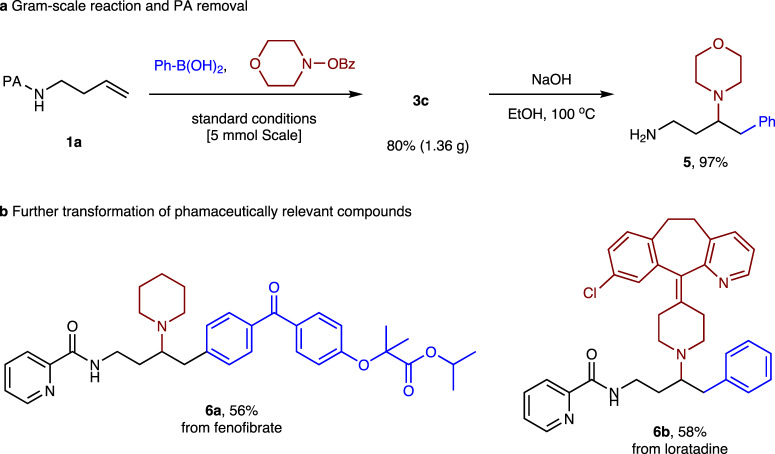
Synthetic potential. **a** Gram-scale reaction and PA removal. **b** Further transformation of phamaceutically relevant compounds.

We next performed the gram-scale reaction to illustrate the synthetic utility of this methodology (Fig. 6a). The reaction of **1a** with phenylboronic acid and morpholine benzoate on a 5 mmol scale afforded **3c** in 80% yield. We were able to remove the PA-directing group with NaOH in EtOH at 100 °C, and the primary amine **5** could be generated in nearly quantitative yield. This methodology was also applied to the modification of phamaceutically relevant compounds, as shown in Fig. 6b. Arylboronic acid and *O*-benzoylhydroxylamine derived from fenofibrate and loratadine independently underwent arylamination, affording corresponding desired products (**6a** and **6b**).

### Mechanistic consideration

**Fig. 7:**
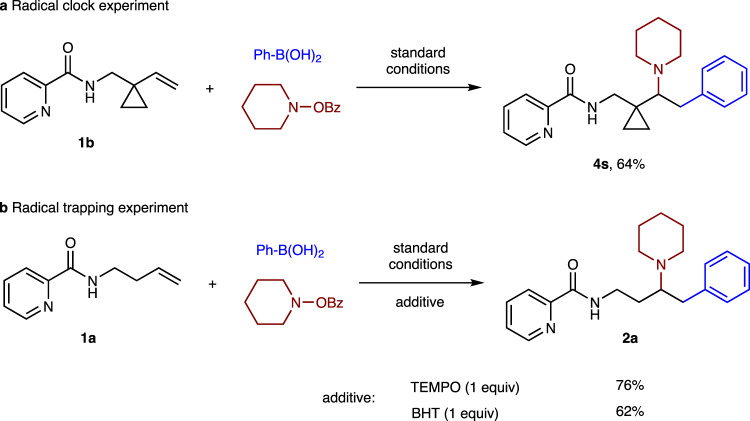
Mechanistic investigations. **a** Radical clock experiment with cyclopropyl-containing substrate **1b**. **b** Radical trapping experiment with TEMPO or BHT. *TEMPO* 2,2,6,6-tetramethyl-1-piperidinyloxy, *BHT* butylated hydroxytoluene.

To elucidate the mechanism, we first conducted the radical clock experiment (Fig. 7a). When the prepared alkene substrate **1b** was subjected to standard reaction conditions, only cyclopropane remained product **4s** was formed in 64% yield, implying that the cyclopropylmethyl radical intermediate known to ring rupture might not be generated in the catalytic cycle. The effect of radical inhibitors was next examined (Fig. 7b). As it turned out, the arylamination was not largely inhibited by the addition of TEMPO or BHT. This suggested that the arylamination probably did not involve a radical process, although the possibility of radical formation followed by fast recombination with Ni within the solvent cage cannot be ruled out.

**Fig. 8:**
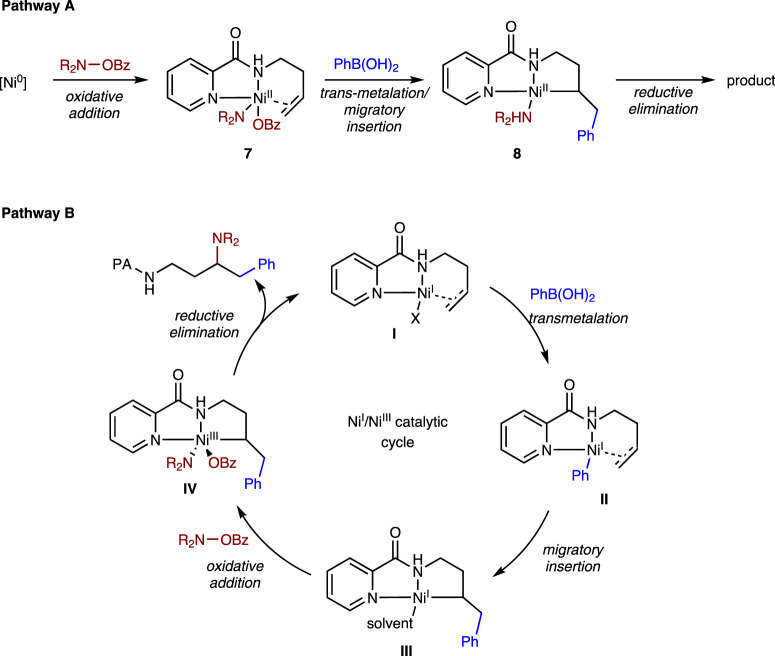
Proposed mechanism. Two possible pathways for the Ni-catalyzed 1,2-arylamination of alkenyl amines.

Regarding redox manifolds of Ni catalysis, Ni^0^/Ni^II^ and Ni^I^/Ni^III^ catalytic systems were considered as two possible pathways (Fig. 8). In Pathway A, a Ni(II) species **7** was generated from Ni(0) species and *O*-benzoylhydroxylamine via oxidative addition. Transmetalation followed by migratory insertion of the alkene into the Ni-carbon bond would form **8**, which could yield the product by reductive elimination. However, we consider this pathway to be less likely because the selective insertion into the Ni-C rather than Ni–N bond was suspicious and the energy barrier of reductive elimination of Ni^II^ amido species is too high under similar catalytic system based on DFT calculations (>50 Kcal/mol)^50^. An alternative pathway in which the alkene inserted into the Ni–N bond precedes transmetalation and C–C reductive elimination was also considered unlikely, because it would involve the formation of thermodynamically unfavored larger nickelacycles, especially for bishomoallylic amine substrates (seven-membered nickelacycles). Alternatively in Pathway B, the reaction was initiated by a Ni(I) species (**I**)^51,52^, which was formed probably from comproportionation between NiBr_2_ and Ni(0) species^53,54^. After further transmetalation with arylboronic acids and olefin migratory insertion, nickel-alkyl species (**III**) was generated. The species stabilized by bidentate PA-directing group underwent oxidative addition with the aminating reagent much faster than protonation with the alcohol solvent or β-hydride elimination, forming Ni^III^ amido species **IV**, which was believed to be able to undergo facile reductive elimination^55–58^. Finally, the active Ni(I)-X catalyst **I** was regenerated, and the desired product was furnished through the subsequent ligand exchange with the alkene substrate.

**Fig. 9:**
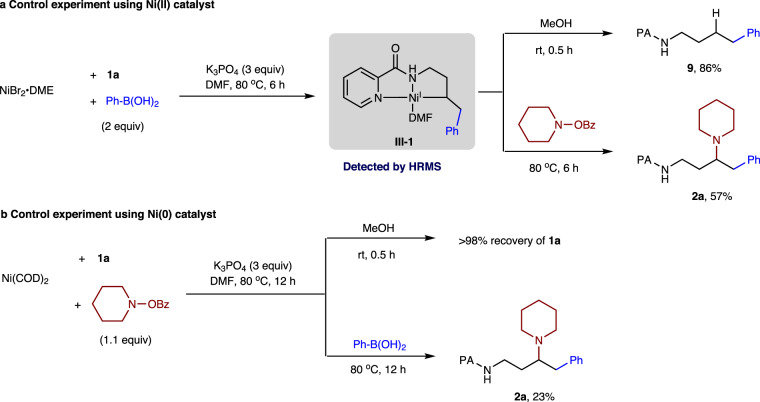
Control experiments. **a** Control experiment using Ni(II) catalyst. **b** Control experiment using Ni(0) catalyst. See the Supplemental Information for detailed experimental procedures and HR-MS (high-resolution mass spectrometer) analysis. *DMF* dimethylformamide.

To further support our aforementioned mechanism, a series of control experiments were conducted. Stoichiometric reaction of **1a** with NiBr_2_•DME followed by treatment with two equivalents of phenylboronic acid for 6 h was conducted to generate Ni(I) (**III-1**) complex (Fig. 9a), addition of methanol to the solution of complex **III-1** furnished the hydroarylation product **9** (phenyl unit located at terminal position) in 86% yield upon alcoholic protonation. Besides, the **III-1** complex that was subjected to piperidino benzoate at 80 °C for 6 h successfully afforded the expected product **2a** in 57% yield. These results demonstrated that the reaction could be initiated by transmetalation of arylboronic acid with Ni(II) precatalyst and subsequent alkene insertion occurred prior to engagement with an electrophile to give the observed regiochemical outcome. Although attempts to isolate and identify the intermediate was failed, the proposed nickel intermediate ligated with DMF was detected by HR-MS (ESI) analysis (m/z calcd for [M + H]^+^, 385.1300; found, 385.1308, Supplementary Fig. 2). On the other side, a control experiment of **1a** with Ni(COD)_2_ followed by treatment with piperidino benzoate at 80 °C for 12 h was conducted (Fig. 9b). In contrast, the reaction mixture resulted in >98% recovery of **1a** upon alcoholic quenching, without the formation of any hydroamination product. This result indicated that migratory insertion of the alkene did not occur into the Ni(II)-N bond, whereas oxidative addition of piperidino benzoate to Ni(0) should be easy. In a separate vessel, the same reaction mixture reacted with phenylboronic acid at 80 °C for 12 h afforded **2a** in a low yield (23%), and no aminoarylation side product was detected. Overall, we preferred the reaction proceeded via a Ni^I^/Ni^III^ catalytic cycle (Pathway B) rather than a Ni^0^/Ni^II^ cycle.

## Discussion

In summary, we have disclosed a methodology for the regio- and diastereoselective intermolecular arylamination of unactivated alkenes with base-metal catalyst and readily available reagents under simple conditions. The removable bidentate PA auxiliary facilitated the formation of stabilized 4-, 5-, or 6-membered nickelacycles and enabled the difunctionalization of both terminal and internal alkenyl amines, leading to the concomitant introduction of important aryl groups and structurally diverse amino groups into the C=C bonds with good functional group compatibility. Interestingly, the reaction of α-substituted terminal alkenes led to the formation of *trans*-isomeric products with high levels of diastereoselectivity, in which two stereocenters were remote from each other. Our protocol was suitable for large-scale synthesis and the synthetic utility of this method was further demonstrated by the modification of pharmaceutical agents. The expansion of this strategy to the synthesis of drug and natural products and other electrophiles for alkene difunctionalization is currently underway in our laboratory.

## Methods

### General procedure for the Ni-catalyzed arylamination of alkenyl amines

In an argon-filled glovebox, NiBr_2_•DME (0.03 mmol, 15 mol%), K_3_PO_4_ (0.6 mmol, 3.0 eq), alkene substrate (0.2 mmol, 1.0 eq), appropriate amine benzoate electrophile (0.4 mmol, 2 eq), appropriate arylboronic nucleophile (0.6 mmol, 3.0 eq), *t*-BuOH (2 mL) were added to a 10 mL schlenk flask. The reaction mixture was stirred at 80 °C for 24 h and the resulting solution was concentrated in vacuum. The crude product was purified by column chromatography on alumina gel with a mixture of ethyl acetate and hexane as eluent. The conditions for flash chromatography and data for characterization of the products are listed below.

## Supplementary information

Supplementary Information

## Data Availability

X-ray crystallographic data for compounds **4b** (CCDC 2054628) and **4i** (CCDC 2054629) is freely available from the Cambridge Crystallographic Data Centre. Copies of the data can be obtained free of charge via https://www.ccdc.cam.ac.uk/structures/. All other data in support of the findings of this study are available within the article and its Supplementary Information or from the corresponding author. Source data are provided with this paper.

## Source data

Source Data
