# Supplementary material for: Directed nickel-catalyzed regio- and diastereoselective arylamination of unactivated alkenes
Source: Nat Commun. 2021 Nov 1;12:6280. doi: 10.1038/s41467-021-26527-x (PMC8560905; doi:10.1038/s41467-021-26527-x)
Supplement: Supplementary file 1 — Supplementary Information [file 41467_2021_26527_MOESM1_ESM.pdf]

Supplementary Information

Directed Nickel-Catalyzed Regio- and Diastereoselective Arylamination of Unactivated Alkenes

Leipeng Xie[§], Shenghao Wang[§], Lanlan Zhang[§], Lei Zhao, Chun Luo, Linping Mu, Xiuguang Wang, and Chao Wang*

Tianjin Key Laboratory of Structure and Performance for Functional Molecules; College of Chemistry, Tianjin Normal University, Tianjin 300387, People's Republic of China

E-mail: chwang@tjnu.edu.cn

Table of Contents

I. Supplementary Methods……………………………………………………………….S3

1. General Remarks.. S3

2. Alkene Substrate Synthesis S3

3. General Procedure for the Ni-Catalyzed Arylamination of Alkenyl Amines S13

4. Gram-scale Reaction and PA Removal.. S31

5. Further Transformation of Phamaceutically Relevant Compounds.................................. S32

6. Radical Trapping Experiment S33

7. Radical Clock Experiment S34

8. Control Experiment.. S35

9. HRMS(ESI) Analysis.. S36

10. X-ray Crystallographic Data S37

11. Models for a-Substituted Terminal Alkenes S38

12. NMR Spectra S39

II. Supplementary References…………………………………………………………....S106

I. Supplementary Methods

1. General Remarks

All the manipulations were performed in an argon-filled glovebox, unless mentioned otherwise. Anhydrous solvent was purchased from commercial sources and transferred under argon atmosphere. Alkene substrates and Amine benzoate substrates were prepared according to previously reported procedures, all arylboronic acids were purchased from commercial sources and used without further purification. All reagents were purchased from Energy Chemicals and used as received.

^{1}H NMR, ^{13}C NMR spectra were recorded using Bruker 400 MHz NMR spectrometer. ^{1}H NMR and ^{13}C NMR spectra were referenced to resonances of the residual protons in the deuterated solvents. Multiplicities are recorded as: s = singlet, d = doublet, t = triplet, dd = doublet of doublets, br = broad singlet and m = multiplet. GC-MS analysis was performed on Shimadzu GC-2010 gas chromatography coupled to a Shimadzu QP2010 mass selective detector. Analytical HPLC/MS was performed with an Agilent 6520 Series HPLC. Agilent 1200 Series HPLC.

2. Alkene Substrate Synthesis

Supplementary Table 1. Picolinamide-containing alkene substrates 1b-1k.

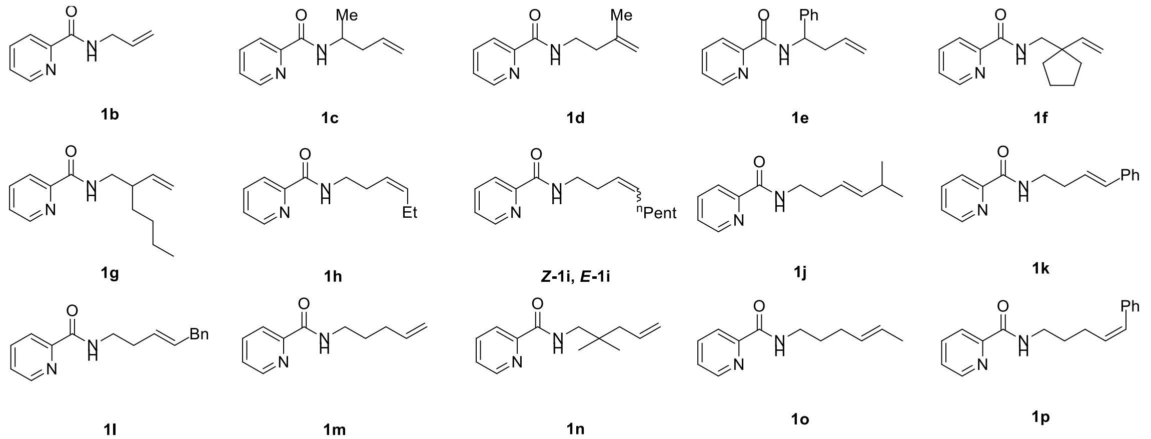

General Procedure for Amide Coupling (GP1):

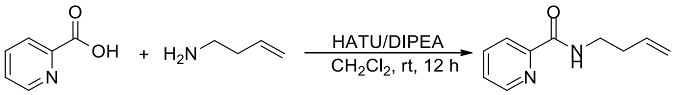

Compound **1b, 1m** were synthesized from amines[1]

To a 50 mL flask was added alkenyl carboxylic amide (10 mmol, 1.0 eq), picolinic acid (12 mmol, 1.2 eq), HATU (11 mmol, 1.1 eq), DIPEA (20 mmol, 2.0 eq) and CH_2Cl_2 (30 mL). The reaction mixture was left to stir for 12 h. Upon completion, the reaction was quenched with brine (10 mL), and extracted with CH_2Cl_2 (50 mL × 4). The organic layers were combined, and the solvent was removed in vacuo to yield a yellow residue. Purification using column chromatography gave the pure product.

General Procedure for Amide Coupling(GP2):

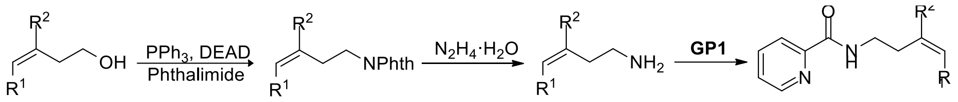

Compound **1c-1e, 1h-1i, 1o** were synthesized from enols[2].

To a mixture of triphenylphosphine (25 mmol, 1.0 eq), phthalimide (25 mmol, 1.0 eq) and the corresponding allyl alcohol (25 mmol, 1.0 eq) in THF (30 mL) was slowly added diethyl azodicarboxylate (DEAD) (25 mmol, 1.0 eq) at 0 °C. The mixture was stirred at 0 ºC for 3 h. After the completion of the reaction, the reaction mixture was diluted with *n*-hexane and filtered. The filtrate was dried over Na_2SO_4 and concentrated in vacuo to give the crude product, which was used without further purification.

To the solution of phthalimide product in ethanol (100 mL) was added hydrazine monohydrate (25 mmol) at 50 ºC. The mixture was stirred for 1 h and quenched with 6 M HCl (20 mL). The precipitates formed were removed by filtration, and the resultant filtrate was dried over Na_2SO_4 and concentrated in vacuo to give an unsaturated amine hydrochloride. Aqueous NaOH (6.0 M, 10 mL) was added to the amine salt, and the resulting solution was extracted with CH_2Cl_2 (25 mL × 3). The combined organic extracts was then washed again with brine (10 mL), dried over Na_2SO_4, and filtered. The amine solution was used without further purification.

To the solution of amine (25 mmol, 1.0 eq) was successively added picolinic acid (30 mmol, 1.2 eq), HATU (27.5 mmol, 1.1 eq) and DIPEA (50 mmol, 2.0 eq). The resultant mixture was stirred at room temperature overnight. Water was added and the mixture was extracted with CH_2Cl_2 (50 mL × 3). The combined organic layers were washed with water and brine, dried over anhydrous Na_2SO_4, filtered and concentrated in vacuo. The resulting residue was purified by alumina gel flash chromatography (ethyl acetate:hexanes = 1:8) to give the desired product.

Synthesis of compound **1f**.

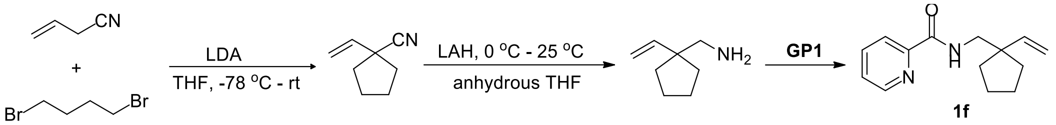

To a 100 mL schlenk flask was added lithium diisopropylamide (44 mmol, 1.1 eq, 2.0 M in THF), anhydrous THF (10 mL) under Ar atmosphere. The resulting solution was submerged in a -78 °C dry ice bath. A solution of acrylonitrile (40 mmol, 1.0 eq) in 10 mL THF was added dropwise over 5 min, and a solution of 1,4-dibromobutane (38 mmol, 0.95 eq) in 10 mL THF was added dropwise over 0.5 h. After 4 h at this temperature, the solution was quenched slowly with water (10 mL). The aqueous layer was transferred to a separatory funnel and washed with Et_2O (50 mL × 2) before being charged back into the schlenk flask. Hydrochloric acid was added dropwise into the vigorously stirring solution at 0 °C until pH = 3. The milky solution was then extracted with EtOAc (100 mL × 2). The combined organic extracts were washed with brine (50 mL), dried over Na_2SO_4, concentrated, and carried forward to the next step without further purification.

To a 250 mL oven-dried flask under Ar atmosphere was added anhydrous THF (100 mL) followed by $LiAlH_4$ (15 mL, 2.4 M in THF). A solution of nitrile (40 mmol, 1.0 eq) in THF (50 mL) was added dropwise at 0 °C. The reaction vessel was allowed to warm to room temperature and left to stir for 3 h. After this time, the reaction mixture was diluted with Et_2O, washed with 1 M HCl, sat. $NaHCO_3$ solution, brine and extracted with EtOAc (10 mL × 5)[3]. The organic solvent is removed to get a yellow oil which is used to synthesize the final product **1f** through the process **GP1**.

Synthesis of compound **1g**.

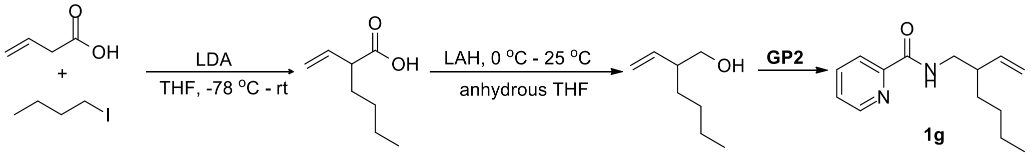

To a 100 mL schlenk flask was added LDA (55 mmol, 2.2 eq, 2.0 M in THF), anhydrous THF (10 mL) under Ar atmosphere. The resulting solution was submerged in a -78 °C dry ice bath. A solution of 3-butenoic acid (25 mmol, 1.0 eq) in 5 mL THF was added dropwise over 5 min, and a solution of 1-iodobutane (25 mmol, 1.0 eq) in 5 mL THF was added dropwise over 5 min. After 4 h at this temperature, the solution was quenched slowly with water (10 mL). The aqueous layer

was transferred to a separatory funnel and washed with Et_2O (50 mL × 2) before being charged back into the schlenk flask. Hydrochloric acid was added dropwise into the vigorously stirring solution at 0 °C until pH = 3. The milky solution was then extracted with EtOAc (100 mL × 2). The combined organic extracts were washed with brine (50 mL), dried over Na_2SO_4, concentrated, and carried forward to the next step without further purification.

To a 250 mL oven-dried flask under Ar atmosphere was added anhydrous THF (100 mL) followed by LAH (15 mL, 2.4 M in THF). A solution of acid (25 mmol, 1.0 eq) in THF (50 mL) was added dropwise at 0 °C. The reaction vessel was allowed to warm to room temperature and left to stir for 3 h. After this time, the reaction mixture was diluted with Et_2O, washed with 1 M HCl, sat. $NaHCO_3$ solution, brine and extracted with EtOAc (10 mL × 5)[3]. The organic solvent is removed to get a yellow oil which is used to synthesize the final product **1g** through the process **GP2**.

Synthesis of compound **1j, 1l**.

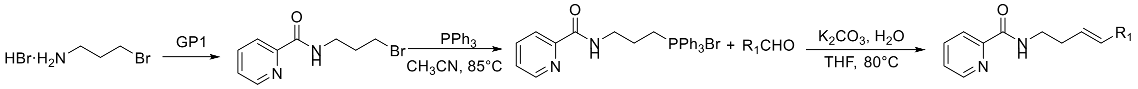

To a 100 mL schlenk flask was added 3-Bromopropylamine hydrobromide (30 mmol, 1.0 eq), then through the process **GP1**. After that, the reaction mixture was diluted with EtOAc (100 mL) and washed with brine (3×100 mL). The organic layer was separated, dried over Na_2SO_4 , and concentrated, and carried forward to the next step without further purification.

PPh_3 (30 mmol, 1.0 eq), CH_3CN (150 mL) was added to the resulting solution and then the reaction vessel was allowed to heat to reflux under argon for 48 h. After this time, the reaction vessel was cooled to room temperature, removed solvent by vacuum. Then added aldehyde (30 mmol, 1.0 eq), K_2CO_3 (45 mmol, 1.5 eq), H_2O (30 mmol, 1.0 eq) in THF (80 mL) was stirred at 80 °C for 12 h. After that, the combined organic layers were washed with water and brine, dried over anhydrous Na_2SO_4, filtered and concentrated in vacuo. The resulting residue was purified by silica gel flash chromatography (ethyl acetate:hexanes = 1:8) to give the desired product.

Synthesis of compound **1k**.

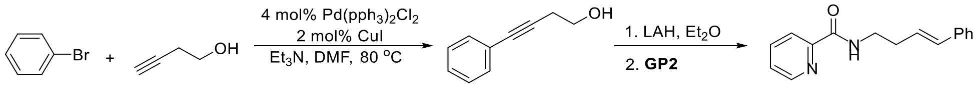

To a 100 mL flask was added bromobenzene (50 mmol, 1.0 eq), but-3-yn-1-ol (55 mmol, 1.1 eq), $Pd(pph_3)_2Cl_2$ (0.008 mmol, 4 mol%), CuI (0.004 mmol, 2 mol%), Et_3N (60 mmol, 1.2 eq) and DMF (50 mL). The reaction mixture was left to stir for 12 h at 80 °C. Upon completion, the reaction was quenched with brine (10 mL), and extracted with CH_2Cl_2 (50 mL × 4). The organic layers were combined, and the solvent was removed in vacuo to yield a brown residue. Purification using column chromatography gave the pure product[4].

To a suspension of LAH (75 mmol, 1.5 eq) in diethyl ether (0.3 M) under N_2 at 0 °C was slowly added a solution of 4-phenylbut-3-yn-1-ol (50 mmol, 1.0 eq). After 15 min the reaction was allowed to warm to room temperature and stirred for an additional 3 h. The reaction was re-cooled to 0 °C, diluted with wet Et_2O, quenched by slow addition of aq. NaOH (1 M), stirred for an additional 0.5 h, filtered through celite and concentrated under reduced pressure. The resulting crude product was used to synthesize the final product **1k** through the process **GP2**.

Synthesis of compound **1n**.

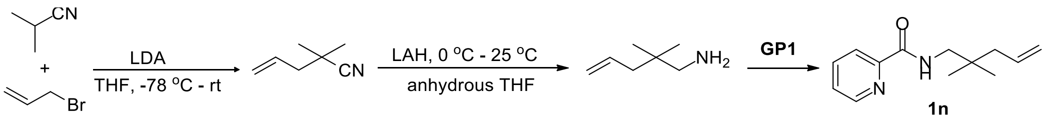

To a 100 mL schlenk flask was added LDA (66 mmol, 1.1 eq, 2.0 M in THF), anhydrous THF (10 mL) under Ar atmosphere. The resulting solution was submerged in a -78 °C dry ice bath. A solution of isobutyronitrile (60 mmol, 1.0 eq) in 10 mL THF was added dropwise over 5 min, and a solution of allyl bromide (72 mmol, 1.2 eq) in 10 mL THF was added dropwise over 0.5 h. After 4 h at this temperature, the solution was quenched slowly with water (10 mL). The aqueous layer was transferred to a separatory funnel and washed with Et_2O (50 mL × 2) before being charged back into the schlenk flask. Hydrochloric acid was added dropwise into the vigorously stirring solution at 0 °C until pH = 3. The milky solution was then extracted with EtOAc (100 mL × 2). The combined organic extracts were washed with brine (50 mL × 1), dried over Na_2SO_4, concentrated, and carried forward to the next step without further purification.

To a 250 mL oven-dried flask under Ar atmosphere was added anhydrous THF (100 mL) followed by $LiAlH_4$ (15 mL, 2.4 M in THF). A solution of nitrile (60 mmol, 1.0 eq) in THF (50 mL) was added dropwise at 0 °C. The reaction vessel was allowed to warm to room temperature and left to stir for 3 h. After this time, the reaction mixture was diluted with Et_2O, washed with 1 M HCl, sat.

$NaHCO_3$ solution, brine and extracted with EtOAc (10 mL × 5)[3]. The organic solvent is removed to get a yellow oil which is used to synthesize the final product **1n** through the process **GP1**.

Synthesis of compound **1p**.

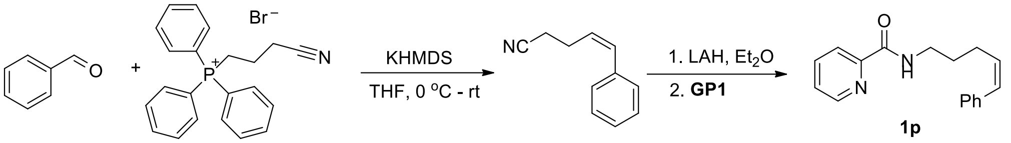

1p

To a suspension of (3-cyanopropyl)triphenylphosphonium bromide (7.3 mmol, 1.1 eq) in 20 mL THF at 0 °C was added KHMDS (20 wt% in THF, 8.3 mmol, 1.25 eq) slowly over 2 min, which resulted in an orange suspension. The reaction was stirred at 0 °C for 15 min before the benzaldehyde (6.65 mmol, 1.0 eq) was added in one portion. The reaction was stirred an additional 0.5 h at 0 °C, 1.5 h at room temperature. The resulting mixture was filtered through a silica plug, concentrated and purified by flash column silica gel chromatography using the indicated solvent system[5].

To a suspension of LAH (10 mmol, 1.5 eq) in Et_2O (0.3 M) under N_2 at 0 °C was slowly added a solution of nitrile (6.65 mmol, 1.0 eq). After 15 min the reaction was allowed to warm to room temperature and stirred for an additional 3 h. The reaction was re-cooled to 0 °C, diluted with wet Et_2O, quenched by slow addition of aq. NaOH (1 M), stirred for an additional 0.5 h, filtered through celite and concentrated under reduced pressure. The resulting crude product was used to synthesize the final product **1p** through the process **GP1**.

N-allylpicolinamide **(1b)**

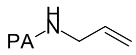

The title compound was isolated as a colorless oil after chromatography on silica with ethyl acetate/hexane (1:6). ^1H NMR (400 MHz, $CDCl_3$) δ 8.54 (m, 1H), 8.20 (m, 1H), 8.14 (s, 1H), 7.84 (m, 1H), 7.42 (m, 1H), 5.94 (m, 1H), 5.27 (m, 1H), 5.17 (m, J = 10.2, 1.5 Hz, 1H), 4.10 (m, 2H); ^{13}C NMR (101 MHz, CDCl3) δ 164.18, 149.87, 148.09, 137.36, 134.07, 126.18, 122.29, 116.44, 41.80. GC-MS (EI): Calcd for $C_{11}H_{14}N_2O$: 162.08, found: 162.10.

N-(pent-4-en-2-yl)picolinamide **(1c)**

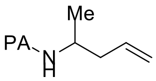

The title compound was isolated as a yellow oil after chromatography on silica with ethyl acetate/hexane (1:8). ^{1}H NMR (400 MHz, $CDCl_3$) δ 8.50 (m, 1H), 8.19–8.12 (m, 1H), 7.92 (br, 1H), 7.80 (m, 1H), 7.41–7.34 (m, 1H), 5.89–5.73 (m, 1H), 5.16–5.01 (m, 2H), 4.23 (m, 1H), 2.33 (d, J = 6.7 Hz, 2H), 1.26–1.20 (m, 3H); 13C NMR (101 MHz, CDCl3) δ 163.55, 150.11, 148.05, 137.38, 134.45, 126.09, 122.23, 117.88, 44.81, 41.02, 20.32. GC-MS (EI): Calcd for $C_{11}H_{14}N_2O$: 190.11, found: 190.15.

***N-(3-methylbut-3-en-1-yl)picolinamide* (1d)**

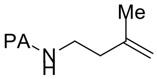

The title compound was isolated as a yellow oil after chromatography on silica with ethyl acetate/hexane (1:8). ^{1}H NMR (400 MHz, $CDCl_3$) δ 8.57–8.51 (m, 1H), 8.19 (d, *J* = 7.8 Hz, 1H), 8.08 (br, 1H), 7.84 (m, 1H), 7.41 (m, 1H), 4.88–4.77 (m, 2H), 3.61 (dd, *J* = 12.9, 6.9 Hz, 2H), 2.35 (t, *J* = 6.9 Hz, 2H), 1.79 (s, 3H); ^{13}C NMR (101 MHz, $CDCl_3$) δ 164.32, 150.13, 148.18, 142.72, 137.48, 126.19, 122.33, 112.41, 37.62, 37.40, 22.38. GC-MS (EI): Calcd for $C_{11}H_{14}N_2O$: 190.11, found: 190.14.

***N-(1-phenylbut-3-en-1-yl)picolinamide* (1e)**

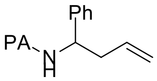

The title compound was isolated as a yellow solid after chromatography on silica with ethyl acetate/hexane (1:8). ^{1}H NMR (400 MHz, $CDCl_3$) δ 8.55 (dd, *J* = 4.8, 0.7 Hz, 1H), 8.44 (br, 1H), 8.18 (d, *J* = 7.8 Hz, 1H), 7.83 (m, 1H), 7.46–7.29 (m, 5H), 7.25 (m, 1H), 5.77 (m, 1H), 5.27 (dd, 1H), 5.22–5.04 (m, 2H), 2.77–2.65 (m, 2H); ^{13}C NMR (101 MHz, $CDCl_3$) δ 163.67, 149.96, 148.18, 141.85, 137.48, 134.08, 128.74, 127.46, 126.70, 126.30, 122.46, 118.38, 52.90, 40.90. GC-MS (EI): Calcd for $C_{16}H_{16}N_2O$: 252.13, found: 252.10.

***N-((1-vinylcyclopentyl)methyl)picolinamide* (1f)**

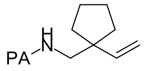

The title compound was isolated as a colorless oil after chromatography on silica with ethyl acetate/hexane (1:8). ^{1}H NMR (400 MHz, $CDCl_3$) δ 8.54 (m, 1H), 8.19 (m, 1H), 8.11 (br, 1H), 7.83 (m, 1H), 7.41 (m, 1H), 5.90 (dd, 1H), 5.16–5.07 (m, 2H), 3.47 (d, *J* = 6.1 Hz, 2H), 1.74–1.63 (m, 6H), 1.59 (d, J = 6.9 Hz, 2H); 13C NMR (101 MHz, CDCl3) δ 164.37, 150.02, 148.11, 144.25, 144.22, 137.26, 126.00, 122.24, 113.22, 113.18, 50.03, 46.43, 46.40, 46.36, 34.92, 23.99. GC-MS (EI): Calcd for C14H18N2O: 230.14, found: 230.15.

***N-(2-vinylhexyl)picolinamide* (1g)**

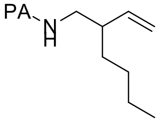

The title compound was isolated as a yellow oil after chromatography on silica with ethyl acetate/hexane (1:8). ^{1}H NMR (400 MHz, $CDCl_3$) δ 8.52 (dd, *J* = 2.8, 1.9 Hz, 1H), 8.17 (d, *J* = 7.8 Hz, 1H), 8.10 (br, 1H), 7.82 (dd, *J* = 10.7, 4.7 Hz, 1H), 7.48–7.34 (m, 1H), 5.63 (m, 1H), 5.11 (dd, *J* = 13.3, 5.3 Hz, 2H), 3.76–3.49 (m, 1H), 3.34–3.09 (m, 1H), 2.28 (dd, *J* = 8.3, 4.7 Hz, 1H), 1.34 (m, 6H), 0.87 (s, 3H); ^{13}C NMR (101 MHz, $CDCl_3$) δ 164.29, 150.15, 148.19, 140.49, 137.37, 126.11, 122.29, 116.77, 44.46, 43.35, 32.14, 29.32, 22.79, 14.13. GC-MS (EI): Calcd for $C_{14}H_{20}N_2O$: 232.16, found: 232.15.

***(Z)-N-(hex-3-en-1-yl)picolinamide* (1h)**

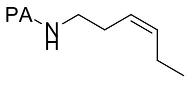

The title compound was isolated as a yellow oil after chromatography on silica with ethyl acetate/hexane (1:8). 1H NMR (400 MHz, $CDCl_3$) δ 8.64–8.35 (m, 1H), 8.30–8.07 (m, 1H), 8.11 (s, 1H), 7.82 (m, 1H), 7.40 (m, 1H), 5.61–5.47 (m, 1H), 5.37 (m, 1H), 3.49 (q, J = 6.7 Hz, 2H), 2.37 (m, 2H), 2.05 (m, 2H), 0.93 (t, J = 7.5 Hz, 3H); 13C NMR (101 MHz, CDCl3) δ 164.34, 150.12, 148.11, 137.45, 134.78, 126.16, 125.14, 122.29, 39.25, 27.51, 20.75, 14.35. GC-MS (EI): Calcd for $C_{12}H_{16}N_2O$: 204.13, found:204.10.

***(Z)-N-(non-3-en-1-yl)picolinamide* (Z-1i)**

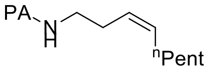

The title compound was isolated as a yellow oil after chromatography on silica with ethyl acetate/hexane (1:8). ^{1}H NMR (400 MHz, $CDCl_3$) δ 8.50 (d, *J* = 4.6 Hz, 1H), 8.16 (dd, *J* = 7.3, 3.4 Hz, 1H), 8.10 (br, 1H), 7.85–7.74 (m, 1H), 7.37 (m, 1H), 5.55–5.32 (m, 2H), 3.52–3.41 (m, 2H), 2.41–2.29 (m, 2H), 2.01 (d, *J* = 6.6 Hz, 2H), 1.24 (dd, 6H), 0.84–0.73 (m, 3H); ^{13}C NMR (101 MHz, $CDCl_3$) δ 164.27, 150.08, 148.04, 137.34, 133.12, 126.07, 125.66, 122.20, 39.18, 31.51, 29.36, 27.56, 27.35, 22.59, 14.07. GC-MS (EI): Calcd for $C_{15}H_{22}N_2O$: 246.17, found: 246.15.

***(E)-N-(non-3-en-1-yl)picolinamide* (E-1i)**

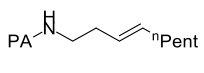

The title compound was isolated as a colorless oil after chromatography on silica with ethyl acetate/hexane (1:8). ^{1}H NMR (400 MHz, $CDCl_3$) δ 8.53 (d, *J* = 4.7

Hz, 1H), 8.19 (d, J = 7.8 Hz, 1H), 8.09 (br, 1H), 7.84 (m, 1H), 7.41 (m, 1H), 5.61–5.52 (m, 1H), 5.49–5.39 (m, 1H), 3.50 (dd, J = 12.9, 6.8 Hz, 2H), 2.32 (m, 2H), 2.01 (m, 2H), 1.38–1.32 (m, 2H), 1.28–1.22 (m, 4H), 0.86 (t, J = 6.8 Hz, 3H); ^{13}C NMR (101 MHz, $CDCl_3$) δ 164.30, 150.24, 148.14, 137.40, 133.86, 126.44, 126.12, 122.28, 39.16, 32.80, 32.73, 31.43, 29.27, 22.67, 14.16. GC-MS (EI): Calcd for $C_{15}H_{22}N_2O$: 246.17, found: 246.20.

***(E)-N-(5-methylhex-3-en-1-yl)picolinamide* (1j)**

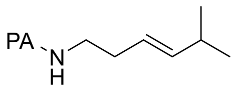

The title compound was isolated as a yellow oil after chromatography on silica with ethyl acetate/hexane (1:8). 1H NMR (400 MHz, $CDCl_3$) δ 8.66–8.39 (m, 1H), 8.18 (m, 1H), 8.12 (s, 1H), 7.82 (td, J = 7.7, 1.8 Hz, 1H), 7.39 (m, 1H), 5.40–5.29 (m, 1H), 5.25 (dd, J = 10.9, 7.3 Hz, 1H), 3.48 (q, J = 6.7 Hz, 2H), 2.59 (m, 1H), 2.37 (m, 2H), 0.91 (d, J = 6.7 Hz, 6H); 13C NMR (101 MHz, CDCl3) δ 164.35, 150.15, 148.11, 140.65, 137.40, 126.15, 123.35, 122.25, 39.34, 27.73, 26.72, 23.22. GC-MS (EI): Calcd for $C_{13}H_{18}N_2O$: 218.14, found:218.18.

***(E)-N-(4-phenylbut-3-en-1-yl)picolinamide* (1k)**

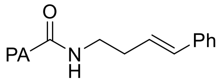

The title compound was isolated as a colorless oil after chromatography on silica with ethyl acetate/hexane (1:8). ^{1}H NMR (400 MHz, $CDCl_3$) δ 8.52 (m, 1H), 8.20 (d, J = 7.8 Hz, 1H), 8.17 (br, 1H), 7.84 (m, 1H), 7.42–7.38 (m, 1H), 7.38–7.33 (m, 2H), 7.30 (dd, J = 8.2, 6.8 Hz, 2H), 7.24–7.19 (m, 1H), 6.51 (d, 1H), 6.24 (m, 1H), 3.63 (m, 2H), 2.56 (m, 2H); ^{13}C NMR (101 MHz, $CDCl_3$) δ 164.45, 150.08, 148.18, 137.46, 137.44, 132.44, 128.64, 127.34, 127.02, 126.25, 126.21, 122.30, 39.13, 33.33. GC-MS (EI): Calcd for $C_{16}H_{16}N_2O$: 252.13, found: 252.15.

***(E)-N-(5-phenylpent-3-en-1-yl)picolinamide* (1l)**

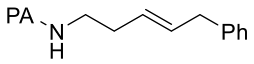

The title compound was isolated as a yellow oil after chromatography on silica with ethyl acetate/hexane (1:8). ^{1}H NMR (400 MHz, $CDCl_3$) δ 8.54 (d, J = 4.7 Hz, 1H), 8.20 (d, J = 7.8 Hz, 1H), 8.16 (br, 1H), 7.85 (m, 1H), 7.44–7.41 (m, 1H), 7.22 (dd, J = 8.6, 6.1 Hz, 2H), 7.18–7.13 (m, 3H), 5.73 (m, 1H), 5.61–5.54 (m, 1H), 3.57 (dd, J = 13.2, 6.8 Hz, 2H), 3.43 (d, J = 7.3 Hz, 2H), 2.52 (m, 2H); ^{13}C NMR (101 MHz, $CDCl_3$) δ 164.42, 150.10,

148.15, 140.74, 137.43, 131.20, 128.51, 128.45, 126.90, 126.18, 126.01, 122.31, 39.18, 33.68, 27.75. GC-MS (EI): Calcd for $C_{15}H_{22}N_2O$: 266.14, found: 266.20.

N-(pent-4-en-1-yl)picolinamide **(1m)**

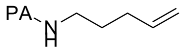

The title compound was isolated as a colorless oil after chromatography on silica with ethyl acetate/hexane (1:6). ^1H NMR (400 MHz, $CDCl_3$) δ 8.54 (m, 1H), 8.20 (m, 1H), 8.09 (br, 1H), 7.84 (m, 1H), 7.42 (m, 1H), 5.84 (m, 1H), 5.13–4.94 (m, 2H), 3.49 (dd, J = 13.4, 7.0 Hz, 2H), 2.22–2.12 (m, 2H), 1.78–1.70 (m, 2H); ^{13}C NMR (101 MHz, $CDCl_3$) δ 164.35, 150.13, 148.11, 137.87, 137.52, 126.20, 122.35, 115.37, 39.03, 31.27, 28.93. GC-MS (EI): Calcd for $C_{11}H_{14}N_2O$: 190.11, found: 190.15.

N-(2,2-dimethylpent-4-en-1-yl)picolinamide **(1n)**

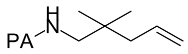

The title compound was isolated as a colorless oil after chromatography on silica with ethyl acetate/hexane (1:6). ^1H NMR (400 MHz, $CDCl_3$) δ 8.56 (m, 1H), 8.20 (m, 2H), 7.86–7.81 (m, 1H), 7.42 (m, 1H), 5.92–5.81 (m, 1H), 5.11–5.06 (m, 2H), 3.31 (d, J = 6.7 Hz, 2H), 2.06 (d, J = 7.5 Hz, 2H), 0.97 (s, 6H); ^{13}C NMR (101 MHz, $CDCl_3$) δ 164.48, 150.20, 148.19, 137.47, 134.93, 126.17, 122.43, 117.76, 49.10, 44.70, 35.23, 25.17. GC-MS (EI): Calcd for $C_{13}H_{18}N_2O$: 218.14, found: 218.15.

(E)-N-(hex-4-en-1-yl)picolinamide **(1o)**

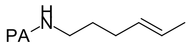

The title compound was isolated as a colorless oil after chromatography on silica with ethyl acetate/hexane (1:8). ^1H NMR (400 MHz, $CDCl_3$) δ 8.57–8.51 (m, 1H), 8.19 (d, J = 7.8 Hz, 1H), 8.07 (br, 1H), 7.84 (m, 1H), 7.41 (m, 1H), 5.57–5.36 (m, 2H), 3.47 (m, 2H), 2.09 (m, 2H), 1.75–1.67 (m, 2H), 1.65 (d, J = 4.8 Hz, 3H); ^{13}C NMR (101 MHz, $CDCl_3$) δ 164.32, 150.21, 148.11, 137.42, 130.36, 126.12, 125.90, 122.27, 39.08, 30.15, 29.49, 18.03. GC-MS (EI): Calcd for $C_{12}H_{16}N_2O$: 204.13, found: 204.15.

(Z)-N-(5-phenylpent-4-en-1-yl)picolinamide **(1p)**

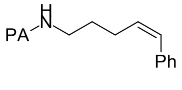

The title compound was isolated as a colorless oil after chromatography on silica with ethyl acetate/hexane (1:8). ^1H NMR (400 MHz, $CDCl_3$) δ 8.52 (d, J = 4.8 Hz,

1H), 8.19 (d, J = 7.8 Hz, 1H), 8.06 (br, 1H), 7.83 (m, 1H), 7.41 (dd, J = 7.5, 4.8 Hz, 1H), 7.28 (m, 4H), 7.20 (t, J = 6.9 Hz, 1H), 6.47 (d, J = 11.6 Hz, 1H), 5.69 (m, 1H), 3.50 (m, 2H), 2.52–2.38 (m, 2H), 1.83–1.75 (m, 2H); ^{13}C NMR (101 MHz, $CDCl_3$) δ 164.39, 150.13, 148.12, 137.57, 137.44, 131.70, 129.89, 128.84, 128.30, 126.72, 126.17, 122.29, 39.12, 29.96, 26.11. GC-MS (EI): Calcd for $C_{17}H_{18}N_2O$:266.14, found: 266.20.

3. General Procedure for the Ni-Catalyzed Arylamination of Alkenyl Amines

In an argon-filled glovebox, $NiBr_2$•DME (0.03 mmol, 15 mol%), K_3PO_4 (0.6 mmol, 3.0 eq), alkene substrate (0.2 mmol, 1.0 eq), appropriate amine benzoate electrophile (0.4 mmol, 2 eq), appropriate aryl boronic nucleophile (0.6 mmol, 3.0 eq), *t*-BuOH (2 mL) were added to a 10 mL schlenk flask. The reaction mixture was stirred at 80 °C for 24 h and the resulting solution was concentrated in vacuum. The crude product was purified by column chromatography on alumina gel with a mixture of ethyl acetate and hexane as eluent. The conditions for flash chromatography and data for characterization of the products are listed below.

N-(4-phenyl-3-(piperidin-1-yl)butyl)picolinamide **(2a)**

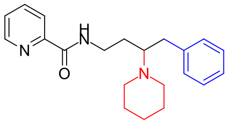

The title compound was isolated as a colorless oil (80% yield) after chromatography on alumina with ethyl acetate/hexane (1:3). ^{1}H NMR (400 MHz, $CDCl_3$) δ 9.22 (br, 1H), 8.48–8.42 (m, 1H), 8.07 (d, J = 7.8 Hz, 1H), 7.72 (m, 1H), 7.32–7.28 (m, 1H), 7.19 (t, J = 7.4 Hz, 2H), 7.11 (d, J = 7.3 Hz, 1H), 7.05 (d, J = 7.2 Hz, 2H), 3.59 (m, 1H), 3.11 (m, 1H), 2.98 (dd, J = 13.0, 3.2 Hz, 1H), 2.74 (m, 3H), 2.44 (m, 2H), 2.23 (dd, J = 13.0, 10.5 Hz, 1H), 1.73–1.60 (m, 5H), 1.55–1.41 (m, 3H); ^{13}C NMR (101 MHz, $CDCl_3$) δ 164.60, 150.46, 147.94, 140.57, 137.20, 129.28, 128.54, 126.00, 125.94, 122.23, 68.14, 49.89, 39.53, 34.53, 28.15, 26.01, 25.15. HRMS (ESI) m/z calculated for $C_{21}H_{27}N_3O$ $[M+H]^+$ 338.2232, found 338.2235.

N-(4-(4-methoxyphenyl)-3-(piperidin-1-yl)butyl)picolinamide **(2b)**

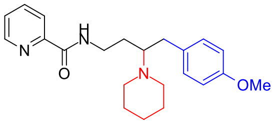

The title compound was isolated as a colorless oil (67% yield) after chromatography on alumina with ethyl acetate/hexane (1:3). ^{1}H NMR (400 MHz, $CDCl_3$) δ 9.30 (br, 1H), 8.56–8.49 (m, 1H), 8.16 (d, J = 7.8 Hz, 1H), 7.81 (m, 1H), 7.39 (m, 1H), 7.06 (d, J = 7.7 Hz, 2H), 6.85–6.79 (m, 2H), 3.79 (s, 3H), 3.68 (s, 1H), 3.20 (m, 1H), 3.00 (d, J = 10.4 Hz, 1H), 2.79 (s, 3H), 2.50 (s, 2H), 2.24 (s, 1H), 1.73 (s, 6H), 1.51 (s, 2H); ^{13}C NMR (101 MHz, $CDCl_3$) δ 162.68, 156.11, 156.01, 146.03, 135.26, 135.20, 128.23, 123.99, 120.28, 112.14, 53.48, 48.01, 47.95, 37.59, 31.71, 26.34, 24.24, 23.25. HRMS (ESI) m/z calculated for $C_{22}H_{29}N_3O_2$ $[M+H]^+$ 368.2338, found 368.2339.

N-(3-(piperidin-1-yl)-4-(4-(trifluoromethoxy)phenyl)butyl)picolinamide **(2c)**

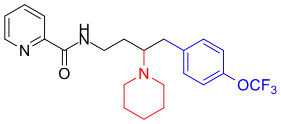

The title compound was isolated as a white solid (71% yield) after chromatography on alumina with ethyl acetate/hexane (1:3). ^{1}H NMR (400 MHz, $CDCl_3$) δ 9.14 (br, 1H), 8.52 (d, J = 4.6 Hz, 1H), 8.16 (d, J = 7.8 Hz, 1H), 7.82 (m, 1H), 7.42–7.36 (m, 1H), 7.13 (m, 4H), 3.68 (m, 1H), 3.26 (m, 1H), 3.04 (dd, J = 13.2, 3.5 Hz, 1H), 2.78 (dd, J = 10.4, 4.5 Hz, 3H), 2.51 (m, 2H), 2.33 (dd, J = 13.1, 10.2 Hz, 1H), 1.73 (m, 5H), 1.54 (dd, J = 13.8, 5.3 Hz, 3H); ^{13}C NMR (101 MHz, $CDCl_3$) δ 164.56, 150.53, 147.99, 147.59, 139.58, 137.28, 137.22, 130.51, 130.45, 126.00, 122.31, 121.91, 121.11, 119.36, 67.82, 49.92, 39.30, 34.05, 28.43, 26.20, 25.24. HRMS (ESI) m/z calculated for $C_{22}H_{26}F_3N_3O_2$ $[M+H]^+$ 422.2055, found 422.2060.

N-(3-(piperidin-1-yl)-4-(o-tolyl)butyl)picolinamide **(2d)**

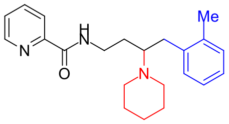

The title compound was isolated as a colorless oil (79% yield) after chromatography on alumina with ethyl acetate/hexane (1:3). ^{1}H NMR (400 MHz, $CDCl_3$) δ 9.32 (s, 1H), 8.52 (m, 1H), 7.80 (m, 1H), 7.38 (m, 1H), 7.18–7.05 (m, 4H), 3.72 (m, 1H), 3.27–3.14 (m, 1H), 3.06 (dd, J = 13.1, 3.0 Hz, 1H), 2.86 (m, 2H), 2.79 (m, 1H), 2.55 (m, 2H), 2.43–2.35 (m, 1H), 2.33 (s, 3H), 1.75 (m, 5H), 1.64–1.45 (m, 3H) ; ^{13}C NMR (101 MHz, $CDCl_3$) δ 164.46, 150.71, 147.91, 138.68, 137.20, 136.17, 130.56, 130.51, 126.19, 126.01, 125.88, 122.28, 66.72, 49.81, 39.76, 31.58, 28.00, 26.21, 25.36, 19.73. HRMS (ESI) m/z calculated for $C_{22}H_{29}N_3O$ $[M+H]^+$ 352.2389, found 352.2389.

N-(3-(piperidin-1-yl)-4-(p-tolyl)butyl)picolinamide **(2e)**

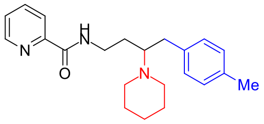

The title compound was isolated as a white solid (75% yield) after chromatography on alumina with ethyl acetate/hexane (1:3). ^{1}H NMR (400 MHz, $CDCl_3$) δ 9.33 (br, 1H), 8.52 (m, 1H), 8.16 (d, *J* = 7.8 Hz, 1H), 7.81 (m, 1H), 7.39 (m, 1H), 7.11–7.02 (m, 4H), 3.69 (dd, *J* = 13.3, 6.9 Hz, 1H), 3.24–3.14 (m, 1H), 3.02 (d, *J* = 12.5 Hz, 1H), 2.87–2.72 (m, 3H), 2.51 (s, 2H), 2.31 (s, 3H), 2.26 (t, *J* = 11.6 Hz, 1H), 1.74 (s, 6H), 1.55–1.48 (m, 2H); ^{13}C NMR (101 MHz, $CDCl_3$) δ 164.37, 150.56, 147.83, 147.64, 145.69, 137.09, 134.50, 125.78, 122.16, 122.00, 109.46, 109.41, 108.21, 100.81, 68.18, 49.78, 39.48, 34.21, 28.09, 26.12, 25.22. HRMS (ESI) m/z calculated for $C_{22}H_{29}N_3O$ $[M+H]^+$ 352.2389, found 352.2389.

N-(4-(naphthalen-2-yl)-3-(piperidin-1-yl)butyl)picolinamide **(2f)**

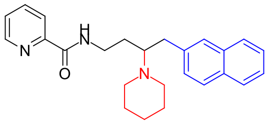

The title compound was isolated as a yellow solid (65% yield) after chromatography on alumina with ethyl acetate/hexane (1:3). ^{1}H NMR (400 MHz, $CDCl_3$) δ 9.13 (br, 1H), 8.51–8.46 (m, 1H), 8.14 (d, *J* = 7.8 Hz, 1H), 7.79–7.74 (m, 3H), 7.61 (s, 1H), 7.50–7.27 (m, 5H), 3.64 (m, 1H), 3.28 (dd, *J* = 13.1, 3.3 Hz, 1H), 3.21 (m, 1H), 3.06 (m, 1H), 2.91 (m, 2H), 2.66 (m, 2H), 2.54 (dd, *J* = 13.0, 10.3 Hz, 1H), 1.95–1.86 (m, 1H), 1.86–1.76 (m, 4H), 1.70–1.63 (m, 1H), 1.58–1.51 (m, 2H); ^{13}C NMR (101 MHz, $CDCl_3$) δ 164.38, 150.56, 147.83, 137.08, 133.61, 131.99, 128.06, 127.76, 127.66, 127.51, 127.37, 126.03, 125.78, 125.25, 122.16, 68.05, 49.89, 39.51, 34.71, 28.23, 26.13, 25.25. HRMS (ESI) m/z calculated for $C_{25}H_{29}N_3O$ $[M+H]^+$ 388.2389, found 388.2390.

N-(4-(benzo[d][1,3]dioxol-5-yl)-3-(piperidin-1-yl)butyl)picolinamide **(2g)**

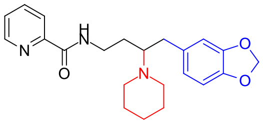

The title compound was isolated as a yellow solid (53% yield) after chromatography on alumina with ethyl acetate/hexane (1:3). ^{1}H NMR (400 MHz, $CDCl_3$) δ 9.28 (br, 1H), 8.52 (d, *J* = 4.4 Hz, 1H), 8.16 (d, *J* = 7.8 Hz, 1H), 7.81 (m, 1H), 7.39 (dd, *J* = 6.7, 4.9 Hz, 1H), 6.71 (d, *J* = 7.9 Hz, 1H), 6.62 (s, 1H), 6.58 (d, J = 7.9 Hz, 1H), 5.92 (s, 2H), 3.70 (m, 1H), 3.22 (m, 1H), 2.99–2.94 (m, 1H), 2.83–2.69 (m, 3H), 2.53–2.42 (m, 2H), 2.21 (dd, *J* = 13.0, 10.5 Hz, 1H), 1.77–1.67 (m, 5H), 1.60–1.48 (m, 3H); ^{13}C NMR (101 MHz, $CDCl_3$) δ 164.37, 150.56, 147.83, 147.64, 145.69, 137.09, 134.50, 125.78,

122.16, 122.00, 109.46, 109.41, 108.21, 100.81, 68.18, 49.78, 39.48, 34.21, 28.09, 26.12, 25.22. HRMS (ESI) m/z calculated for $C_{22}H_{27}N_3O_3$ $[M+H]^+$ 382.2131, found 382.2131.

***N*-(4-(3-(dimethylamino)phenyl)-3-(piperidin-1-yl)butyl)picolinamide** **(2h)**

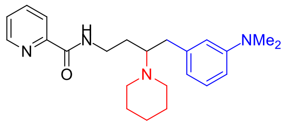

The title compound was isolated as a yellow oil (77% yield) after chromatography on alumina with ethyl acetate/hexane (1:3). ^{1}H NMR (400 MHz, $CDCl_3$) δ 9.35 (br, 1H), 8.52 (m, 1H), 8.16 (d, J = 7.8 Hz, 1H), 7.81 (m, 1H), 7.38 (m, 1H), 7.14 (t, J = 7.9 Hz, 1H), 6.60–6.55 (m, 1H), 6.53 (d, J = 6.8 Hz, 2H), 3.71 (m, 1H), 3.20 (m, 1H), 3.05 (dd, J = 12.9, 3.0 Hz, 1H), 2.93 (s, 6H), 2.84 (m, 3H), 2.59–2.49 (m, 2H), 2.26 (dd, J = 12.8, 10.6 Hz, 1H), 1.83–1.71 (m, 5H), 1.66–1.49 (m, 3H); ^{13}C NMR (101 MHz, $CDCl_3$) δ 164.54, 150.85, 150.61, 147.92, 141.50, 137.18, 129.18, 125.88, 122.25, 117.71, 113.62, 110.41, 68.20, 49.89, 40.72, 39.76, 34.98, 28.18, 26.03, 25.23. HRMS (ESI) m/z calculated for $C_{23}H_{32}N_4O$ $[M+H]^+$ 381.2654, found 381.2660.

***N*-(3-(piperidin-1-yl)-4-(4-(trifluoromethyl)phenyl)butyl)picolinamide** **(2i)**

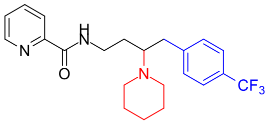

The title compound was isolated as a white solid (74% yield) after chromatography on alumina with ethyl acetate/hexane (1:3). ^{1}H NMR (400 MHz, $CDCl_3$) δ 9.07 (br, 1H), 8.49–8.37 (m, 1H), 8.08 (d, J = 7.8 Hz, 1H), 7.73 (m, 1H), 7.44 (d, J = 8.0 Hz, 2H), 7.32 (m, 1H), 7.21–7.15 (m, 2H), 3.60 (m, 1H), 3.17 (m, 1H), 3.01 (dd, J = 13.0, 3.2 Hz, 1H), 2.72 (m, 3H), 2.44 (m, 2H), 2.31 (dd, J = 13.0, 10.1 Hz, 1H), 1.75–1.61 (m, 5H), 1.49–1.39 (m, 3H); ^{13}C NMR (101 MHz, $CDCl_3$) δ 164.57, 150.39, 147.98, 145.06, 137.28, 129.58, 128.51, 128.18, 126.03, 125.76, 125.46, 125.42, 123.06, 122.26, 67.72, 49.93, 39.25, 34.64, 28.42, 26.12, 25.17. HRMS (ESI) m/z calculated for $C_{22}H_{26}F_3N_3O$ $[M+H]^+$ 406.2106, found 406.2110.

***N*-(4-(4-fluorophenyl)-3-(piperidin-1-yl)butyl)picolinamide** **(2j)**

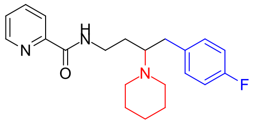

The title compound was isolated as a white solid (72% yield) after chromatography on alumina with ethyl acetate/hexane (1:3). ^{1}H NMR (400 MHz, $CDCl_3$) δ 9.06 (br, 1H), 8.51 (d, J = 4.3 Hz, 1H), 8.15 (d, J = 7.8 Hz, 1H), 7.81 (m, 1H), 7.40–7.36 (m, 1H), 7.11 (dd, J = 8.4, 5.5 Hz, 2H), 6.95 (t, J = 8.7 Hz, 2H), 3.63

(m, 1H), 3.23 (m, 1H), 3.07 (dd, J = 13.2, 3.3 Hz, 1H), 2.85 (m, 3H), 2.57 (m, 2H), 2.34 (dd, J = 13.2, 10.2 Hz, 1H), 1.85–1.70 (m, 5H), 1.56 (m, 3H); ^{13}C NMR (101 MHz, $CDCl_3$) δ 164.36, 162.50, 160.08, 150.52, 147.82, 137.10, 136.35, 130.50, 130.42, 125.79, 122.16, 115.29, 115.08, 67.89, 49.80, 39.28, 33.80, 28.27, 26.14, 25.19. HRMS (ESI) m/z calculated for $C_{21}H_{26}FN_3O$ $[M+H]^+$ 356.2138, found 356.2138.

N-(4-(4-chlorophenyl)-3-(piperidin-1-yl)butyl)picolinamide **(2k)**

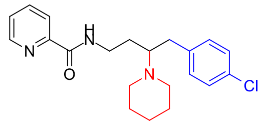

The title compound was isolated as a white solid (79% yield) after chromatography on alumina with ethyl acetate/hexane (1:3). ^{1}H NMR (400 MHz, $CDCl_3$) δ 9.14 (br, 1H), 8.55–8.48 (m, 1H), 8.16 (d, J = 7.8 Hz, 1H), 7.81 (m, 1H), 7.39 (m, 1H), 7.23 (d, J = 8.3 Hz, 2H), 7.06 (d, J = 8.3 Hz, 2H), 3.66 (m, 1H), 3.23 (m, 1H), 3.00 (m, 1H), 2.77 (m, 3H), 2.50 (m, 2H), 2.29 (m, 1H), 1.73 (m, 5H), 1.59–1.48 (m, 3H); ^{13}C NMR (101 MHz, $CDCl_3$) δ 164.58, 150.50, 147.99, 139.22, 137.28, 131.79, 130.63, 128.68, 126.00, 122.29, 67.89, 49.95, 39.36, 34.13, 28.39, 26.15, 25.22. HRMS (ESI) m/z calculated for $C_{21}H_{26}ClN_3O$ $[M+H]^+$ 372.1843, found 372.1842.

N-(4-(3-bromophenyl)-3-(piperidin-1-yl)butyl)picolinamide **(2l)**

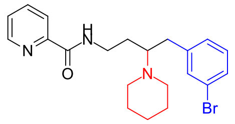

The title compound was isolated as a colorless oil (80% yield) after chromatography on alumina with ethyl acetate/hexane (1:3). ^{1}H NMR (400 MHz, $CDCl_3$) δ 9.17 (br, 1H), 8.52 (m, 1H), 8.16 (d, J = 7.8 Hz, 1H), 7.81 (m, 1H), 7.39 (m, 1H), 7.31 (d, J = 8.7 Hz, 2H), 7.13 (t, J = 7.7 Hz, 1H), 7.06 (d, J = 7.6 Hz, 1H), 3.69 (m, 1H), 3.24 (m, 1H), 3.02 (d, J = 12.5 Hz, 1H), 2.88–2.66 (m, 3H), 2.50 (s, 2H), 2.35–2.22 (m, 1H), 1.73 (s, 6H), 1.54–1.48 (m, 2H); ^{13}C NMR (101 MHz, $CDCl_3$) δ 164.53, 150.50, 147.97, 137.30, 137.26, 132.24, 130.09, 129.15, 127.99, 125.98, 122.61, 122.28, 67.85, 49.91, 39.35, 34.42, 28.34, 26.14, 25.22. HRMS (ESI) m/z calculated for $C_{21}H_{26}BrN_3O$ $[M+H]^+$ 416.1338, found 416.1337.

N-(4-(4-iodophenyl)-3-(piperidin-1-yl)butyl)picolinamide **(2m)**

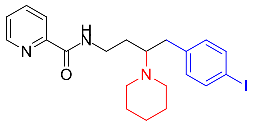

The title compound was isolated as a yellow solid (64% yield) after chromatography on alumina with ethyl acetate/hexane (1:3). ^{1}H NMR (400

MHz, $CDCl_3$) δ 9.14 (s, 1H), 8.56–8.44 (m, 2H), 8.15 (d, J = 7.8 Hz, 2H), 7.79 (m, 2H), 7.61–7.53 (m, 3H), 7.37 (m, 2H), 6.88 (d, J = 8.2 Hz, 3H), 3.67 (m, 2H), 3.22 (m, 2H), 2.95 (m, 2H), 2.74 (m, 5H), 2.46 (m, 3H), 2.23 (dd, J = 13.1, 10.1 Hz, 2H), 1.70 (h, J = 5.2, 4.3 Hz, 8H), 1.58–1.42 (m, 5H); ^{13}C NMR (101 MHz, $CDCl_3$) δ 164.37, 150.42, 147.86, 140.42, 137.45, 137.15, 131.33, 131.29, 125.87, 122.16, 90.95, 67.77, 49.81, 39.25, 34.19, 28.23, 26.04, 25.13. HRMS (ESI) m/z calculated for $C_{21}H_{26}IN_3O$ $[M+H]^+$ 464.1199, found 464.1197.

***N-(3-(piperidin-1-yl)-4-(4-vinylphenyl)butyl)picolinamide* (2n)**

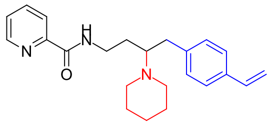

The title compound was isolated as a colorless oil (57% yield) after chromatography on alumina with ethyl acetate/hexane (1:3). ^1H NMR (400 MHz, $CDCl_3$) δ 9.28 (br, 1H), 8.52 (d, J = 4.1 Hz, 1H), 8.16 (d, J = 7.8 Hz, 1H), 7.81 (m, 1H), 7.41–7.37 (m, 1H), 7.32 (d, J = 8.0 Hz, 2H), 7.11 (d, J = 8.0 Hz, 2H), 6.68 (m, 1H), 5.70 (d, 1H), 5.20 (d, J = 11.2 Hz, 1H), 3.68 (m, 1H), 3.20 (m, 1H), 3.06 (m, 1H), 2.87–2.75 (m, 3H), 2.58–2.46 (m, 2H), 2.35–2.27 (m, 1H), 1.81–1.69 (m, 5H), 1.61–1.50 (m, 3H); ^{13}C NMR (101 MHz, $CDCl_3$) δ 164.38, 150.53, 147.83, 140.50, 137.10, 136.59, 135.32, 129.39, 126.31, 125.80, 122.16, 113.14, 68.11, 49.82, 39.49, 34.28, 28.15, 26.06, 25.19. HRMS (ESI) m/z calculated for $C_{23}H_{29}N_3O$ $[M+H]^+$ 364.2389, found 364.2389.

***N-(4-(3-formylphenyl)-3-(piperidin-1-yl)butyl)picolinamide* (2o)**

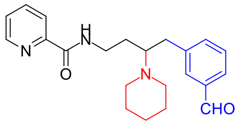

The title compound was isolated as a blue oil (50% yield) after chromatography on alumina with ethyl acetate/hexane (1:3). ^1H NMR (400 MHz, $CDCl_3$) δ 9.97 (br, 1H), 9.00 (br, 1H), 8.50 (d, J = 4.6 Hz, 1H), 8.14 (d, J = 7.8 Hz, 1H), 7.80 (m, 1H), 7.69 (d, J = 4.4 Hz, 2H), 7.44 (d, J = 4.8 Hz, 2H), 7.40–7.37 (m, 1H), 3.62 (dd, J = 13.4, 6.7 Hz, 1H), 3.26 (m, 1H), 3.21–3.16 (m, 1H), 2.91 (d, J = 9.4 Hz, 1H), 2.83 (dd, J = 10.5, 5.2 Hz, 2H), 2.65–2.57 (m, 2H), 2.48 (dd, J = 12.8, 10.3 Hz, 1H), 1.87 (dd, J = 14.5, 5.9 Hz, 1H), 1.76 (d, J = 5.1 Hz, 4H), 1.58–1.49 (m, 3H); ^{13}C NMR (101 MHz, $CDCl_3$) δ 192.57, 164.63, 150.29, 148.03, 137.32, 136.76, 135.57, 130.11, 130.06, 129.31, 128.10, 126.09, 122.28, 67.30, 49.94, 39.05, 34.85, 28.49, 25.82, 24.93. HRMS (ESI) m/z calculated for $C_{23}H_{29}N_3O$ $[M+H]^+$ 366.2182, found 366.2185.

***N-(4-(4-acetylphenyl)-3-(piperidin-1-yl)butyl)picolinamide* (2p)**

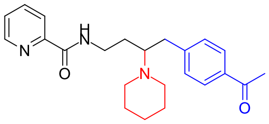

The title compound was isolated as a white solid (76% yield) after chromatography on alumina with ethyl acetate/hexane (1:3). 1H NMR (400 MHz, $CDCl_3$) δ 9.10 (s, 1H), 8.51 (dd, J = 4.8, 2.1 Hz, 1H), 8.15 (d, J = 7.8 Hz, 1H), 7.86 (dd, J = 8.2, 1.9 Hz, 2H), 7.81 (m, 1H), 7.38 (m, 1H), 7.25–7.19 (m, 2H), 3.66 (m, 1H), 3.23 (m, 1H), 3.08 (dd, J = 13.3, 3.6 Hz, 1H), 2.78 (m, 3H), 2.57 (d, J = 2.3 Hz, 3H), 2.50 (p, J = 5.1 Hz, 2H), 2.39 (dd, J = 12.8, 10.1 Hz, 1H), 1.84–1.63 (m, 6H), 1.52 (m, 3H); 13C NMR (101 MHz, $CDCl_3$) δ 197.74, 164.36, 150.40, 147.85, 146.82, 137.14, 135.11, 129.41, 128.59, 125.86, 122.15, 67.66, 49.82, 39.12, 34.81, 28.50, 26.59, 26.51, 26.08, 25.12. HRMS (ESI) m/z calculated for $C_{23}H_{29}N_3O_2$ $[M+H]^+$ 380.2338, found: 380.2431.

***(E)-N-(6-phenyl-3-(piperidin-1-yl)hex-5-en-1-yl)picolinamide* (2q)**

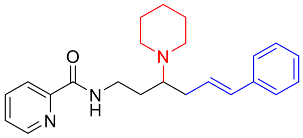

The title compound was isolated as a blue oil (46% yield) after chromatography on alumina with ethyl acetate/hexane (1:3). ^{1}H NMR (400 MHz, $CDCl_3$) δ 9.27 (s, 1H), 8.61–8.36 (m, 1H), 8.16 (d, J = 7.8 Hz, 1H), 7.81 (m, 1H), 7.39 (m, 1H), 7.34–7.30 (m, 2H), 7.10 (d, J = 7.9 Hz, 2H), 6.69 (m, 1H), 5.70 (d, 1H), 5.20 (d, J = 10.9 Hz, 1H), 3.70 (m, 1H), 3.21 (m, 1H), 3.04 (dd, J = 13.1, 3.4 Hz, 1H), 2.81 (m, 3H), 2.50 (m, 2H), 2.29 (dd, J = 13.1, 10.3 Hz, 1H), 1.74 (t, J = 5.6 Hz, 6H), 1.64–1.45 (m, 3H); ^{13}C NMR (101 MHz, $CDCl_3$) δ 164.37, 150.57, 147.82, 140.53, 137.08, 136.61, 135.33, 129.37, 126.30, 125.77, 122.17, 113.11, 68.02, 49.83, 39.44, 34.29, 28.26, 26.10, 25.20. HRMS (ESI) m/z calculated for $C_{23}H_{29}N_3O_2$ $[M+H]^+$ 363.2311, found: 363.2318.

***N-(3-(diethylamino)-4-phenylbutyl)picolinamide* (3a)**

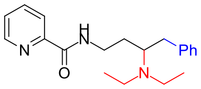

The title compound was isolated as a yellow oil (70% yield) after chromatography on alumina with ethyl acetate/hexane (1:3). ^{1}H NMR (400 MHz, $CDCl_3$) δ 9.02 (br, 1H), 8.54–8.47 (m, 1H), 8.14 (d, *J* = 7.8 Hz, 1H), 7.79 (m, 1H), 7.36 (m, 1H), 7.28–7.23 (m, 2H), 7.15 (m, 3H), 3.59 (m, 1H), 3.35–3.24 (m, 1H), 2.97 (d, *J* = 10.5 Hz, 2H), 2.76 (m, 2H), 2.47 (dd, *J* = 12.8, 6.6 Hz, 2H), 2.31 (t, *J* = 12.3 Hz, 1H), 1.75–1.65 (m, 1H), 1.60 (m, 1H), 1.14 (t, *J* = 7.1 Hz, 6H); ^{13}C NMR (101 MHz, $CDCl_3$) δ 164.37, 150.57, 148.01, 137.22,

137.16, 129.32, 128.56, 125.98, 125.85, 122.20, 62.15, 43.46, 39.14, 35.18, 29.23, 14.41. HRMS (ESI) m/z calculated for $C_{20}H_{27}N_3O$ $[M+H]^+$ 326.2232, found: 326.2240.

N-(3-(benzyl(methyl)amino)-4-phenylbutyl)picolinamide **(3b)**

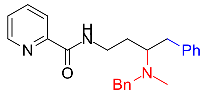

The title compound was isolated as a yellow oil (75% yield) after chromatography on alumina with ethyl acetate/hexane (1:3). ^1H NMR (400 MHz, $CDCl_3$) δ 8.73 (br, 1H), 8.47–8.42 (m, 1H), 8.16 (d, *J* = 7.8 Hz, 1H), 7.81 (m, 1H), 7.40–7.36 (m, 3H), 7.29 (d, *J* = 6.9 Hz, 2H), 7.25–7.20 (m, 3H), 7.17 (dd, *J* = 8.4, 6.3 Hz, 1H), 7.12 (d, *J* = 7.0 Hz, 2H), 3.82 (d, *J* = 13.2 Hz, 1H), 3.70 (d, *J* = 13.2 Hz, 1H), 3.57 (m, 1H), 3.40–3.32 (m, 1H), 3.08 (dd, *J* = 13.0, 3.9 Hz, 1H), 2.94 (m, 1H), 2.40 (dd, *J* = 13.0, 9.8 Hz, 1H), 2.32 (s, 3H), 1.79 (m, 1H), 1.61 (m, 1H); ^{13}C NMR (101 MHz, $CDCl_3$) δ 164.25, 150.43, 148.12, 140.44, 137.25, 129.34, 129.09, 128.54, 128.39, 127.08, 126.96, 126.03, 125.91, 122.19, 63.71, 59.19, 38.34, 35.86, 34.26, 29.93. HRMS (ESI) m/z calculated for $C_{24}H_{27}N_3O$ $[M+H]^+$ 374.2232, found: 374.2232.

N-(3-morpholino-4-phenylbutyl)picolinamide **(3c)**

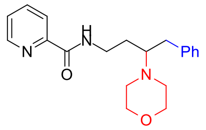

The title compound was isolated as a yellow oil (82% yield) after chromatography on alumina with ethyl acetate/hexane (1:3). ^1H NMR (400 MHz, $CDCl_3$) δ 9.09 (br, 1H), 8.59–8.52 (m, 1H), 8.17 (d, *J* = 7.8 Hz, 1H), 7.83 (m, 1H), 7.42 (s, 1H), 7.28 (t, *J* = 7.4 Hz, 2H), 7.20 (d, *J* = 7.3 Hz, 1H), 7.15 (d, *J* = 7.1 Hz, 2H), 3.89 (t, *J* = 4.4 Hz, 4H), 3.76–3.65 (m, 2H), 3.27 (m, 1H), 3.10 (d, *J* = 13.1 Hz, 1H), 2.87 (dd, *J* = 10.3, 5.3 Hz, 2H), 2.68–2.58 (m, 2H), 2.42–2.32 (m, 1H), 1.84–1.70 (m, 1H), 1.64 (m, 1H); 13C NMR (101 MHz, $CDCl_3$) δ 164.28, 150.31, 147.95, 140.13, 137.24, 129.18, 128.57, 128.53, 126.07, 125.97, 122.21, 67.50, 67.47, 67.14, 48.89, 39.12, 39.07, 34.56, 28.19. HRMS (ESI) m/z calculated for $C_{20}H_{25}N_3O_2$ $[M+H]^+$ 340.2025, found: 340.2030.

N-(4-phenyl-3-thiomorpholinobutyl)picolinamide **(3d)**

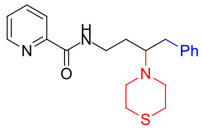

The title compound was isolated as a colorless oil (60% yield) after chromatography on alumina with ethyl acetate/hexane (1:3). ^1H NMR (400 MHz, $CDCl_3$) δ 9.33 (br, 1H), 8.66–8.62 (m, 1H), 8.18 (d, *J* = 7.8 Hz, 1H),

8.15–8.05 (m, 1H), 7.83 (m, 1H), 7.42 (m, 1H), 7.28 (s, 1H), 7.20 (t, *J* = 7.4 Hz, 1H), 7.14 (d, *J* = 7.0 Hz, 2H), 3.76–3.67 (m, 1H), 3.18 (m, 3H), 3.06 (dd, *J* = 13.1, 3.6 Hz, 1H), 2.88 (s, 7H), 2.35 (dd, *J* = 13.1, 10.3 Hz, 1H), 1.77 (m, 1H), 1.59 (m, 1H); ^{13}C NMR (101 MHz, $CDCl_3$) δ 164.52, 150.25, 148.11, 137.43, 130.21, 129.30, 128.67, 128.51, 126.18, 122.33, 69.59, 51.61, 39.67, 35.16, 35.13, 27.98. HRMS (ESI) m/z calculated for $C_{20}H_{25}N_3OS$ $[M+H]^+$ 356.1797, found: 356.1796.

tert-butyl 4-(1-phenyl-4-(picolinamido)butan-2-yl)piperazine-1-carboxylate (3e)

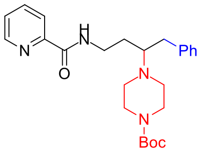

The title compound was isolated as a white solid (61% yield) after chromatography on alumina with ethyl acetate/hexane (1:3). ^{1}H NMR (400 MHz, $CDCl_3$) δ 9.16 (br, 1H), 8.50–8.43 (m, 1H), 8.15 (d, *J* = 7.8 Hz, 1H), 7.80 (m, 1H), 7.42–7.34 (m, 2H), 7.24 (d, *J* = 1.5 Hz, 1H), 7.20–7.14 (m, 1H), 7.14–7.08 (m, 2H), 3.75–3.50 (m, 5H), 3.24 (m, 1H), 3.00 (dd, *J* = 13.1, 3.5 Hz, 1H), 2.94–2.66 (m, 3H), 2.62–2.43 (m, 2H), 2.31 (dd, *J* = 13.0, 10.2 Hz, 1H), 1.76 (dd, *J* = 10.0, 4.9 Hz, 1H), 1.61 (m, 1H), 1.47 (s, 9H); ^{13}C NMR (101 MHz, $CDCl_3$) δ 164.58, 155.06, 150.13, 148.02, 137.47, 137.42, 129.23, 128.66, 126.25, 126.19, 122.34, 79.85, 67.48, 48.46, 48.38, 39.31, 34.78, 28.55, 28.35. HRMS (ESI) m/z calculated for $C_{25}H_{34}N_4O_3$ $[M+H]^+$ 439.2709, found: 439.2709.

***ethyl 1-(1-phenyl-4-(picolinamido)butan-2-yl)piperidine-4-carboxylate* (3f)**

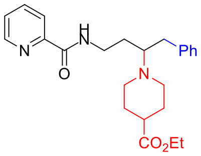

The title compound was isolated as a colorless oil (70% yield) after chromatography on alumina with ethyl acetate/hexane (1:3). ^{1}H NMR (400 MHz, $CDCl_3$) δ 9.47–9.33 (m, 1H), 8.63 (d, *J* = 4.1 Hz, 1H), 8.16 (d, *J* = 7.8 Hz, 1H), 7.80 (m, 1H), 7.39 (m, 1H), 7.28 (d, *J* = 7.5 Hz, 2H), 7.19 (t, *J* = 7.3 Hz, 1H), 7.14 (d, *J* = 7.1 Hz, 2H), 4.19 (m, 2H), 3.84–3.71 (m, 1H), 3.19–3.01 (m, 3H), 2.94–2.82 (m, 2H), 2.73 (m, 1H), 2.39–2.24 (m, 3H), 2.09 (m, 2H), 1.95 (dd, 2H), 1.75 (m, 1H), 1.61 (d, *J* = 3.1 Hz, 1H), 1.29 (t, *J* = 7.1 Hz, 3H); ^{13}C NMR (101 MHz, $CDCl_3$) δ 175.59, 164.51, 150.50, 148.37, 140.50, 137.12, 129.29, 128.60, 126.09, 125.93, 122.14, 68.35, 60.38, 51.77, 45.17, 42.12, 34.57, 28.84, 27.96, 14.41. HRMS (ESI) m/z calculated for $C_{24}H_{31}N_3O_3$ $[M+H]^+$ 410.2444, found: 410.2444.

***N-(4-phenyl-3-(1,4-dioxa-8-azaspiro[4.5]decan-8-yl)butyl)picolinamide* (3g)**

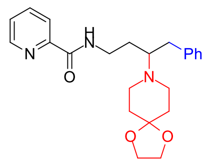

The title compound was isolated as a white solid (69% yield) after chromatography on alumina with ethyl acetate/hexane (1:3). ^{1}H NMR (400 MHz, $CDCl_3$) δ 9.76–9.62 (m, 1H), 8.64 (d, *J* = 4.2 Hz, 1H), 8.16 (d, *J* = 7.8 Hz, 1H), 7.80 (m, 1H), 7.42–7.36 (m, 1H), 7.29 (t, *J* = 7.6 Hz, 2H), 7.19 (t, *J* = 7.3 Hz, 1H), 7.14 (d, *J* = 7.2 Hz, 2H), 4.00 (s, 4H), 3.83–3.76 (m, 1H), 3.15–3.05 (m, 2H), 2.99 (m, 2H), 2.89 (t, *J* = 10.6 Hz, 1H), 2.71–2.58 (m, 2H), 2.31 (m, 1H), 2.07–1.93 (m, 4H), 1.73 (m, 1H), 1.60 (m, 1H); ^{13}C NMR (101 MHz, $CDCl_3$) δ 164.51, 150.54, 148.26, 140.53, 137.15, 129.31, 128.61, 126.07, 125.96, 122.11, 108.02, 68.41, 64.37, 40.17, 40.12, 34.98, 34.61, 27.59. HRMS (ESI) m/z calculated for $C_{23}H_{29}N_3O_3$ $[M+H]^+$ 396.2287, found: 396.2287.

***N*-(3-(azepan-1-yl)-4-phenylbutyl)picolinamide** (3h)

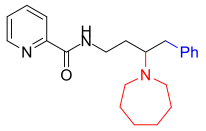

The title compound was isolated as a colorless oil (70% yield) after chromatography on alumina with ethyl acetate/hexane (1:3). ^{1}H NMR (400 MHz, $CDCl_3$) δ 8.73 (br, 1H), 8.55–8.49 (m, 1H), 8.16 (d, *J* = 7.8 Hz, 1H), 7.81 (m, 1H), 7.39 (m, 1H), 7.26 (d, *J* = 14.6 Hz, 2H), 7.19–7.12 (m, 3H), 3.55 (m, 1H), 3.42 (m, 1H), 2.98 (m, 1H), 2.92–2.84 (m, 3H), 2.67 (m, 2H), 2.39 (m, 1H), 1.71 (m, 6H), 1.66–1.60 (m, 4H); ^{13}C NMR (101 MHz, $CDCl_3$) δ 164.47, 150.44, 148.02, 137.28, 137.21, 129.31, 128.49, 128.42, 125.96, 122.27, 67.99, 51.75, 38.89, 35.73, 30.25, 29.43, 27.03. HRMS (ESI) m/z calculated for $C_{22}H_{29}N_3O$ $[M+H]^+$ 352.2389, found: 352.2391.

***N*-(3-(tert-butylamino)-4-phenylbutyl)picolinamide** (3i)

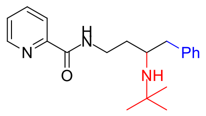

The title compound was isolated as a yellow oil (76% yield) after chromatography on alumina with ethyl acetate/hexane (1:3). ^{1}H NMR (400 MHz, $CDCl_3$) δ 9.19 (s, 1H), 8.45 (m, 1H), 8.11 (m, 1H), 7.74 (m, 1H), 7.34–7.27 (m, 1H), 7.26–7.17 (m, 2H), 7.13 (m, 3H), 3.59–3.42 (m, 2H), 3.00 (m, 1H), 2.73 (dd, J = 13.3, 6.2 Hz, 1H), 2.61 (dd, J = 13.3, 7.3 Hz, 1H), 1.73 (m, 1H), 1.56–1.43 (m, 1H), 1.18 (s, 1H), 1.00 (s, 10H); ^{13}C NMR (101 MHz, $CDCl_3$) δ 164.28, 150.61, 148.11, 139.46, 137.26, 129.53, 128.56, 126.44, 125.91, 122.28, 53.04, 51.34, 43.84, 37.46, 34.99, 29.95. HRMS (ESI) m/z calculated for $C_{20}H_{27}N_3O$ $[M+H]^+$ 326.2232, found: 326.2333.

***N*-(4-phenyl-3-((2-phenylpropan-2-yl)amino)butyl)picolinamide** (**3j**)

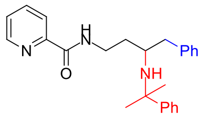

The title compound was isolated as a yellow oil (68% yield) after chromatography on alumina with ethyl acetate/hexane (1:3). ^{1}H NMR (400 MHz, $CDCl_3$) δ 8.99 (br, 1H), 8.55 (m, 1H), 8.20 (d, J = 7.8 Hz, 1H), 7.84 (m, 1H), 7.43 (m, 2H), 7.42–7.38 (m, 1H), 7.30–7.24 (m, 3H), 7.22–7.16 (m, 3H), 7.03–6.88 (m, 2H), 3.62–3.36 (m, 2H), 2.91–2.80 (m, 1H), 2.49 (m, 2H), 1.58 (m, 1H), 1.51 (d, J = 10.1 Hz, 6H), 1.46–1.39 (m, 1H); ^{13}C NMR (101 MHz, $CDCl_3$) δ 164.26, 150.56, 148.10, 147.80, 139.40, 137.30, 129.39, 128.47, 128.15, 126.54, 126.25, 126.20, 125.95, 122.28, 56.00, 53.42, 42.77, 36.85, 33.94, 30.46. HRMS (ESI) m/z calculated for $C_{25}H_{29}N_3O$ $[M+H]^+$ 388.2389, found: 388.2389.

***N*-(4-phenyl-3-((2,4,4-trimethylpentan-2-yl)amino)butyl)picolinamide** (**3k**)

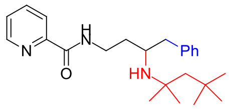

The title compound was isolated as a colorless oil (80% yield) after chromatography on alumina with ethyl acetate/hexane (1:3). ^{1}H NMR (400 MHz, $CDCl_3$) δ 8.99 (br, 1H), 8.46 (d, J = 4.7 Hz, 1H), 8.11 (d, J = 7.8 Hz, 1H), 7.75 (m, 1H), 7.41–7.24 (m, 2H), 7.22–7.18 (m, 2H), 7.15–7.09 (m, 3H), 3.55–3.42 (m, 2H), 3.04 (m, 1H), 2.71 (m, 1H), 2.63 (dd, J = 13.3, 7.3 Hz, 1H), 1.77–1.70 (m, 1H), 1.53 (dd, J = 13.9, 6.3 Hz, 1H), 1.41 (d, J = 14.4 Hz, 1H), 1.28 (d, J = 14.3 Hz, 1H), 1.19 (d, J = 6.0 Hz, 1H), 1.07 (s, 3H), 1.00 (s, 3H), 0.88 (s, 9H); ^{13}C NMR (101 MHz, $CDCl_3$) δ 164.42, 150.37, 148.11, 139.35, 137.29, 129.51, 128.56, 126.47, 126.00, 122.28, 55.96, 52.32, 43.55, 37.10, 35.23, 32.02, 31.60, 29.05, 27.90. HRMS (ESI) m/z calculated for $C_{24}H_{35}N_3O$ $[M+H]^+$ 382.2858, found: 388.2866.

***N*-(3-(((3s,5s,7s)-adamantan-1-yl)amino)-4-phenylbutyl)picolinamide** (**3l**)

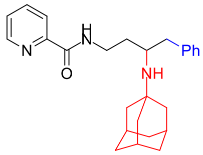

The title compound was isolated as a yellow oil (61% yield) after chromatography on alumina with ethyl acetate/hexane (1:3). ^{1}H NMR (400 MHz, $CDCl_3$) δ 9.24 (s, 1H), 8.53 (d, J = 4.8 Hz, 2H), 7.82 (t, J = 7.7 Hz, 2H), 7.39 (dd, J = 7.6, 4.8 Hz, 2H), 7.34–7.24 (m, 6H), 7.21 (t, J = 8.5 Hz, 7H), 3.57 (m, 4H), 3.27–3.14 (m, 2H), 2.81 (dd, J = 13.3, 6.0 Hz, 2H), 2.67 (dd, J = 13.3, 7.4 Hz, 2H), 2.07–1.97 (m, 8H), 1.84–1.73 (m, 2H), 1.63 (d, J = 12.2 Hz, 21H), 1.55 (d, J = 13.4 Hz, 10H), 1.33–1.17 (m, 2H).; ^{13}C NMR (101 MHz, $CDCl_3$) δ 164.12, 150.51, 147.96, 139.35, 137.17, 129.45, 128.42, 126.30,

125.81, 122.19, 51.33, 50.75, 44.22, 43.56, 37.48, 36.65, 35.15, 29.65. HRMS (ESI) m/z calculated for $C_{26}H_{33}N_3O$ $[M+H]^+$ 404.2702, found: 404.2702.

***N-(5-phenyl-4-(piperidin-1-yl)pentan-2-yl)picolinamide* (4a)**

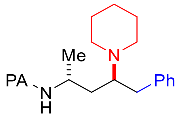

The title compound was isolated as a colorless oil (51% yield) after chromatography on alumina with ethyl acetate/hexane (1:3). This product was isolated as a >20:1 mixture of diastereomers. The reported dr was determined by 1H NMR analysis. ^{1}H NMR (400 MHz, $CDCl_3$) δ 9.70 (d, J = 7.4 Hz, 1H), 8.52 (d, J = 4.2 Hz, 1H), 8.17 (d, J = 7.8 Hz, 1H), 7.81 (m, 1H), 7.41–7.36 (m, 1H), 7.27 (d, J = 6.1 Hz, 2H), 7.17 (dd, 3H), 4.30 (m, 1H), 3.05 (t, J = 12.4 Hz, 2H), 2.83 (m, 2H), 2.62–2.42 (m, 2H), 2.32–2.20 (m, 1H), 1.88 (m, 1H), 1.79–1.74 (m, 3H), 1.56–1.49 (m, 2H), 1.40–1.34 (m, 1H), 1.27–1.25 (m, 1H), 1.08 (d, J = 6.7 Hz, 3H); ^{13}C NMR (101 MHz, $CDCl_3$) δ 164.69, 150.67, 148.06, 141.80, 137.21, 128.99, 128.41, 125.95, 125.88, 122.40, 71.50, 52.32, 46.59, 34.55, 26.20, 24.94, 21.63, 12.44. HRMS (ESI) m/z calculated for $C_{22}H_{29}N_3O$ $[M+H]^+$ 352.2389, found: 352.2390.

***N-(1,4-diphenyl-3-(piperidin-1-yl)butyl)picolinamide* (4b)**

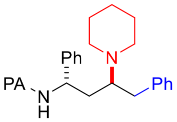

The title compound was isolated as a yellow solid (41% yield) after chromatography on alumina with ethyl acetate/hexane (1:3). This product was isolated as a >20:1 mixture of diastereomers. The reported dr was determined by 1H NMR analysis. ^{1}H NMR (400 MHz, $CDCl_3$) δ 10.45 (d, J = 7.6 Hz, 1H), 8.59 (d, J = 4.4 Hz, 1H), 8.17 (d, J = 7.8 Hz, 1H), 7.83 (m, 1H), 7.43 (dd, J = 6.7, 4.8 Hz, 1H), 7.14 (d, J = 6.3 Hz, 6H), 6.99 (d, J = 6.4 Hz, 2H), 6.86–6.77 (m, 2H), 5.37 (m, 1H), 2.94 (dd, J = 13.2, 3.0 Hz, 1H), 2.85–2.74 (m, 2H), 2.69 (t, J = 10.4 Hz, 1H), 2.49–2.37 (m, 2H), 2.25 (dd, J = 13.0, 11.2 Hz, 1H), 2.11 (m, 1H), 1.93–1.78 (m, 4H), 1.76–1.70 (m, 1H), 1.57 (d, J = 5.5 Hz, 2H); ^{13}C NMR (101 MHz, $CDCl_3$) δ 164.40, 150.74, 147.93, 141.98, 140.16, 137.17, 129.02, 128.35, 128.24, 126.41, 126.03, 125.96, 125.78, 122.56, 62.94, 52.36, 49.99, 33.57, 33.44, 26.03, 25.49. HRMS (ESI) m/z calculated for $C_{27}H_{31}N_3O$ $[M+H]^+$ 414.2545, found: 414.2545.

***N-(2-(2-phenyl-1-(piperidin-1-yl)ethyl)hexyl)picolinamide* (4c)**

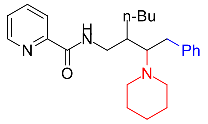

The title compound was isolated as a colorless oil (62% yield) after chromatography on alumina with ethyl acetate/hexane (1:3). This product was isolated as a 1.9:1 mixture of diastereomers. The reported dr was determined by ^{1}H NMR analysis. The following analytical data correspond to the mixture. ^{1}H NMR (400 MHz, $CDCl_3$) δ 9.56 (s, 0.35H), 8.89 (s, 0.65H), 8.54 (d, J = 4.7 Hz, 0.35H), 8.51 (d, J = 4.2 Hz, 0.65H), 8.17 (d, J = 7.9 Hz, 2H), 7.82 (m, 1H), 7.47 (s, 1H), 7.41–7.38 (m, 1H), 7.23–7.16 (m, 3H), 3.90 (dd, J = 13.6, 7.3 Hz, 0.36H), 3.61–3.53 (m, 0.65H), 3.45–3.36 (m, 0.65H), 3.18–3.12 (m, 0.35H), 3.06–2.98 (m, 1H), 2.98–2.75 (m, 2H), 2.74–2.53 (m, 4H), 1.75 (dd, J = 10.7, 5.8 Hz, 3H), 1.52–1.40 (m, 3H), 1.38–1.20 (m, 7H), 0.89–0.84 (m, 2H), 0.82 (d, J = 7.1 Hz, 1H); ^{13}C NMR (101 MHz, $CDCl_3$) δ 164.65, 164.47, 164.19, 150.66, 147.93, 147.79, 142.31, 141.30, 137.31, 137.09, 137.03, 129.08, 128.40, 128.25, 125.99, 125.79, 125.68, 125.62, 122.22, 122.16, 71.50, 69.52, 52.73, 51.04, 42.38, 41.83, 40.76, 40.71, 38.90, 34.28, 32.24, 30.13, 30.11, 29.69, 29.45, 26.63, 26.47, 26.24, 24.97, 22.90, 22.68, 14.00. HRMS (ESI) m/z calculated for $C_{25}H_{35}N_3O$ $[M+H]^+$ 394.2858, found: 394.2859.

N-((1-(2-phenyl-1-(piperidin-1-yl)ethyl)cyclopentyl)methyl)picolinamide **(4d)**

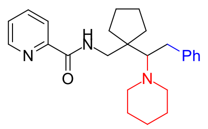

The title compound was isolated as a colorless oil (54% yield) after chromatography on alumina with ethyl acetate/hexane (1:3). ^{1}H NMR (400 MHz, $CDCl_3$) δ 8.99 (s, 1H), 8.47 (m, 1H), 8.20 (d, J = 7.7 Hz, 1H), 7.82 (m, 1H), 7.37 (m, 1H), 7.30–7.21 (m, 5H), 7.16 (m, 1H), 3.68 (dd, J = 13.1, 8.6 Hz, 1H), 3.16 (m, 1H), 3.08–2.96 (m, 2H), 2.53 (dd, J = 7.0, 3.8 Hz, 2H), 1.80 (m, 2H), 1.76–1.51 (m, 5H), 1.47–1.30 (m, 4H); ^{13}C NMR (101 MHz, $CDCl_3$) δ 164.63, 150.61, 147.99, 142.87, 137.13, 129.18, 128.28, 125.71, 125.63, 122.22, 71.67, 51.86, 46.71, 36.30, 31.73, 30.88, 27.00, 26.69, 24.00. HRMS (ESI) m/z calculated for $C_{25}H_{33}N_3O$ $[M+H]^+$ 392.2702, found: 392.2710.

N-(3-methyl-4-phenyl-3-(piperidin-1-yl)butyl)picolinamide **(4e)**

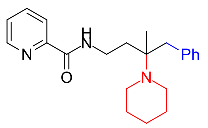

The title compound was isolated as a yellow oil (73% yield) after chromatography on alumina with ethyl acetate/hexane (1:3). ^{1}H NMR (400 MHz, $CDCl_3$) δ 9.00 (s, 1H), 8.68–8.42 (m, 2H), 8.18 (d, J = 7.8 Hz, 2H), 7.81 (m, 2H), 7.38 (dd, J = 7.6, 4.8 Hz, 2H), 7.25 (dd, J = 7.7, 6.1 Hz, 5H), 7.21–7.12 (m, 7H), 3.69–3.50 (m,

2H), 3.47–3.35 (m, 2H), 2.83–2.62 (m, 14H), 2.05 (m, 2H), 1.71 (q, J = 5.0 Hz, 9H), 1.48 (m, 7H), 1.08 (s, 7H); ^{13}C NMR (101 MHz, $CDCl_3$) δ 164.14, 150.04, 147.97, 144.22, 137.31, 128.51, 128.06, 126.00, 125.87, 122.17, 51.99, 43.27, 39.52, 31.40, 31.06, 29.90, 28.06, 26.57.HRMS (ESI) m/z calculated for $C_{22}H_{29}N_3O$ $[M+H]^+$ 352.2389, found: 352.2384.

N-(4-phenyl-3-(piperidin-1-yl)nonyl)picolinamide **(4f)**

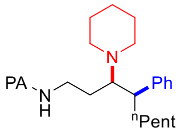

The title compound was isolated as a colorless oil (70% yield) after chromatography on alumina with ethyl acetate/hexane (1:3). This product was isolated as a >20:1 mixture of diastereomers. The reported dr was determined by 1H NMR analysis. ^{1}H NMR (400 MHz, $CDCl_3$) δ 8.50 (d, J = 4.9 Hz, 1H), 8.14 (d, J = 7.7 Hz, 2H), 7.80 (m, 1H), 7.38 (dd, J = 7.7, 4.7 Hz, 1H), 7.29–7.21 (m, 3H), 7.15 (dd, J = 14.7, 7.3 Hz, 3H), 3.21 (m, 2H), 2.68 (q, J = 4.7 Hz, 5H), 2.47 (s, 0H), 2.05–1.90 (m, 1H), 1.67 (m, 1H), 1.59 (m, 3H), 1.48 (q, J = 5.6 Hz, 3H), 1.39–1.28 (m, 1H), 1.30–1.09 (m, 3H), 0.99 (t, J = 7.4 Hz, 2H), 0.80 (t, J = 6.7 Hz, 3H); ^{13}C NMR (101 MHz, $CDCl_3$) δ 164.09, 150.26, 147.88, 144.29, 137.19, 128.67, 128.33, 126.07, 125.86, 122.13, 68.27, 50.26, 49.63, 39.04, 34.20, 31.92, 28.92, 27.15, 26.78, 25.19, 22.54, 14.04. HRMS (ESI) m/z calculated for $C_{26}H_{37}N_3O$ $[M+H]^+$ 408.3015, found: 408.3023.

N-(4-phenyl-3-(piperidin-1-yl)nonyl)picolinamide **(4g)**

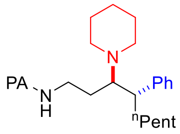

The title compound was isolated as a colorless oil (72% yield) after chromatography on alumina with ethyl acetate/hexane (1:3). This product was isolated as a >20:1 mixture of diastereomers. The reported dr was determined by 1H NMR analysis. ^{1}H NMR (400 MHz, $CDCl_3$) δ 8.79 (br, 1H), 8.53–8.50 (m, 1H), 8.19 (d, J = 7.8 Hz, 1H), 7.82 (m, 1H), 7.41–7.37 (m, 1H), 7.25 (m, 2H), 7.17 (d, J = 7.2 Hz, 3H), 3.66 (dd, J = 13.4, 6.6 Hz, 1H), 3.43 (dd, J = 11.9, 6.8 Hz, 1H), 2.83 (dd, J = 10.2, 4.8 Hz, 1H), 2.79–2.70 (m, 1H), 2.57 (m, 2H), 2.47–2.36 (m, 2H), 1.84 (m, 2H), 1.63 (d, J = 8.9 Hz, 2H), 1.54–1.49 (m, 3H), 1.41–1.36 (m, 2H), 1.29–1.25 (m, 2H), 1.19 (dd, J = 7.2, 2.7 Hz, 3H), 1.05 (d, J = 6.9 Hz, 2H), 0.82–0.78 (m, 3H); ^{13}C NMR (101 MHz, $CDCl_3$) δ 164.38, 150.35, 147.91, 137.29, 137.18, 128.30, 128.17, 126.02, 125.88, 122.16, 69.93, 50.62, 45.88, 39.33, 32.02, 31.72, 27.36, 27.26, 26.36,

24.95, 22.55, 14.05. HRMS (ESI) m/z calculated for $C_{26}H_{37}N_3O$ $[M+H]^+$ 408.3015, found: 408.3019.

***N-(4-phenyl-3-(piperidin-1-yl)hexyl)picolinamide* (4h)**

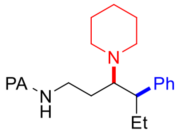

The title compound was isolated as a colorless oil (71% yield) after chromatography on alumina with ethyl acetate/hexane (1:3). This product was isolated as a >20:1 mixture of diastereomers. The reported dr was determined by ^{1}H NMR analysis. ^{1}H NMR (400 MHz, $CDCl_3$) δ 8.43 (d, J = 4.2 Hz, 1H), 8.15 (br, 1H), 8.06 (d, J = 7.8 Hz, 1H), 7.72 (m, 1H), 7.30 (m, 1H), 7.18 (t, J = 7.4 Hz, 2H), 7.10 (d, J = 7.3 Hz, 1H), 7.07–7.01 (m, 2H), 3.13 (m, 2H), 2.75–2.43 (m, 6H), 1.63 (m, 1H), 1.52 (m, 4H), 1.41 (m, 3H), 1.28 (m, 1H), 1.24–1.15 (m, 1H), 0.56 (t, J = 7.4 Hz, 3H); ^{13}C NMR (101 MHz, $CDCl_3$) δ 164.06, 150.24, 147.88, 147.85, 143.97, 137.20, 128.70, 128.34, 126.09, 125.87, 122.12, 68.16, 51.67, 50.22, 39.08, 29.01, 27.12, 26.81, 25.22, 12.15, 12.12. HRMS (ESI) m/z calculated for $C_{23}H_{31}N_3O[M+H]^+$ 366.2545, found: 366.2555.

***N-(4-(4-acetylphenyl)-3-(piperidin-1-yl)hexyl)picolinamide* (4i)**

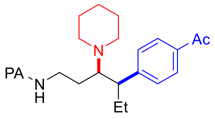

The title compound was isolated as a white solid (51% yield) after chromatography on alumina with ethyl acetate/hexane (1:3). This product was isolated as a 15:1 mixture of diastereomers. The reported dr was determined by GC-MS analysis. ^{1}H NMR (400 MHz, $CDCl_3$) δ 8.50 (d, J = 4.2 Hz, 1H), 8.14 (d, J = 7.8 Hz, 1H), 8.12–8.09 (m, 1H), 7.85 (d, J = 8.2 Hz, 2H), 7.80 (dd, J = 7.7, 1.5 Hz, 1H), 7.39 (dd, J = 7.0, 5.2 Hz, 1H), 7.23 (d, J = 8.2 Hz, 2H), 3.28 (dd, J = 18.1, 6.9 Hz, 2H), 2.86–2.59 (m, 6H), 2.57 (s, 3H), 2.11–2.02 (m, 1H), 1.69 (dd, J = 14.8, 7.1 Hz, 1H), 1.58 (d, J = 4.9 Hz, 4H), 1.54–1.50 (m, 1H), 1.48 (d, J = 5.5 Hz, 2H), 1.29 (m, 2H), 0.63 (t, J = 7.4 Hz, 3H); ^{13}C NMR (101 MHz, $CDCl_3$) δ 197.96, 164.21, 150.29, 150.23, 148.04, 137.39, 135.44, 129.04, 128.68, 126.09, 122.25, 68.01, 51.95, 50.45, 39.02, 29.37, 27.10, 26.96, 26.65, 25.29, 12.21. HRMS (ESI) m/z calculated for $C_{25}H_{33}N_3O_2$ $[M+H]^+$ 408.2651, found: 408.2650.

***N-(4,4-diphenyl-3-(piperidin-1-yl)butyl)picolinamide* (4j)**

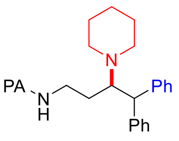

The title compound was isolated as a colorless oil (72% yield) after chromatography on alumina with ethyl acetate/hexane (1:3). ^{1}H NMR (400 MHz, $CDCl_3$) δ 8.40 (m, 1H), 8.37 (s, 0H), 8.07 (d, J = 7.9 Hz, 1H), 7.70 (m, 1H), 7.27 (dd, J = 7.8, 2.8 Hz, 3H), 7.19 (d, J = 7.3 Hz, 2H), 7.12 (m, 4H), 7.02 (q, J = 7.4 Hz, 2H), 3.92 (d, J = 10.5 Hz, 1H), 3.44 (m, 1H), 3.39–3.31 (m, 2H), 2.47 (m, 2H), 2.34 (m, 2H), 1.61 (m, 1H), 1.44 (m, 1H), 1.30 (m, 3H), 1.17 (q, J = 5.8 Hz, 2H).; ^{13}C NMR (101 MHz, $CDCl_3$) δ 164.27, 150.28, 147.91, 137.23, 128.77, 128.60, 128.13, 126.14, 125.92, 122.16, 67.08, 55.70, 50.41, 39.01, 30.03, 26.69, 25.03. HRMS (ESI) m/z calculated for $C_{27}H_{31}N_3O$ $[M+H]^+$ 414.2545, found: 414.2540.

***N-(4,5-diphenyl-3-(piperidin-1-yl)pentyl)picolinamide* (4k)**

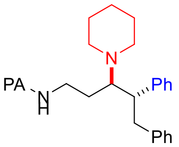

The title compound was isolated as a colorless oil (74% yield) after chromatography on alumina with ethyl acetate/hexane (1:3). This product was isolated as a >20:1 mixture of diastereomers. The reported dr was determined by 1H NMR analysis. ^{1}H NMR (400 MHz, $CDCl_3$) δ 8.50 (d, J = 4.4 Hz, 1H), 8.16 (d, J = 7.8 Hz, 1H), 8.05 (s, 1H), 7.81 (m, 1H), 7.39 (m, 1H), 7.22–7.01 (m, 9H), 6.87 (d, J = 6.8 Hz, 2H), 3.50 (dd, J = 13.3, 3.2 Hz, 1H), 3.29 (dd, J = 13.3, 7.1 Hz, 1H), 3.15 (dd, J = 13.6, 7.1 Hz, 1H), 2.93 (d, J = 7.0 Hz, 1H), 2.89–2.63 (m, 7H), 1.79 (dd, J = 14.2, 6.7 Hz, 1H), 1.69–1.59 (m, 6H), 1.51 (d, J = 5.6 Hz, 2H), 1.46–1.37 (m, 1H); ^{13}C NMR (101 MHz, $CDCl_3$) δ 164.17, 150.25, 148.00, 143.24, 141.38, 137.35, 129.14, 129.02, 128.33, 127.93, 126.33, 126.04, 125.52, 122.23, 67.13, 52.17, 50.31, 41.02, 39.04, 28.69, 26.99, 25.31. HRMS (ESI) m/z calculated for $C_{28}H_{33}N_3O$ $[M+H]^+$ 428.2702, found: 428.2712.

***N-(5-methyl-4-phenyl-3-(piperidin-1-yl)hexyl)picolinamide* (4l)**

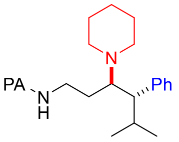

The title compound was isolated as a colorless oil (72% yield) after chromatography on alumina with ethyl acetate/hexane (1:3). This product was isolated as a 10:1 mixture of diastereomers. The reported dr was determined by ^{1}H NMR analysis. The following analytical data correspond to the mixture. ^{1}H NMR (400 MHz, $CDCl_3$) δ 8.53 (d, *J* = 4.7 Hz, 0.09H), 8.50 (d, *J* = 4.6 Hz, 0.91H), 8.21 (d, *J* = 7.8 Hz, 0.09H), 8.16 (d, *J* = 7.8 Hz, 0.91H), 8.13 (br, 0.74H), 7.85 (dd, *J* = 7.8, 1.7 Hz, 0.09H), 7.81 (m, 0.91H), 7.43–7.40 (m, 0.09H), 7.38 (m, 0.91H), 7.25–7.16 (m, 3H), 7.10 (d, *J* = 6.9 Hz, 2H), 3.48–3.22 (m, 2H),

3.03 (m, 1H), 2.67 (t, *J* = 4.8 Hz, 5H), 2.27 (dd, *J* = 11.9, 6.7 Hz, 1H), 1.74 (dd, *J* = 14.5, 6.3 Hz, 1H), 1.66–1.49 (m, 4H), 1.45 (t, *J* = 14.2 Hz, 2H), 1.40–1.33 (m, 1H), 1.25 (s, 1H), 0.77 (d, *J* = 6.8 Hz, 3H), 0.72 (d, *J* = 7.0 Hz, 3H); ^{13}C NMR (101 MHz, $CDCl_3$) δ 164.19, 150.34, 148.01, 141.17, 137.33, 130.18, 127.87, 126.18, 126.02, 122.24, 63.94, 54.89, 50.36, 39.31, 29.26, 28.70, 27.06, 25.36, 21.99, 17.58. HRMS (ESI) m/z calculated for $C_{24}H_{33}N_3O$ $[M+H]^+$ 380.2702, found: 380.2720.

N-(3-phenyl-2-(piperidin-1-yl)propyl)picolinamide **(4m)**

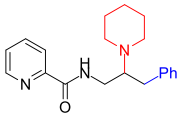

The title compound was isolated as a colorless oil (56% yield) after chromatography on alumina with ethyl acetate/hexane (1:3). ^{1}H NMR (400 MHz, $CDCl_3$) δ 8.54–8.49 (m, 1H), 8.20–8.16 (m, 1H), 8.01 (br, 1H), 7.83 (m, 1H), 7.40 (m, 1H), 7.27–7.21 (m, 2H), 7.20–7.07 (m, 3H), 3.37 (d, *J* = 6.9 Hz, 1H), 3.07–2.92 (m, 1H), 2.66 (s, 2H), 2.45 (s, 1H), 2.34–2.22 (m, 1H), 1.79–1.65 (m, 2H), 1.54 (dd, *J* = 13.0, 6.6 Hz, 5H), 1.48–1.36 (m, 2H); ^{13}C NMR (101 MHz, $CDCl_3$) δ 164.19, 150.34, 148.01, 141.17, 137.33, 130.18, 127.87, 126.18, 126.02, 122.24, 63.94, 54.89, 50.36, 39.31, 29.26, 28.70, 27.06, 25.36, 21.99, 17.58. HRMS (ESI) m/z calculated for $C_{20}H_{25}N_3O$ $[M+H]^+$ 324.2076, found: 324.2080.

N-(5-phenyl-4-(piperidin-1-yl)pentyl)picolinamide **(4n)**

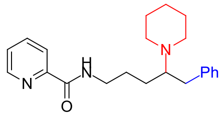

The title compound was isolated as a colorless oil (81% yield) after chromatography on alumina with ethyl acetate/hexane (1:3). ^{1}H NMR (400 MHz, $CDCl_3$) δ 8.51 (d, J = 4.8 Hz, 1H), 8.17 (d, J = 7.8 Hz, 1H), 8.01 (s, 1H), 7.82 (t, J = 7.7 Hz, 1H), 7.39 (dd, J = 7.6, 4.8 Hz, 1H), 7.22 (t, J = 7.5 Hz, 2H), 7.13 (q, J = 3.8, 3.3 Hz, 3H), 3.37 (p, J = 6.5 Hz, 2H), 3.00 (dd, J = 13.1, 4.1 Hz, 1H), 2.66 (m, 3H), 2.55–2.40 (m, 2H), 2.30 (dd, J = 13.1, 9.3 Hz, 1H), 1.69 (t, J = 8.3 Hz, 1H), 1.55 (m, 5H), 1.41 (m, 3H); ^{13}C NMR (101 MHz, $CDCl_3$) δ 164.18, 150.15, 147.93, 141.15, 137.26, 129.18, 128.24, 125.95, 125.63, 122.15, 66.52, 49.58, 39.45, 35.24, 27.85, 26.85, 26.53, 25.05. HRMS (ESI) m/z calculated for $C_{22}H_{29}N_3O$ $[M+H]^+$ 352.2389, found: 352.2390.

N-(4-(tert-butylamino)-5-phenylpentyl)picolinamide **(4o)**

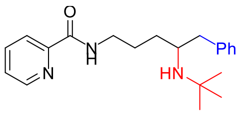

The title compound was isolated as a yellow oil (71% yield) after chromatography on alumina with ethyl acetate/hexane (1:3). ^{1}H NMR (400 MHz, $CDCl_3$) δ 9.19 (s, 1H), 8.45 (m, 1H), 8.11 (m, 1H), 7.74 (m, 1H), 7.35–7.28 (m, 1H), 7.25–7.18 (m, 2H), 7.13 (m, 3H), 3.60–3.41 (m, 2H), 3.00 (m, 1H), 2.73 (dd, J = 13.3, 6.2 Hz, 1H), 2.61 (dd, J = 13.3, 7.3 Hz, 1H), 1.73 (m, 1H), 1.59–1.41 (m, 1H), 1.18 (s, 1H), 1.00 (s, 9H); ^{13}C NMR (101 MHz, $CDCl_3$) δ 164.16, 150.50, 148.00, 139.36, 137.14, 129.41, 128.44, 126.32, 125.79, 122.16, 52.89, 51.18, 43.75, 37.35, 37.30, 34.91, 29.87, 29.82. HRMS (ESI) m/z calculated for $C_{21}H_{29}N_3O$ $[M+H]^+$ 340.4824, found: 340.2394.

N-(2,2-dimethyl-5-phenyl-4-(piperidin-1-yl)pentyl)picolinamide **(4p)**

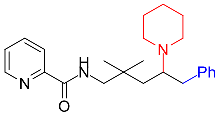

The title compound was isolated as a colorless oil (45% yield) after chromatography on alumina with ethyl acetate/hexane (1:3). ^{1}H NMR (400 MHz, $CDCl_3$) δ 8.69 (br, 1H), 8.54 (d, *J* = 4.1 Hz, 1H), 8.17 (s, 1H), 8.11 (s, 1H), 7.84 (m, 1H), 7.45–7.41 (m, 2H), 7.22 (s, 1H), 7.17 (d, *J* = 7.3 Hz, 1H), 7.09 (d, *J* = 7.1 Hz, 1H), 3.21 (s, 1H), 3.06 (dd, *J* = 13.6, 6.1 Hz, 1H), 2.98 (dd, *J* = 12.9, 3.4 Hz, 1H), 2.87–2.71 (m, 3H), 2.58 (s, 2H), 2.29–2.23 (m, 1H), 1.82–1.66 (m, 4H), 1.57–1.43 (m, 3H), 1.10 (d, *J* = 14.9 Hz, 1H), 0.88 (m, 1H), 0.78 (s, 3H), 0.35 (s, 3H); ^{13}C NMR (101 MHz, $CDCl_3$) δ 164.53, 150.65, 147.71, 140.97, 137.03, 129.41, 128.23, 125.66, 125.64, 122.41, 64.44, 50.07, 47.47, 39.54, 34.74, 27.22, 27.18, 26.19, 25.16, 24.84. HRMS (ESI) m/z calculated for $C_{24}H_{33}N_3O$ $[M+H]^+$ 380.2702, found: 380.2710.

N-(5-phenyl-4-(piperidin-1-yl)hexyl)picolinamide **(4q)**

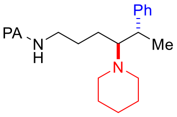

The title compound was isolated as a colorless oil (58% yield) after chromatography on alumina with ethyl acetate/hexane (1:3). This product was isolated as a >20:1 mixture of diastereomers. The reported dr was determined by ^{1}H NMR analysis. ^{1}H NMR (400 MHz, $CDCl_3$) δ 8.59 (d, J = 5.8 Hz, 1H), 8.55 (dd, J = 4.9, 1.6 Hz, 1H), 8.14 (d, J = 7.9 Hz, 1H), 7.80 (m, 1H), 7.44–7.35 (m, 1H), 7.31–7.23 (m, 3H), 7.20–7.12 (m, 2H), 3.68–3.44 (m, 1H), 3.20 (m, 1H), 3.06 (dd, J = 13.3, 4.2 Hz, 1H), 2.95 (m, 1H), 2.75 (m, 2H), 2.56–2.37 (m, 2H), 2.04 (s, 0H), 1.64 (m, 4H), 1.50 (q, J = 5.8 Hz, 2H), 1.37–1.17 (m, 1H), 0.90 (m, 0H); ^{13}C NMR (101 MHz, $CDCl_3$) δ 164.33, 150.30, 148.12, 147.26, 137.44, 128.04,

127.91, 126.12, 125.71, 122.32, 70.05, 50.87, 40.85, 39.72, 28.10, 27.03, 25.66, 25.21, 18.85. HRMS (ESI) m/z calculated for $C_{23}H_{31}N_3O$ $[M+H]^+$ 366.2545, found: 366.2545.

N-(5,5-diphenyl-4-(piperidin-1-yl)pentyl)picolinamide **(4r)**

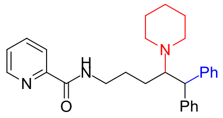

The title compound was isolated as a colorless oil (65% yield) after chromatography on alumina with ethyl acetate/hexane (1:3). ^1H NMR (400 MHz, $CDCl_3$) δ 8.53 (d, J = 4.9 Hz, 1H), 8.20 (d, J = 7.8 Hz, 1H), 7.92 (s, 0H), 7.84 (m, 1H), 7.41 (dd, J = 7.6, 4.8 Hz, 1H), 7.33 (d, J = 7.7 Hz, 2H), 7.27 (d, J = 7.0 Hz, 1H), 7.21 (t, J = 7.4 Hz, 2H), 7.18–7.11 (m, 3H), 7.05 (d, J = 7.3 Hz, 1H), 3.93 (d, J = 10.4 Hz, 1H), 3.38 (m, 3H), 2.55 (m, 2H), 2.39 (dd, J = 11.2, 5.2 Hz, 2H), 1.75–1.62 (m, 1H), 1.52 (m, 2H), 1.25 (d, J = 4.3 Hz, 7H); ^{13}C NMR (101 MHz, $CDCl_3$) δ 164.16, 150.11, 147.96, 137.30, 128.56, 128.15, 127.95, 125.99, 125.81, 122.17, 66.99, 50.25, 39.13, 29.72, 27.83, 27.53, 26.87, 25.01. HRMS (ESI) m/z calculated for $C_{28}H_{33}N_3O$ $[M+H]^+$ 428.2702, found: 428.2710.

4. Gram-scale Reaction and PA Removal

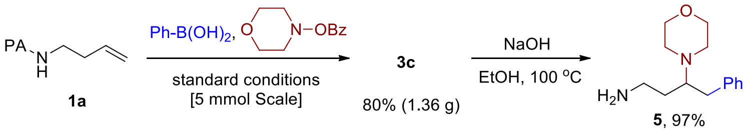

General Procedure for Removal of Picolinamide Directing Group(GP3):

Removal of picolinic acid directing group was carried out by adapting a literature procedure[6]. To an oven-dried schlenk flask was added the aryl amination product **3c** (0.2 mmol, 1.0 eq), NaOH (1 mmol, 5 eq), and EtOH (1 mL). The resulting mixture was stirred at 100 °C for 12 h. After this time, the reaction mixture was allowed to cool to room temperature, diluted by addition of EtOAc (5 mL) and H_2O (2 mL × 2). The aqueous layers were combined and extracted with EtOAc (10 mL × 2). The organic layers were combined, dried over Na_2SO_4, and concentrated in vacuo to give pure orimary amine product.

3-morpholino-4-phenylbutan-1-amine **(5)**

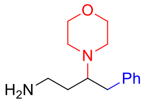

The title compound was isolated as a yellow oil (97% yield) after chromatography on alumina with ethyl acetate/hexane (1:1). ^{1}H NMR (400 MHz, $CDCl_3$) δ 7.19 (t, *J* = 7.4 Hz, 2H), 7.09 (dd, 3H), 3.68–3.54 (m, 4H), 2.93 (dd, *J* = 13.1, 4.0 Hz, 1H), 2.65 (m, 5H), 2.48–2.39 (m, 2H), 2.25 (dd, *J* = 13.0, 9.6 Hz, 1H), 1.92 (s, 2H), 1.53 (m, 1H), 1.34 (m, 1H); ^{13}C NMR (101 MHz, $CDCl_3$) δ 140.53, 129.19, 128.38, 125.88, 67.51, 65.44, 48.76, 40.31, 40.18, 35.02, 33.06. HRMS (ESI) m/z calculated for $C_{14}H_{22}N_2O$ $[M+H]^+$ 235.1810, found: 235.1820.

5. Further Transformation of Phamaceutically Relevant Compounds

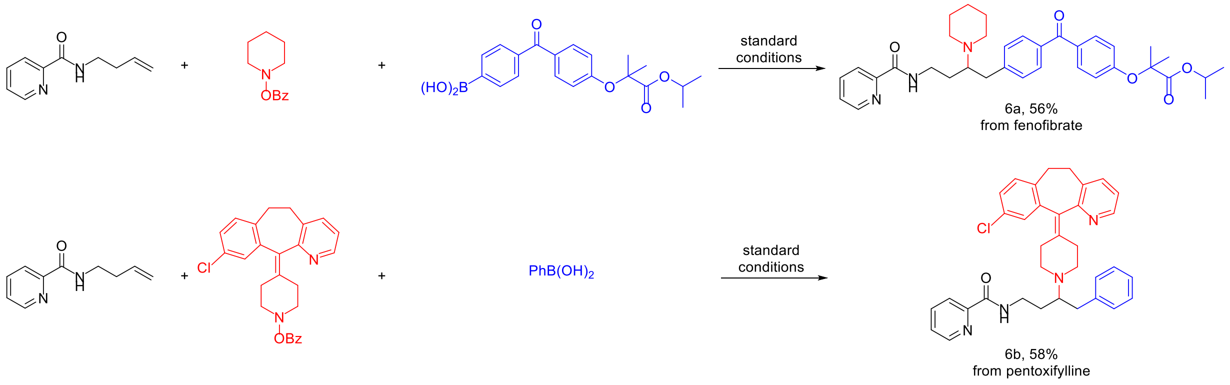

Isopropyl 2-methyl-2-(4-(4-(4-(picolinamido)-2-(piperidin-1-yl)butyl)benzoyl)phenoxy)propanoate (6a)

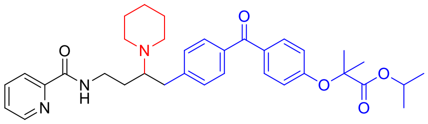

The title compound was isolated as a yellow oil (46% yield) after chromatography on alumina with ethyl acetate/hexane (1:3). ^{1}H NMR (400 MHz, $CDCl_3$) δ 8.54 (br, 1H), 8.20 (d, *J* = 7.4 Hz, 1H), 8.07 (s, 1H), 7.85 (t, *J* = 7.7 Hz, 1H), 7.77–7.66 (m, 5H), 7.42 (t, *J* = 7.2 Hz, 2H), 6.86 (d, *J* = 8.7 Hz, 2H), 5.11–5.06 (m, 1H), 3.72 (d, *J* = 6.9 Hz, 1H), 3.69–3.63 (m, 1H), 3.52 (d, *J* = 6.5 Hz, 1H), 2.75 (t, *J* = 7.3 Hz, 1H), 2.61 (d, *J* = 6.9 Hz, 1H), 1.75 (m, 4H), 1.66 (s, 6H), 1.26 (t, *J* = 6.6 Hz, 6H), 1.20 (d, *J* = 6.2 Hz, 8H); ^{13}C NMR (101 MHz, $CDCl_3$) δ 173.24, 164.44, 164.38, 159.52, 159.48, 150.12, 148.11, 147.95, 146.86, 137.43, 137.21, 135.93, 132.00, 131.05, 130.97, 130.21, 130.15, 129.14, 128.37, 126.16, 125.93, 122.27, 117.30, 79.46, 69.32, 39.24, 29.39, 28.51, 25.48, 21.63, 21.59. HRMS (ESI) m/z calculated for $C_{35}H_{43}N_3O_5$ $[M+H]^+$ 586.3281, found: 586.3288.

***N*-(3-(4-(9-chloro-5H-benzo[5,6]cyclohepta[1,2-b]pyridin-11(6H)-ylidene)piperidin-1-yl)-4-phenylbutyl)picolinamide** (6b)

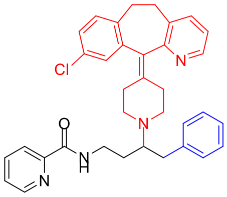

The title compound was isolated as a brown oil (58% yield) after chromatography on alumina with ethyl acetate/hexane (1:3). ^{1}H NMR (400 MHz, $CDCl_3$) δ 9.08 (m, 1H), 8.47 (t, J = 5.1 Hz, 1H), 8.33 (d, J = 4.9 Hz, 1H), 8.06 (m, 1H), 7.71 (dd, J = 8.7, 6.5 Hz, 1H), 7.36–7.33 (m, 1H), 7.30 (t, J = 6.2 Hz, 1H), 7.16 (t, J = 7.4 Hz, 2H), 7.09–6.99 (m, 7H), 3.58 (m, 1H), 3.41–3.28 (m, 2H), 3.28–3.14 (m, 2H), 2.93 (m, 2H), 2.76–2.70 (m, 2H), 2.68–2.63 (m, 2H), 2.56–2.50 (m, 1H), 2.34 (m, 2H), 2.20 (dd, J = 13.0, 10.0 Hz, 1H), 1.65 (m, 1H), 1.56–1.47 (m, 1H), 1.17 (d, J = 2.1 Hz, 1H), 1.15 (d, J = 7.2 Hz, 1H); ^{13}C NMR (101 MHz, $CDCl_3$) δ 171.05, 164.27, 157.89, 150.30, 147.92, 146.60, 140.32, 139.65, 139.44, 139.40, 137.81, 137.75, 137.12, 133.41, 133.37, 132.54, 132.39, 130.96, 129.11, 128.39, 125.88, 122.06, 67.13, 51.33, 39.14, 34.79, 31.92, 31.35, 31.08, 28.48. HRMS (ESI) m/z calculated for $C_{35}H_{35}ClN_4O$ $[M+H]^+$ 563.2578, found: 563.2585.

6. Radical Trapping Experiment

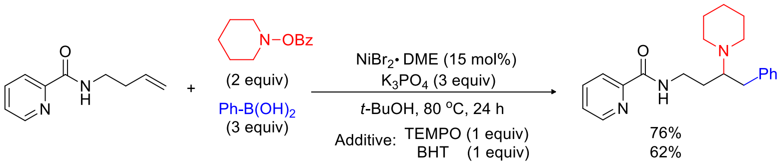

Procedure: To a 25 mL Schlenk tube were added $NiBr_2$•DME (0.03 mmol, 15 mol%), K_3PO_4 (0.6 mmol, 3 eq), alkene substrate (0.2 mmol, 1.0 eq), amine benzoate substrates (0.4 mmol, 2 eq), phenylboronic acid (0.6 mmol, 3 eq), Additive (0.2mmol, 1 equiv) and *t*-BuOH (2 mL). The resulting mixture was stired for 24 h at 80 °C. The products were separately obtained with a isolated yield of 76% and 62%. This result indicates that the reaction likely did not involve a radical process.

7. Radical Clock Experiment

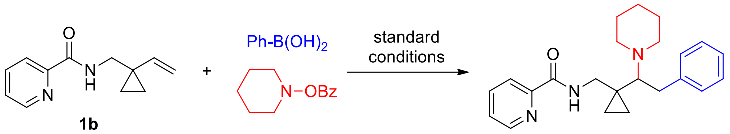

To a 25 mL Schlenk tube were added $NiBr_2$•DME (0.03 mmol, 15 mol%), K_3PO_4 (0.5 mmol, 2.5 eq), alkene substrate (0.2 mmol, 1.0 eq), phenylboronic acid (0.4 mmol, 2 eq), *t*-BuOH (2 mL). The resulting mixture was stired for 24 h at 80 °C. Finally, only cyclopropane remained product 4s was formed in 64% yield, implying that the cyclopropylmethyl radical intermediate known to ring rupture might not be generated in the catalytic cycle.

N-((1-(2-phenyl-1-(piperidin-1-yl)ethyl)cyclopropyl)methyl)picolinamide **(4s)**

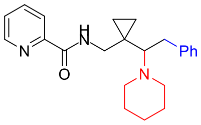

The title compound was isolated as a white oil (64% yield) after chromatography on alumina with ethyl acetate/hexane (1:3). ^{1}H NMR (400 MHz, $CDCl_3$) δ 9.52 (s, 1H), 8.56 (m, 1H), 8.19 (m, 1H), 7.83 (m, 1H), 7.40 (m, 1H), 7.26 (s, 2H), 7.21 – 7.14 (m, 3H), 3.66 (m, 1H), 3.32 (dd, J = 14.1, 6.6 Hz, 1H), 3.04 (dd, J = 14.4, 5.5 Hz, 1H), 2.68 – 2.55 (m, 6H), 1.69 – 1.65 (m, 4H), 1.46 – 1.39 (m, 2H), 0.61 (m, 1H), 0.55 – 0.49 (m, 1H), 0.43 – 0.32 (m, 2H); 13C NMR (101 MHz, CDCl3) δ 164.68, 150.67, 148.05, 141.81, 137.21, 128.98, 128.41, 125.95, 125.87, 122.39, 71.49, 52.33, 34.53, 26.21, 24.94, 21.63, 12.44, 8.76. HRMS (ESI) m/z calculated for C23H29N3O [M+H]+ 364.2389, found: 364.2400.

8. Control Experiment

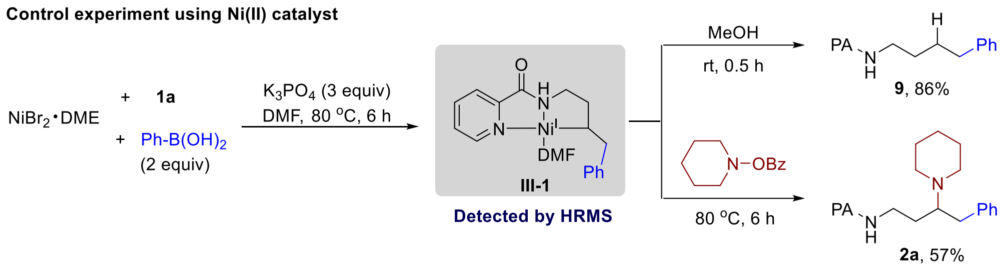

In a nitrogen-filled glovebox, DMF (2 mL) was added to a 25 mL schlenk tube that contained **1a** (35.2 mg, 0.2 mmol), phenylboronic acid (48.8 mg, 0.4mmol), K_3PO_4 (127.3 mg, 0.6 mmol) and $NiBr_2$·DME (61.7 mg, 0.2 mmol). The mixture was stirred for 6 h at 80 °C. After the reaction, the solution was blood red. The reaction solution was divided into two equal parts, one reacted with MeOH (0.040 ml, 1 mmol) for half an hour. After that, the hydroarylation product was isolated after chromatography on silica gel (86 % yield). The other part was subjected to piperidino benzoate (30.8 mg, 1.5 eq, 0.15 mmol) and reacted for 6 h.

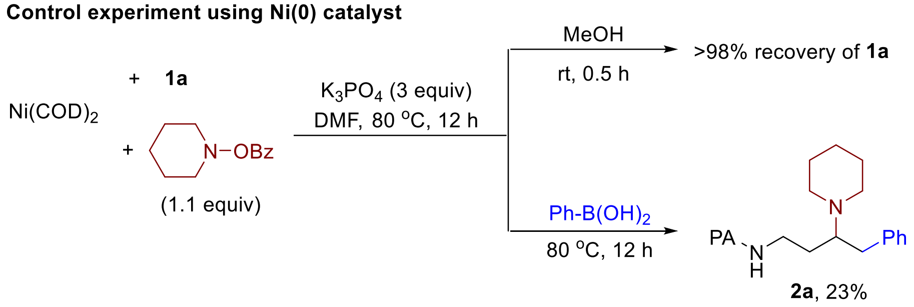

In a nitrogen-filled glovebox, DMF (2 mL) was added to a 25 mL Schlenk tube that contained **1a** (35.2 mg, 0.2 mmol), piperidino benzoate (45.2 mg, 0.22mmol)，K_3PO_4 (127.3 mg, 0.6 mmol) and $Ni(COD)_2$ (55 mg, 0.2 mmol). The mixture was stirred for 12 h at 80 °C. After that, the reaction solution was divided into two equal parts, one reacted with MeOH (0.040 ml, 1 mmol) for half an hour, the other part was subjected to phenylboronic acid (24.4 mg, 0.2 mmol) and reacted for 12 h.

9. HRMS(ESI) Analysis

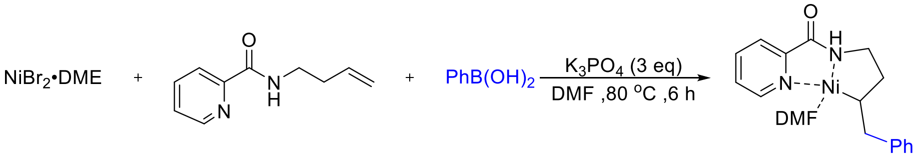

In a nitrogen-filled glovebox, DMF (3 mL) was added to a 25 mL Schlenk tube that contained N-(but-3-en-1-yl)picolinamide (52.9 mg, 0.3 mmol), phenylboronic acid (73.2 mg, 0.6mmol), K_3PO_4 (191 mg, 0.9 mmol) and $NiBr_2$·DME (92.6 mg, 0.3 mmol). The mixture was stirred for 6 h at 80 °C. After that, the blood red solution was analyzed by ESI-HRMS.

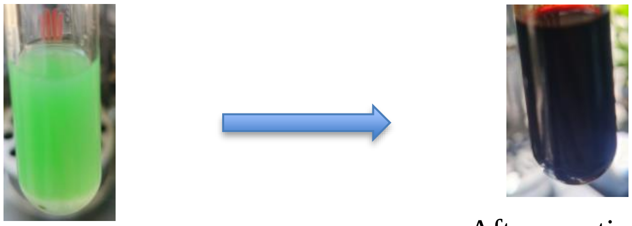

Before reaction After reaction

Supplementary Figure 1. Reaction process.

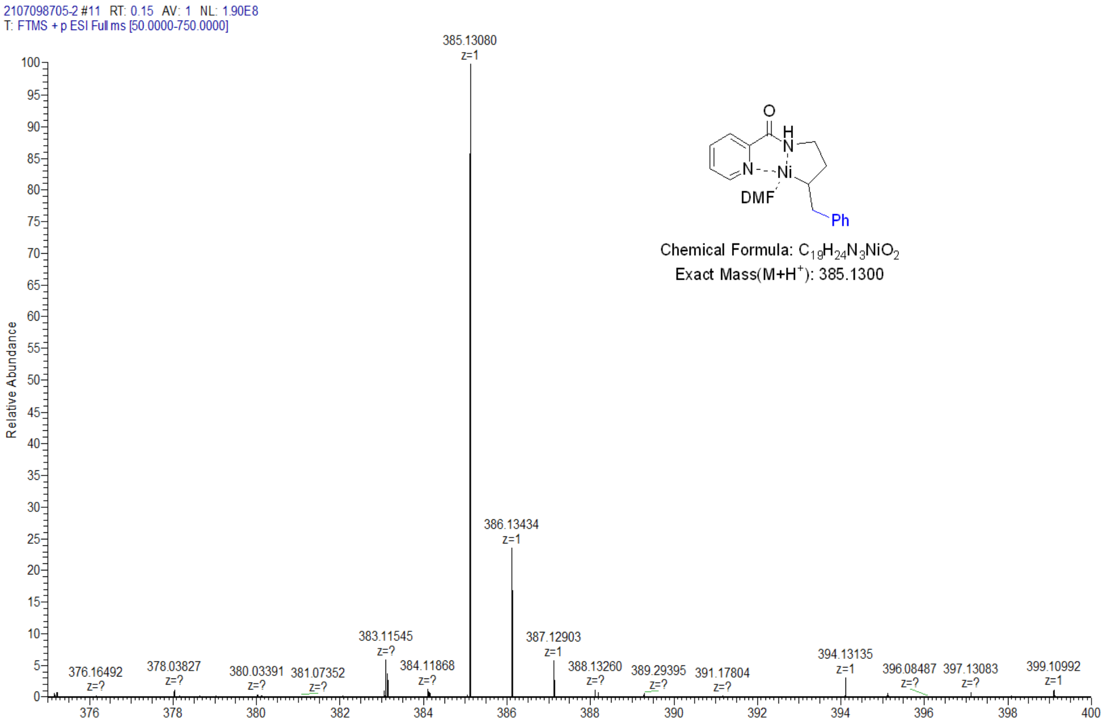

Supplementary Figure 2. HR-MS spectra of **III-1**

10. X-ray Crystallographic Data

Single crystals for X-ray studies were grown by slow evaporation of a solution of compound **4b** in a mixture of petroleum ether and ethyl acetate at room temperature. X-Ray structural analysis of single crystal **4b** was obtained to confirm the absolute configuration. The X-ray data of **4b** is deposited in the Cambridge Crystallographic Data Centre with a number of CCDC 2054628.

Crystal Data for $C_{27}H_{31}N_3O$ (M =413.55 g/mol): triclinic, space group P-1, a = 11.5615(3) Å, b = 12.3247(3) Å, c = 18.0802(4) Å, V = 2284.03(10) Å^3, Z = 4, T = 150.00(10) K, μ(MoKα) = 0.573 mm^{-1}, *Dcalc* = 1.203 g/cm^3, 34752 reflections measured (5.136° ≤ 2Θ ≤ 134.132°), 8156 unique (R_{int} = 0.0478, R_{sigma} = 0.0303) which were used in all calculations. The final R_1 was 0.0411 (I > 2σ (I)) and wR_2 was 0.1155 (all data).

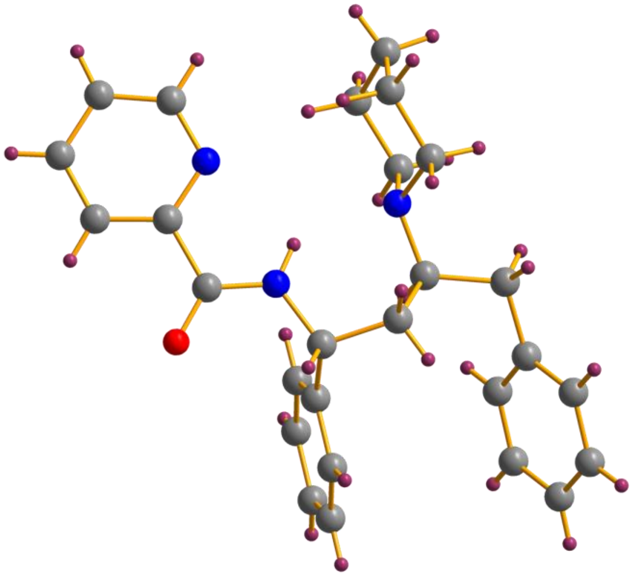

Supplementary Figure 3. X-ray structure of compound **4b** (CCDC 2054628).

Single crystals for X-ray studies were grown by slow evaporation of a solution of compound **4i** in a mixture of petroleum ether and ethyl acetate at room temperature. X-Ray structural analysis of single crystal **4i** was obtained to confirm the absolute configuration. The X-ray data of **4i** is deposited in the Cambridge Crystallographic Data Centre with a number of CCDC 2054629.

Crystal Data for $C_{25}H_{33}N_3O_2$ (M =407.54 g/mol): monoclinic, space group $P2_1/c$, a = 21.748(3) Å, b = 5.4836(6) Å, c = 19.4942(13) Å, V = 2291.3(4) $Å^3$, Z = 4, T = 293(2) K, μ(MoKα) = 0.593 mm^{-1}, *Dcalc* = 1.181 g/cm^3, 7506 reflections measured (8.25° ≤ 2Θ ≤ 134.122°), 4044 unique (R_{int} = 0.0533, R_{sigma} = 0.0617) which were used in all calculations. The final R_1 was 0.0787 (I > 2σ (I)) and wR_2 was 0.2353 (all data).

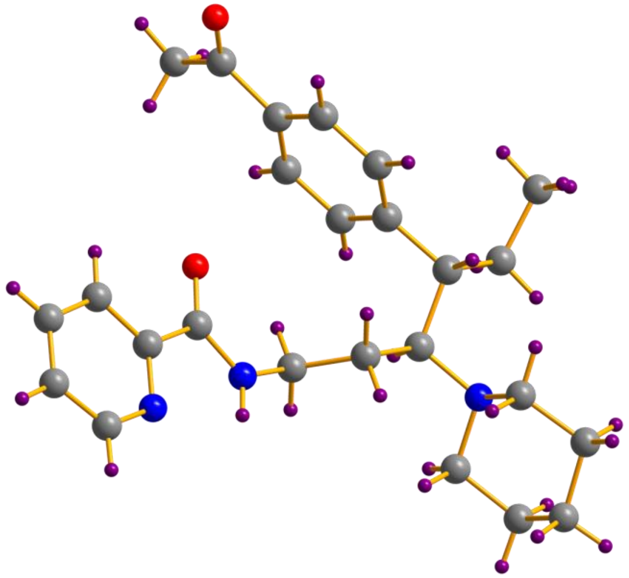

Supplementary Figure 4. X-ray structure of compound **4i** (CCDC 2054629).

11. Models for a-Substituted Terminal Alkenes

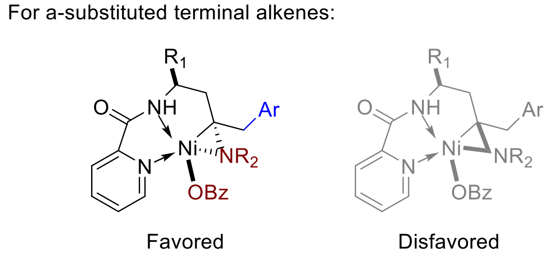

12. NMR Spectra

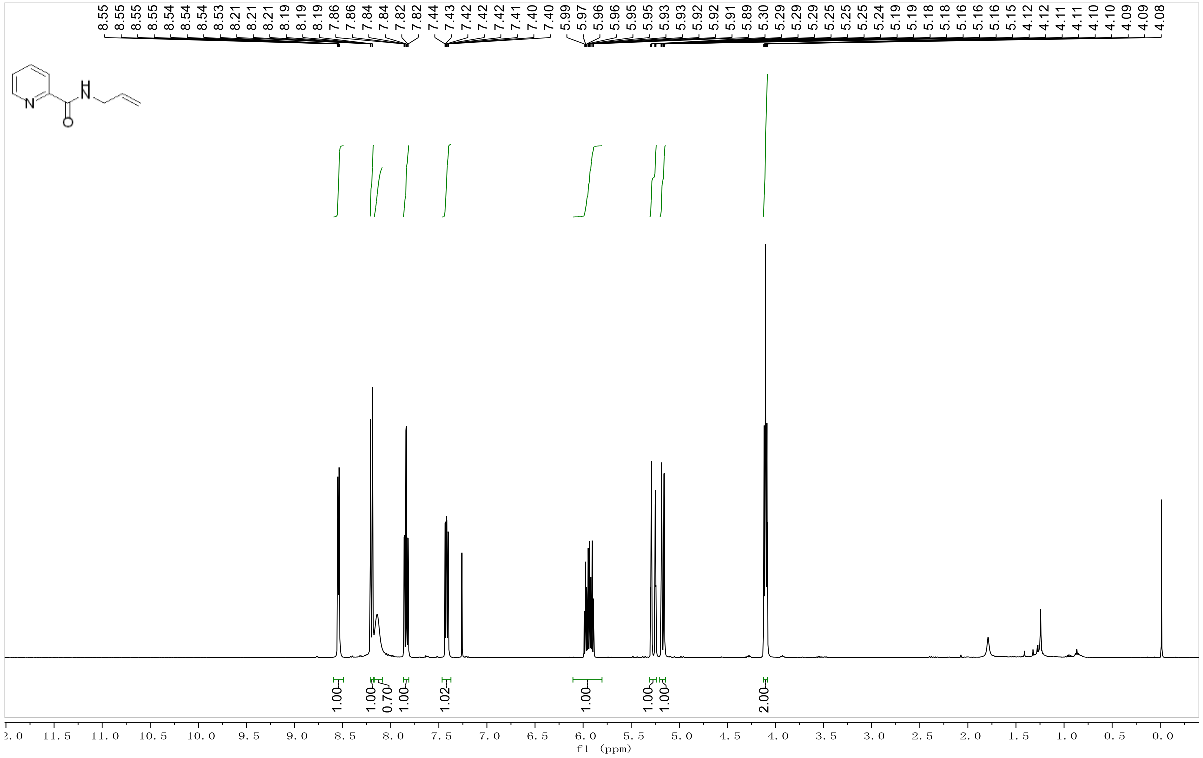

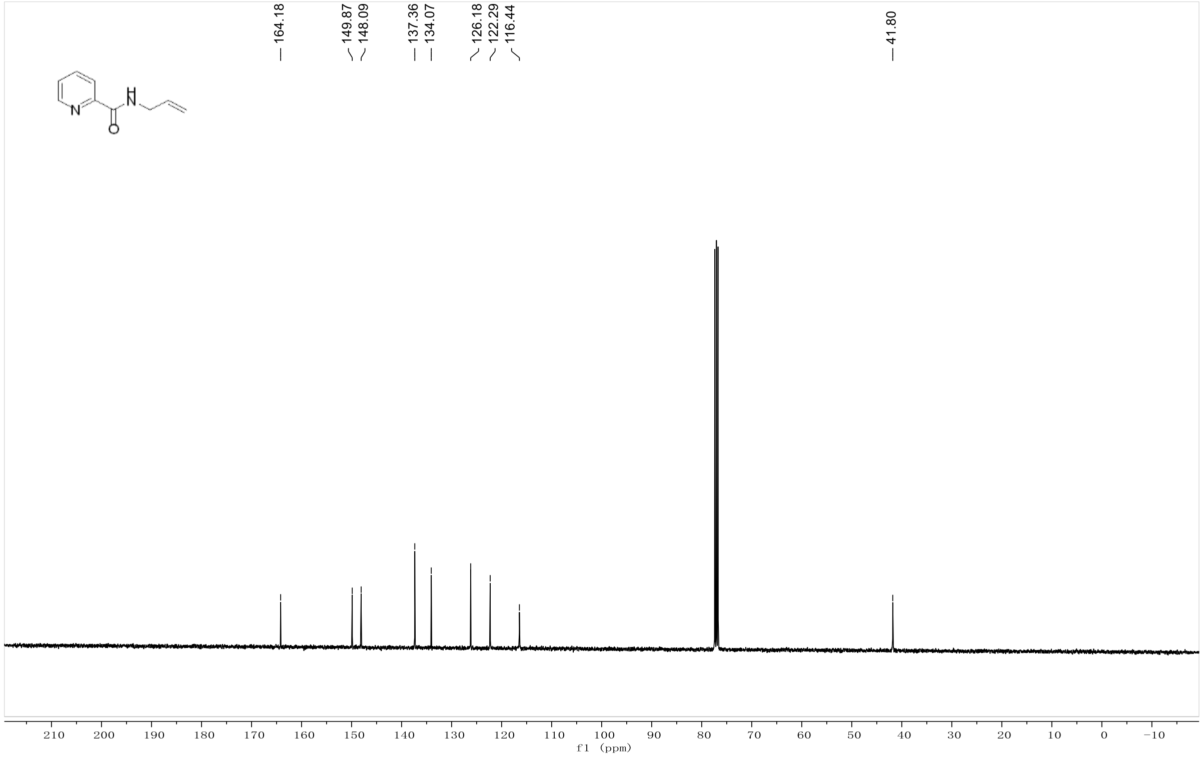

Supplementary Figure 5. ^{1}H NMR and ^{13}C NMR spectra of **1b**.

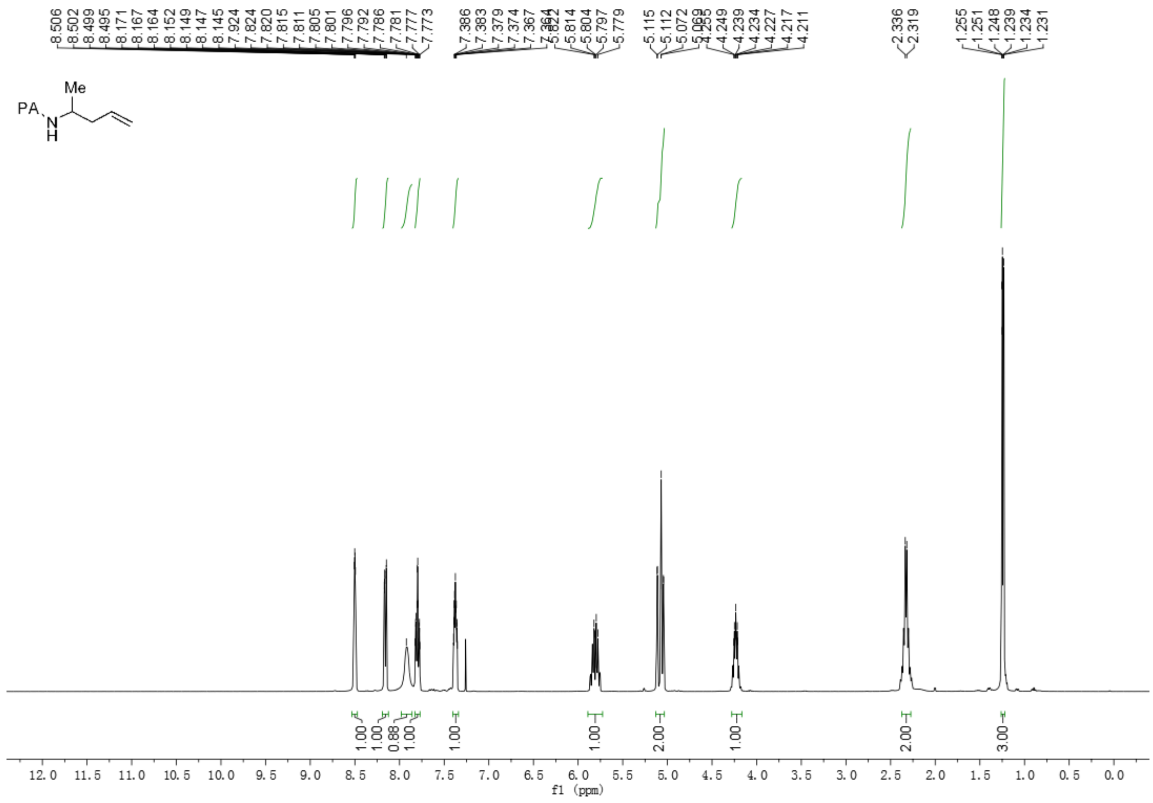

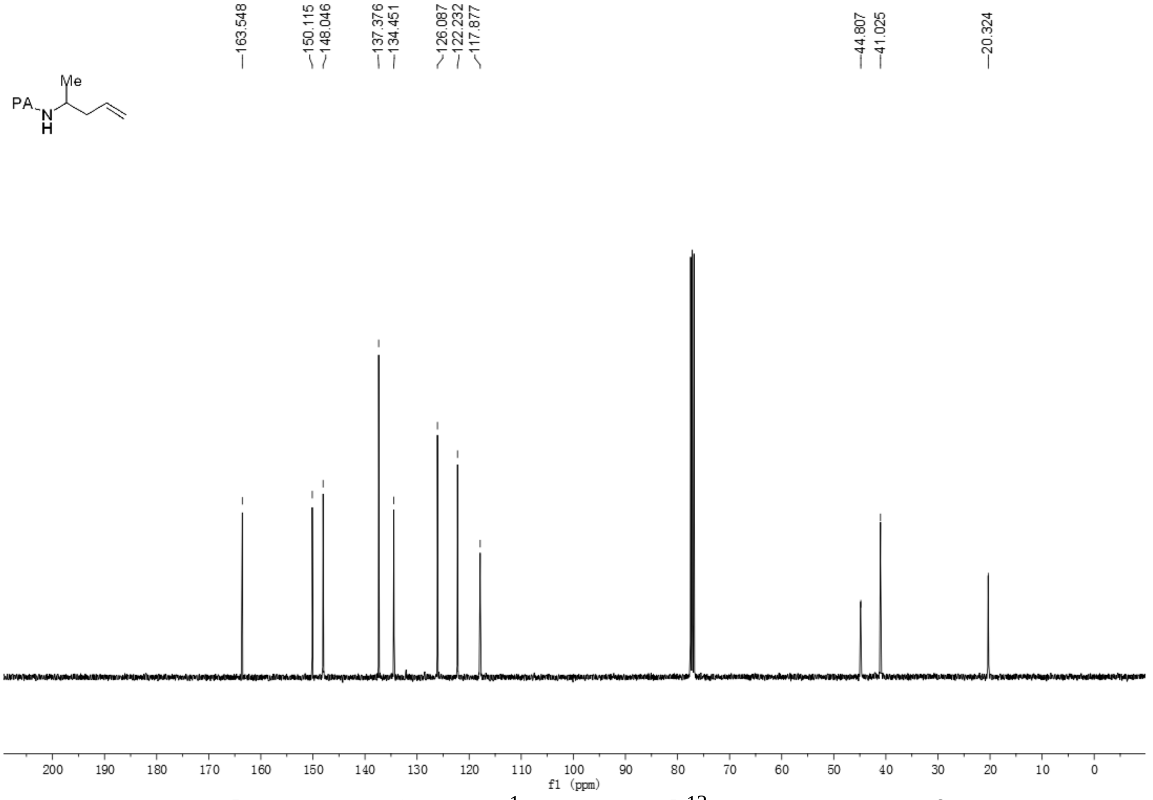

Supplementary Figure 6. ^{1}H NMR and ^{13}C NMR spectra of **1c**.

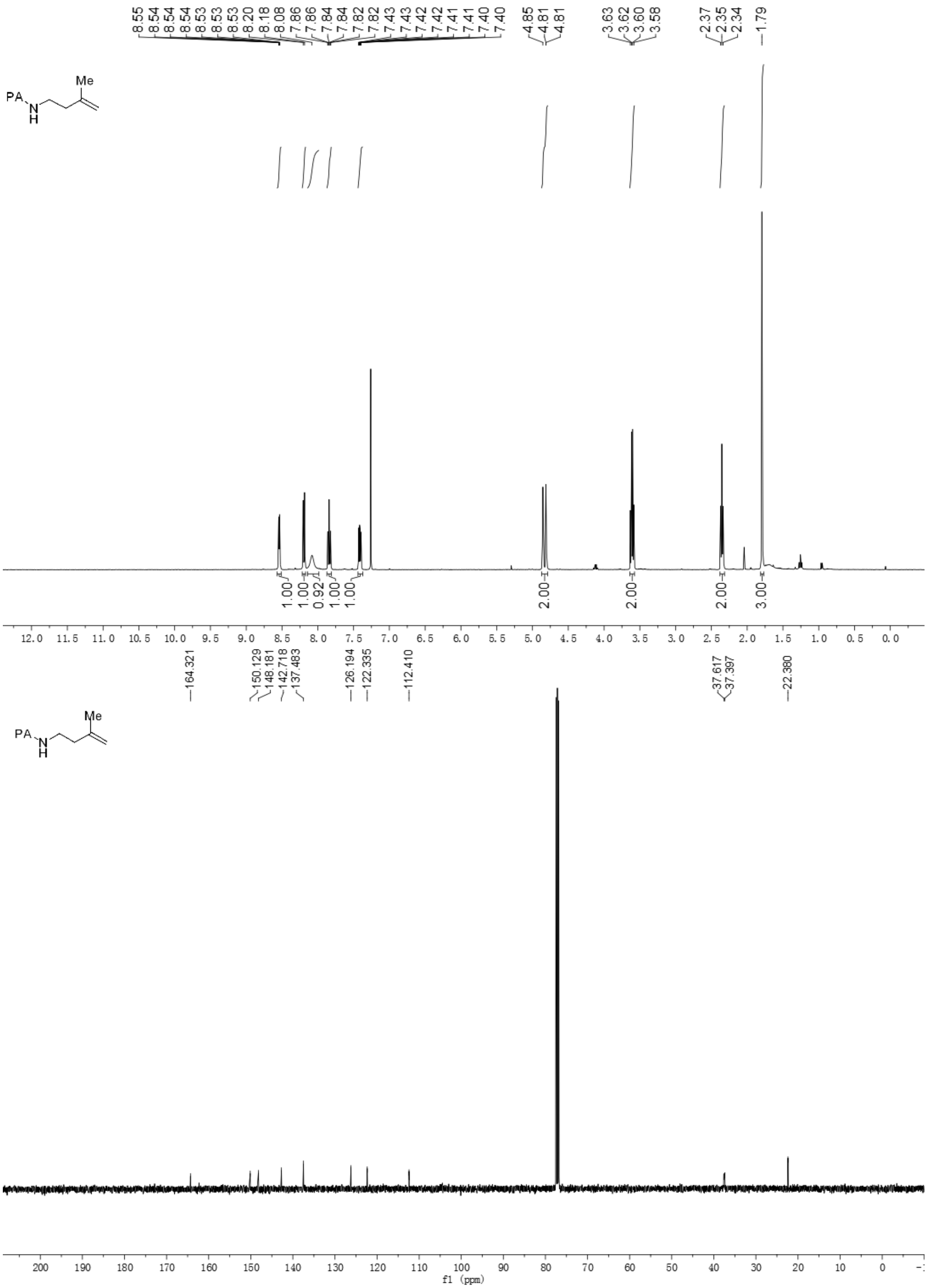

Supplementary Figure 7. ^{1}H NMR and ^{13}C NMR spectra of **1d**.

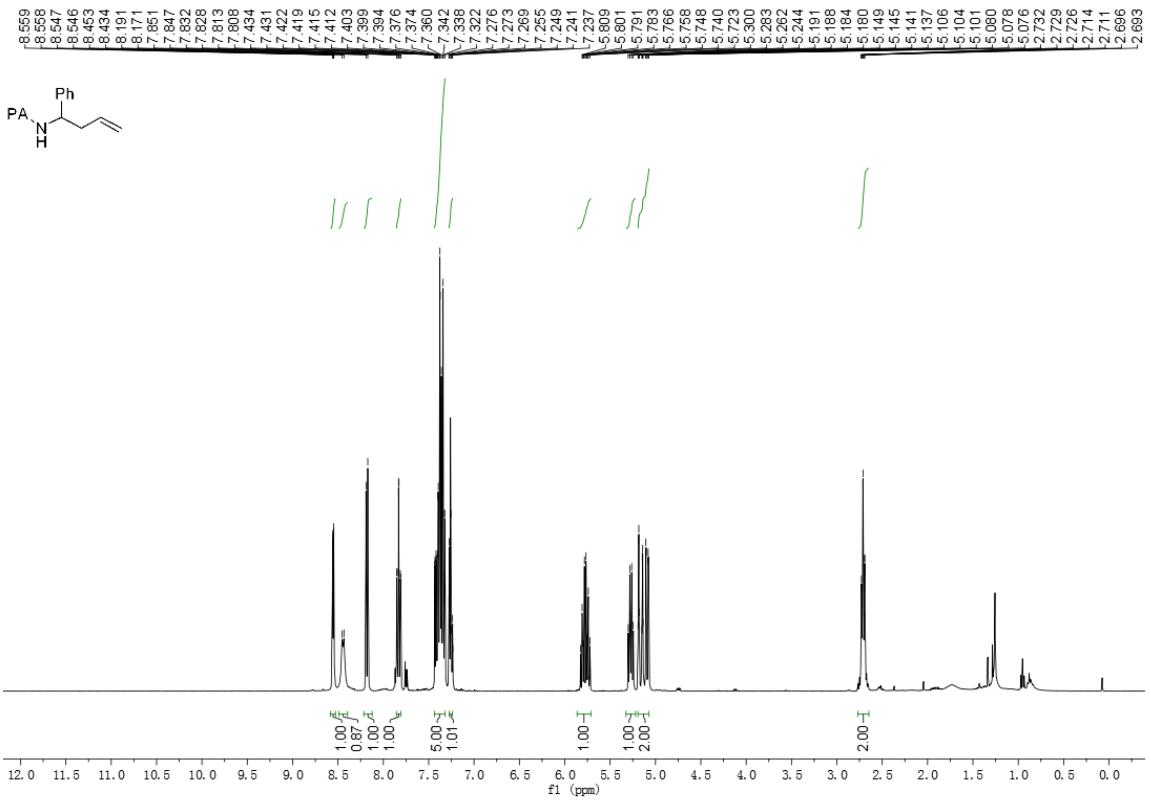

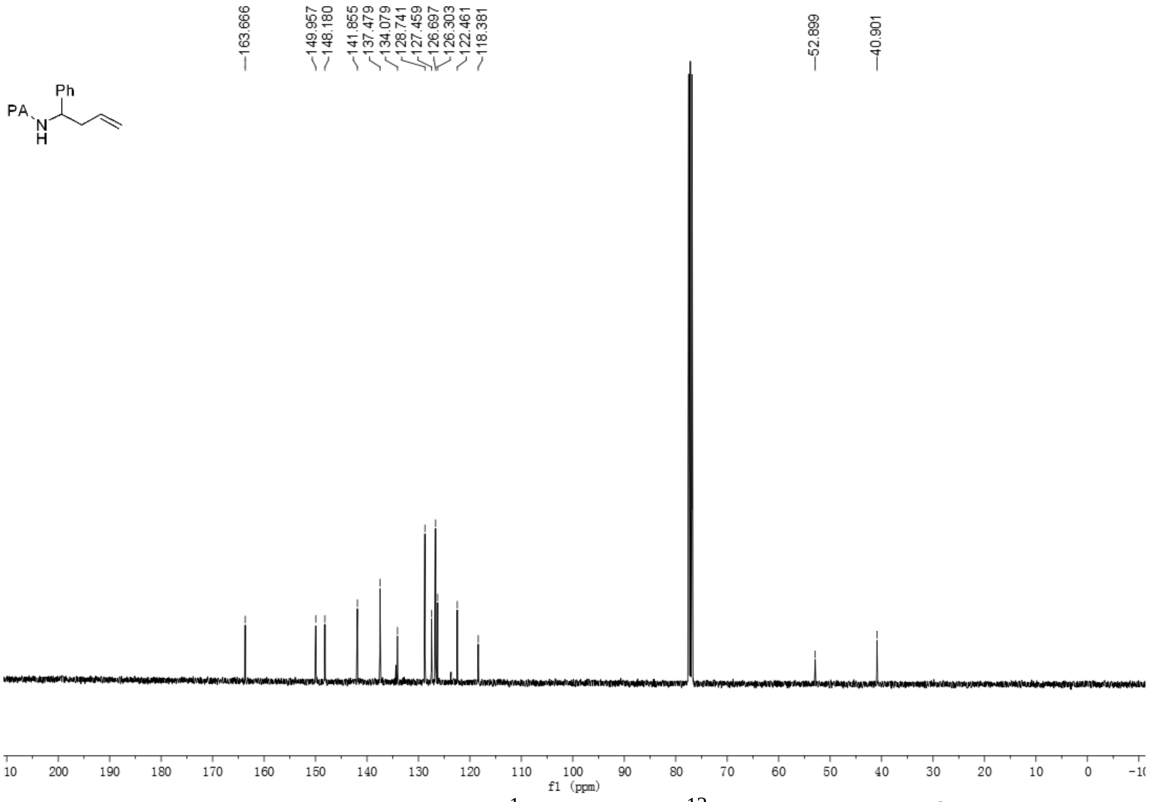

Supplementary Figure 8. ^{1}H NMR and ^{13}C NMR spectra of **1e**.

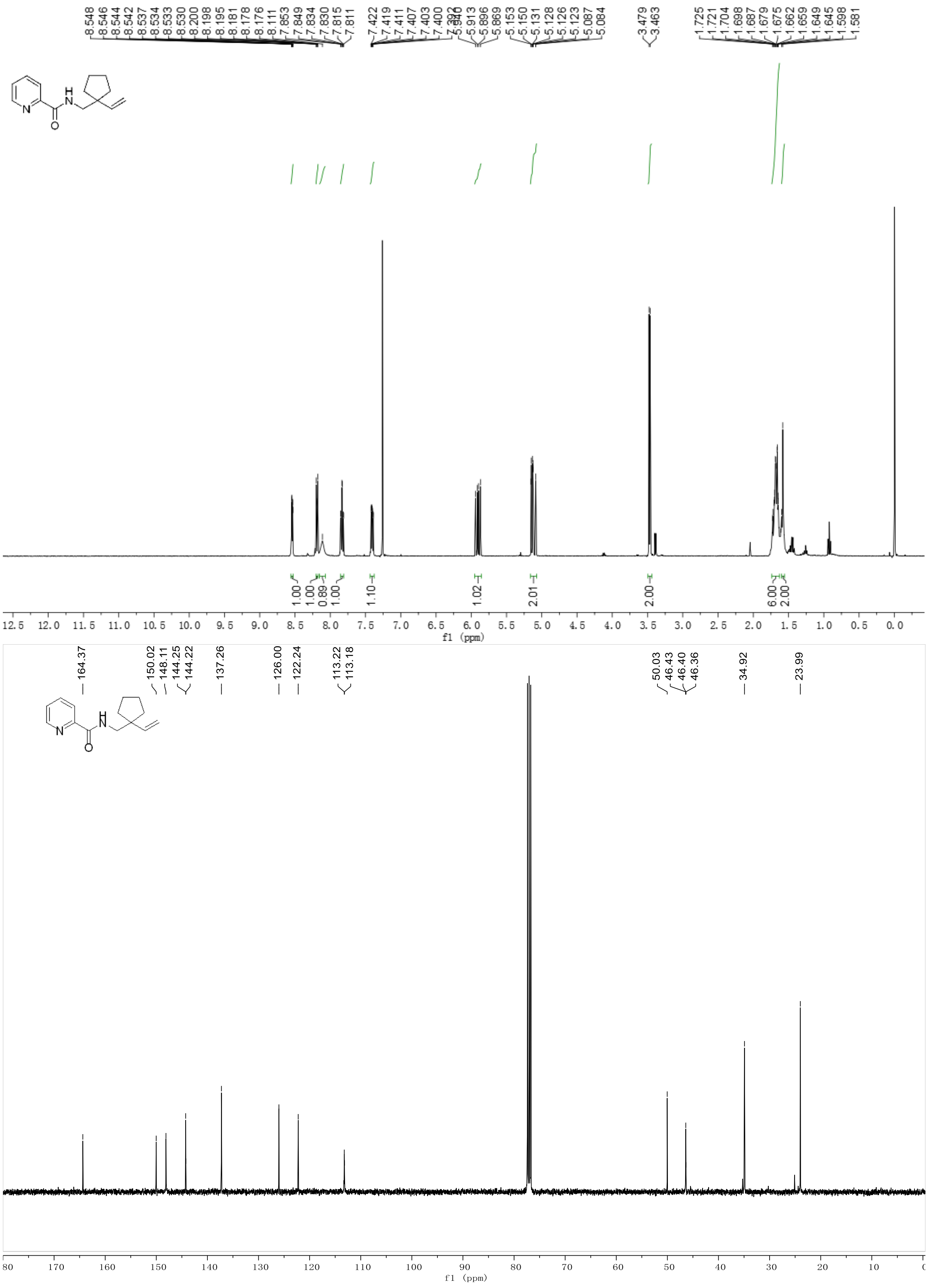

Supplementary Figure 9. ^{1}H NMR and ^{13}C NMR spectra of **1f**.

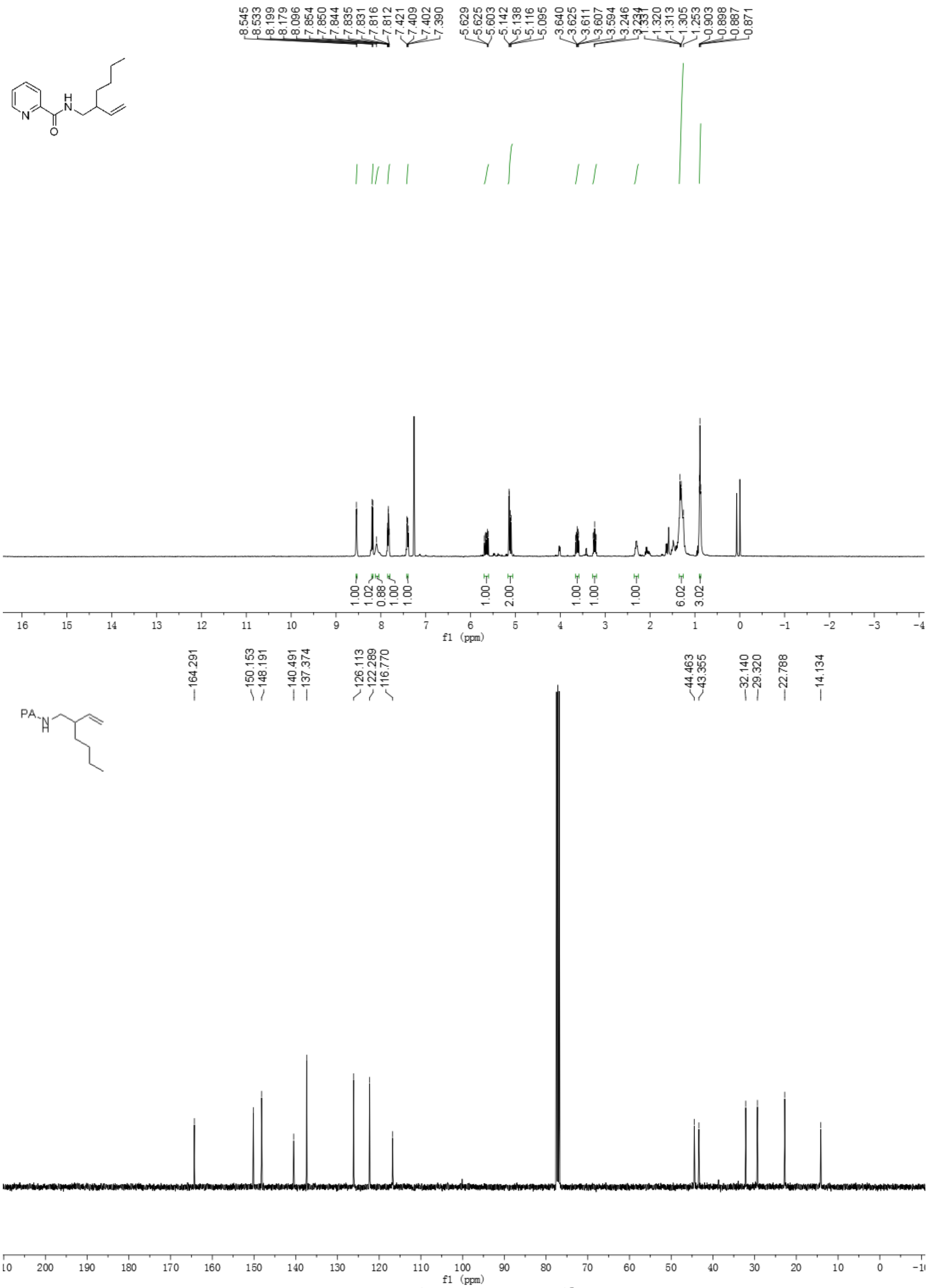

Supplementary Figure 10. ^{1}H NMR and ^{13}C NMR spectra of **1g**.

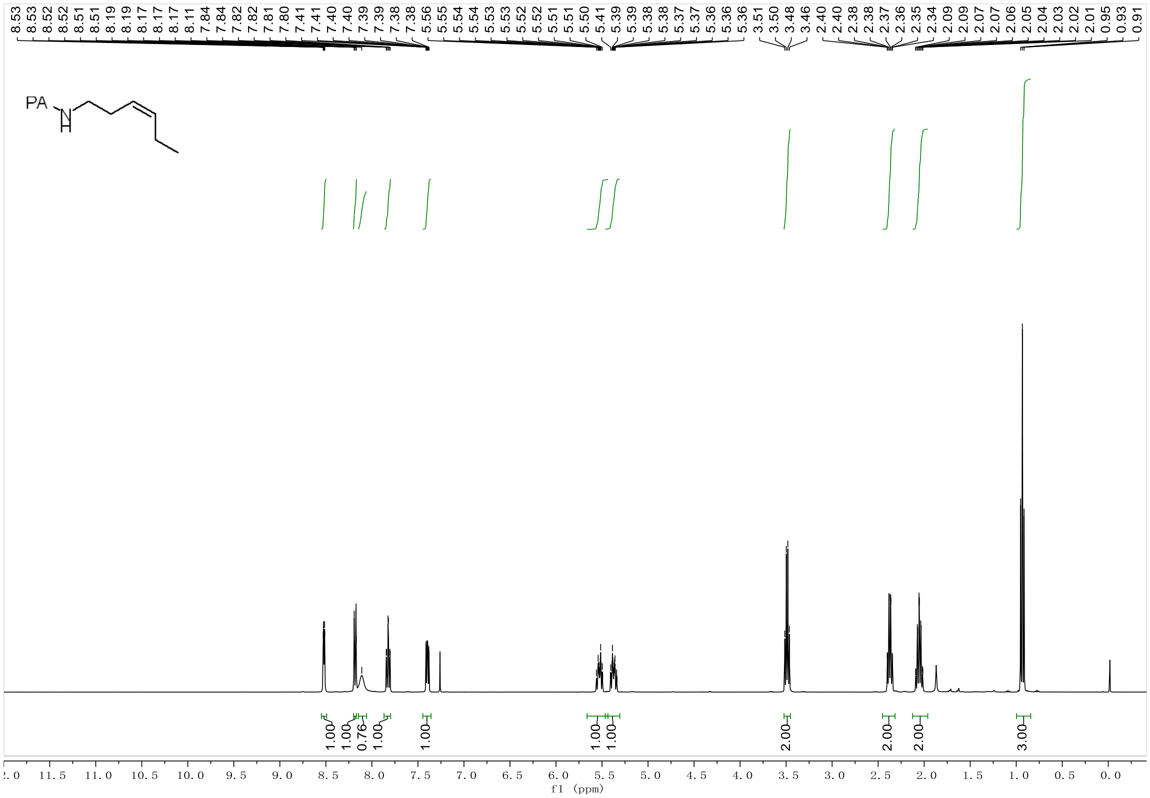

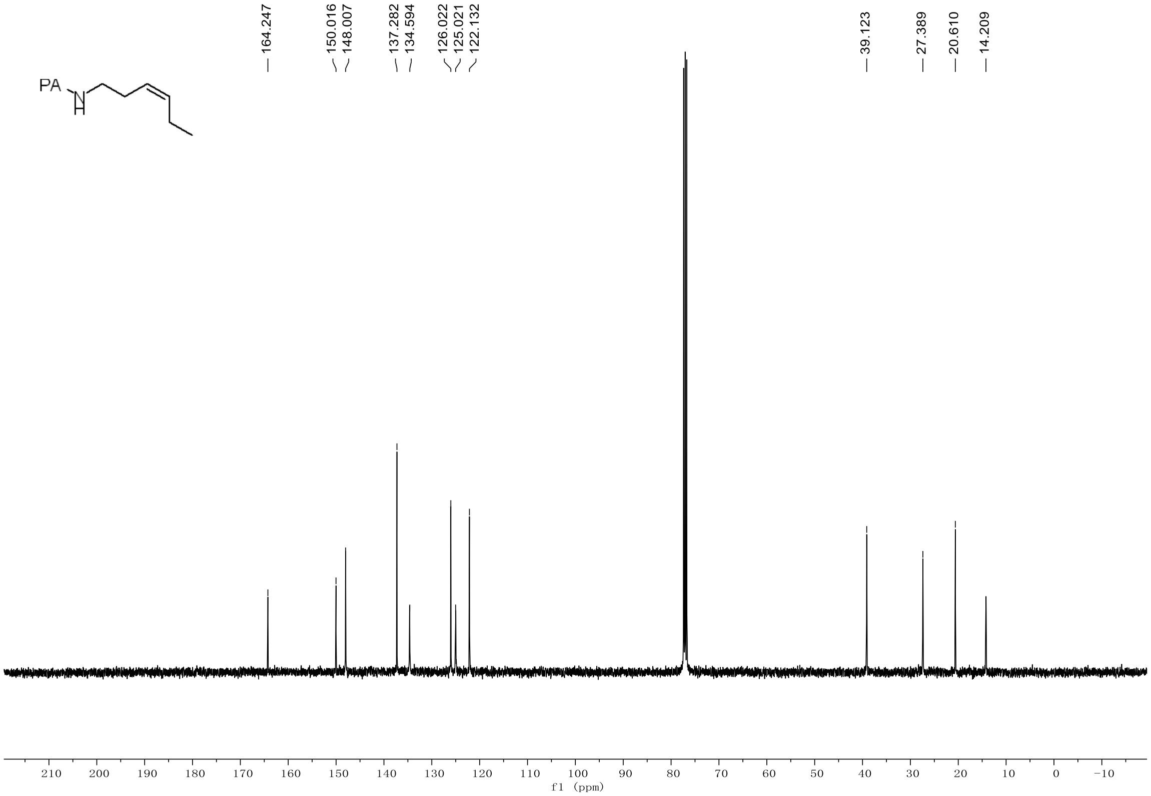

Supplementary Figure 11. ^{1}H NMR and ^{13}C NMR spectra of **1h**.

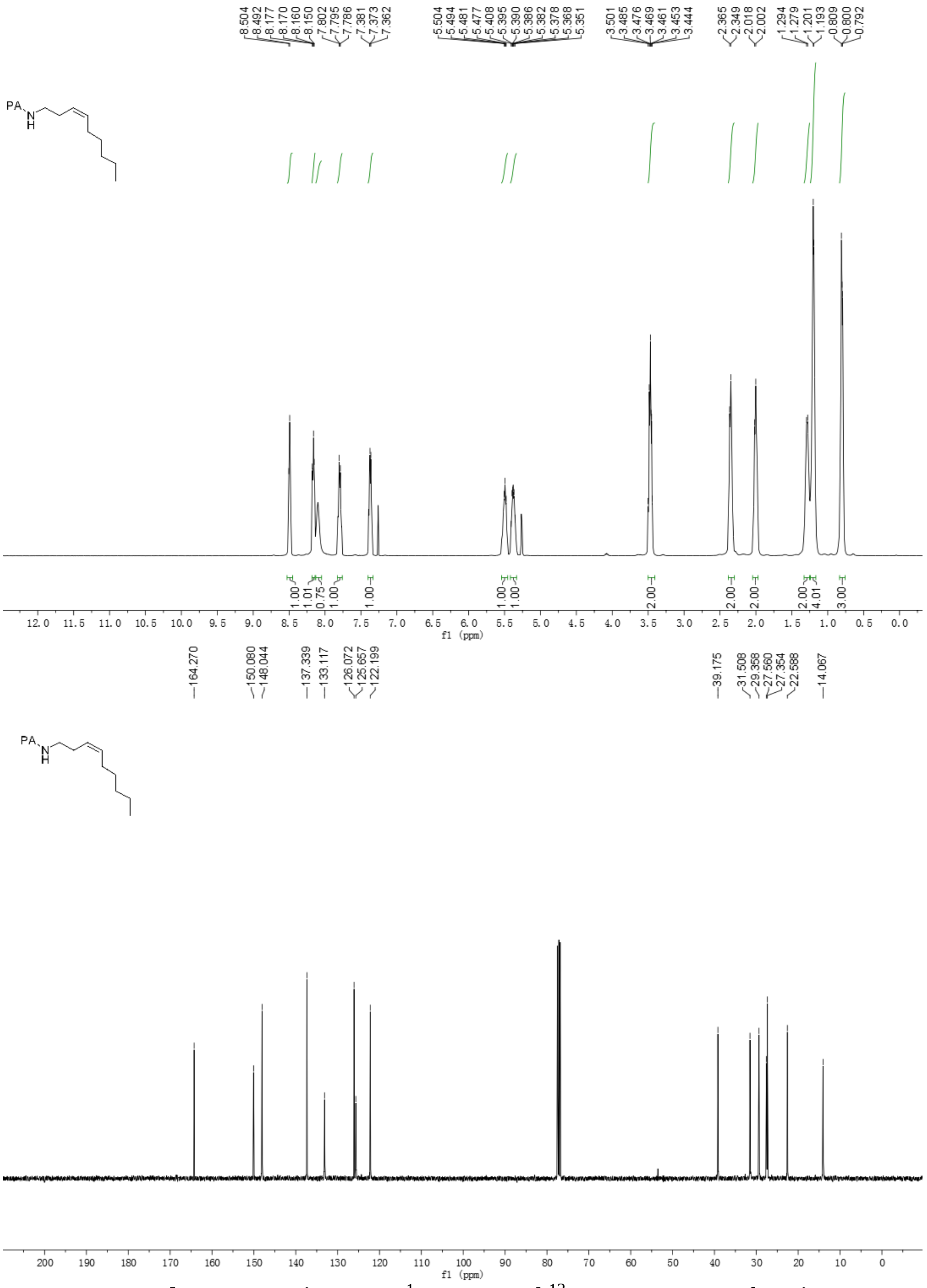

Supplementary Figure 12. ^{1}H NMR and ^{13}C NMR spectra of **Z-1i**.

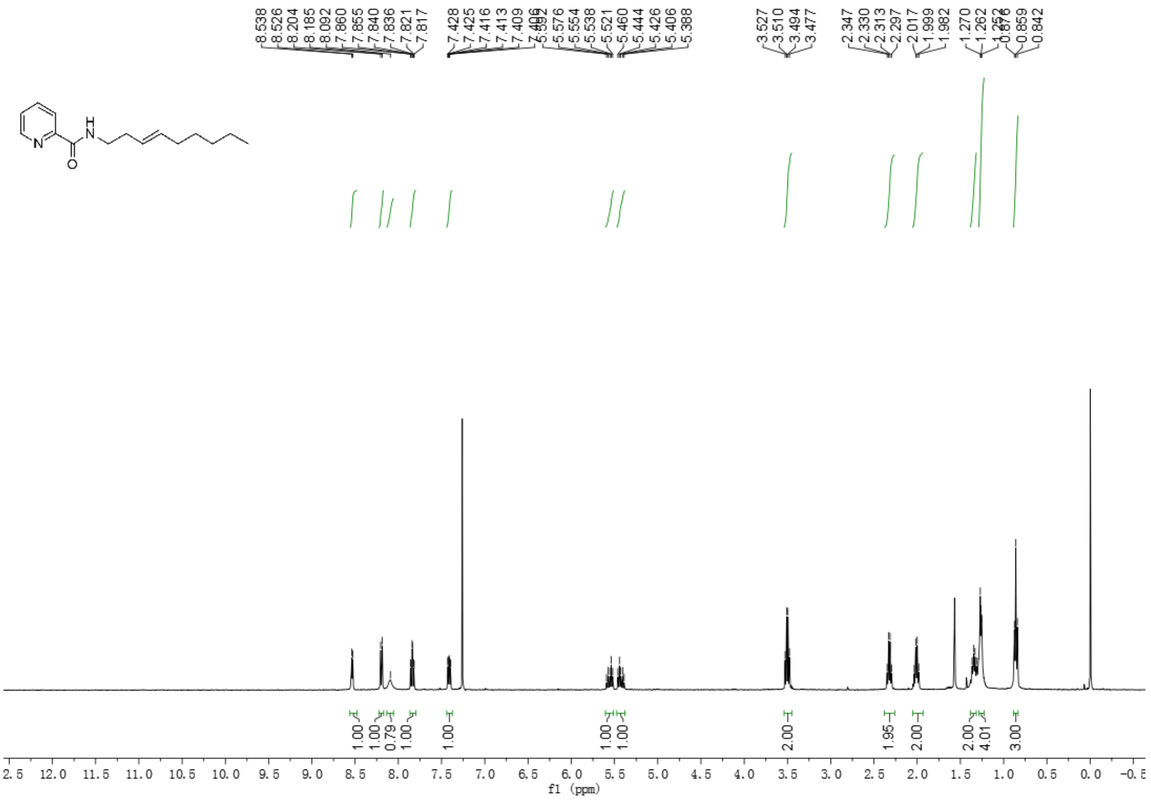

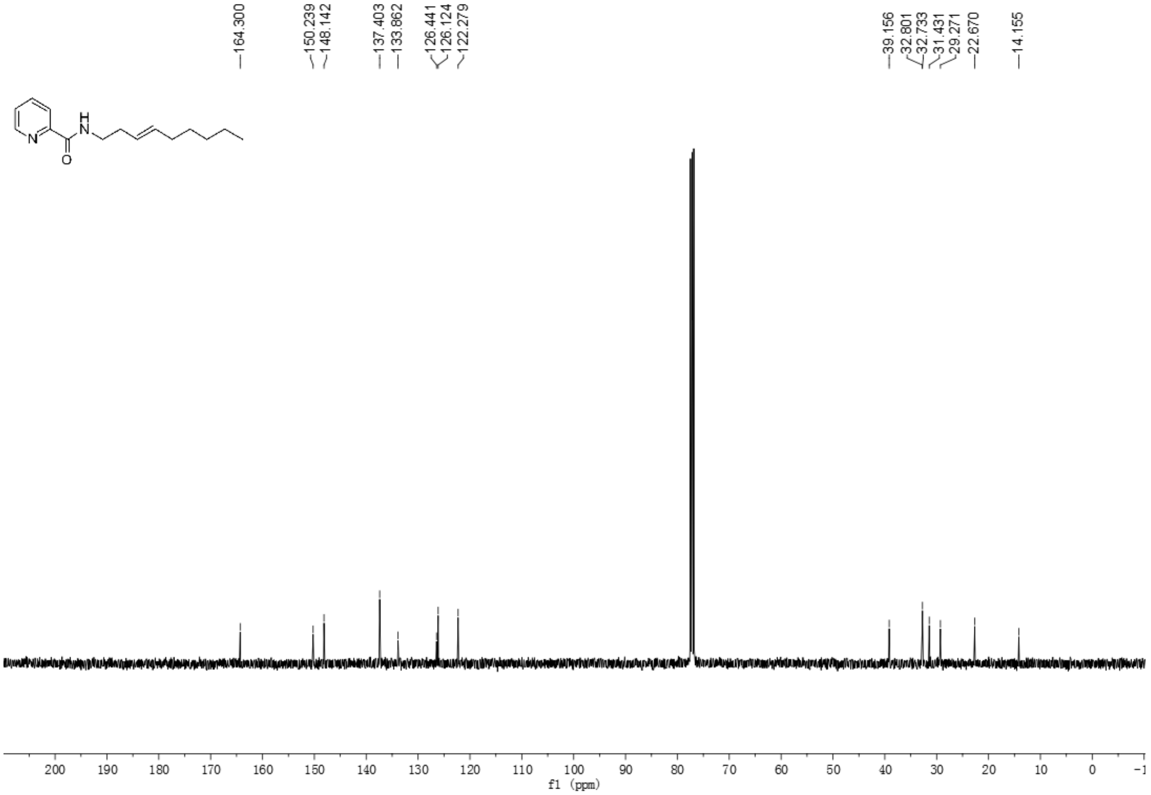

Supplementary Figure 13. ^{1}H NMR and ^{13}C NMR spectra of **E-1i**.

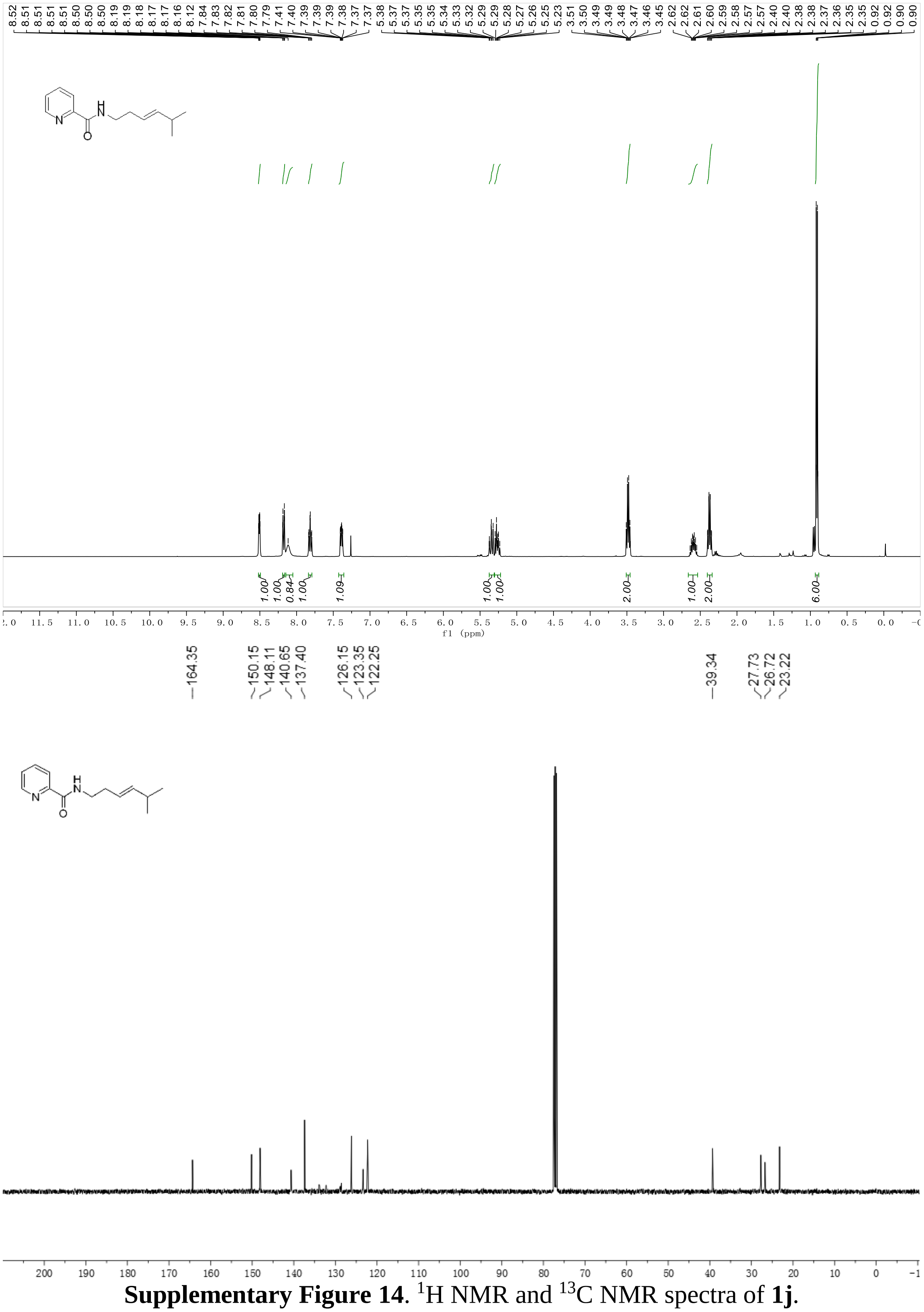

Supplementary Figure 14. ^{1}H NMR and ^{13}C NMR spectra of **1j**.

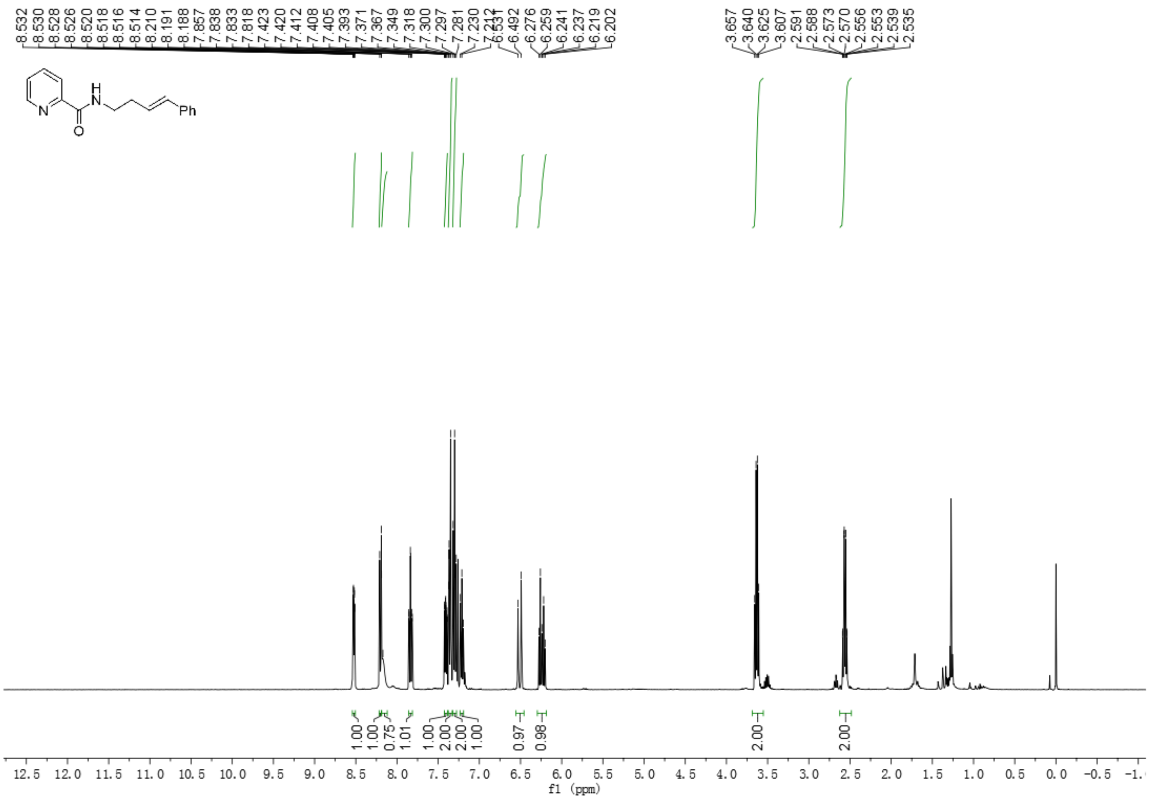

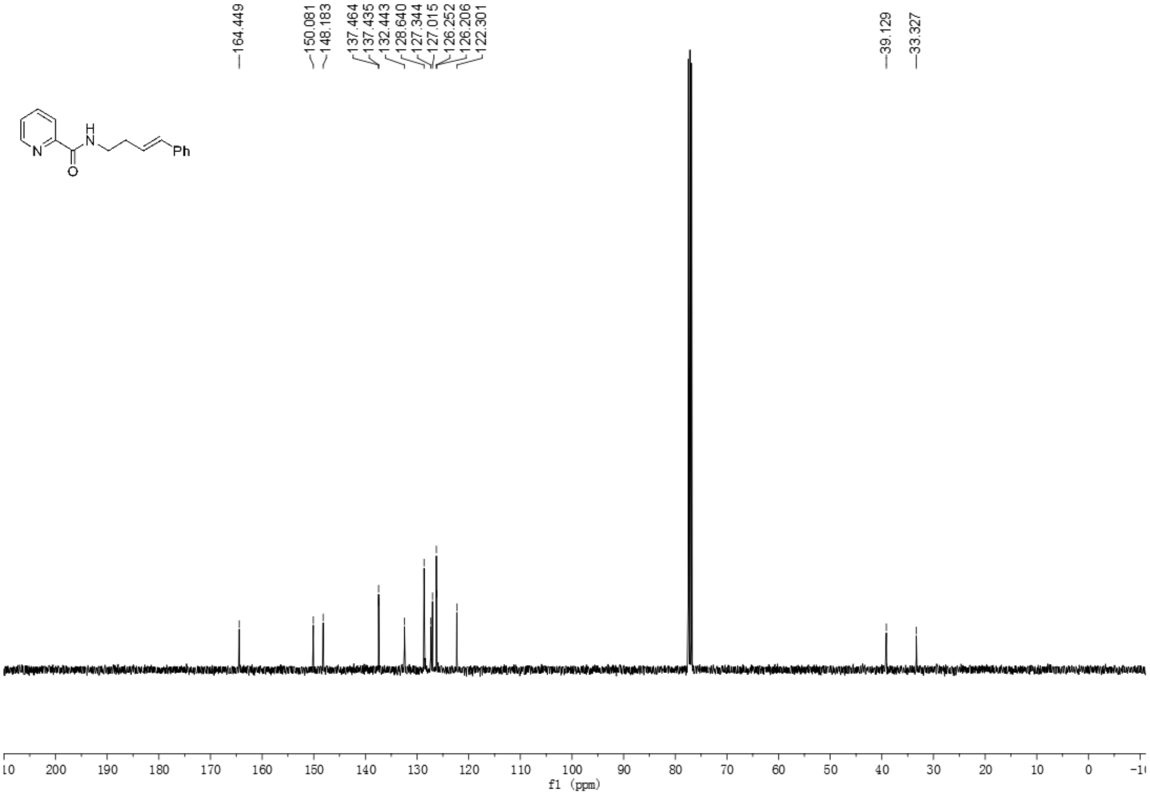

Supplementary Figure 15. ^{1}H NMR and ^{13}C NMR spectra of **1k**.

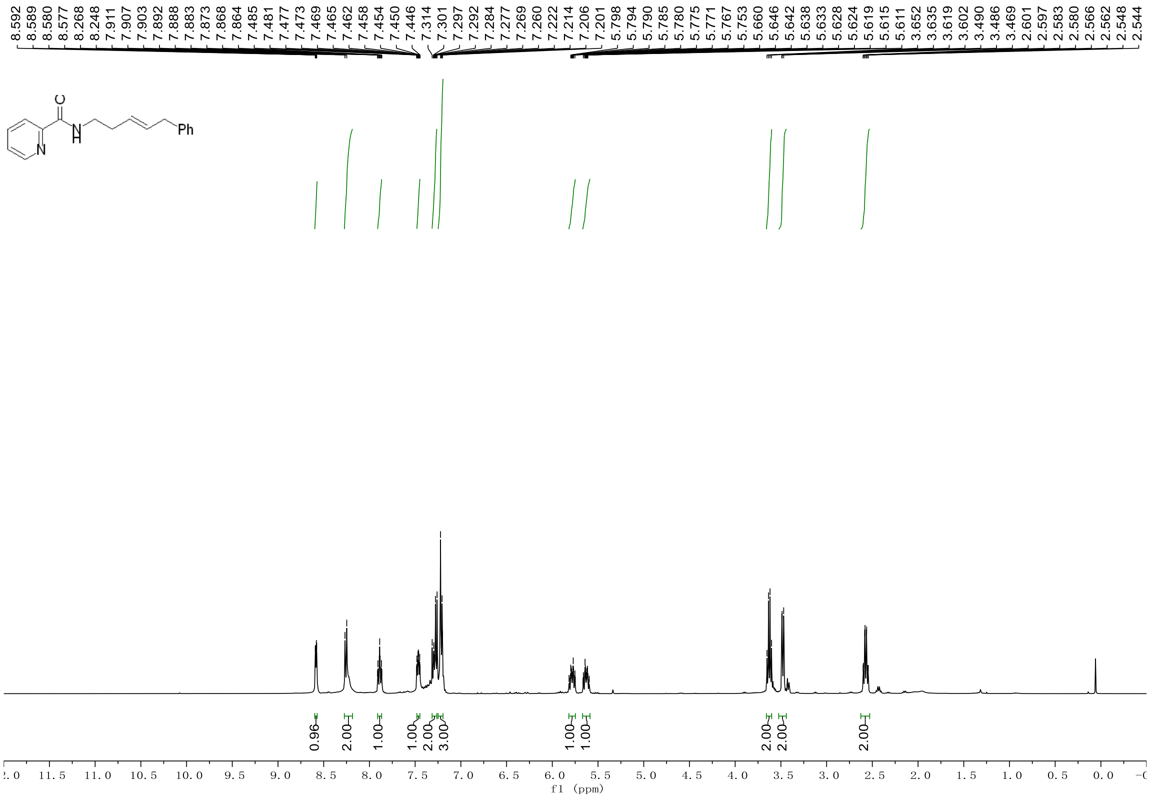

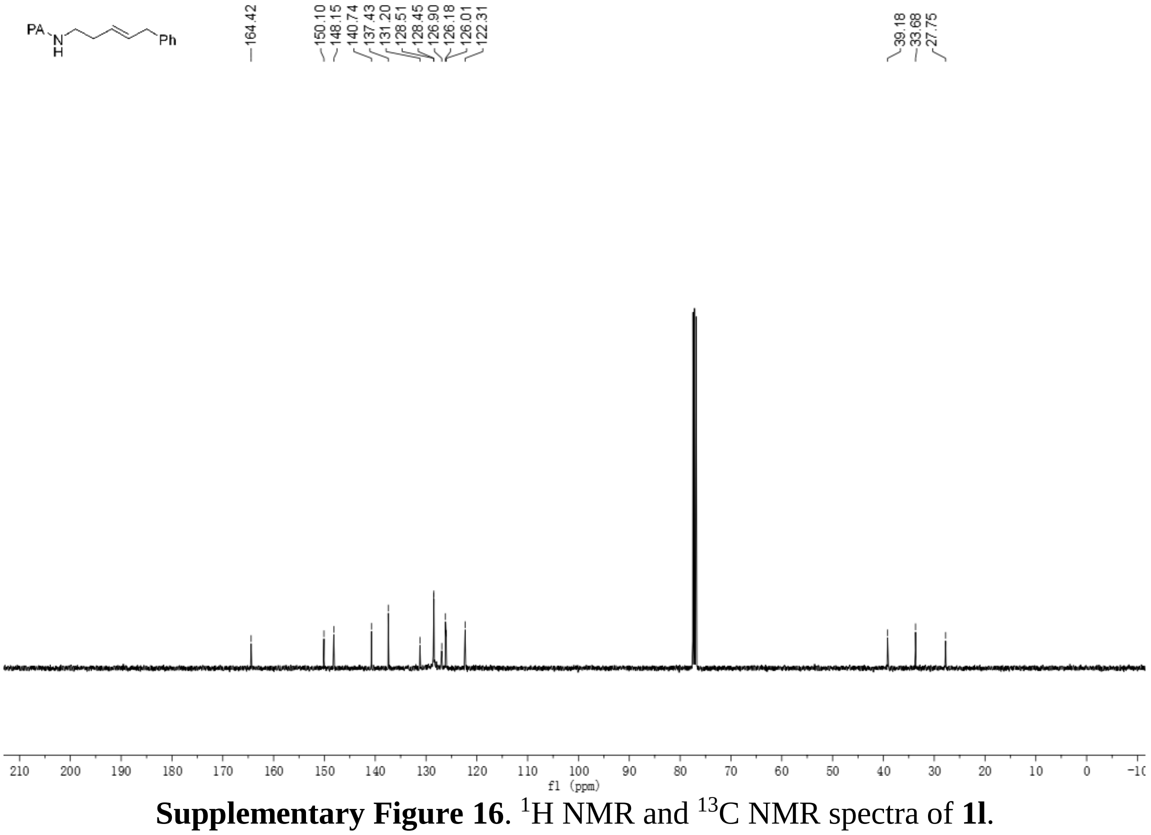

Supplementary Figure 16. ^{1}H NMR and ^{13}C NMR spectra of **1l**.

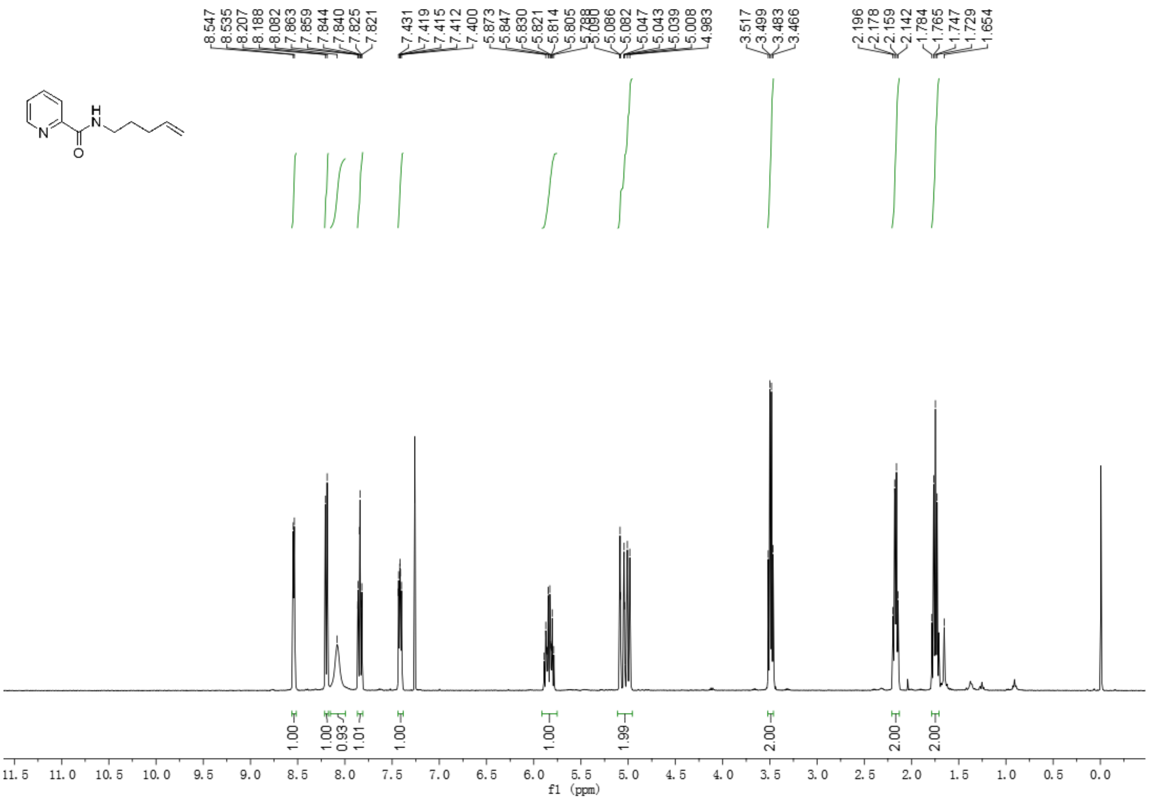

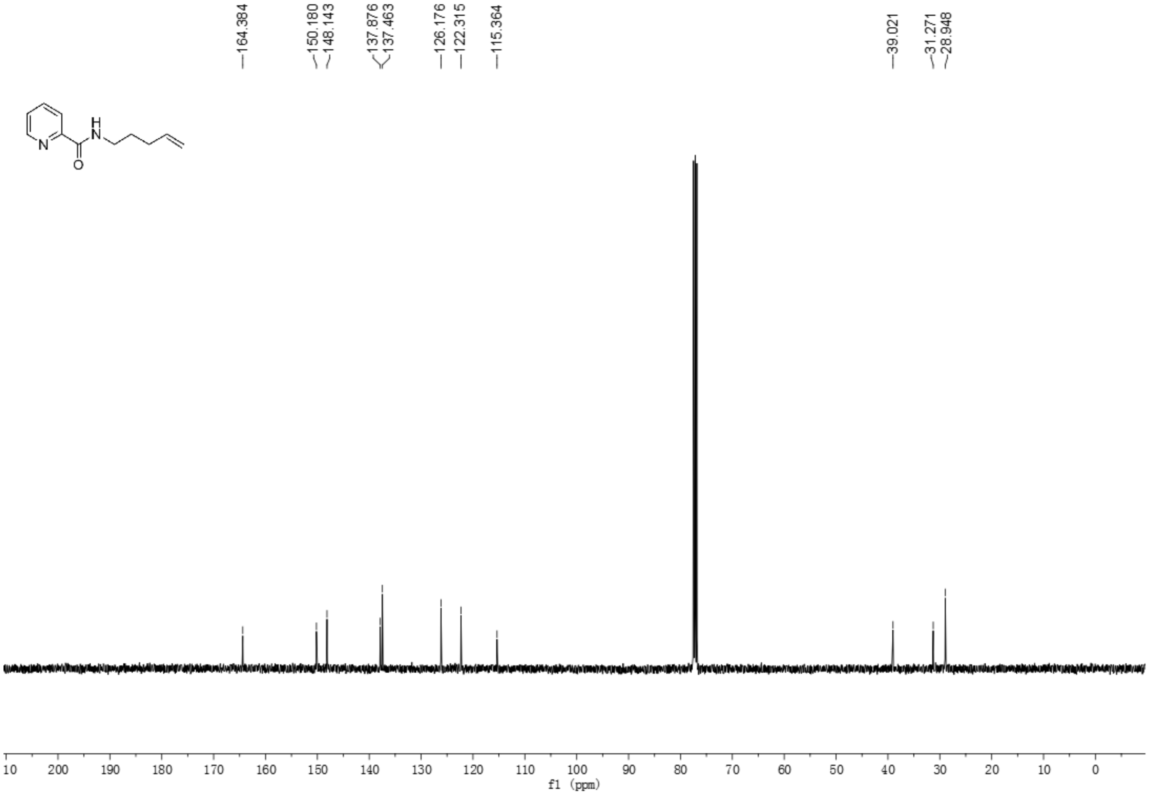

Supplementary Figure 17. ^{1}H NMR and ^{13}C NMR spectra of **1m**.

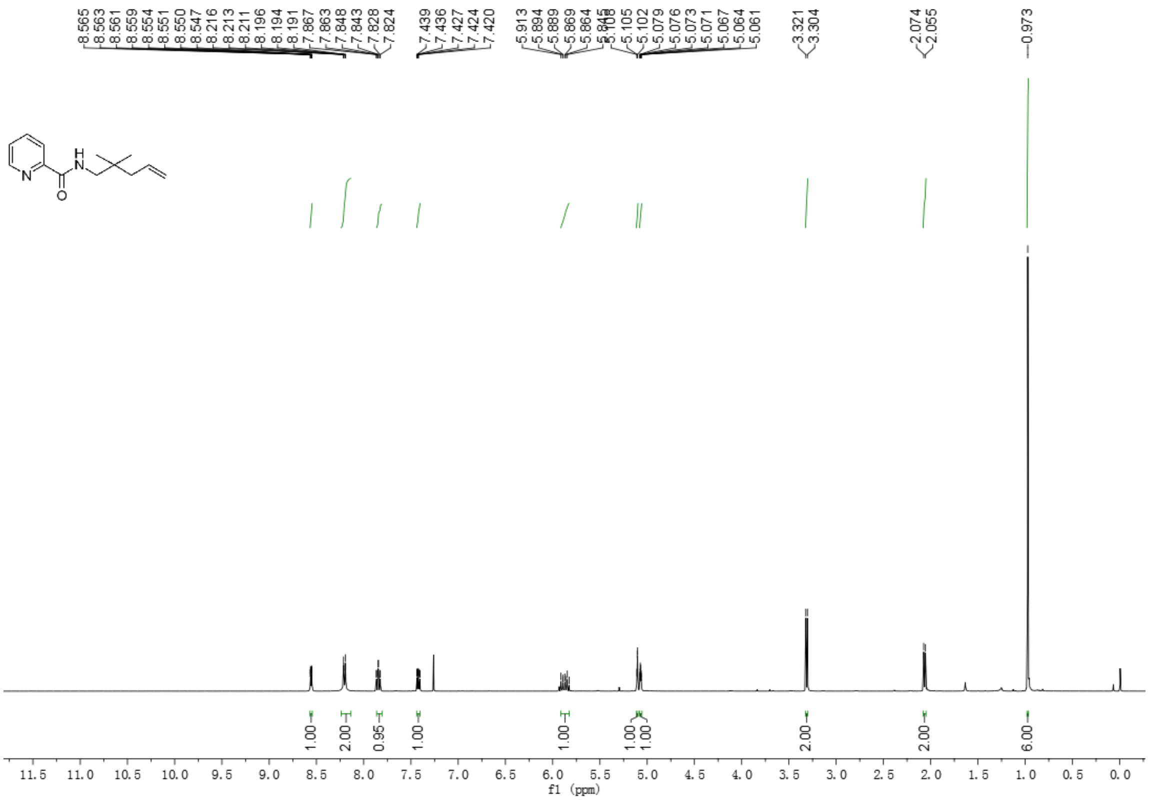

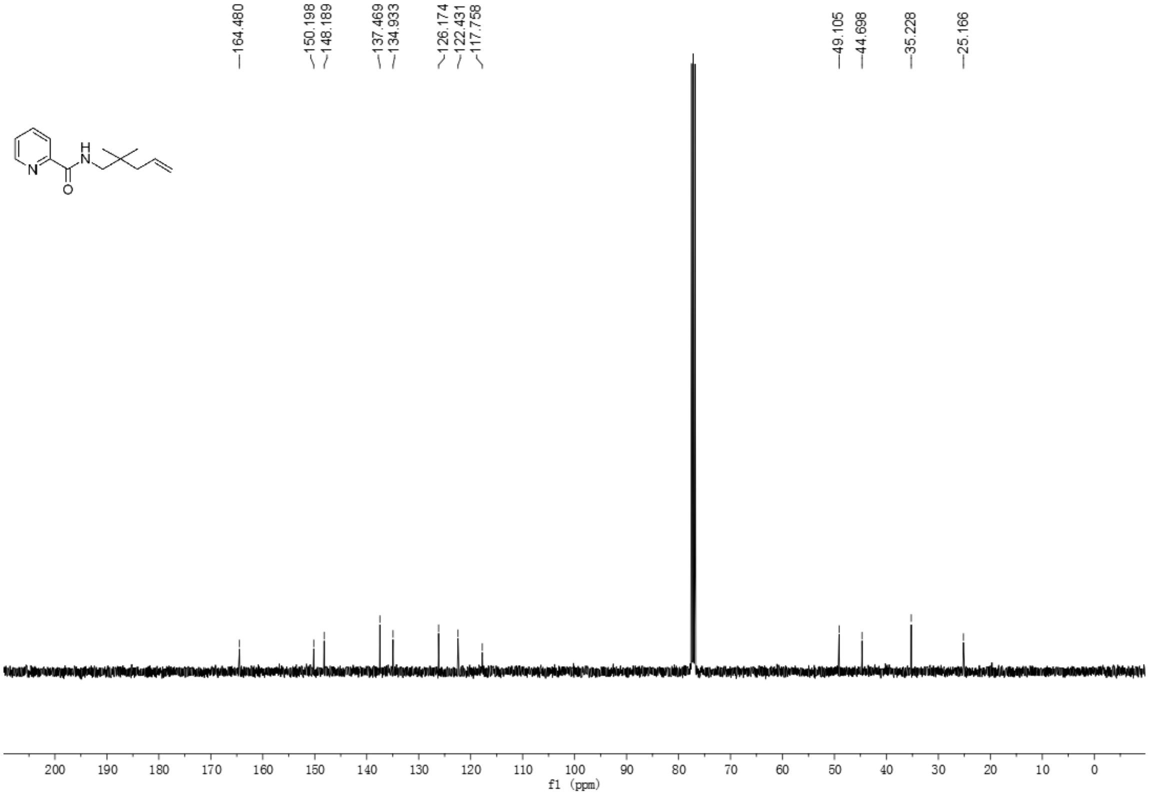

Supplementary Figure 18. ^{1}H NMR and ^{13}C NMR spectra of **1n**.

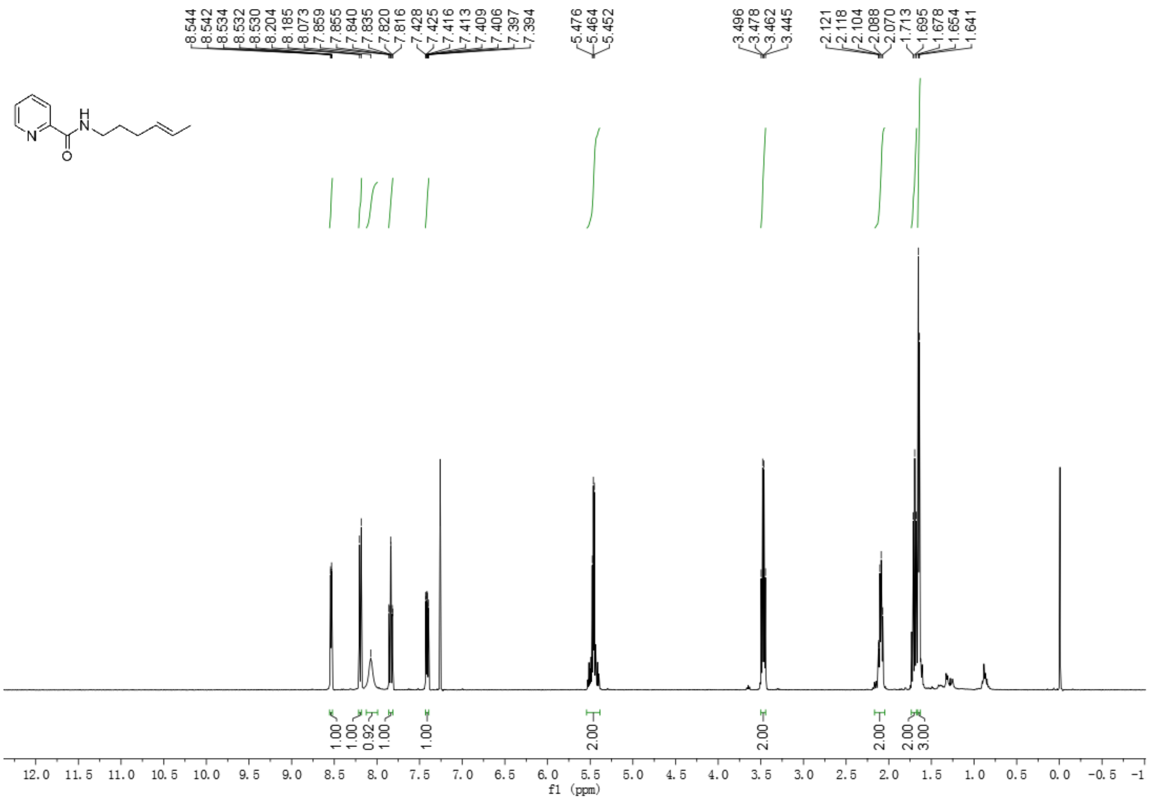

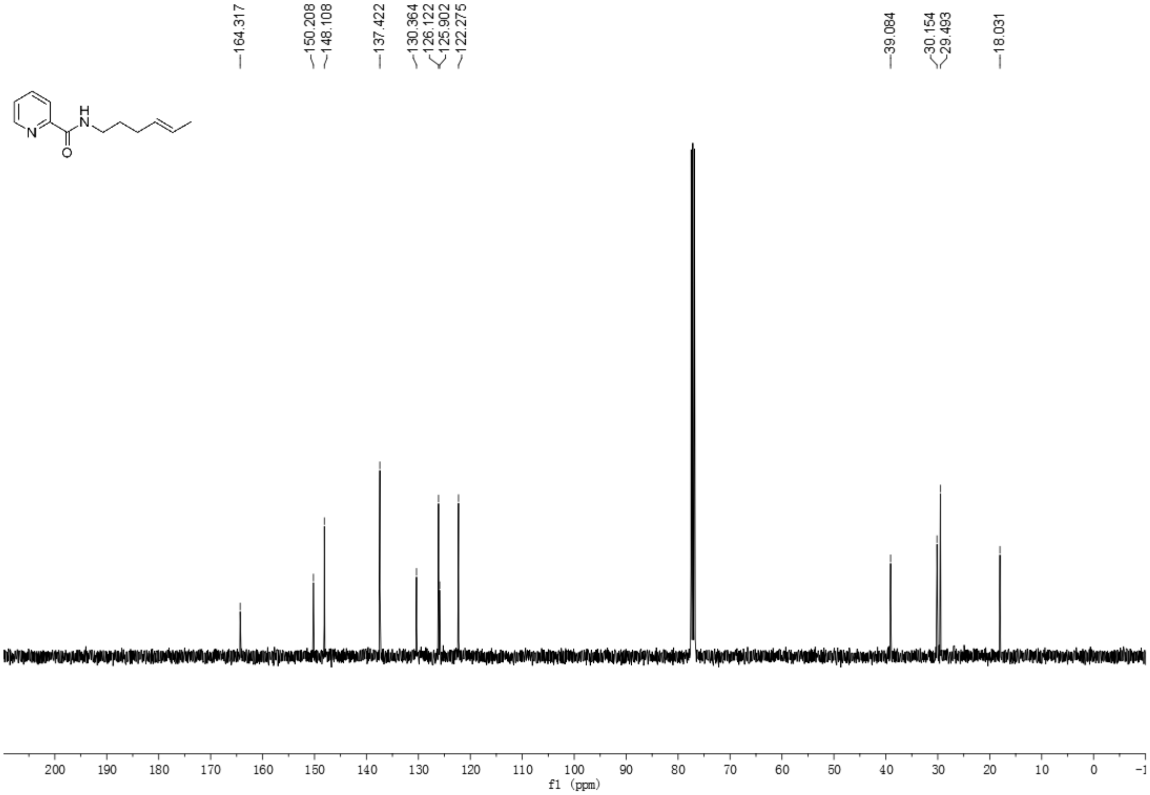

Supplementary Figure 19. ^{1}H NMR and ^{13}C NMR spectra of **1o**.

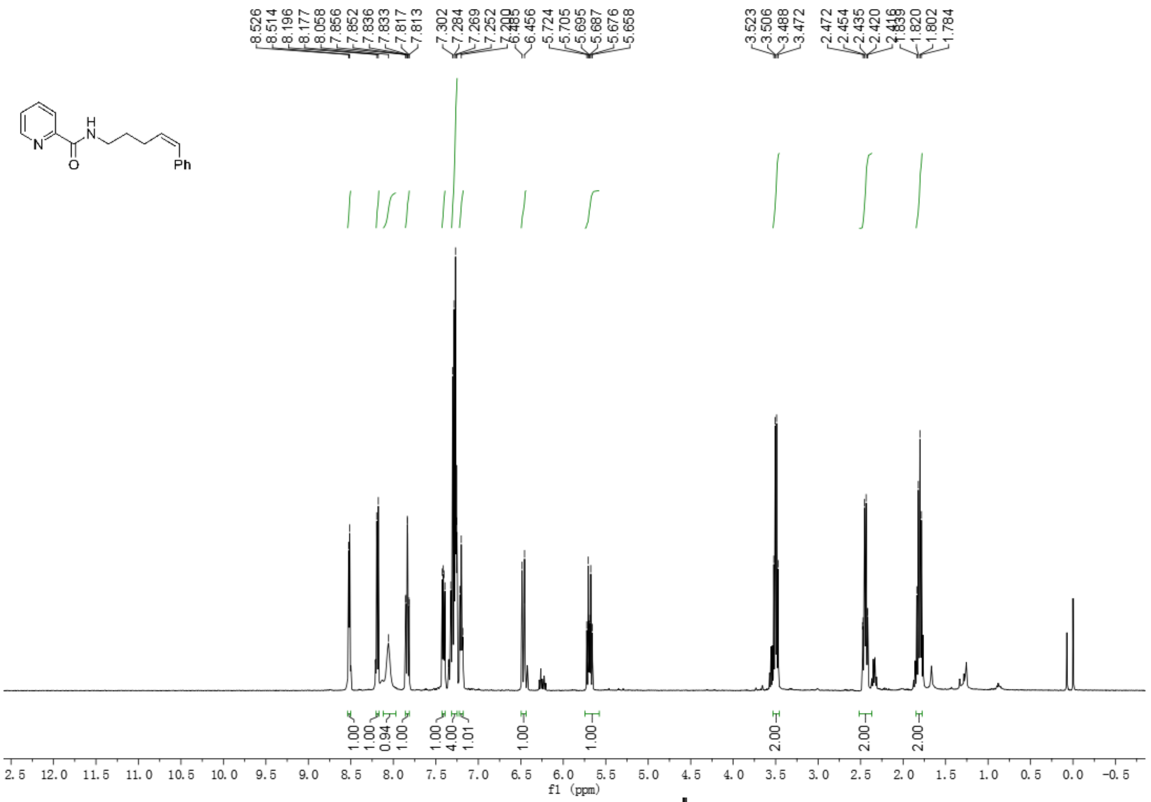

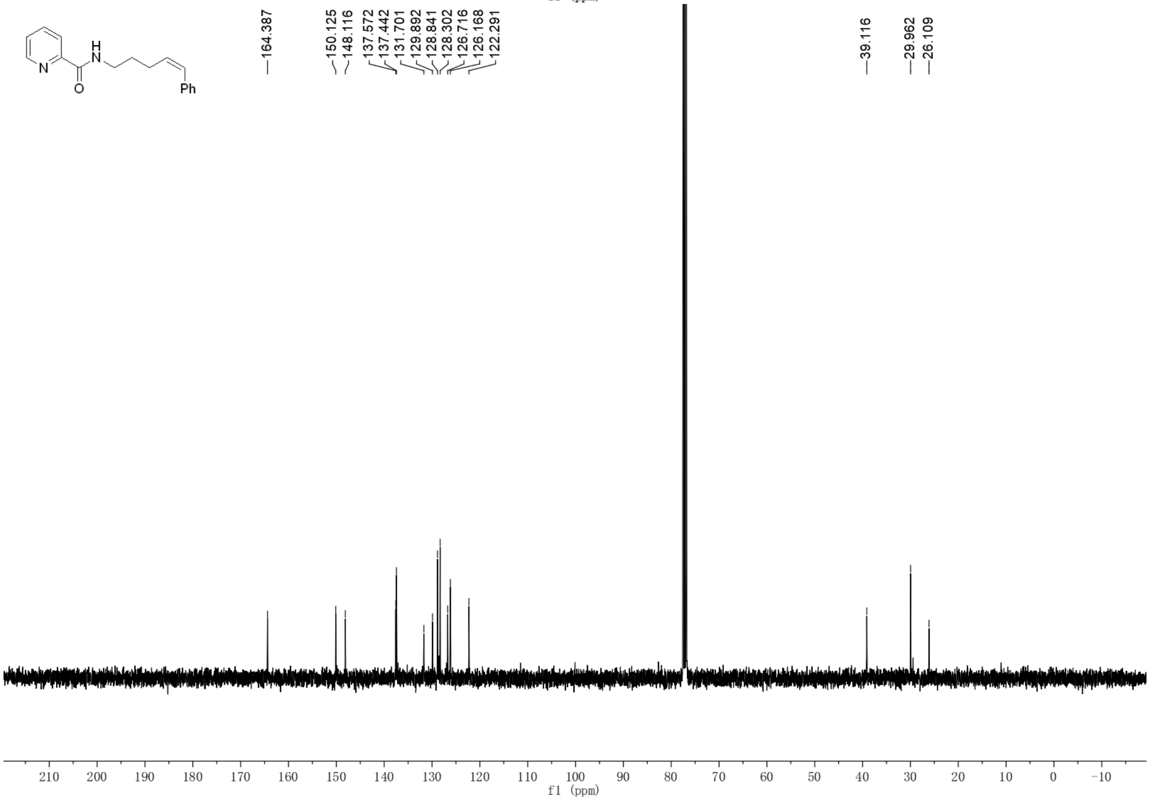

Supplementary Figure 20. ^{1}H NMR and ^{13}C NMR spectra of **1p**.

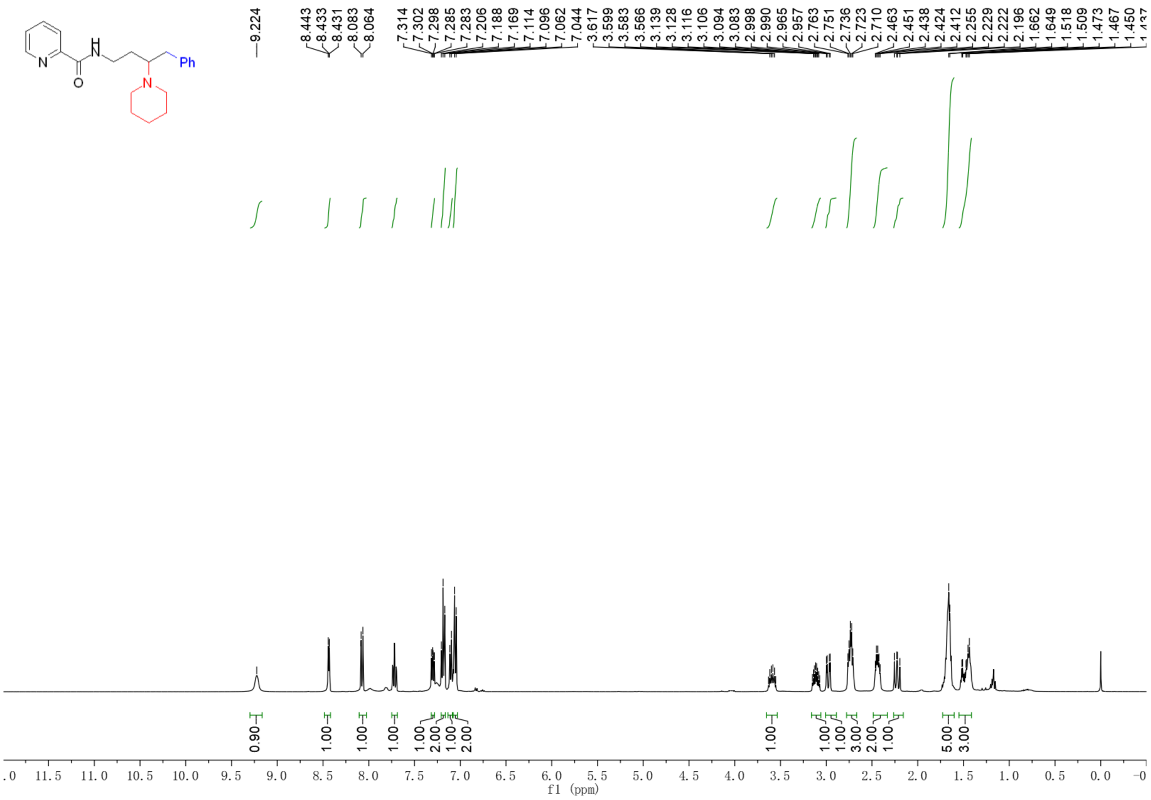

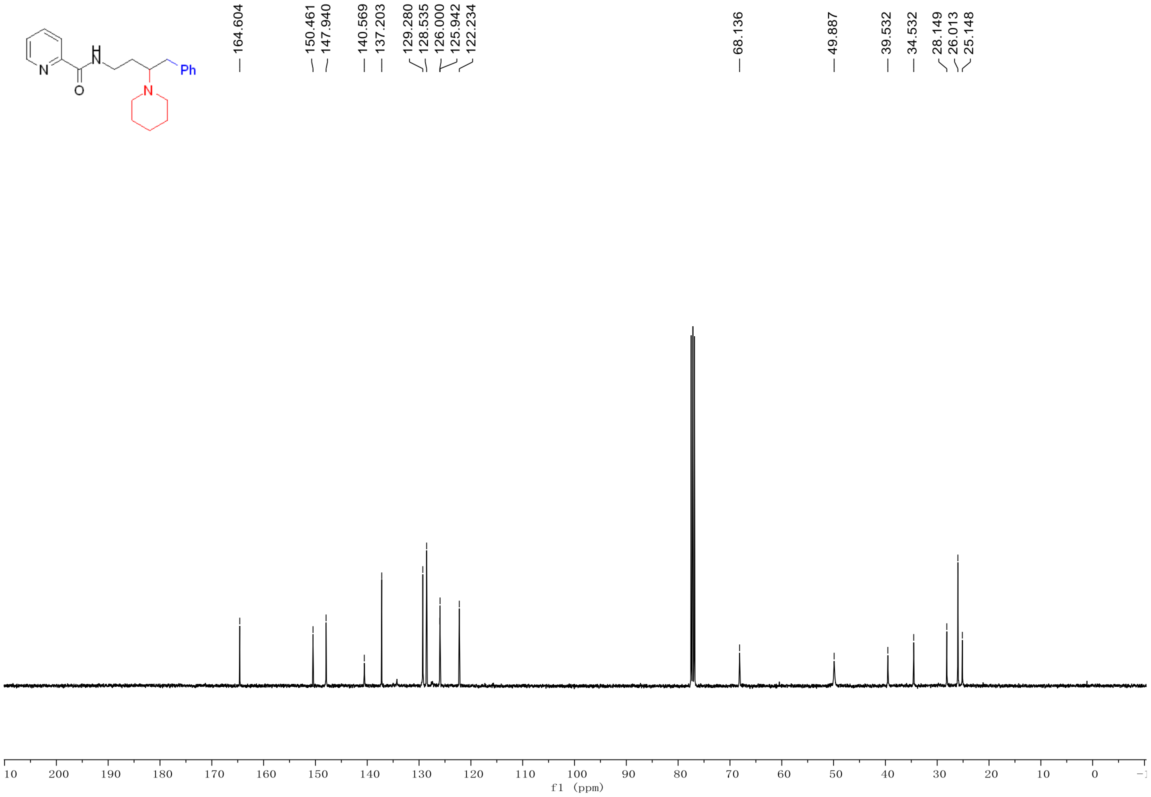

Supplementary Figure 21. ^{1}H NMR and ^{13}C NMR spectra of **2a**.

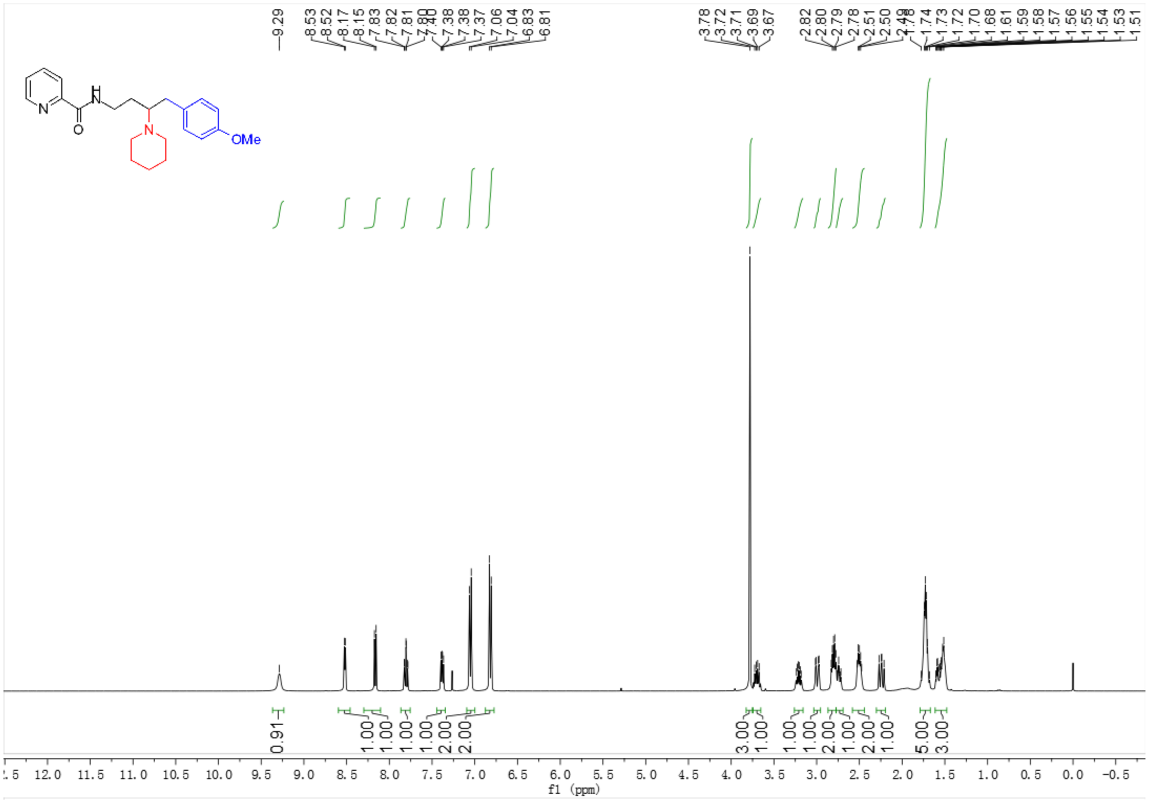

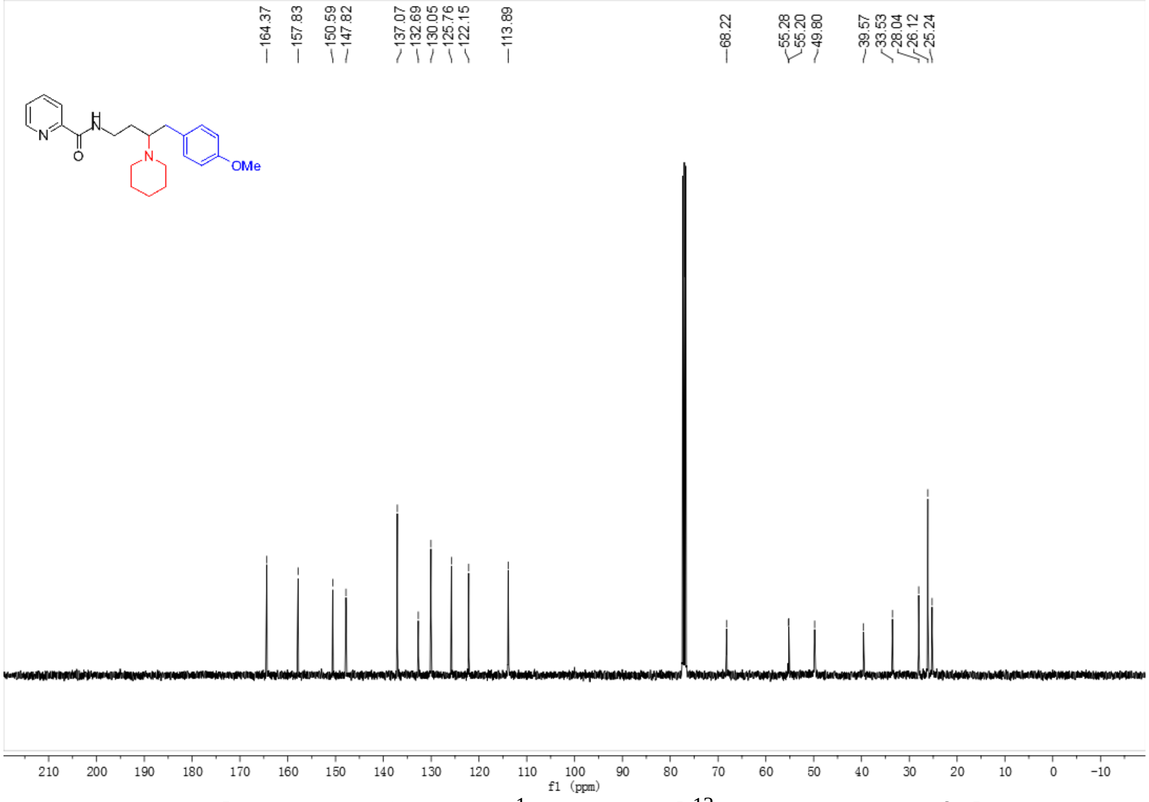

Supplementary Figure 22. ^{1}H NMR and ^{13}C NMR spectra of **2b**.

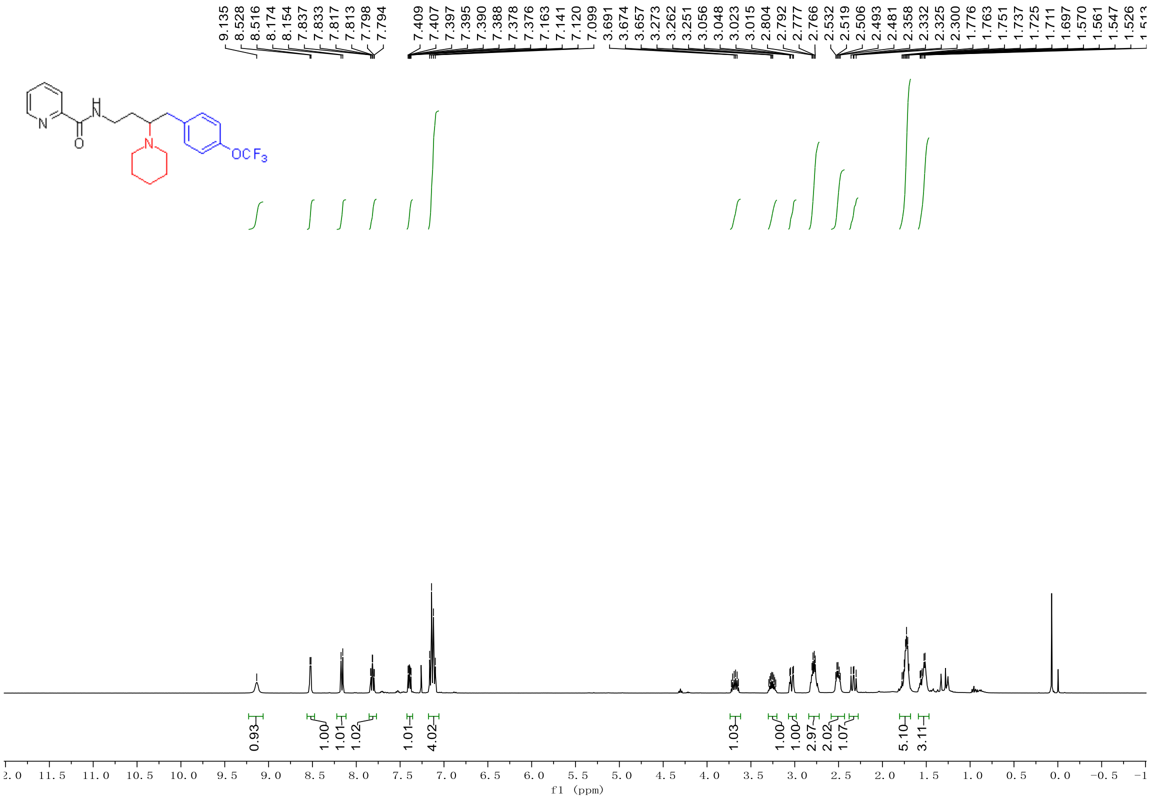

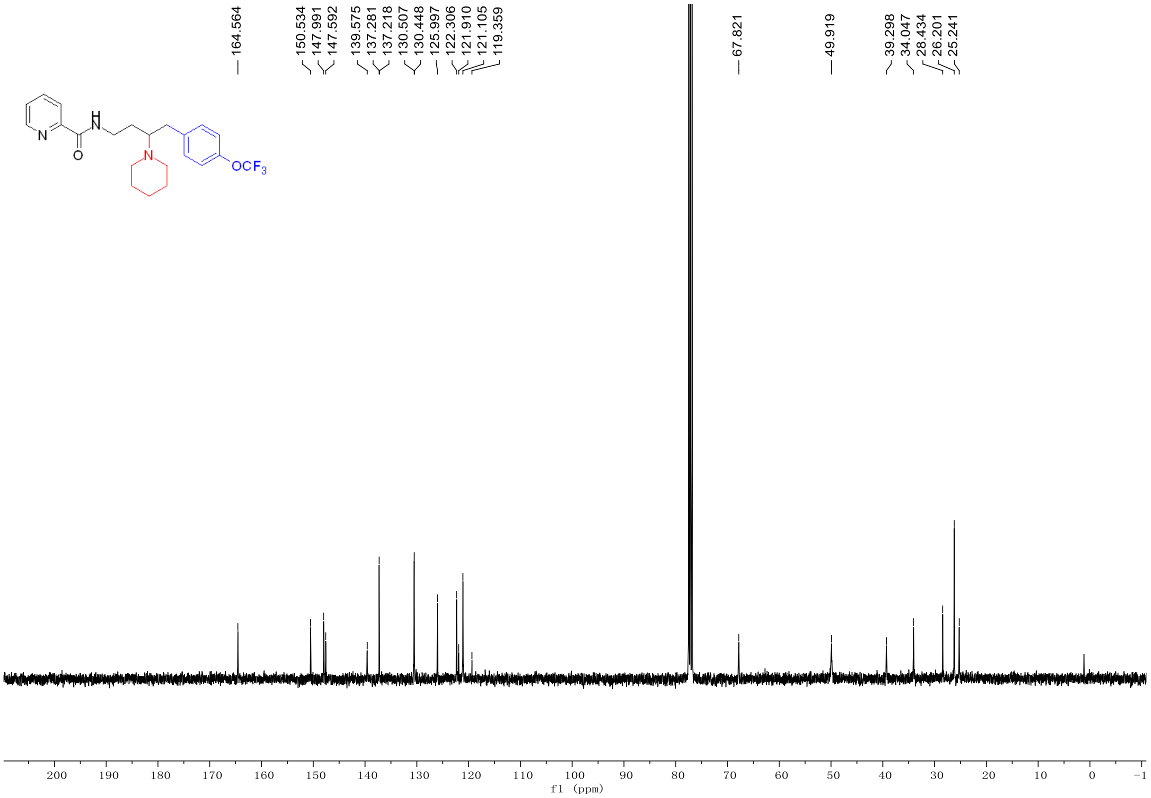

Supplementary Figure 23. ^{1}H NMR and ^{13}C NMR spectra of **2c**.

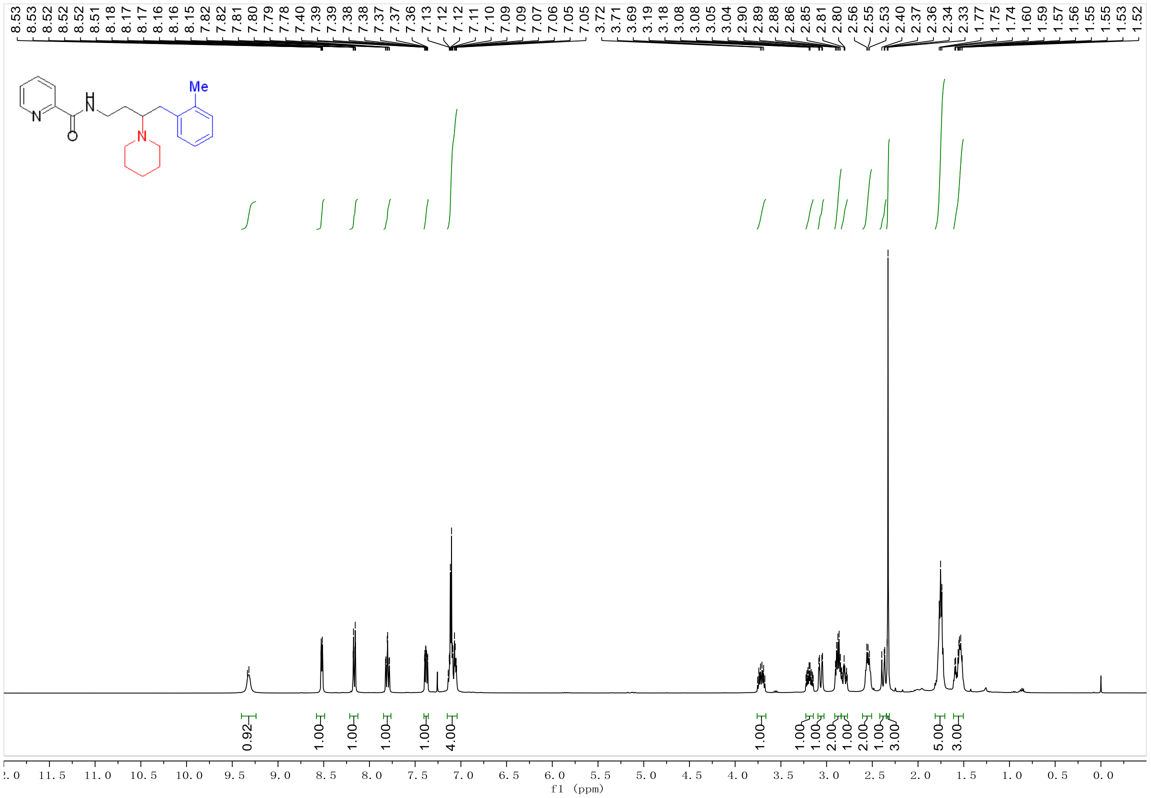

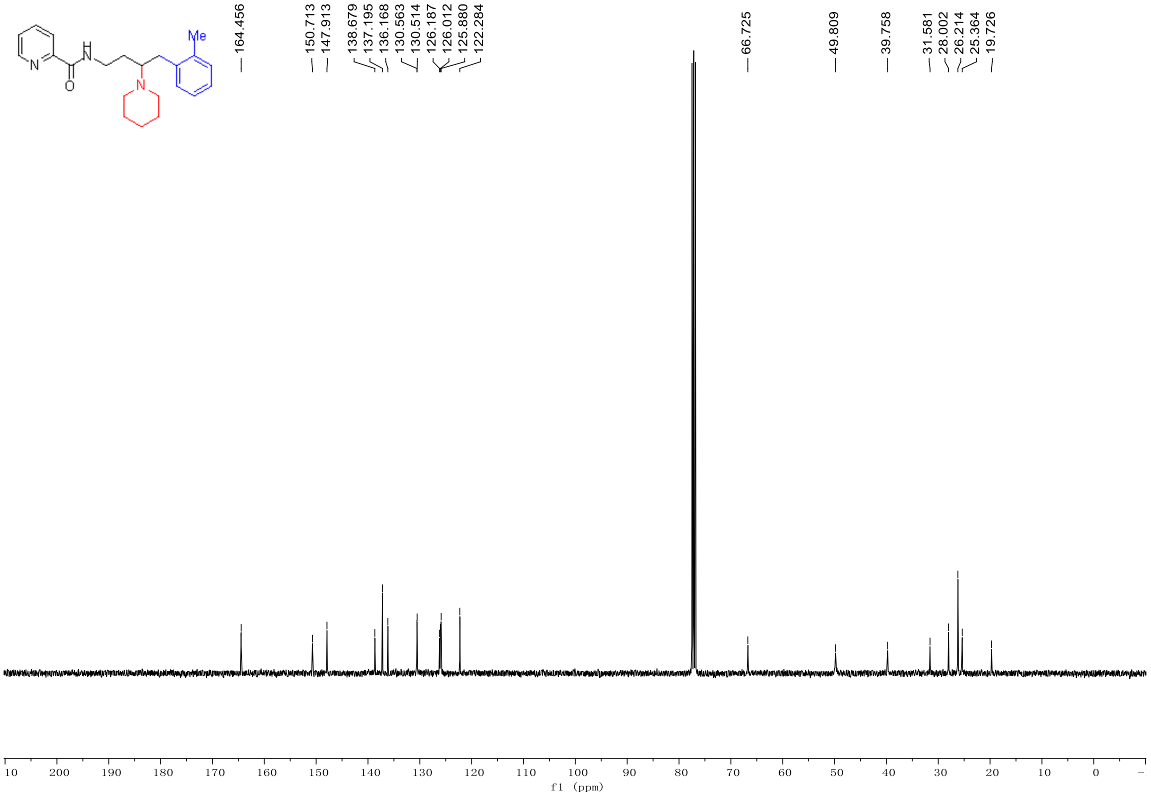

Supplementary Figure 24. ^{1}H NMR and ^{13}C NMR spectra of **2d**.

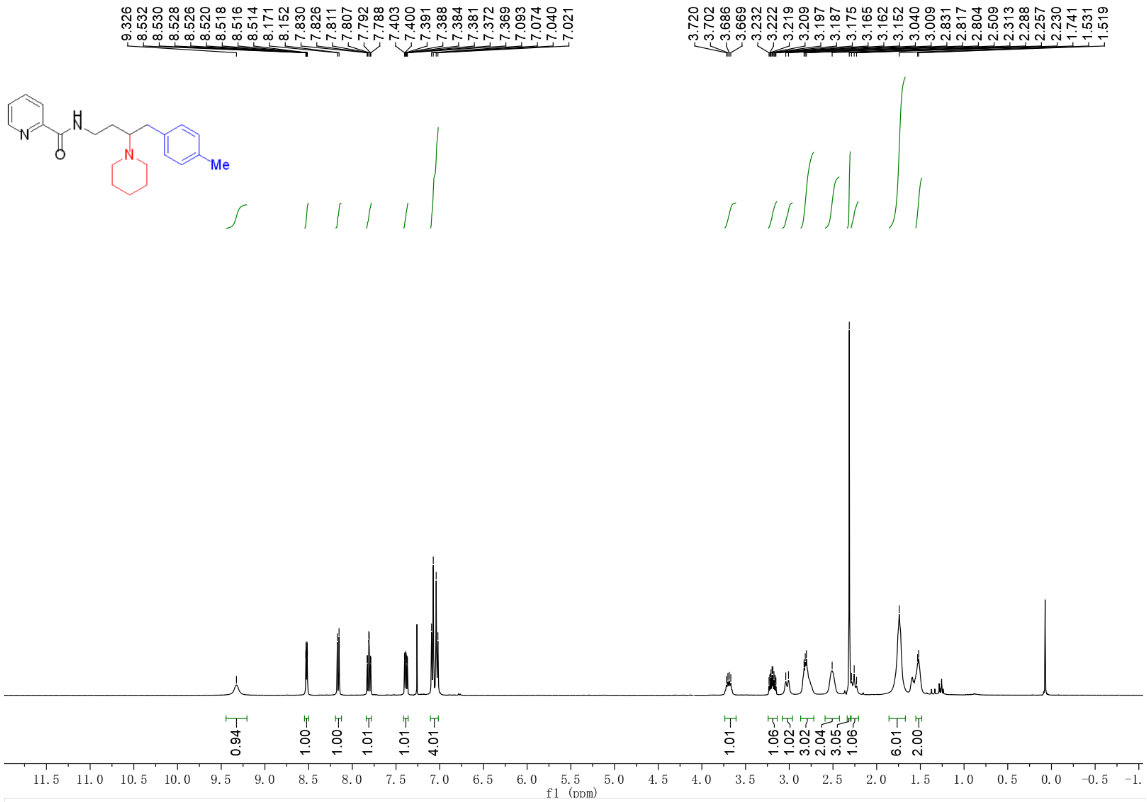

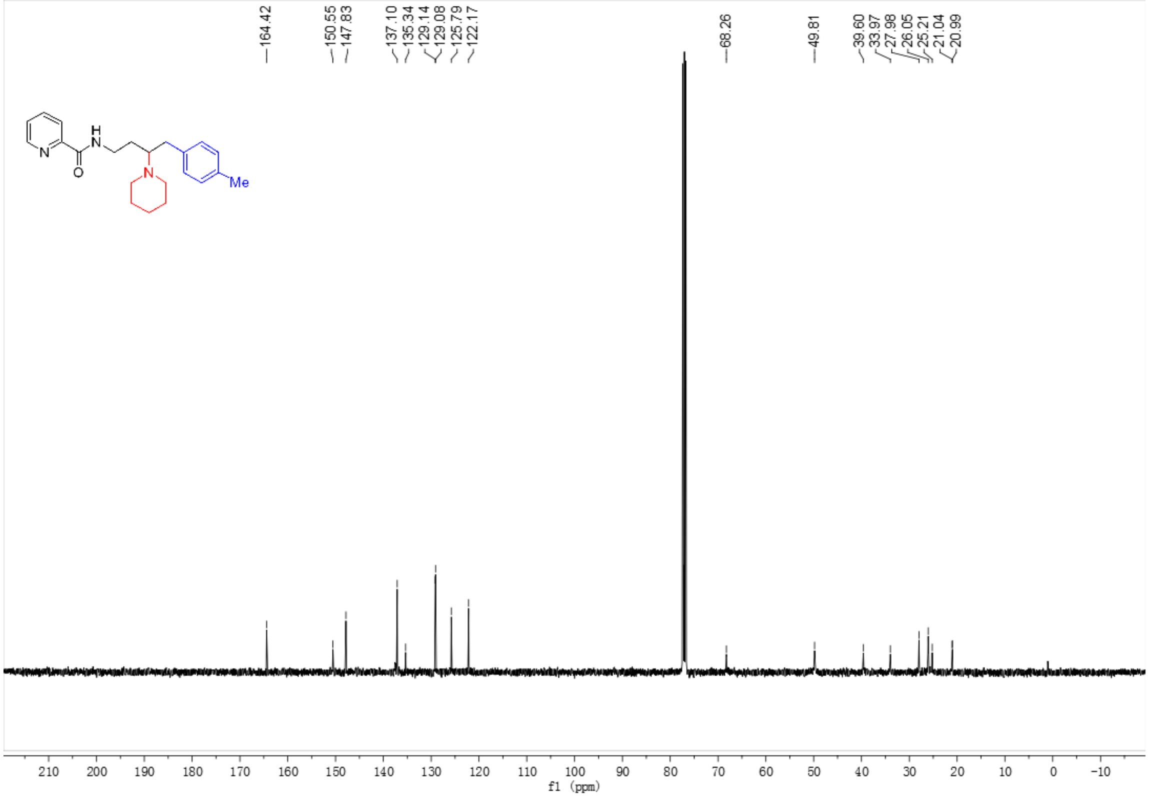

Supplementary Figure 25. ^{1}H NMR and ^{13}C NMR spectra of **2e**.

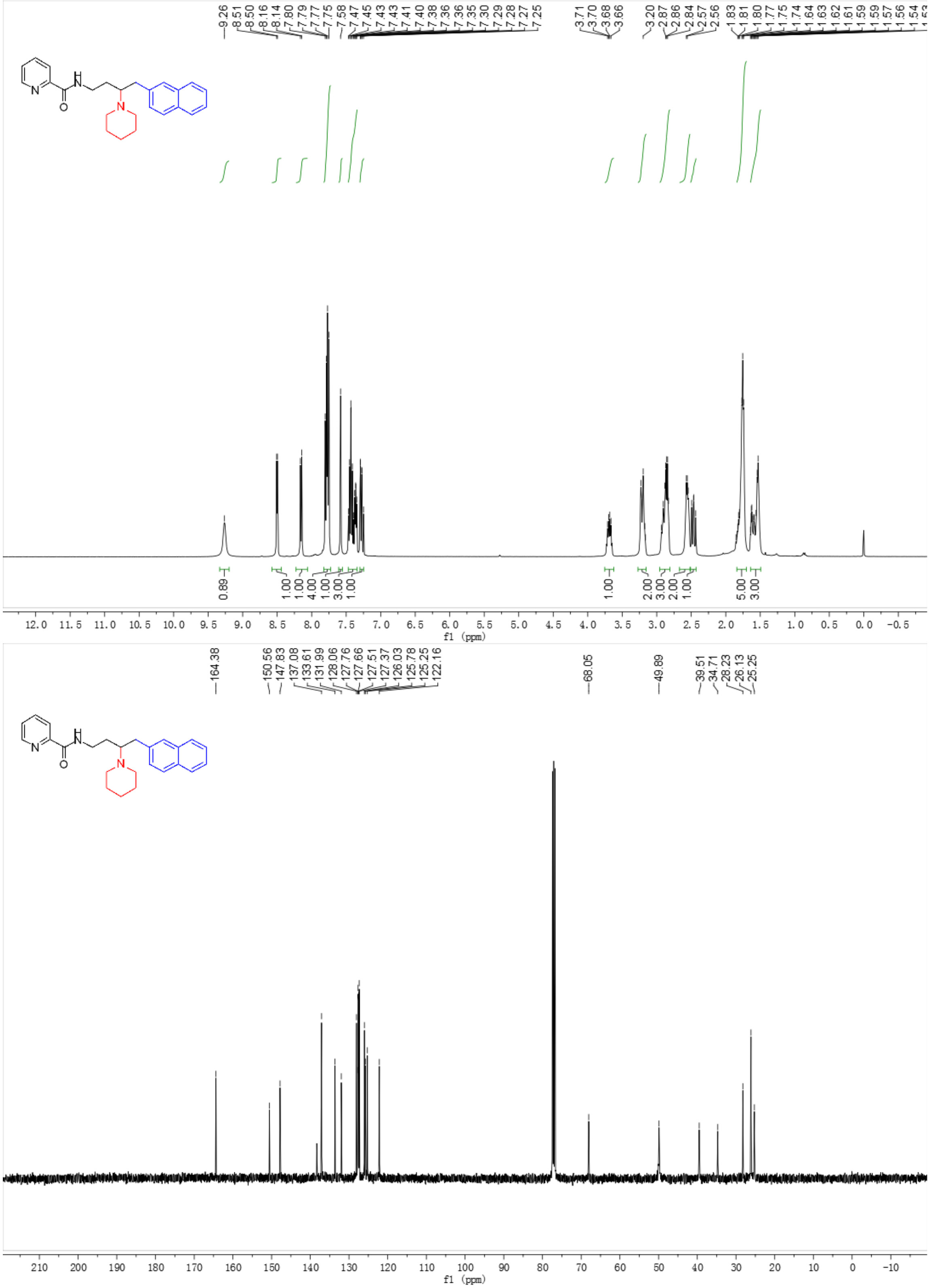

Supplementary Figure 26. ^{1}H NMR and ^{13}C NMR spectra of **2f**.

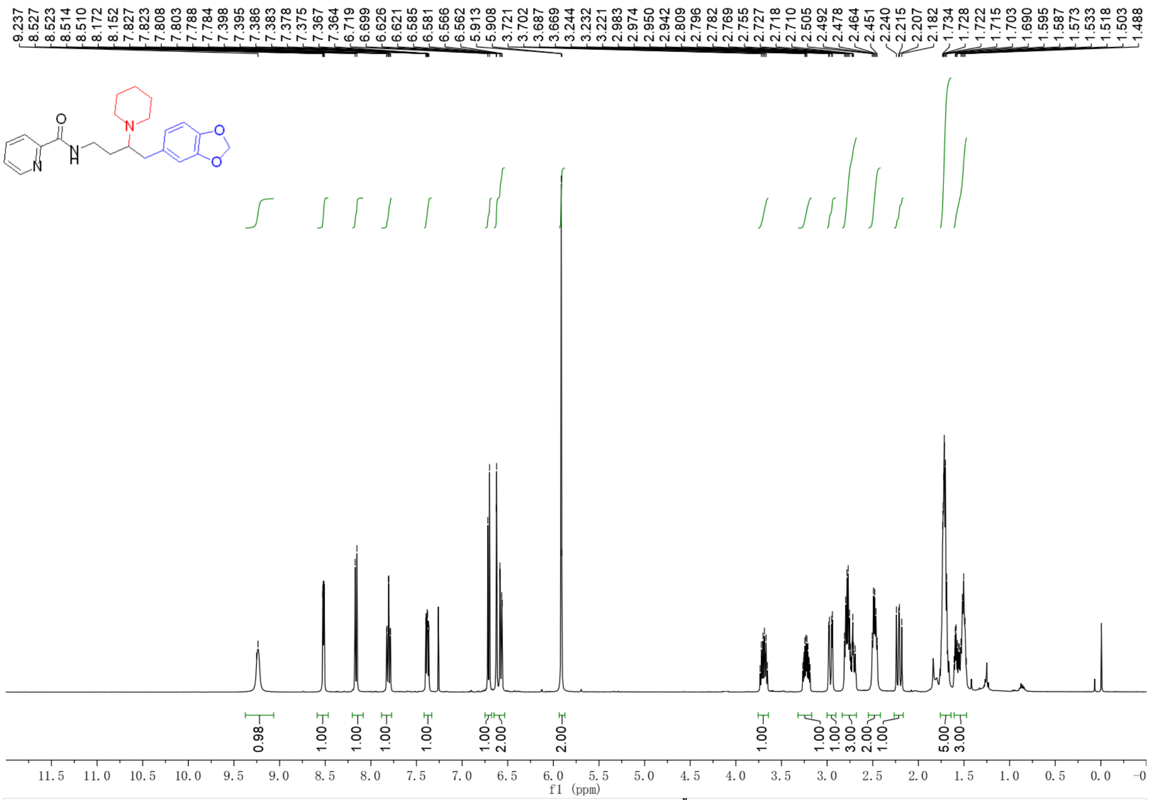

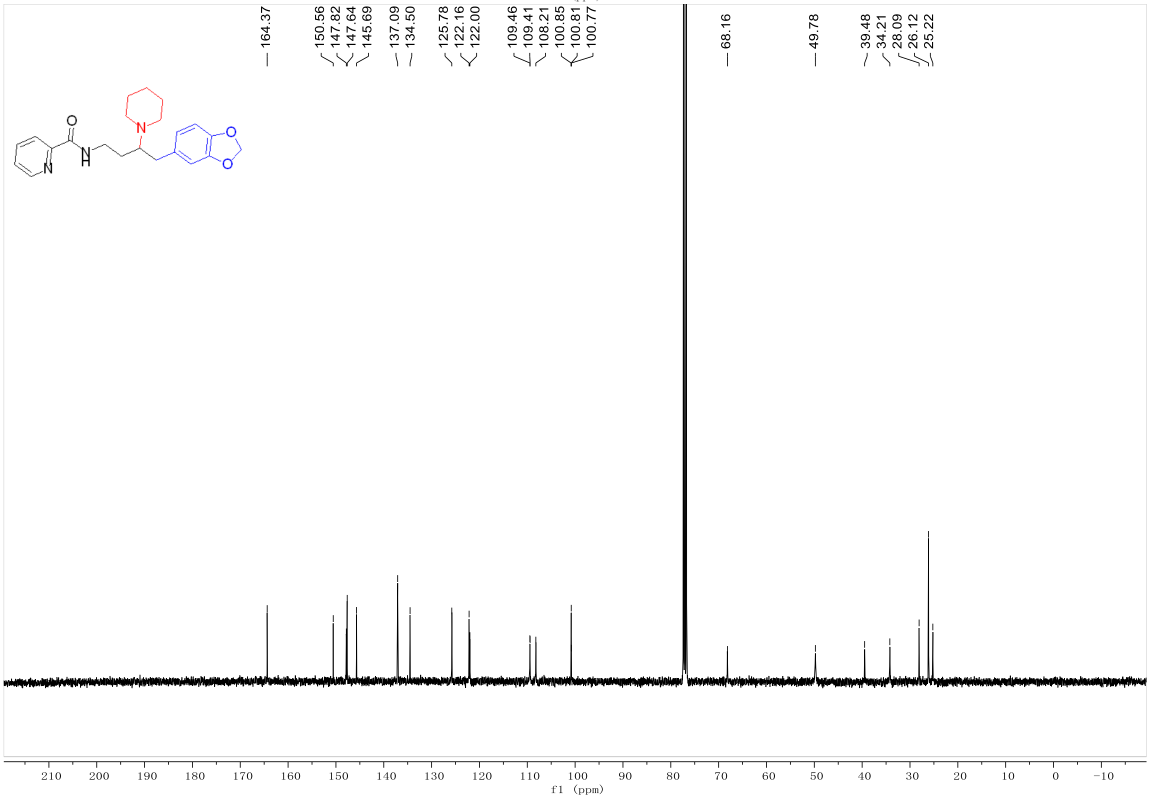

Supplementary Figure 27. ^{1}H NMR and ^{13}C NMR spectra of **2g**.

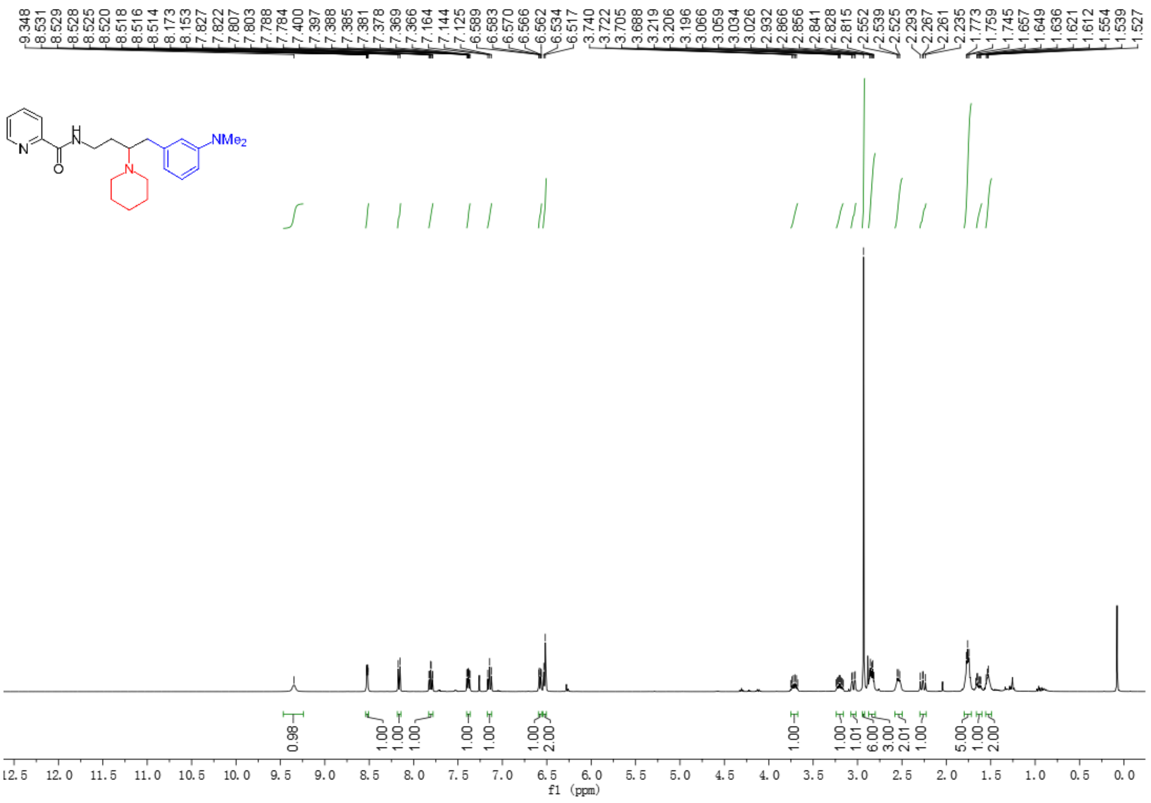

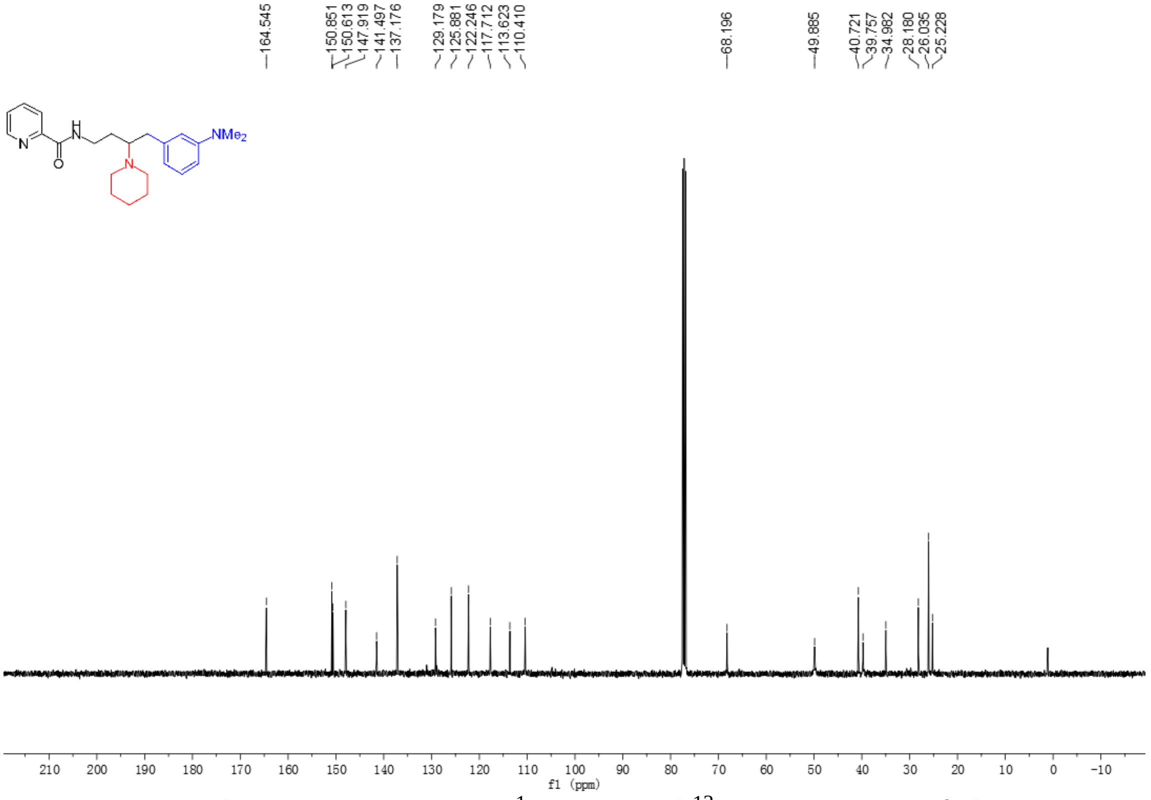

Supplementary Figure 28. ^{1}H NMR and ^{13}C NMR spectra of **2h**.

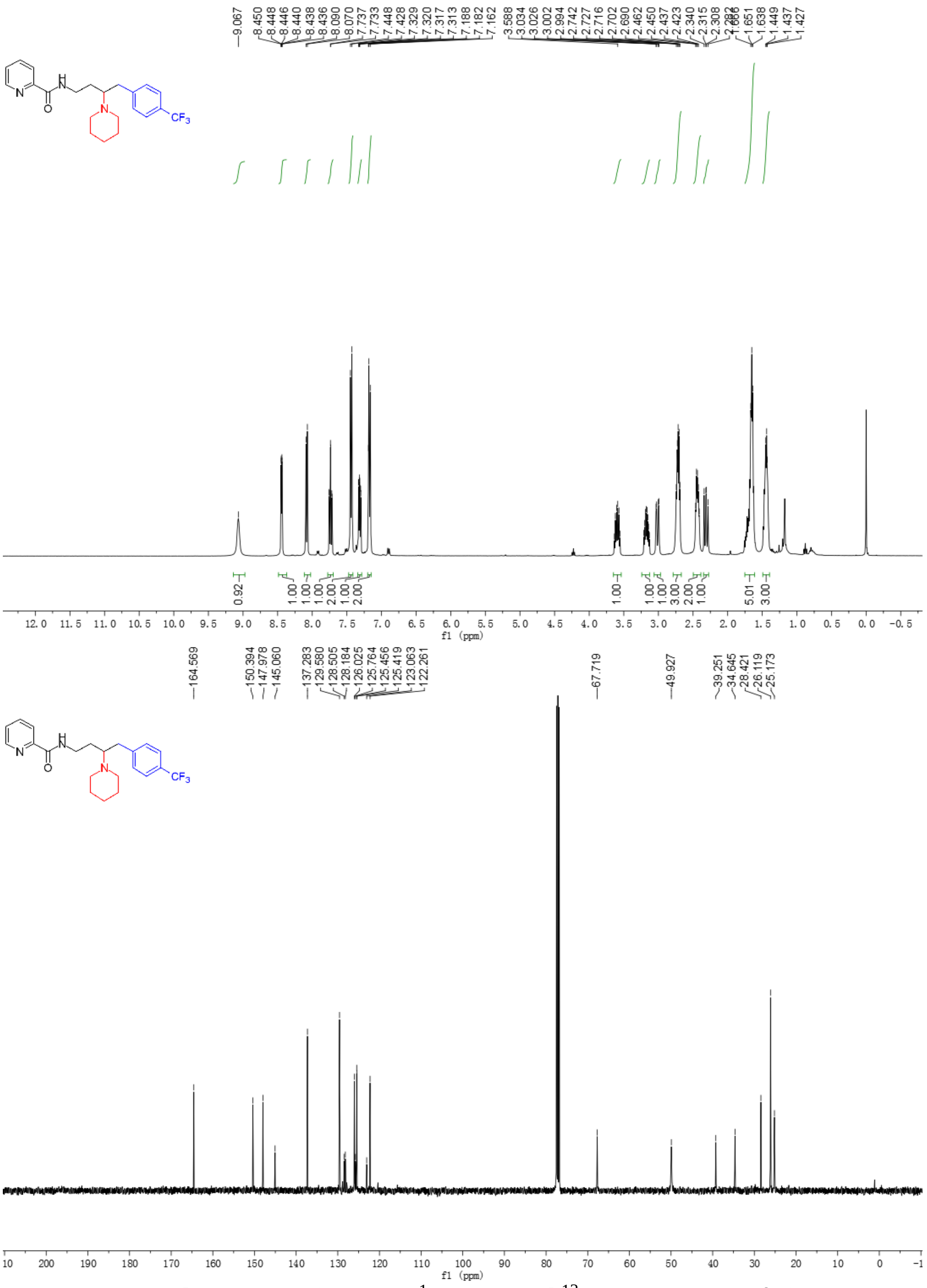

Supplementary Figure 29. ^{1}H NMR and ^{13}C NMR spectra of **2i**.

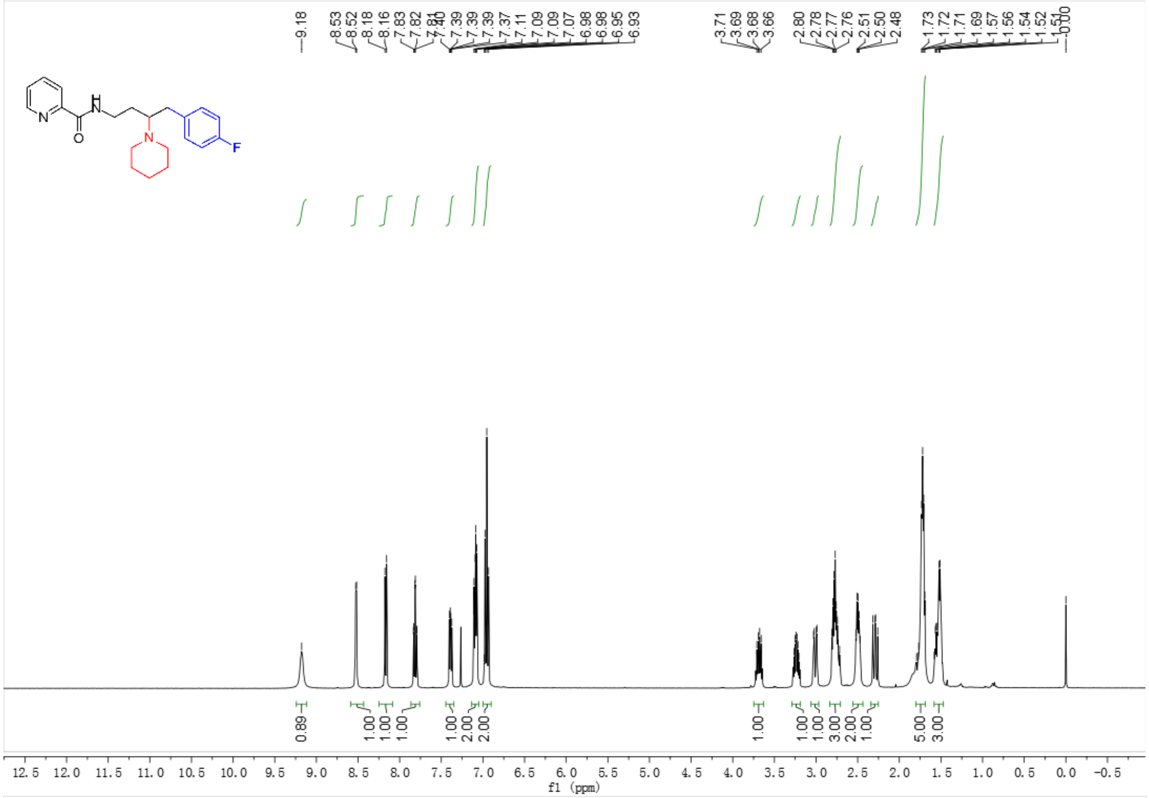

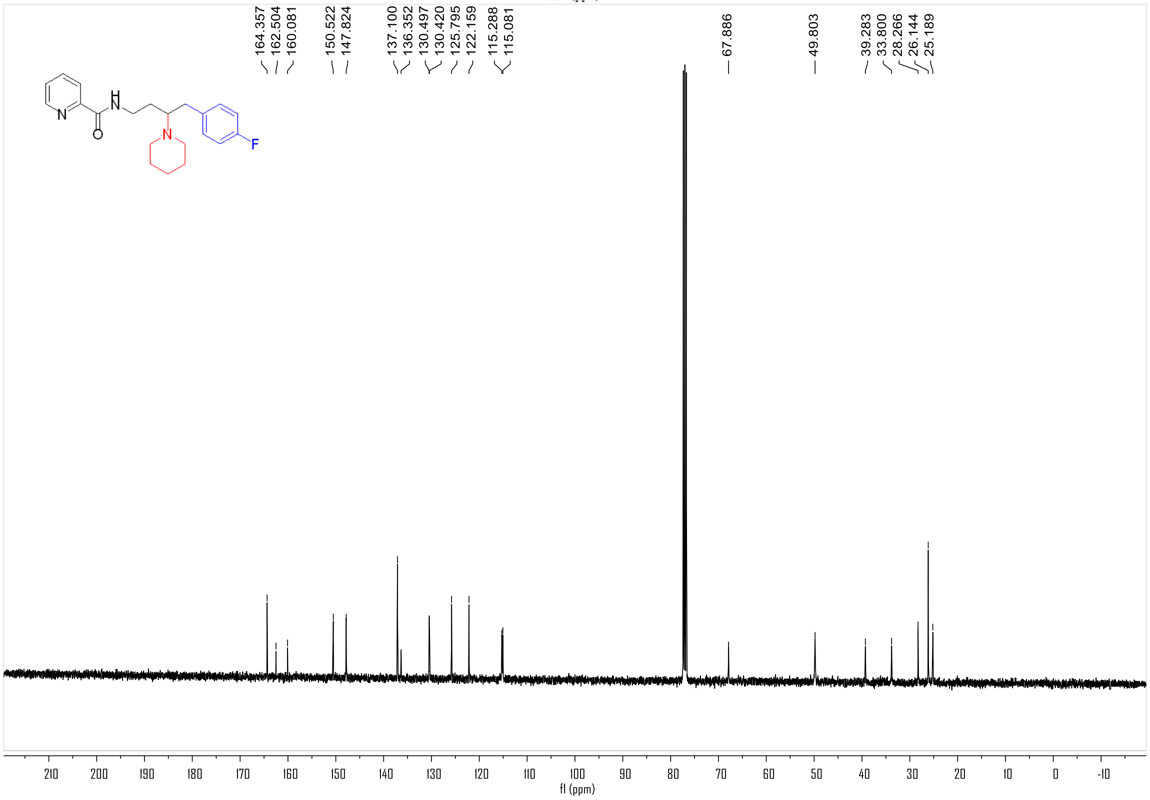

Supplementary Figure 30. ^{1}H NMR and ^{13}C NMR spectra of **2j**.

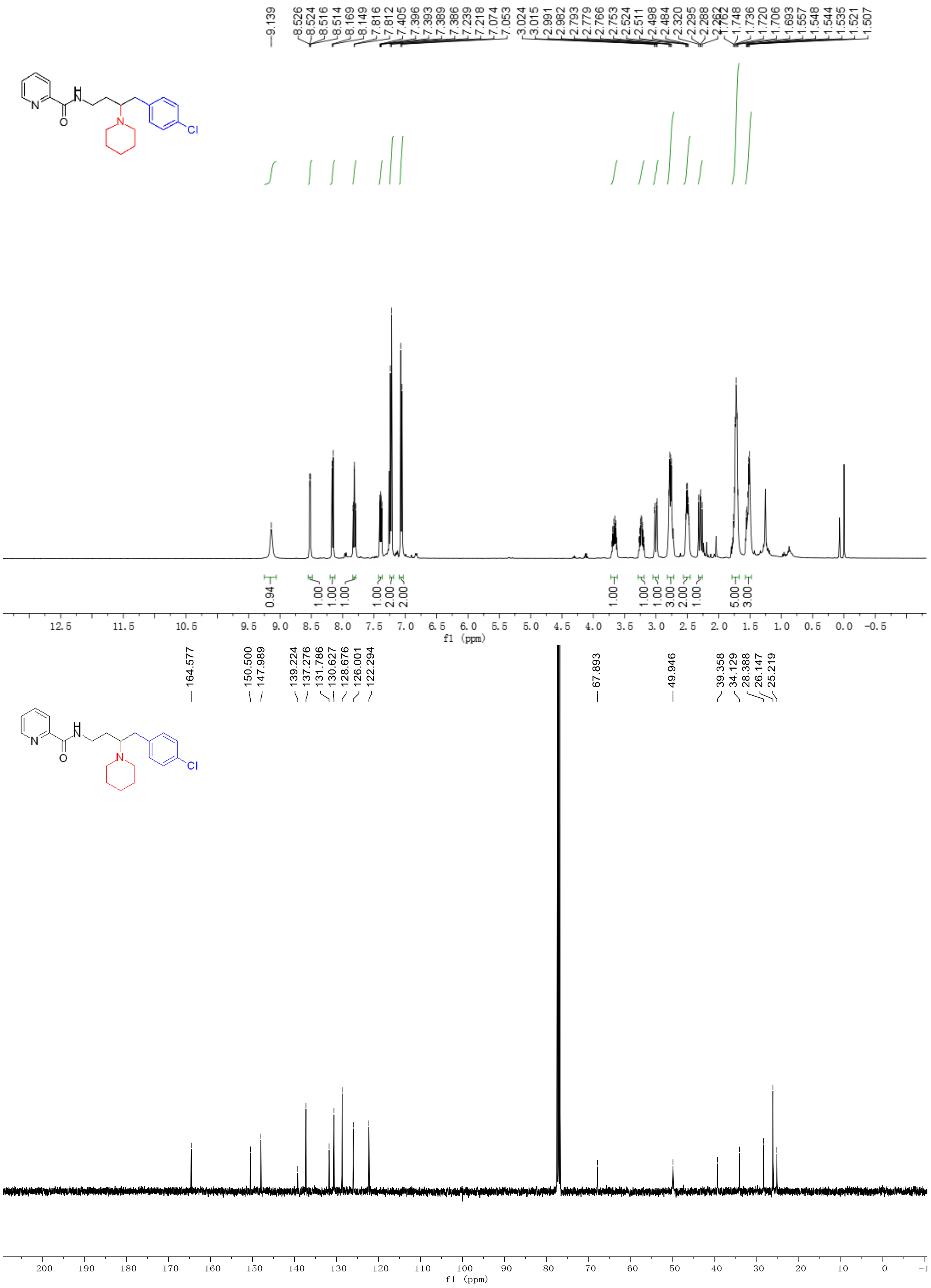

Supplementary Figure 31. ^{1}H NMR and ^{13}C NMR spectra of **2k**.

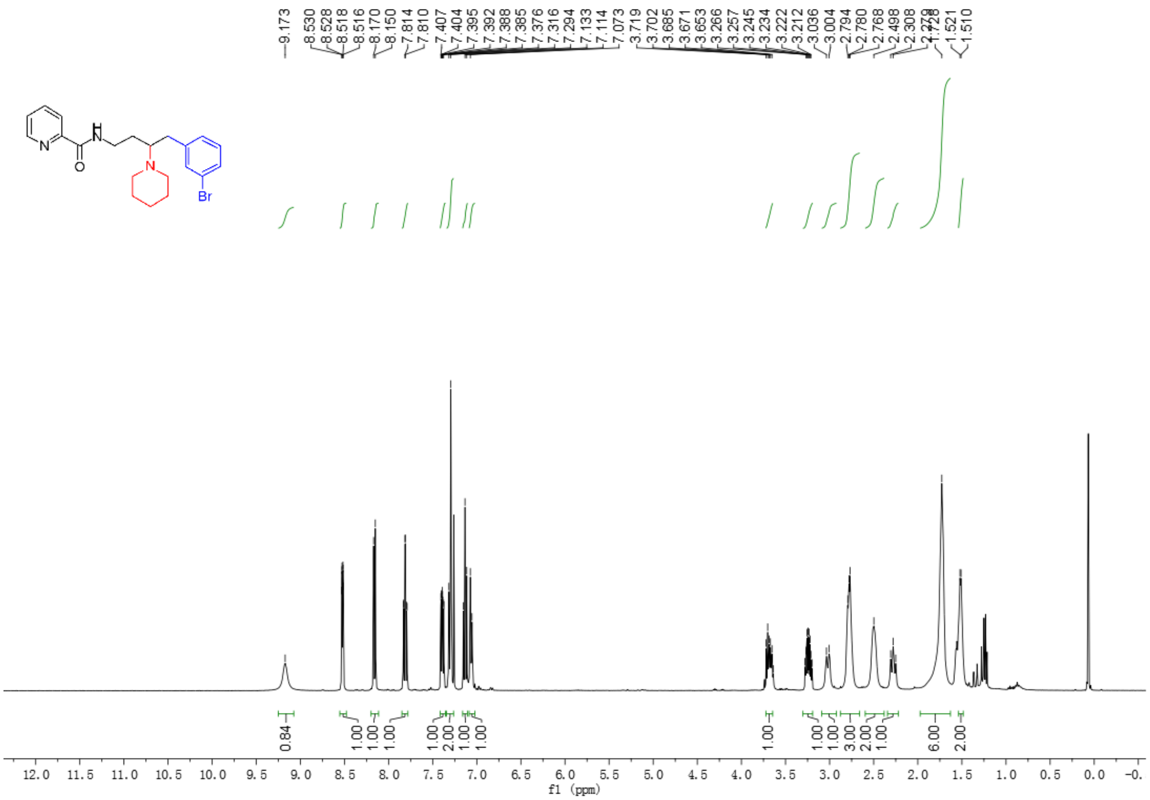

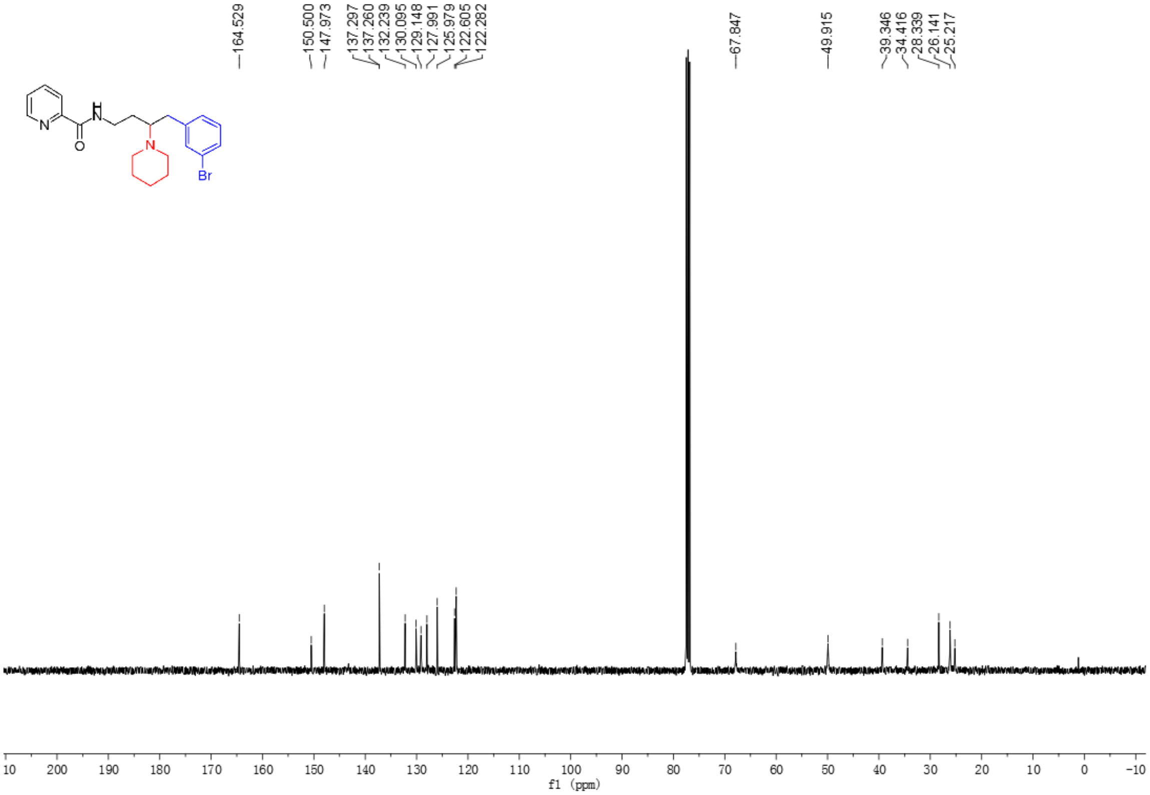

Supplementary Figure 32. ^{1}H NMR and ^{13}C NMR spectra of **2l**.

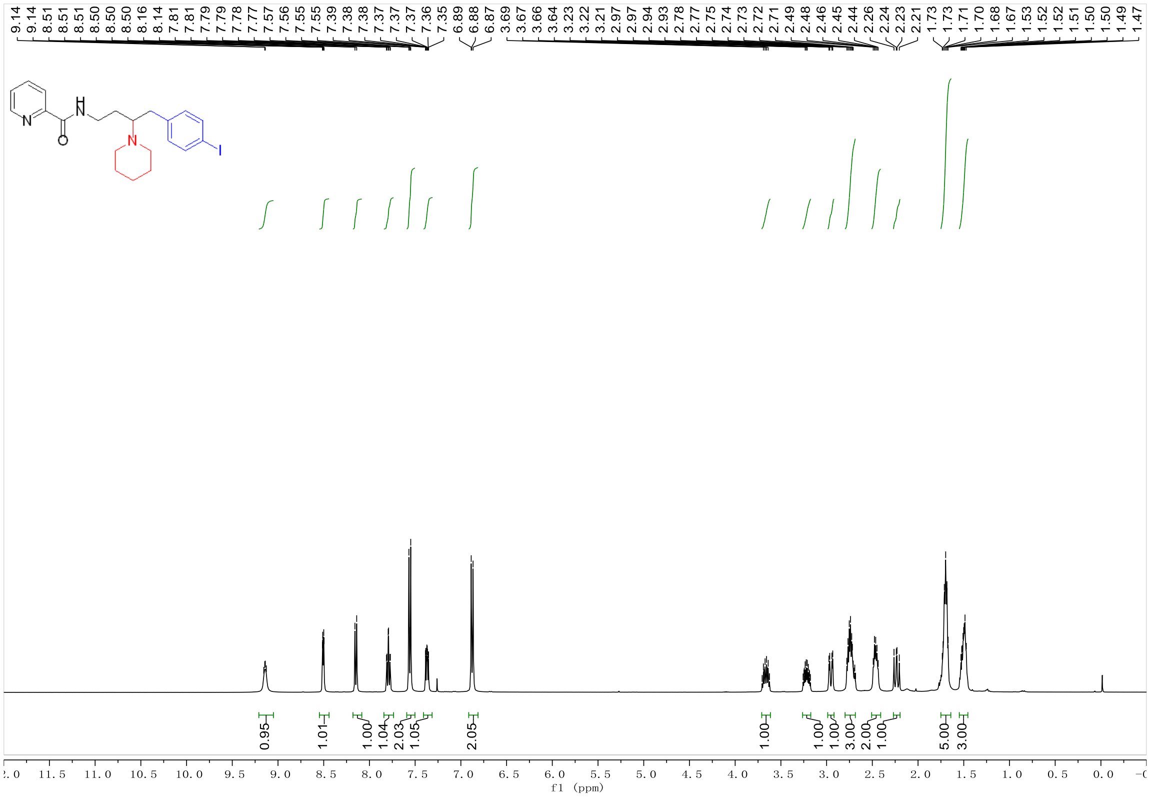

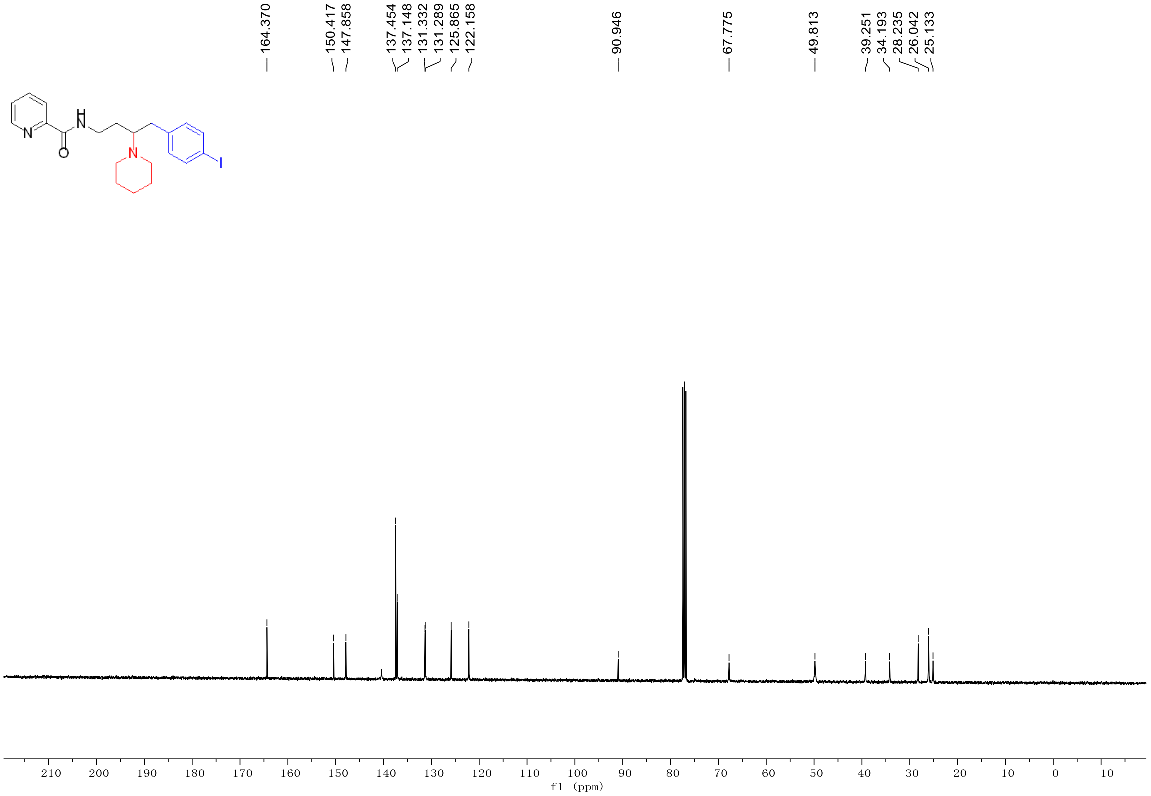

Supplementary Figure 33. ^{1}H NMR and ^{13}C NMR spectra of **2m**.

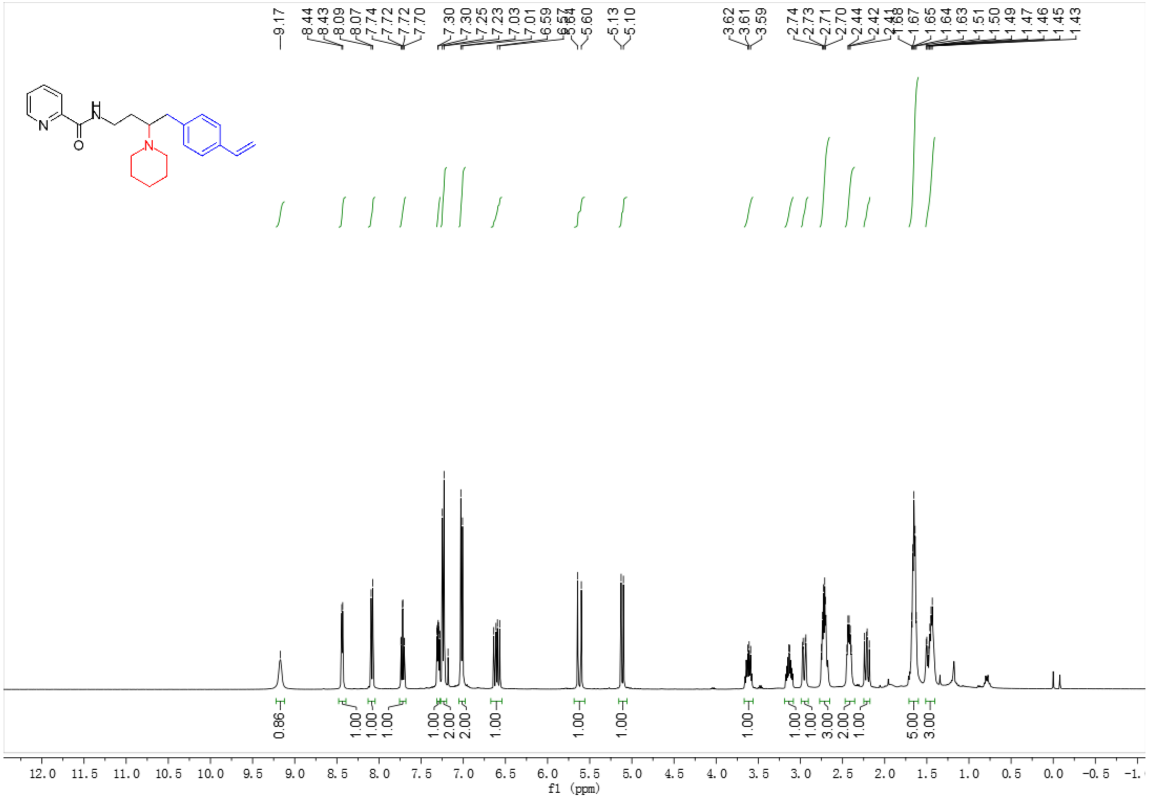

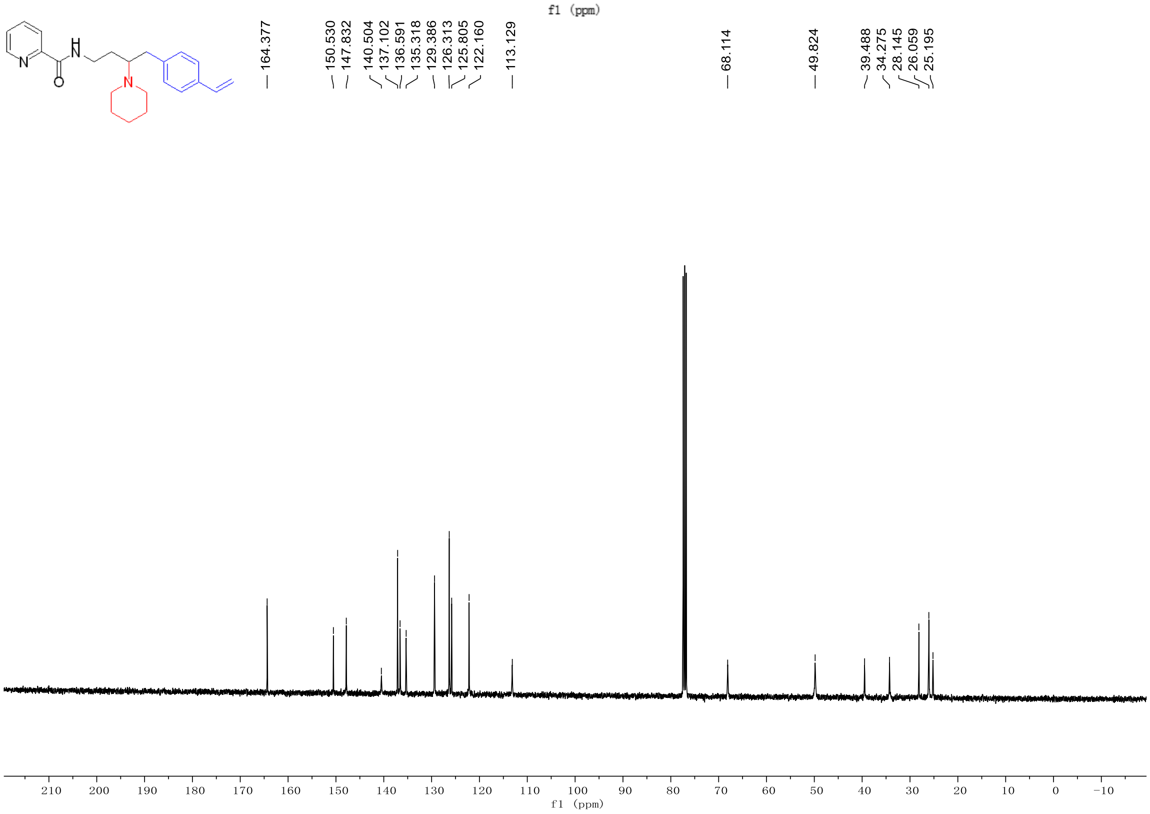

Supplementary Figure 34. ^{1}H NMR and ^{13}C NMR spectra of **2n**.

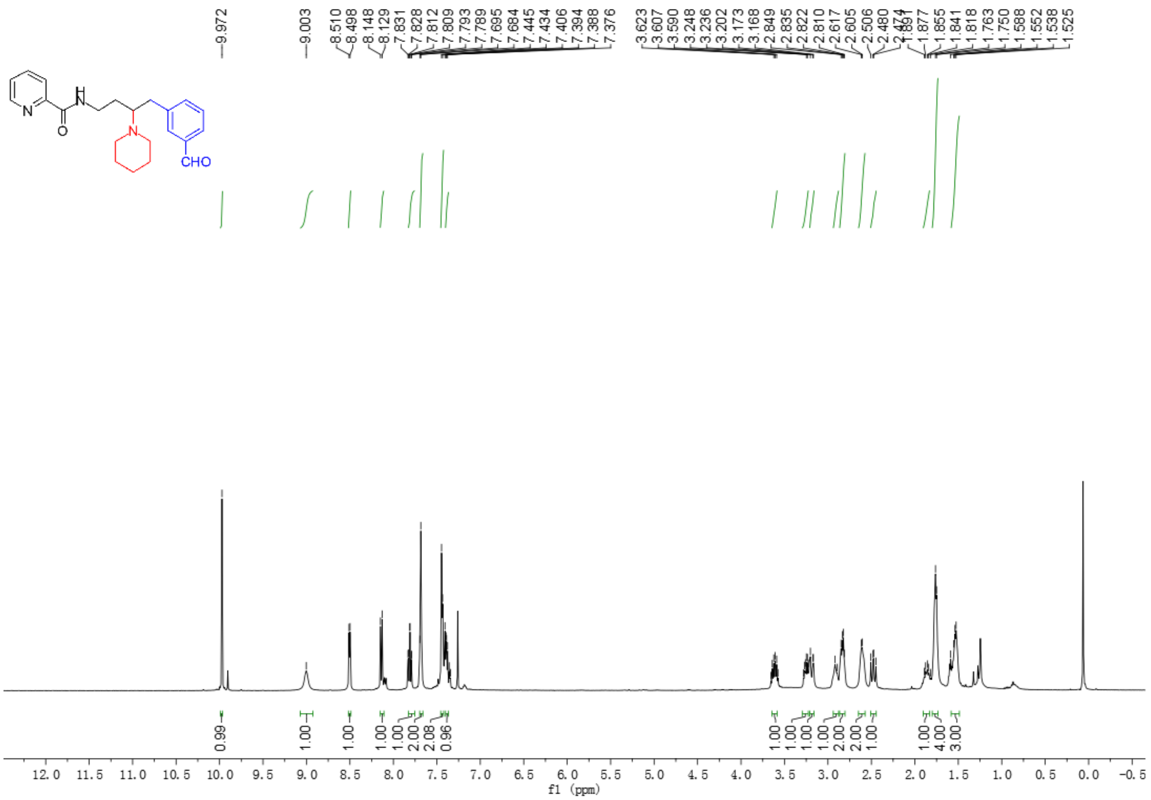

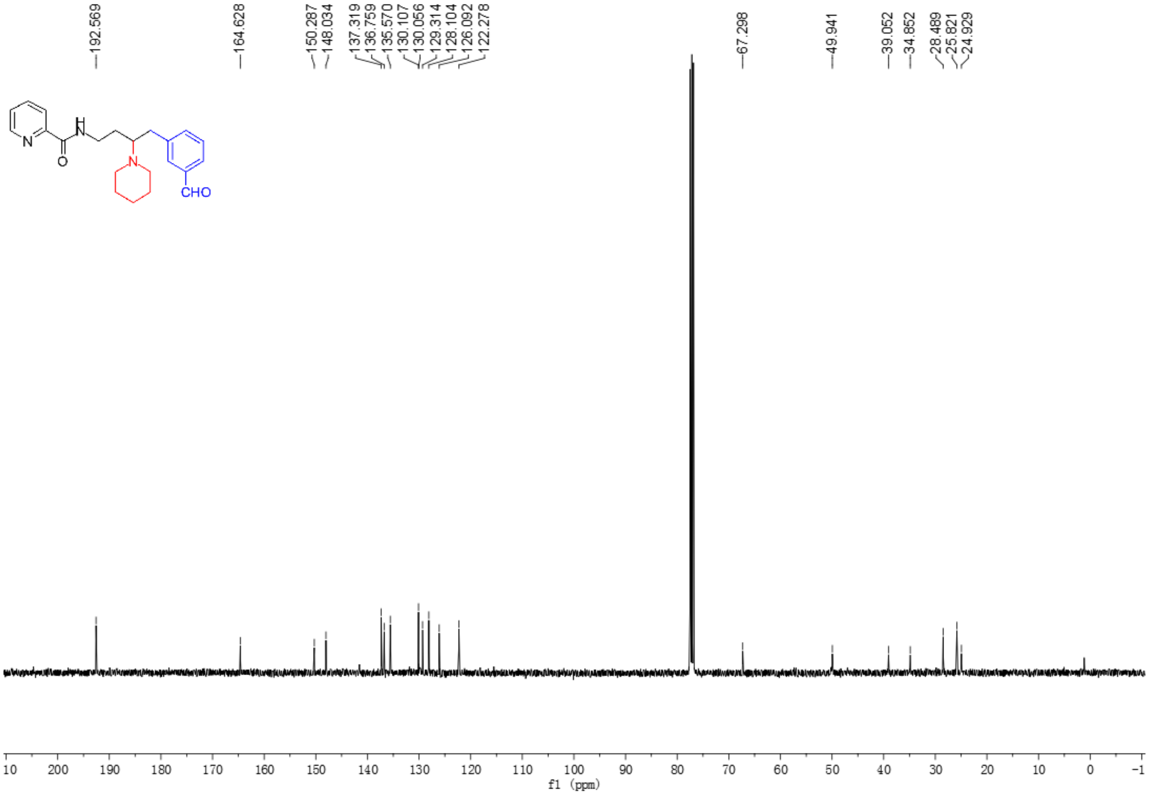

Supplementary Figure 35. ^{1}H NMR and ^{13}C NMR spectra of **2o**.

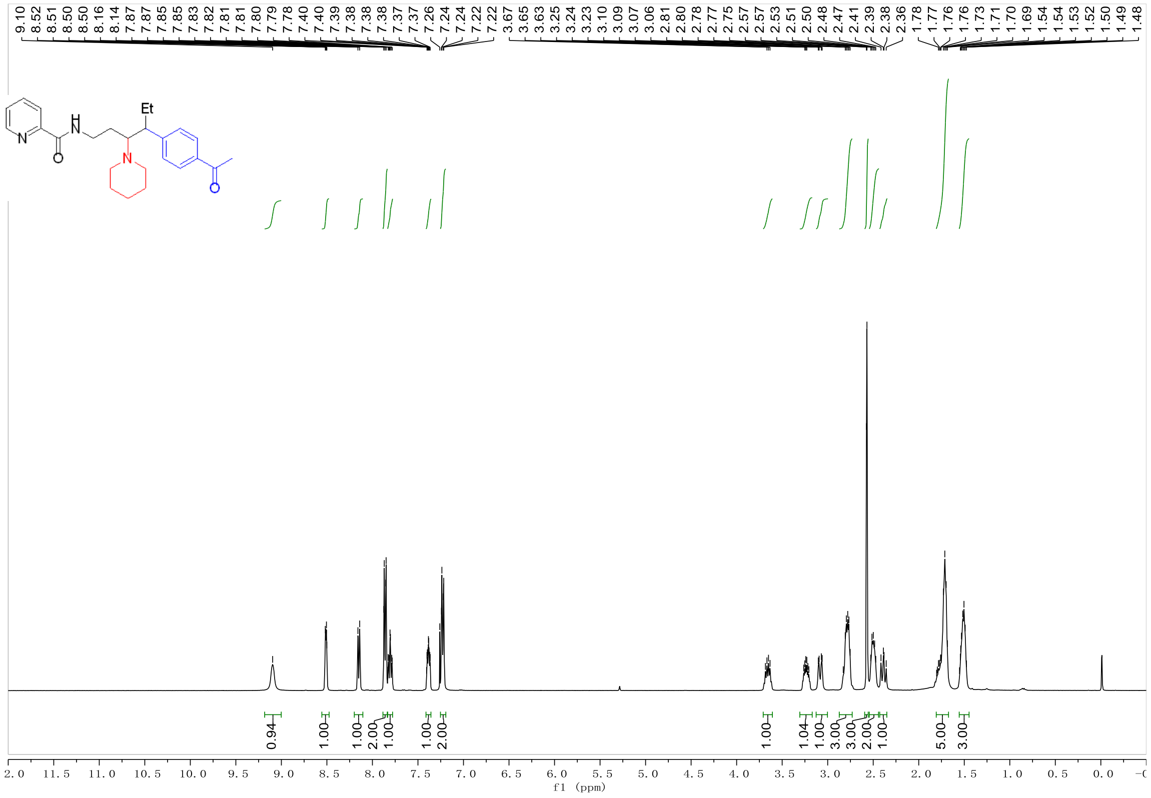

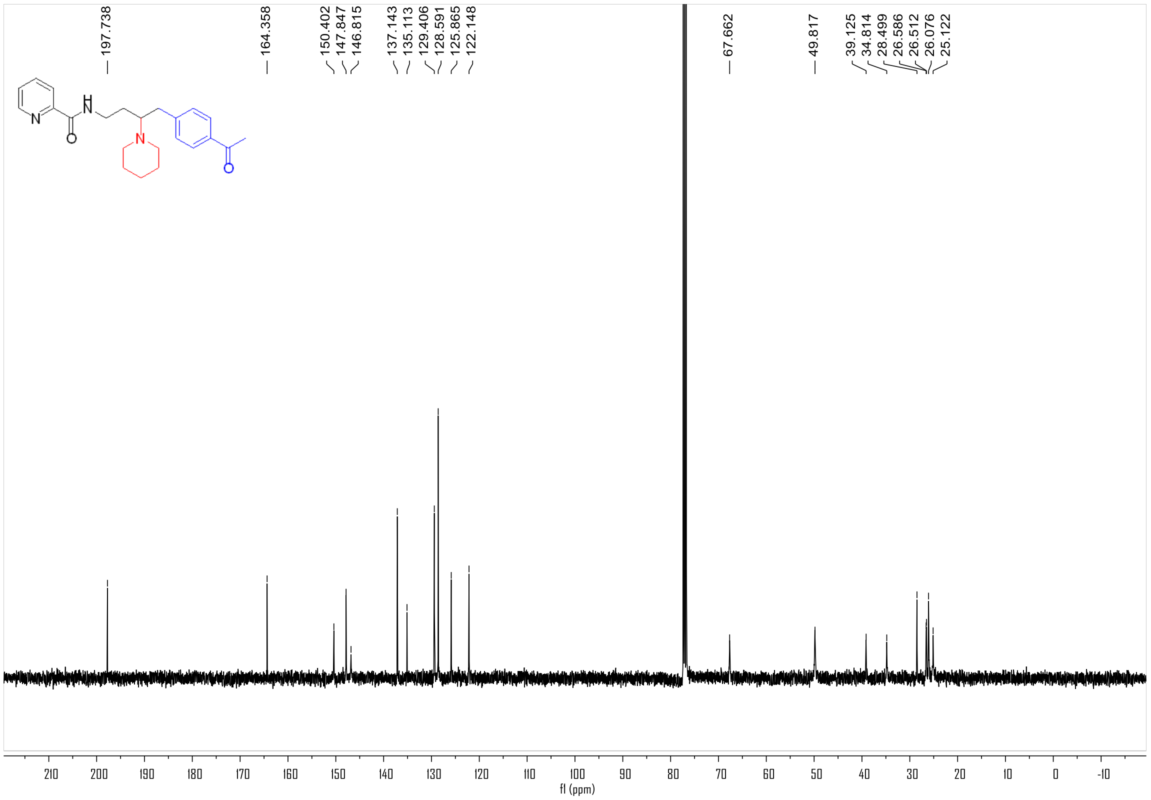

Supplementary Figure 36. ^{1}H NMR and ^{13}C NMR spectra of **2p**.

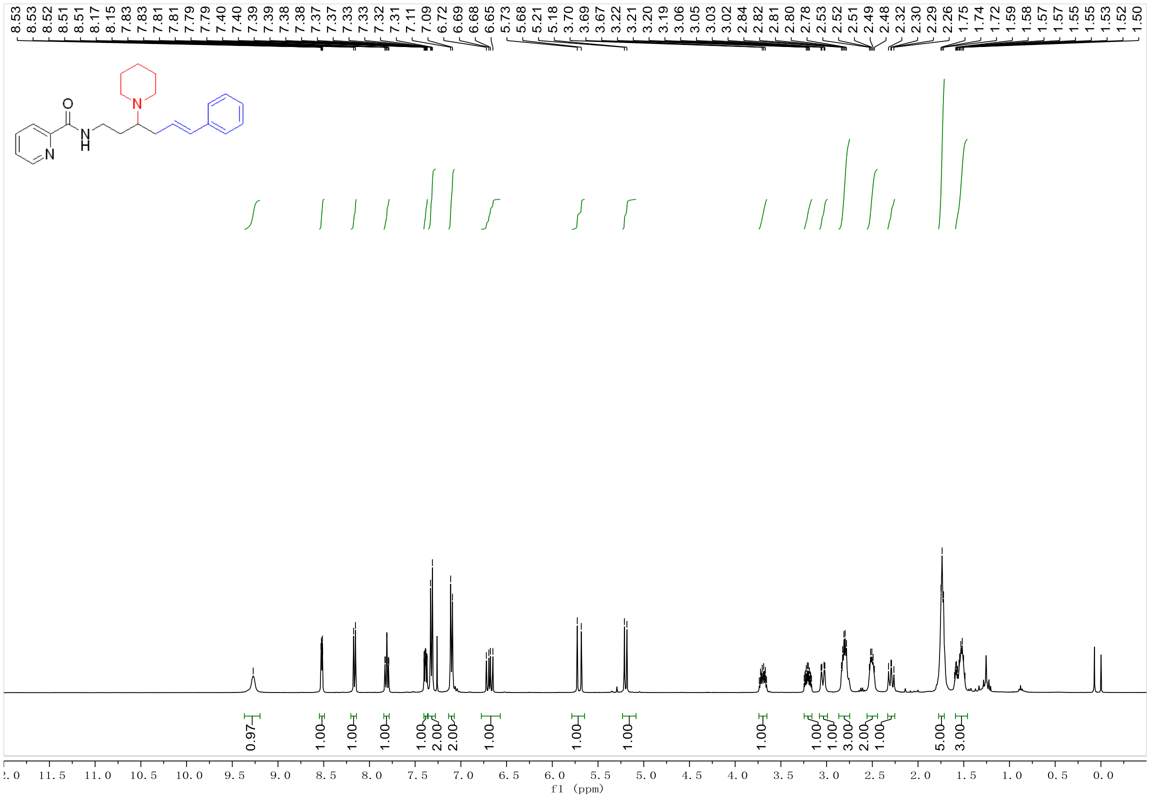

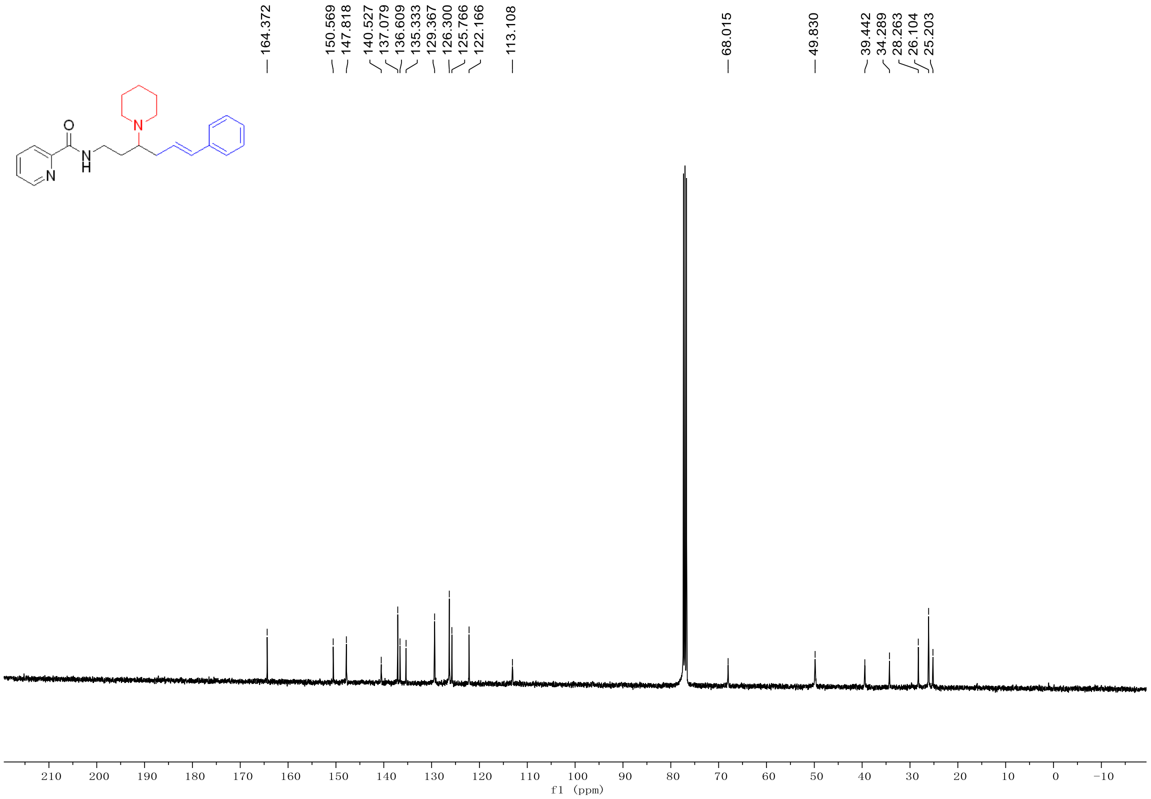

Supplementary Figure 37. ^{1}H NMR and ^{13}C NMR spectra of **2q**.

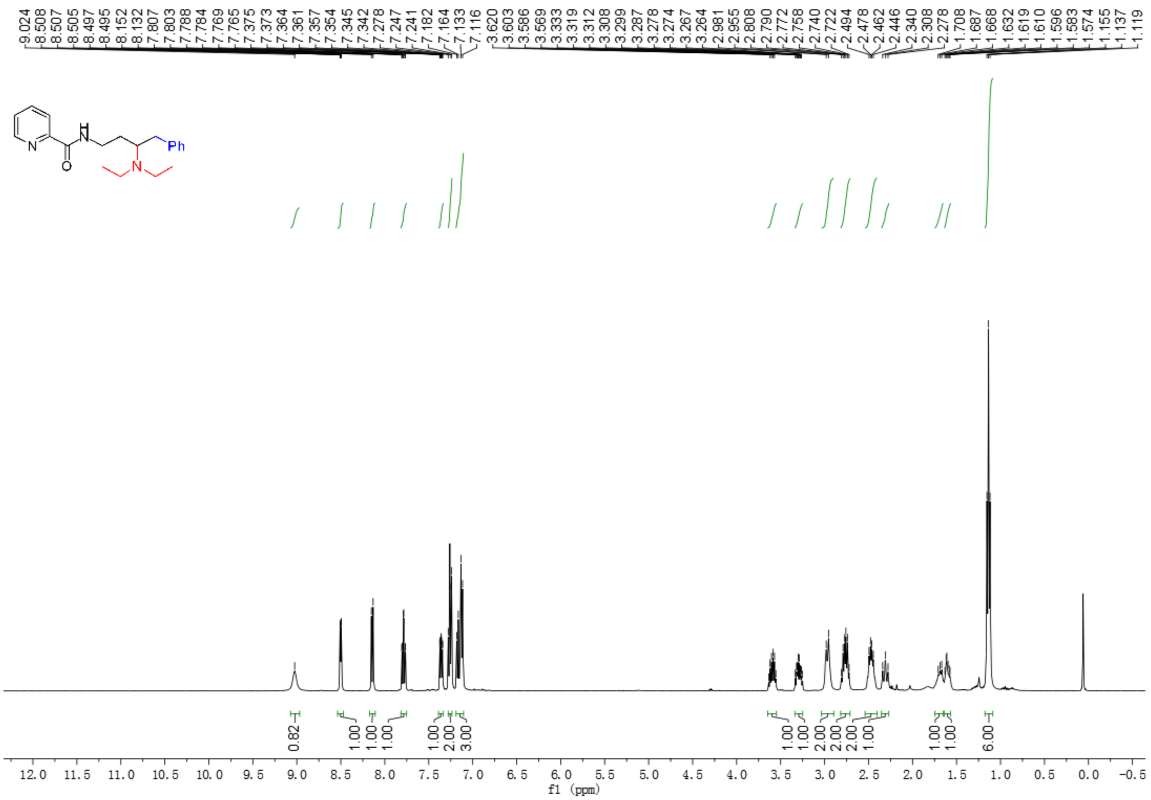

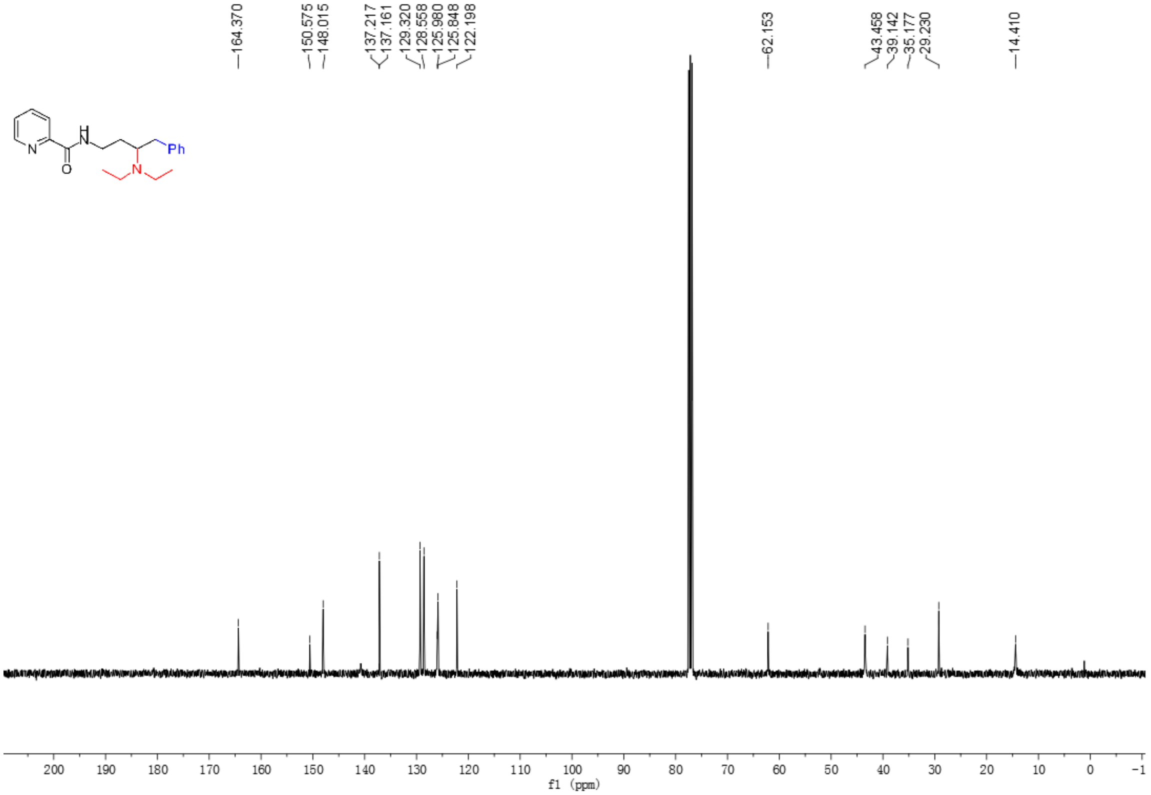

Supplementary Figure 38. ^1H NMR and ^{13}C NMR spectra of **3a**.

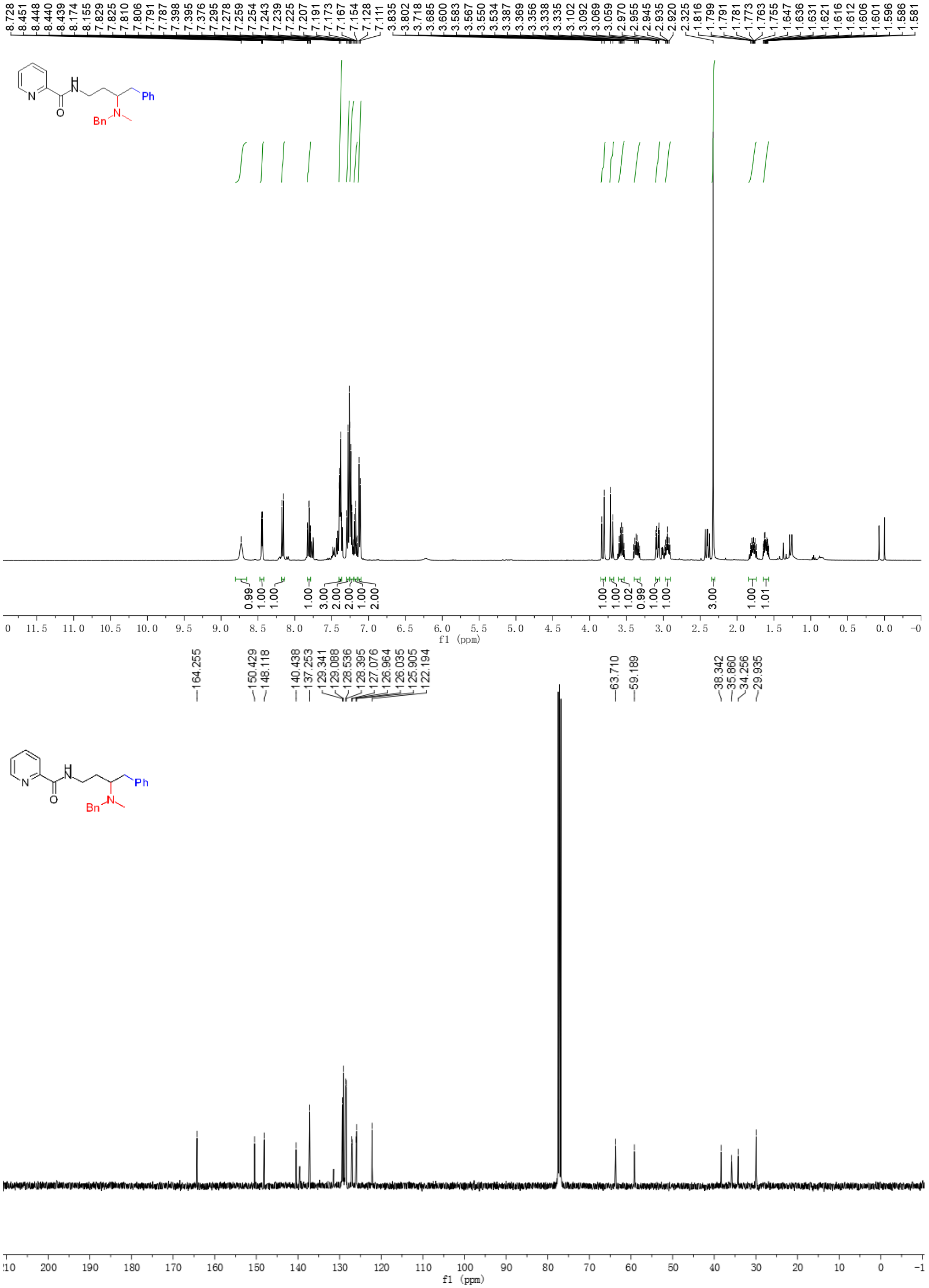

Supplementary Figure 39. ^{1}H NMR and ^{13}C NMR spectra of **3b**.

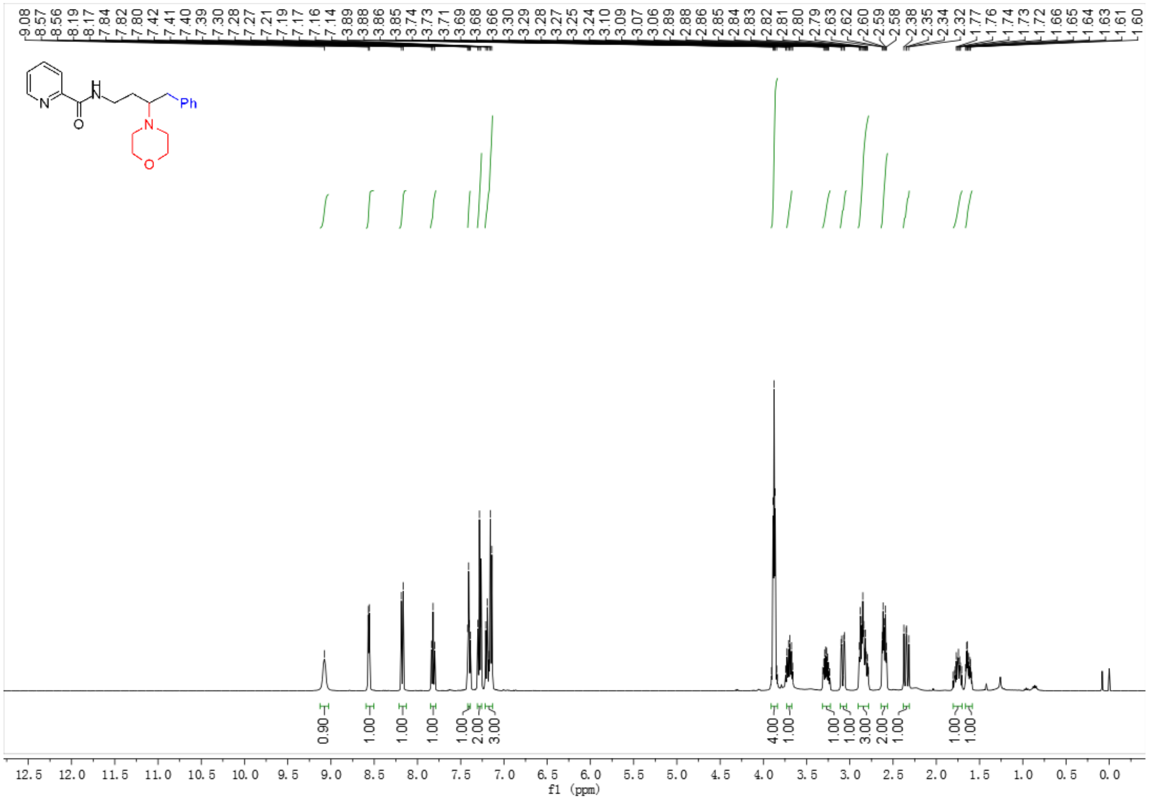

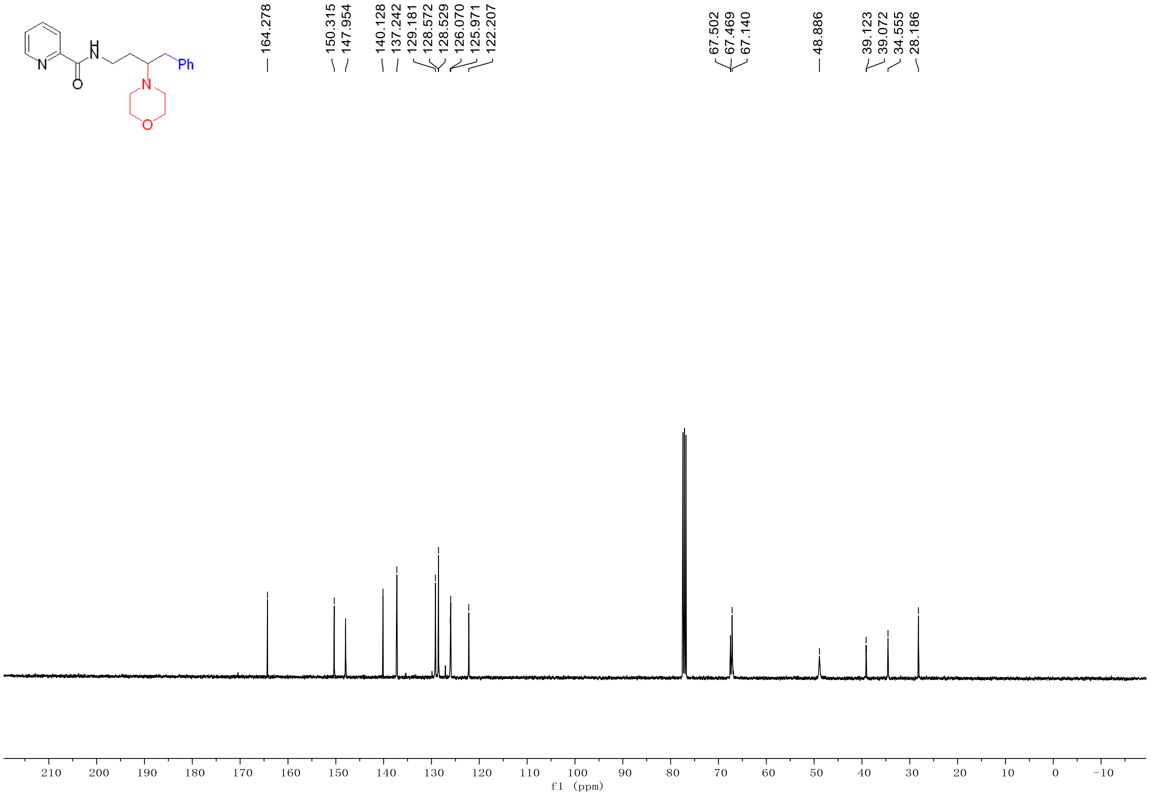

Supplementary Figure 40. ^{1}H NMR and ^{13}C NMR spectra of **3c**.

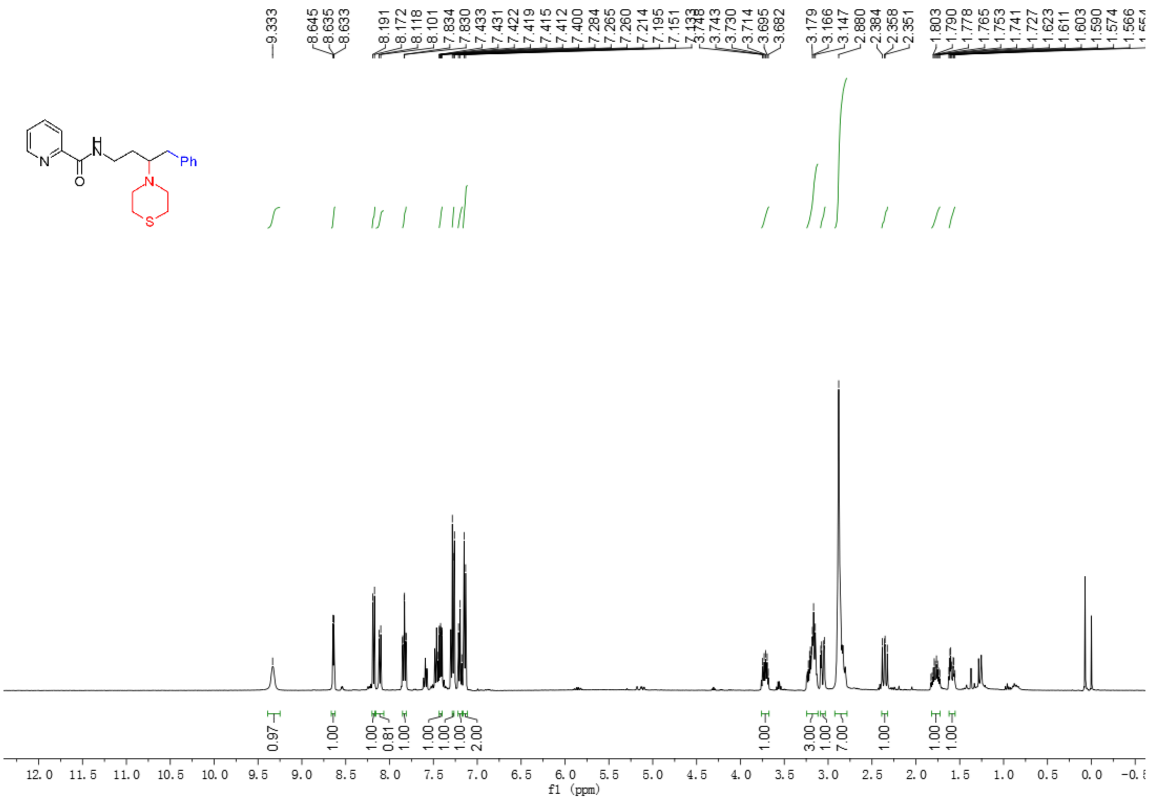

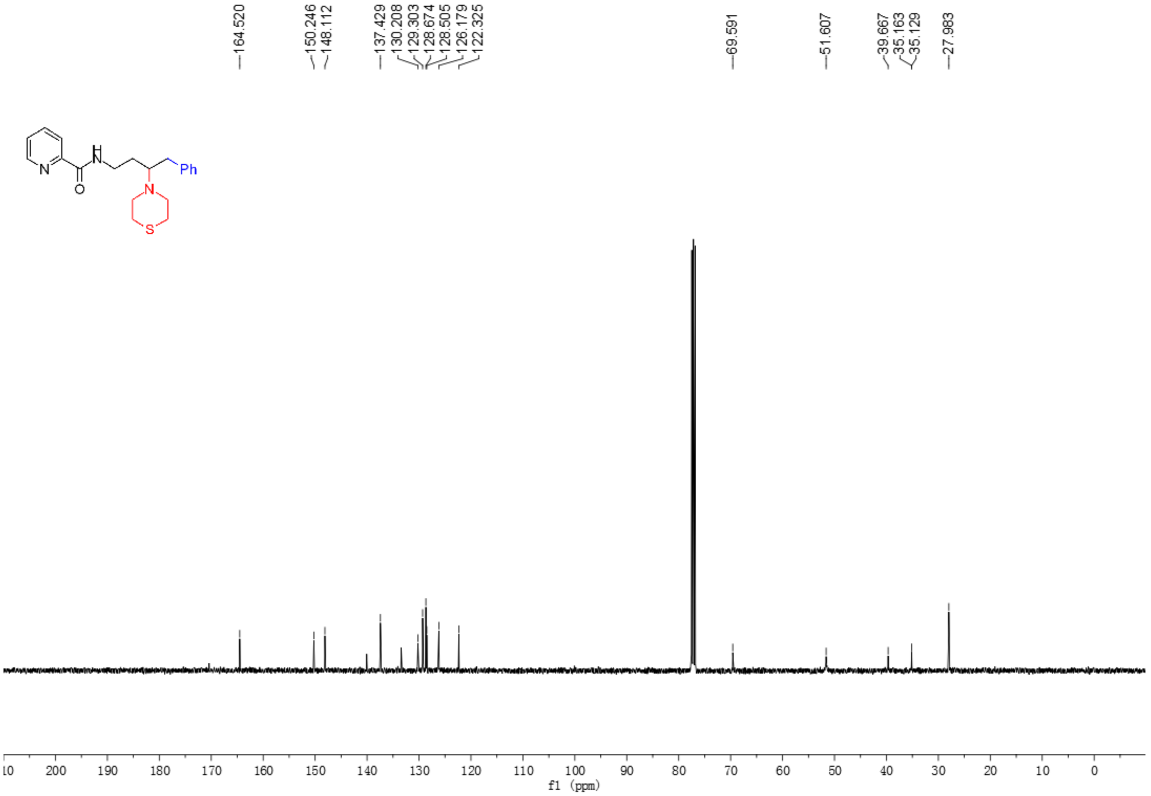

Supplementary Figure 41. ^{1}H NMR and ^{13}C NMR spectra of **3d**.

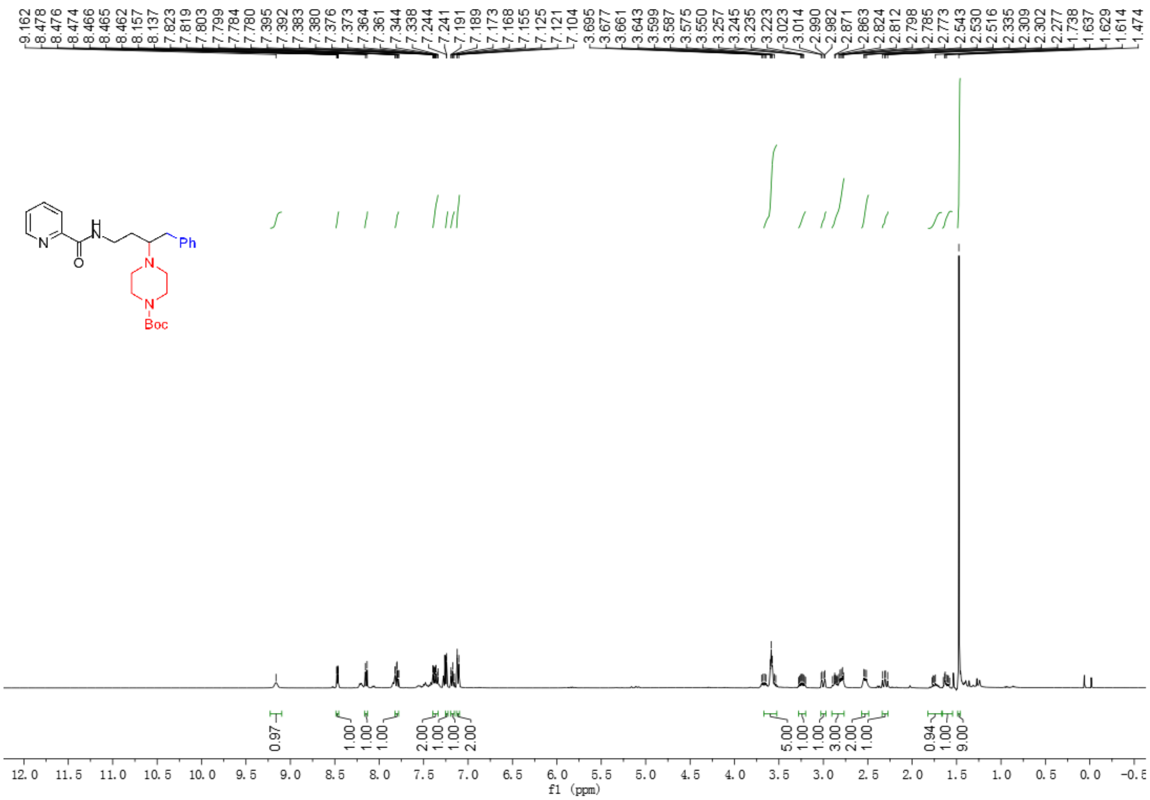

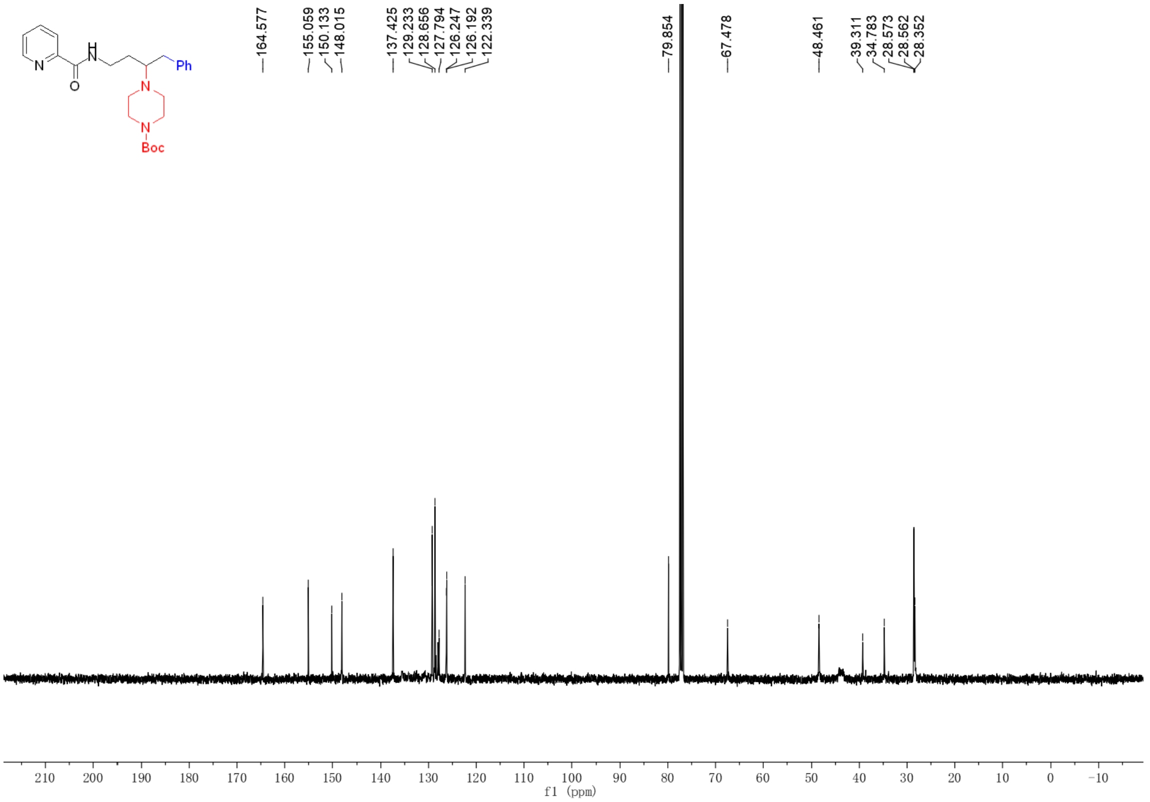

Supplementary Figure 42. ^{1}H NMR and ^{13}C NMR spectra of **3e**.

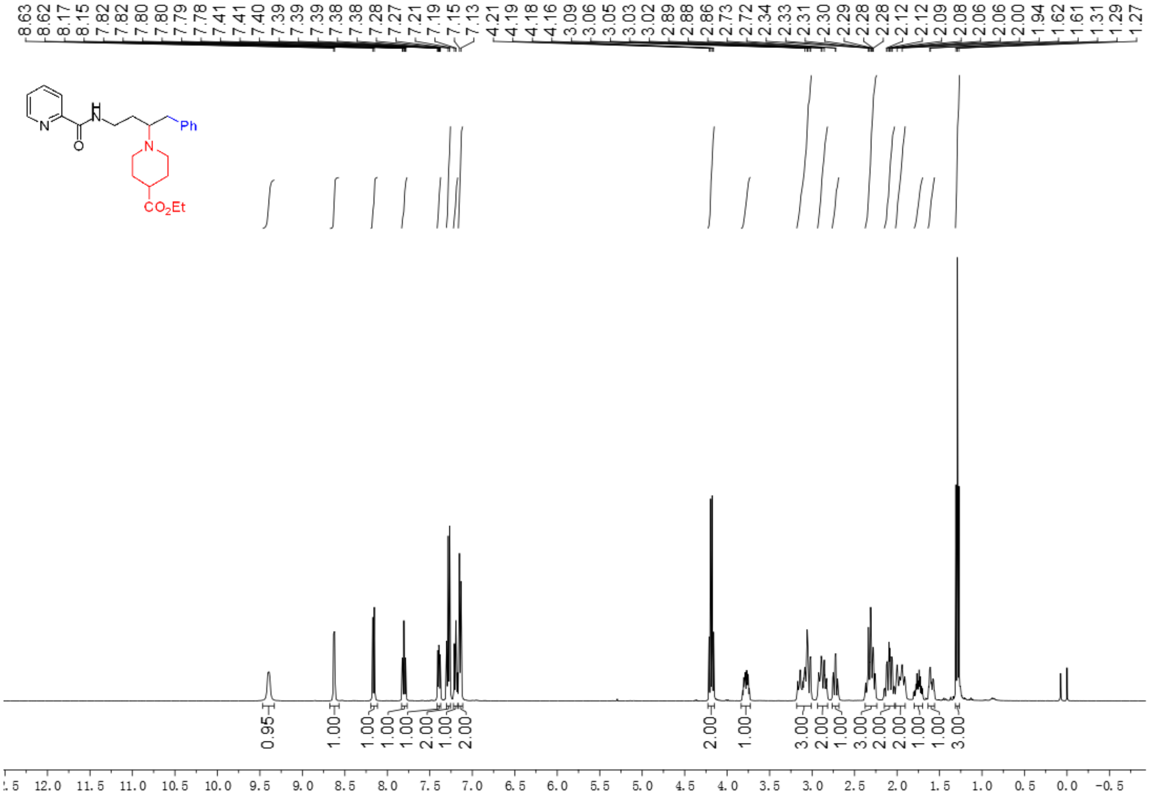

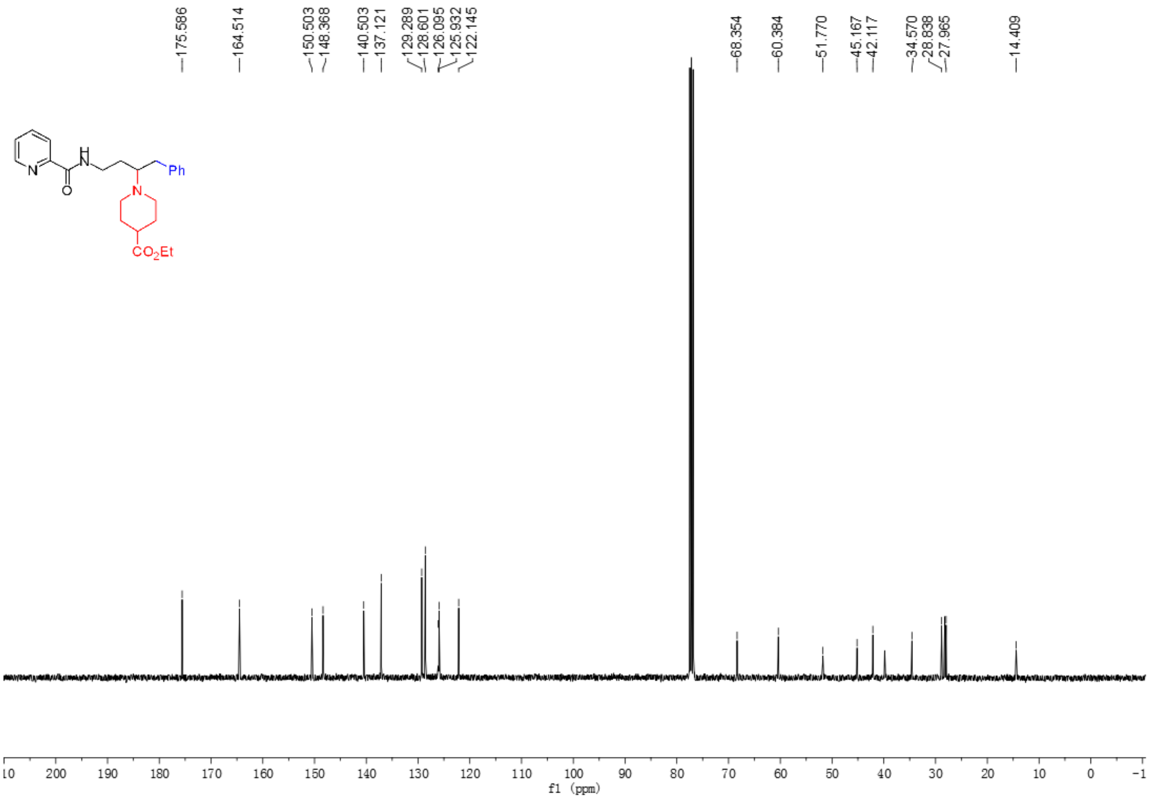

Supplementary Figure 43. ^{1}H NMR and ^{13}C NMR spectra of **3f**.

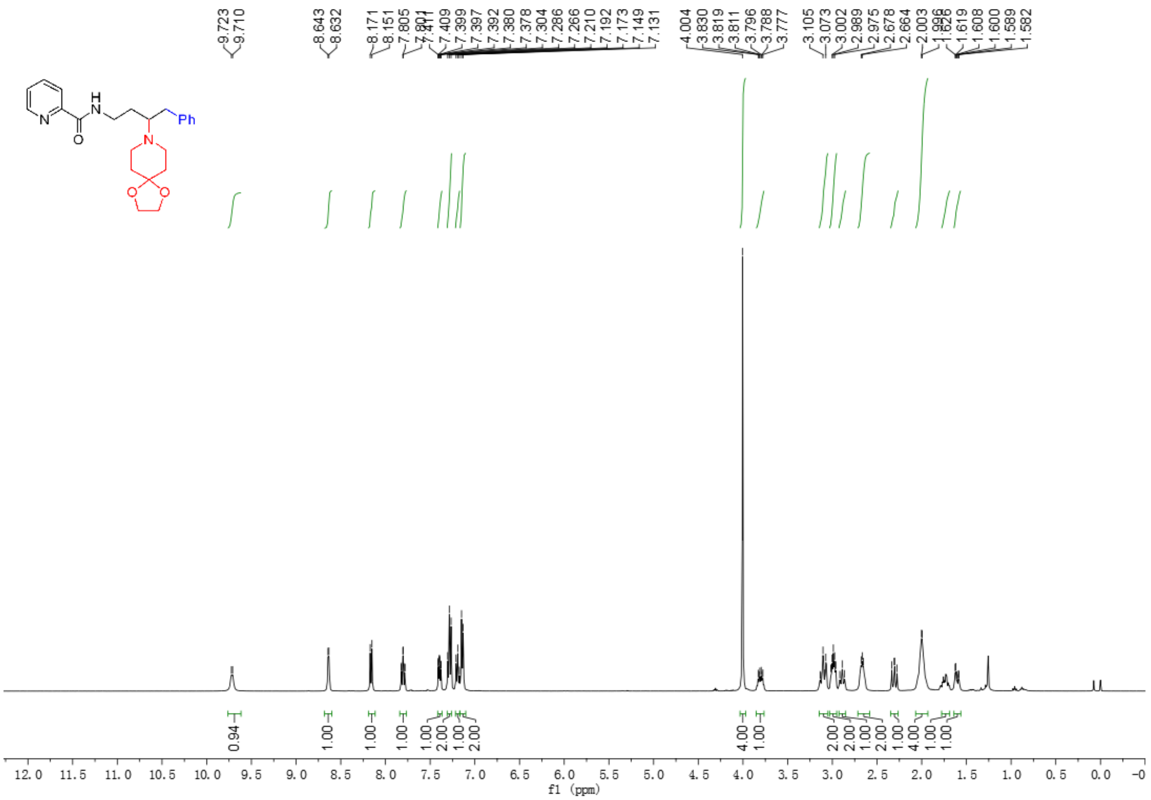

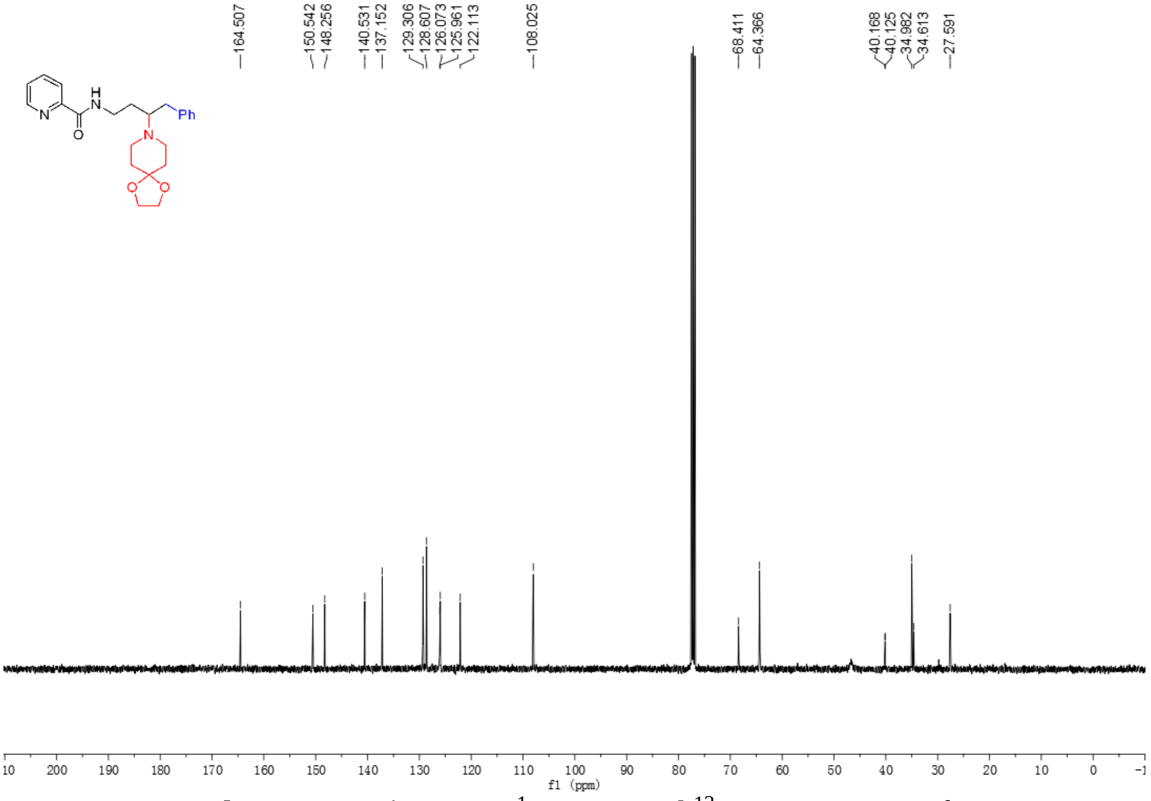

Supplementary Figure 44. ^{1}H NMR and ^{13}C NMR spectra of **3g**.

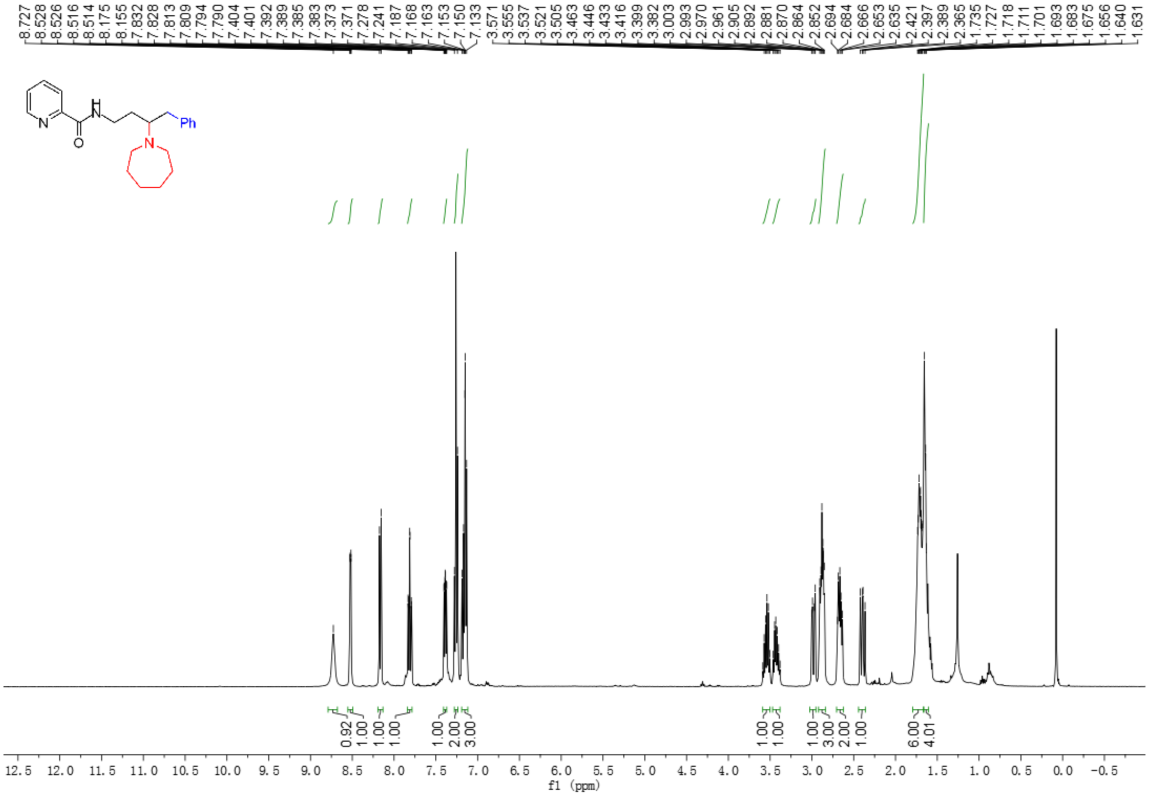

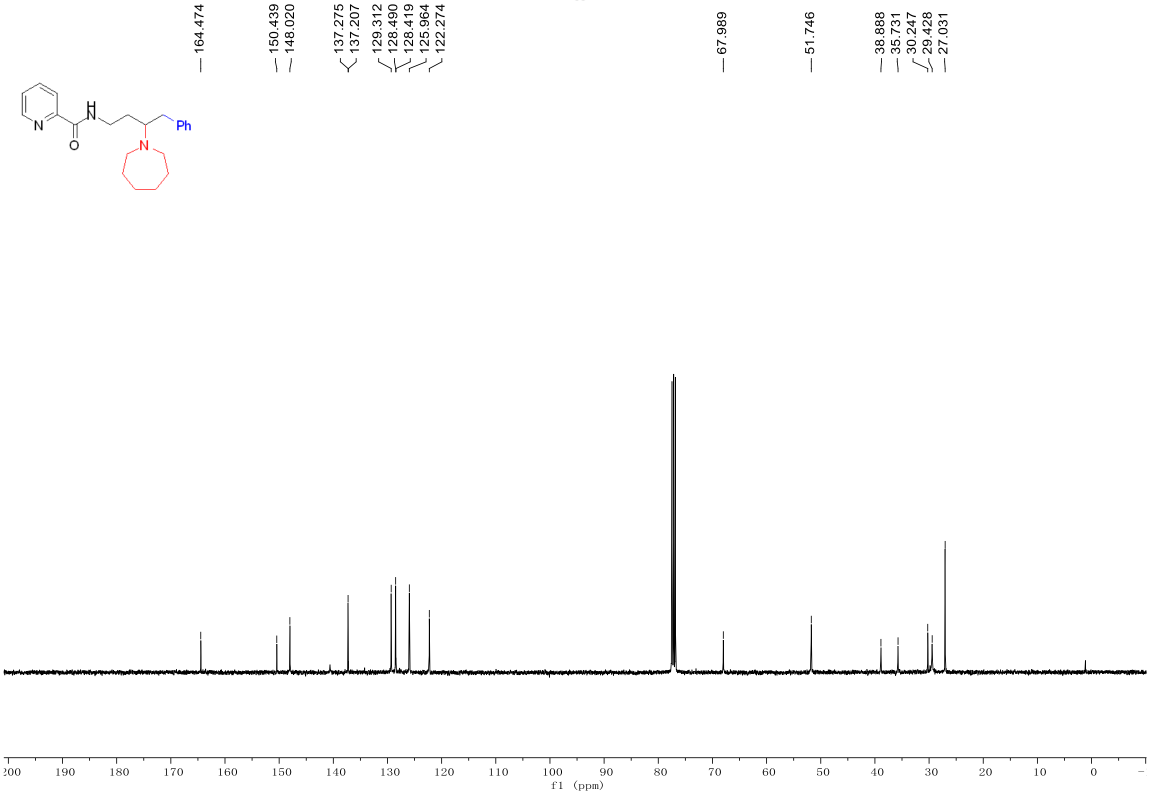

Supplementary Figure 45. ^{1}H NMR and ^{13}C NMR spectra of **3h**.

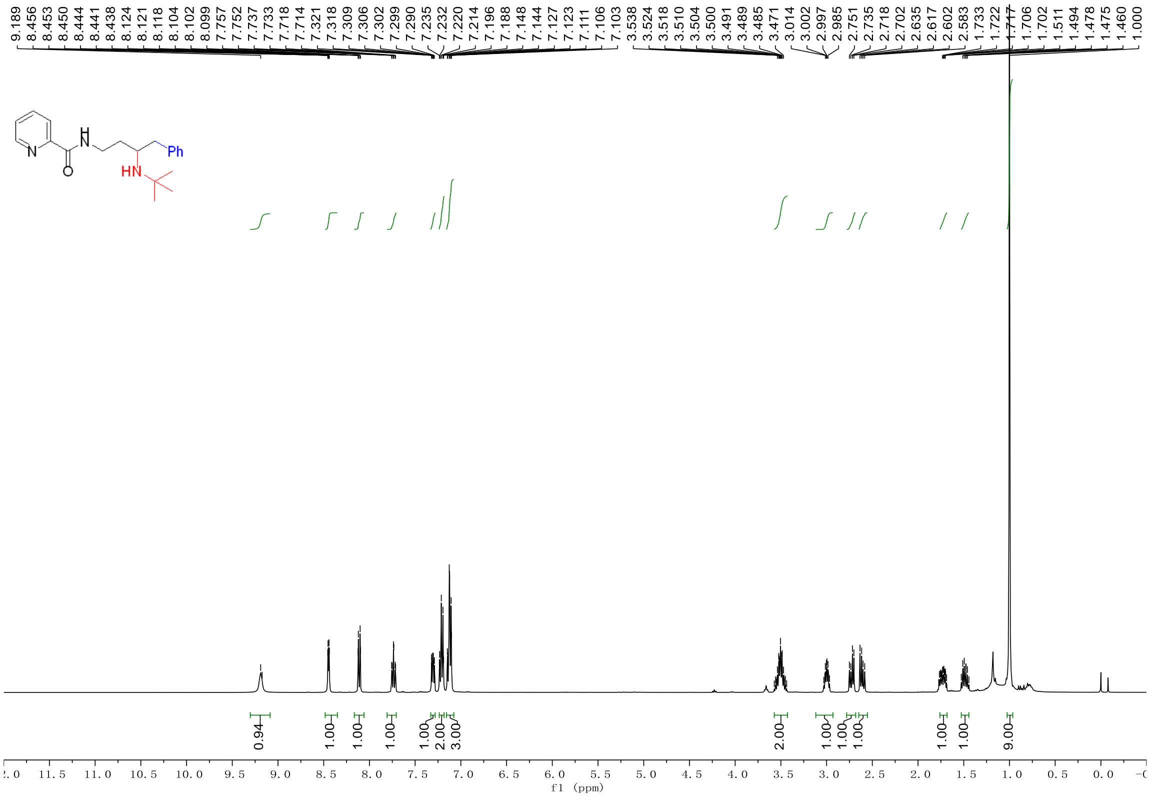

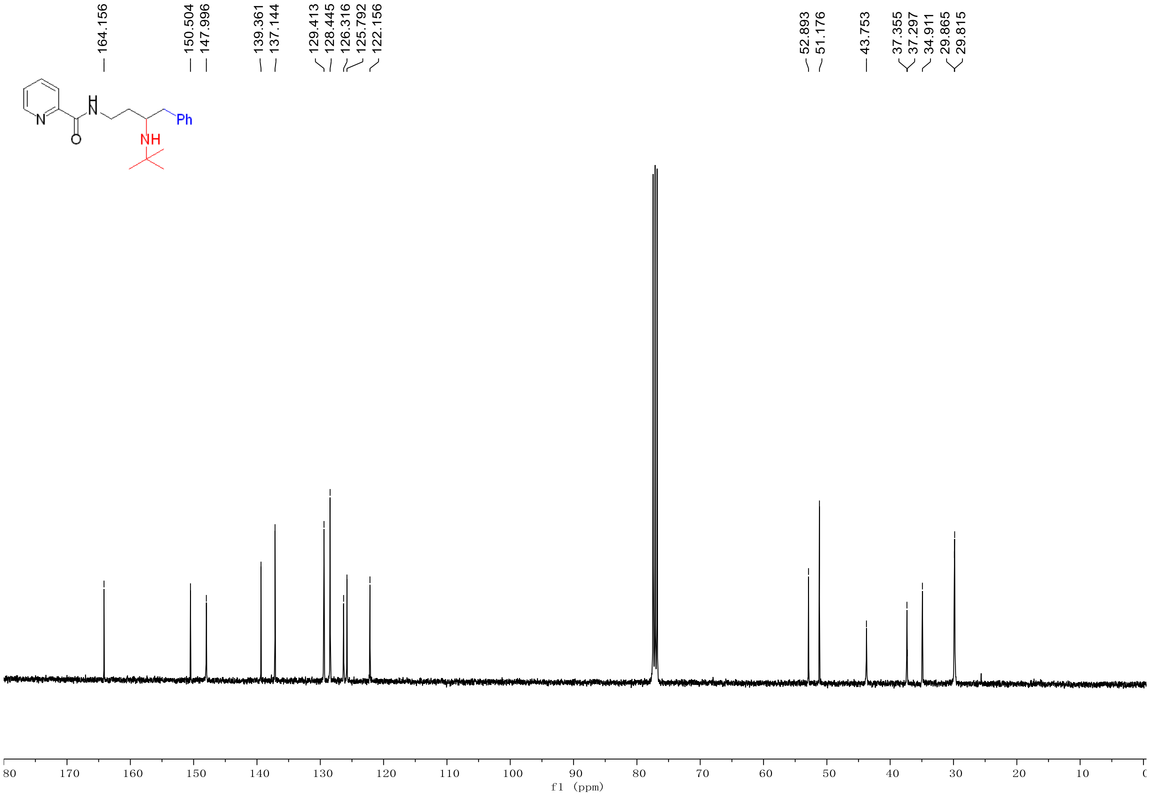

Supplementary Figure 46. ^{1}H NMR and ^{13}C NMR spectra of **3i**.

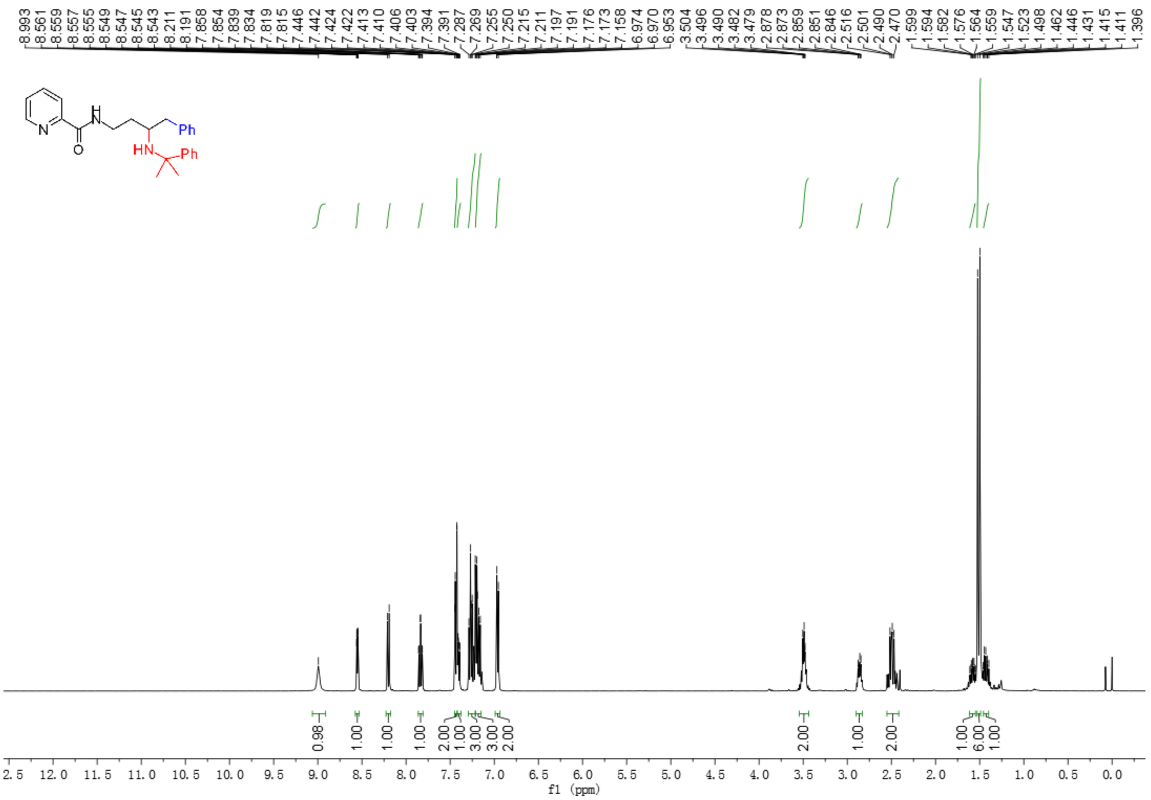

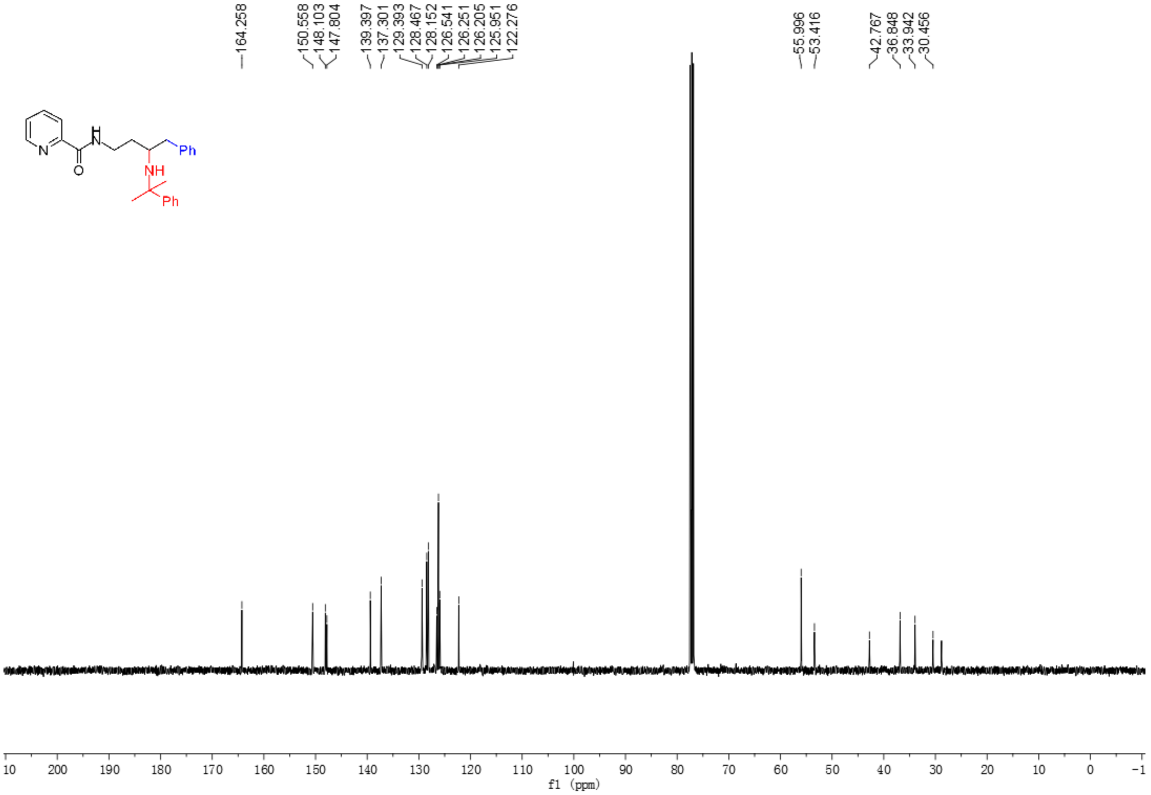

Supplementary Figure 47. ^{1}H NMR and ^{13}C NMR spectra of **3j**.

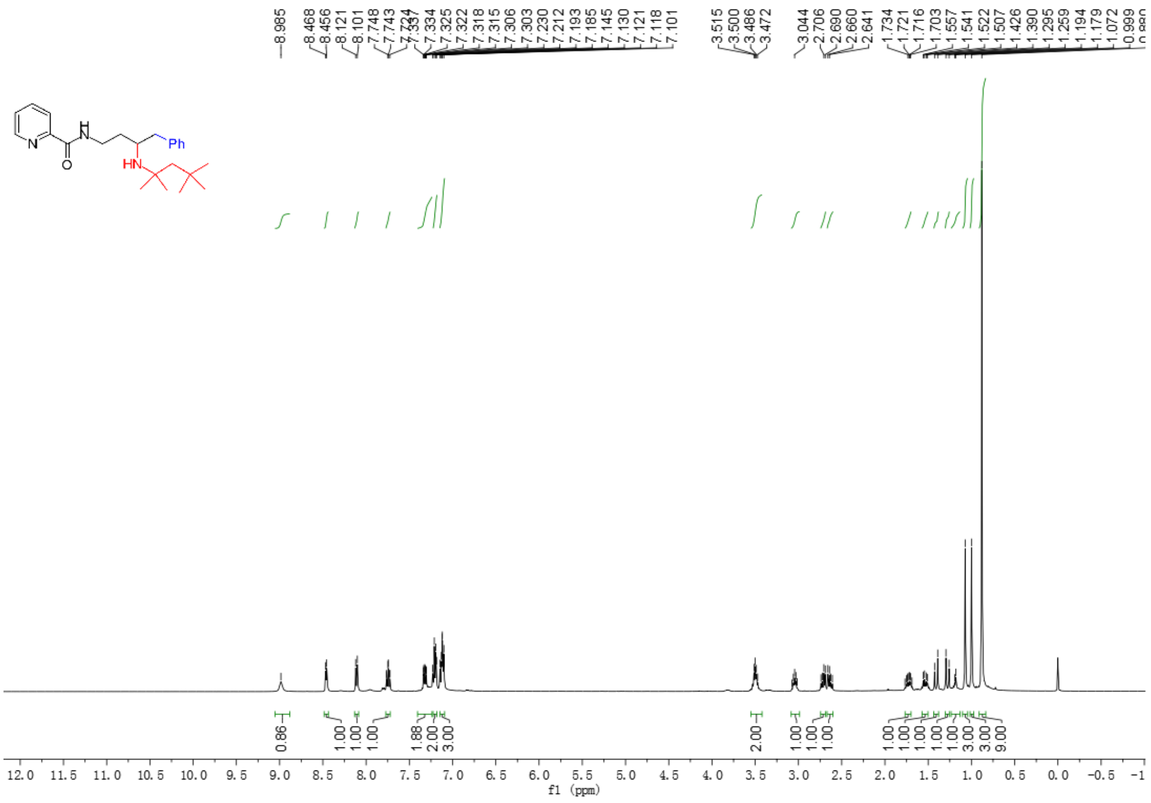

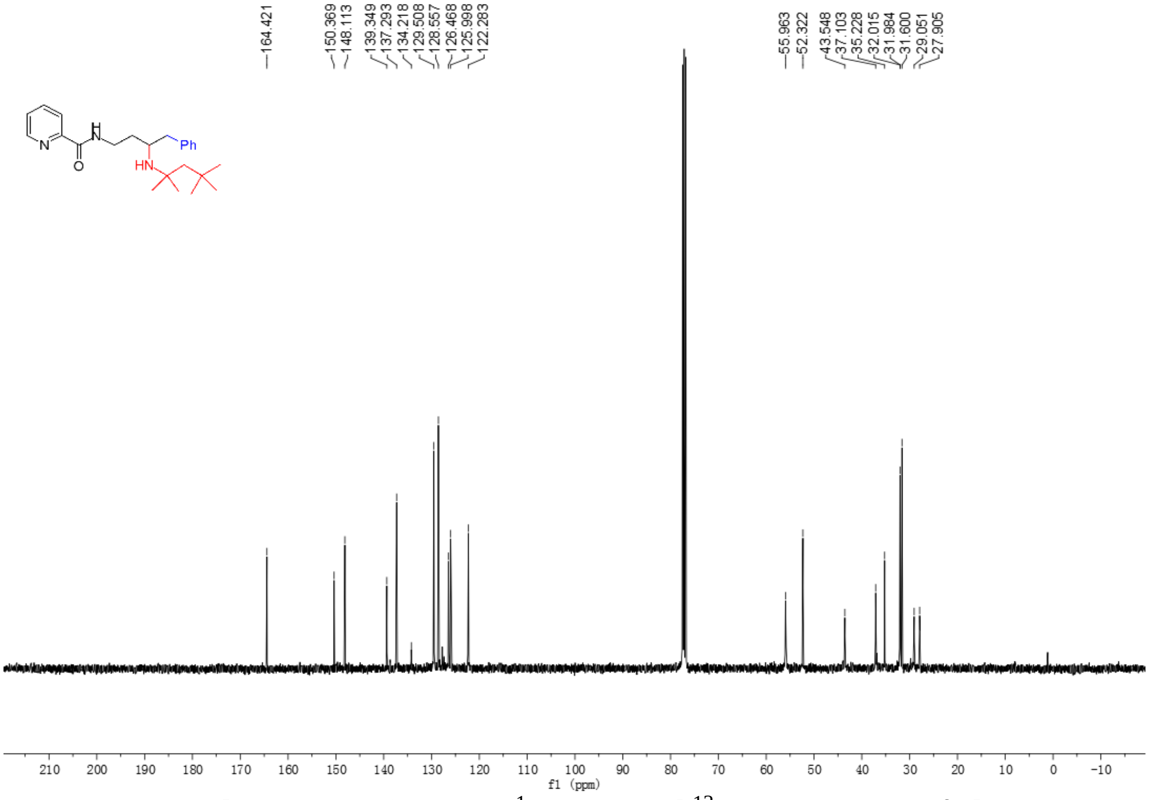

Supplementary Figure 48. ^{1}H NMR and ^{13}C NMR spectra of **3k**.

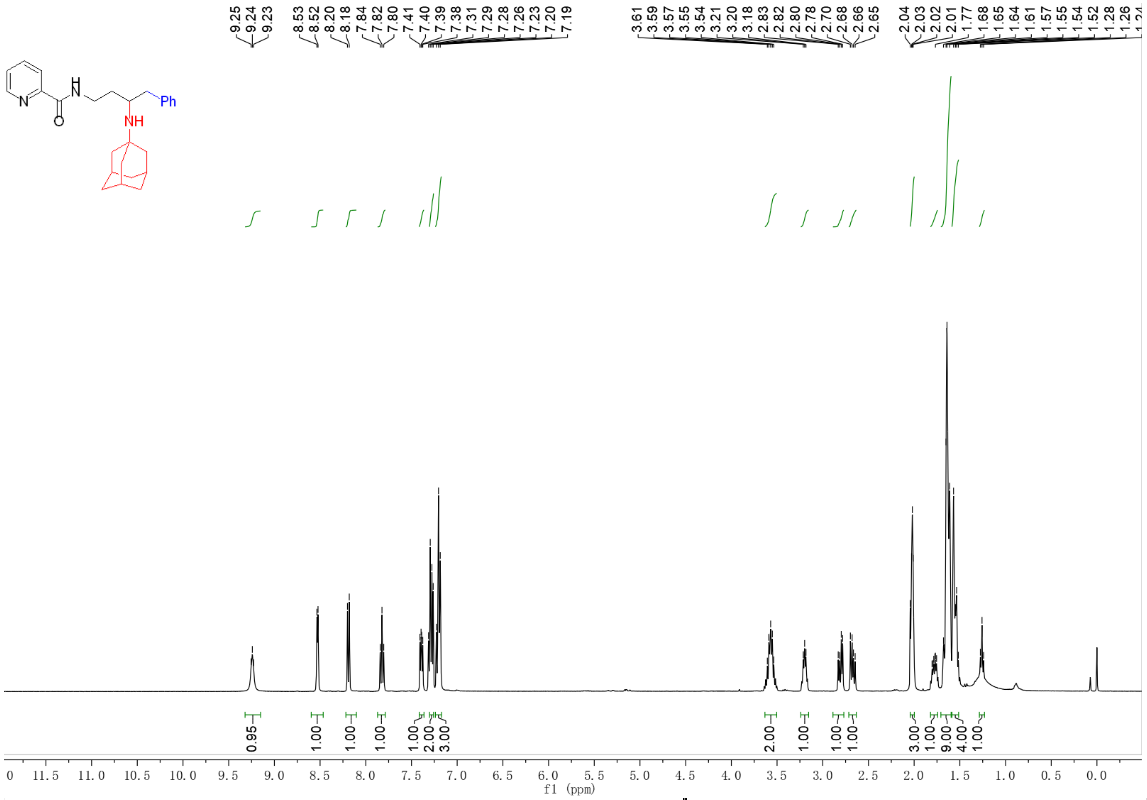

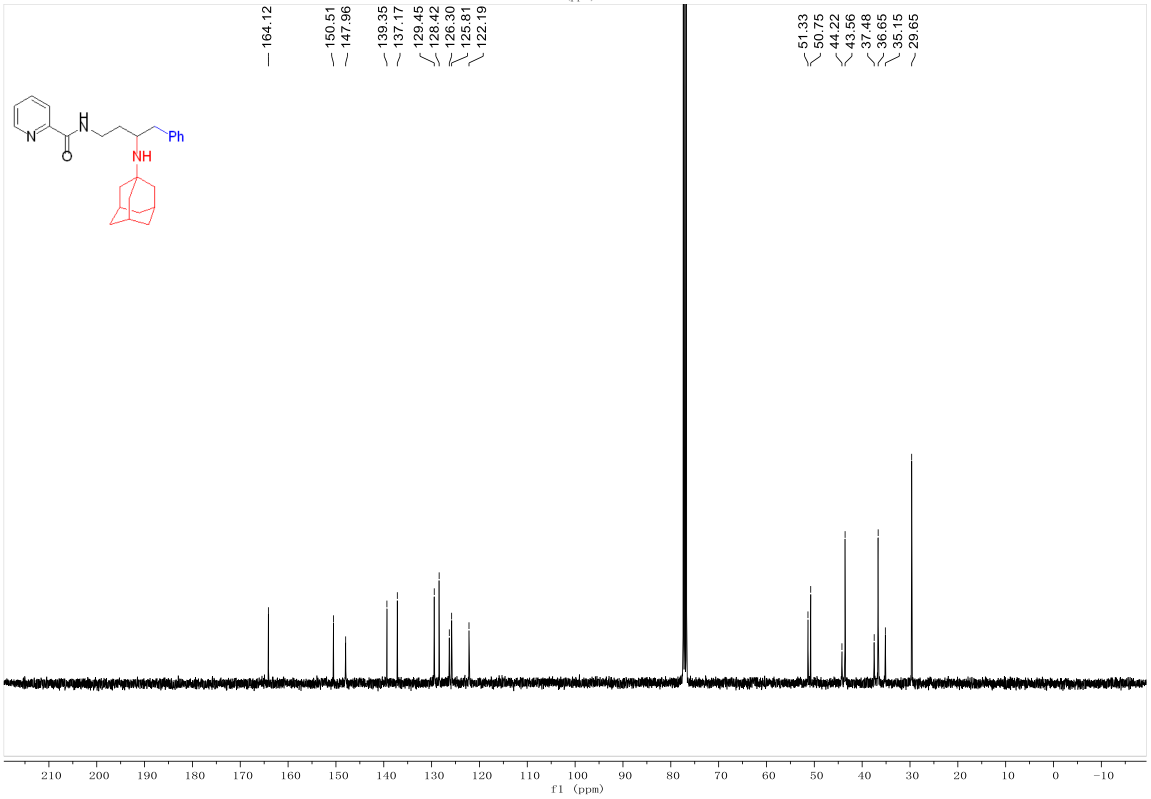

Supplementary Figure 49. ^{1}H NMR and ^{13}C NMR spectra of **3l**.

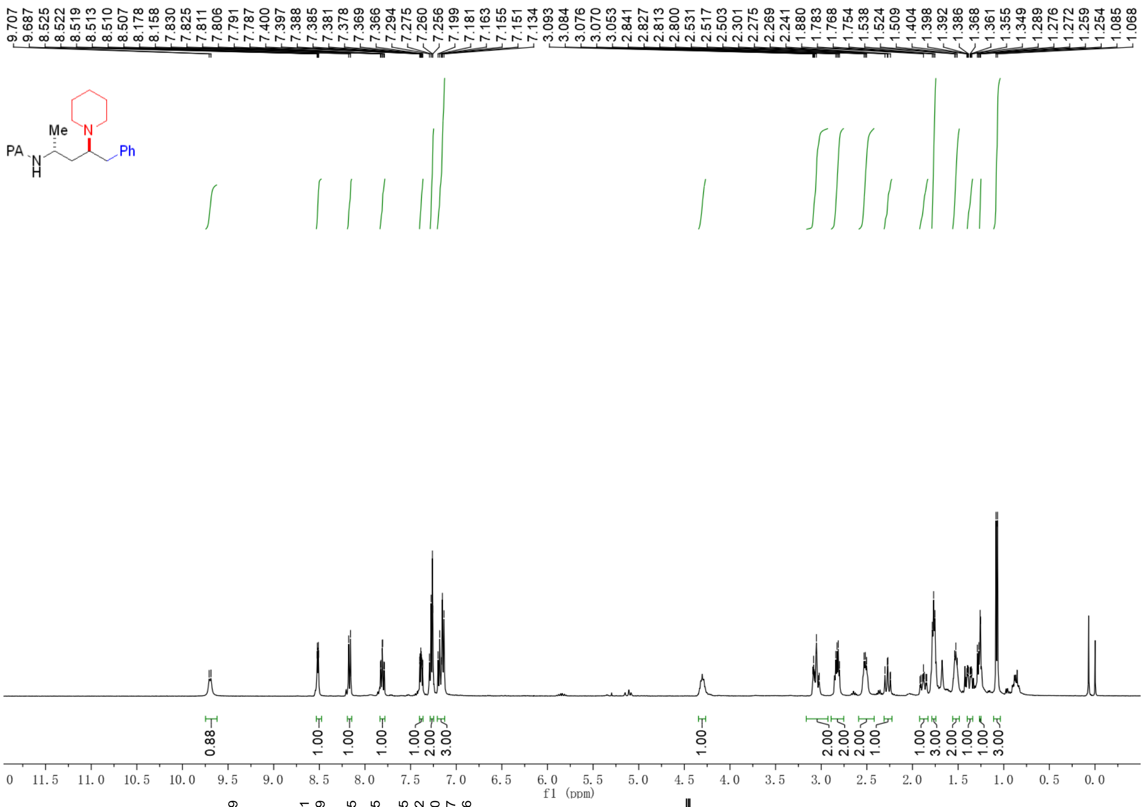

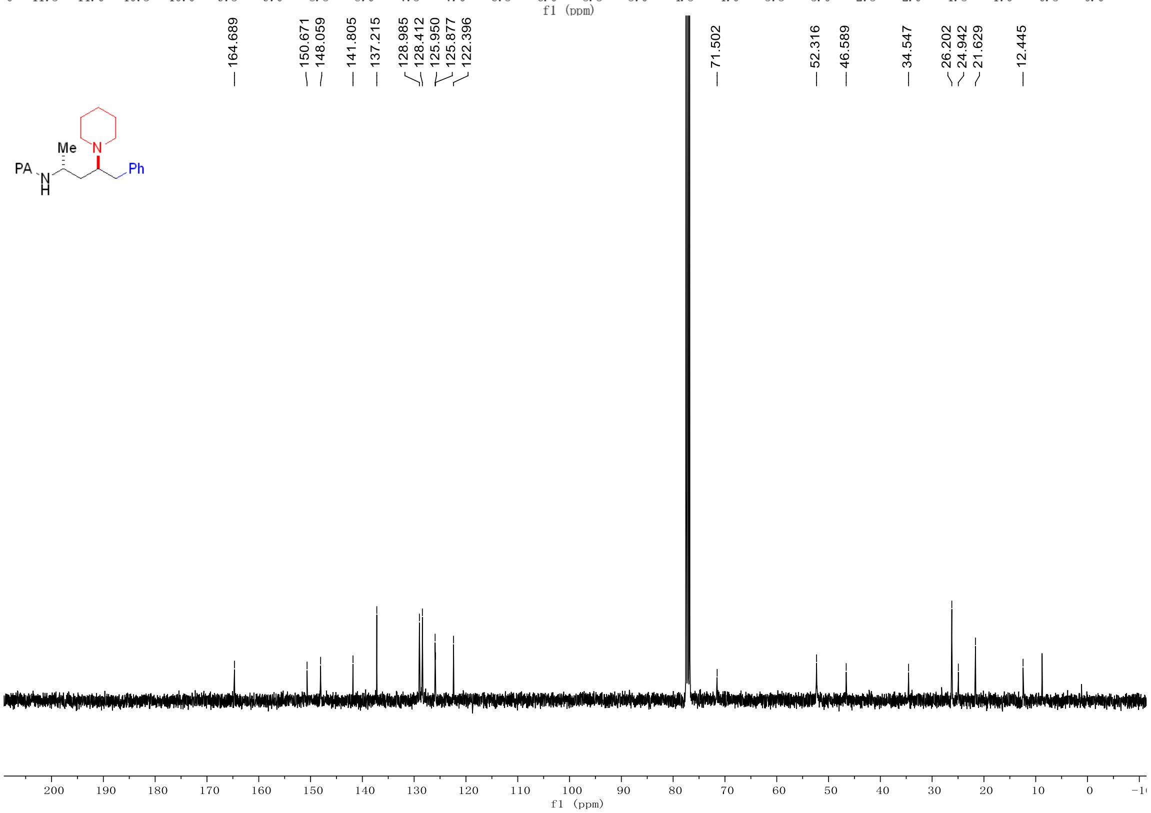

Supplementary Figure 50. ^{1}H NMR and ^{13}C NMR spectra of **4a**.

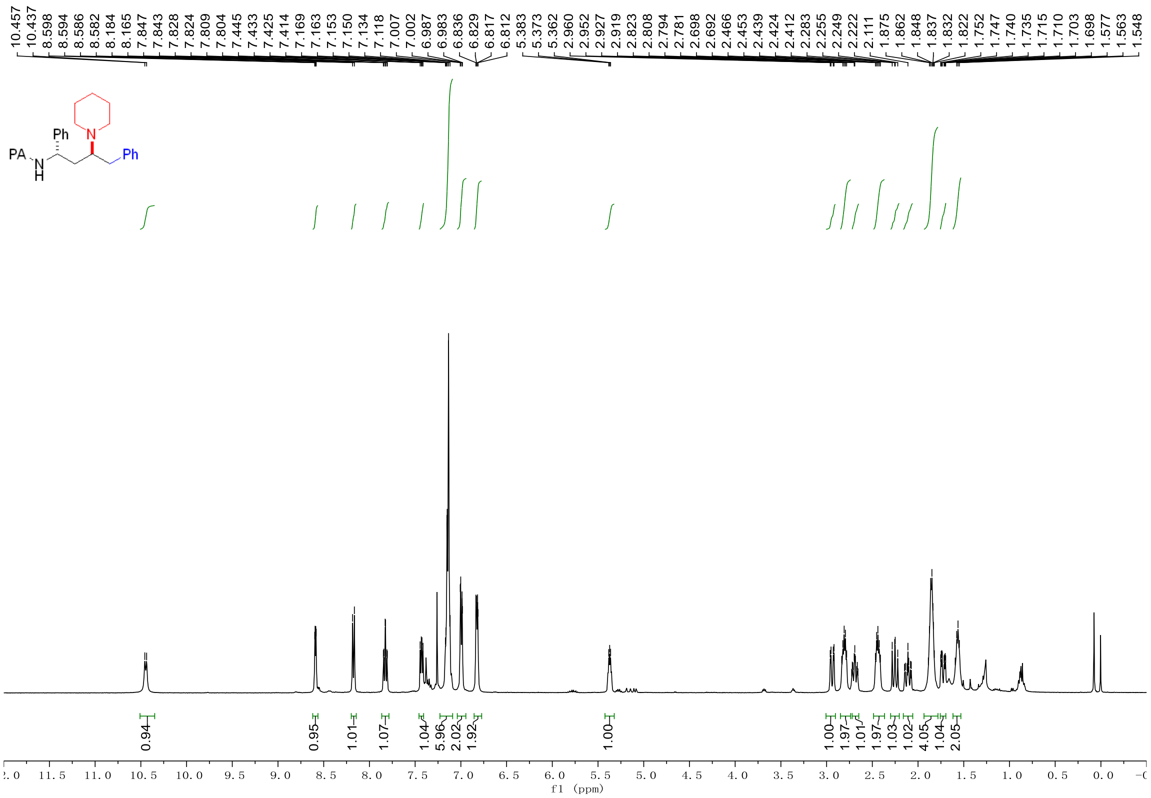

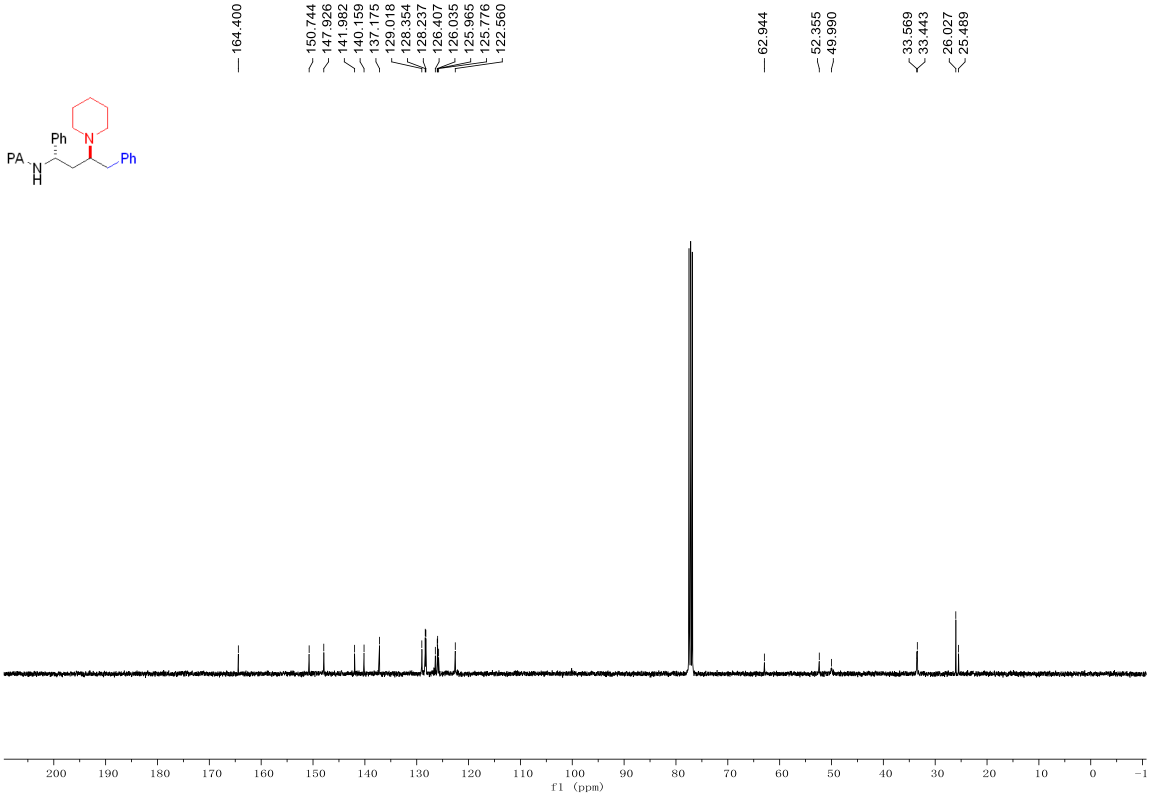

Supplementary Figure 51. ^{1}H NMR and ^{13}C NMR spectra of **4b**.

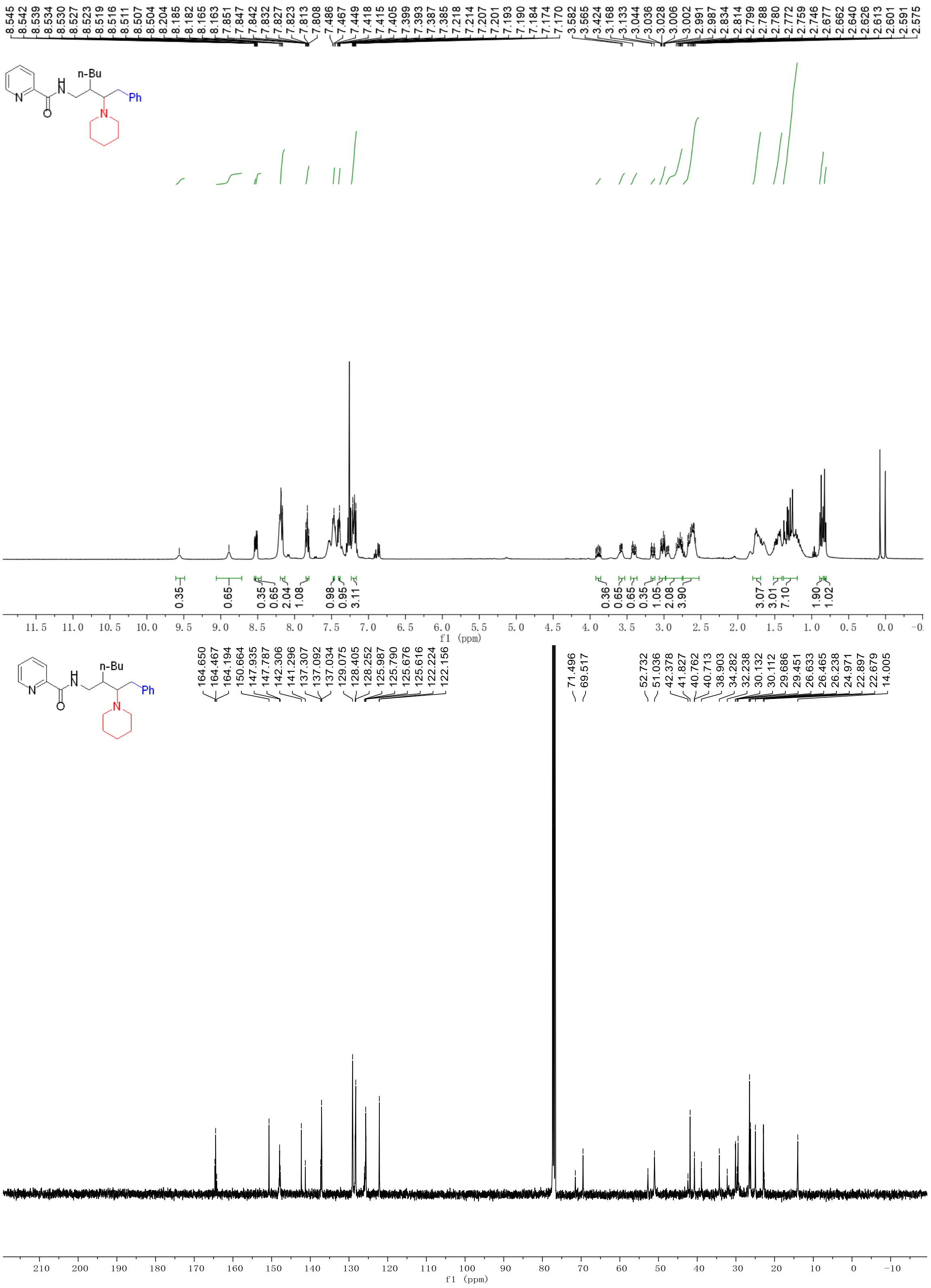

Supplementary Figure 52. ^{1}H NMR and ^{13}C NMR spectra of **4c**.

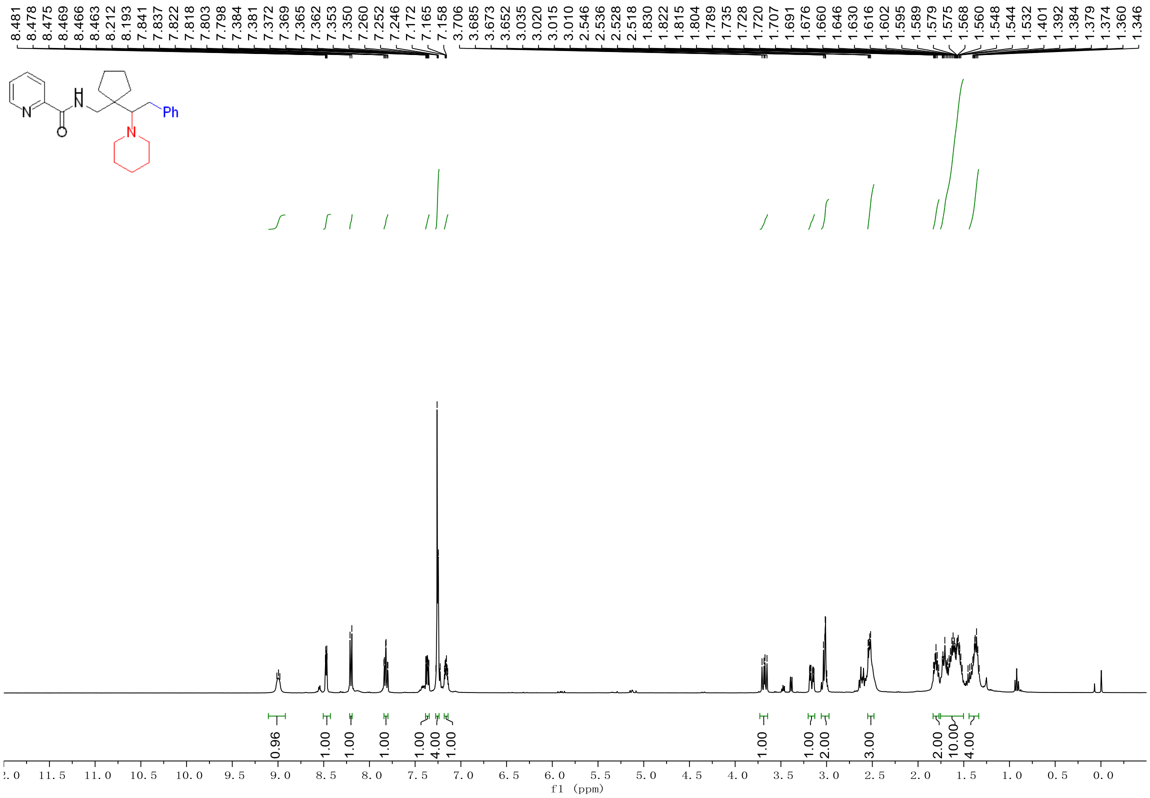

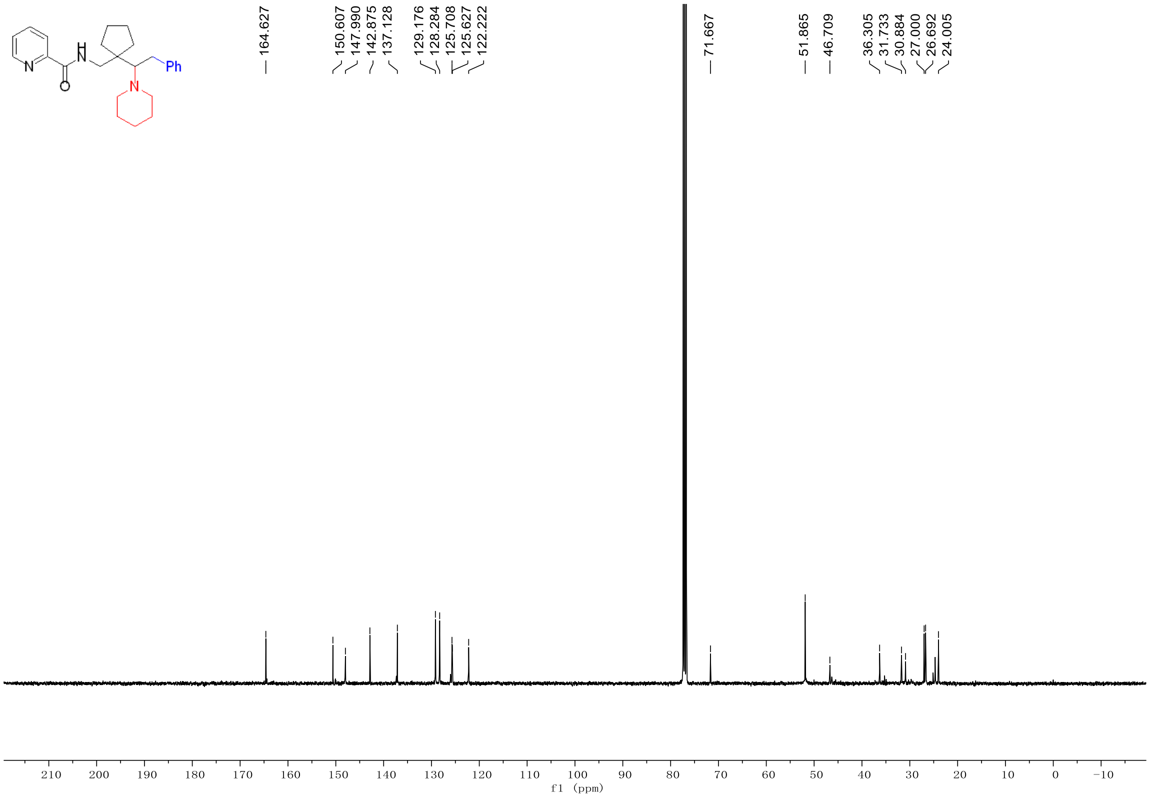

Supplementary Figure 53. ^{1}H NMR and ^{13}C NMR spectra of **4d**.

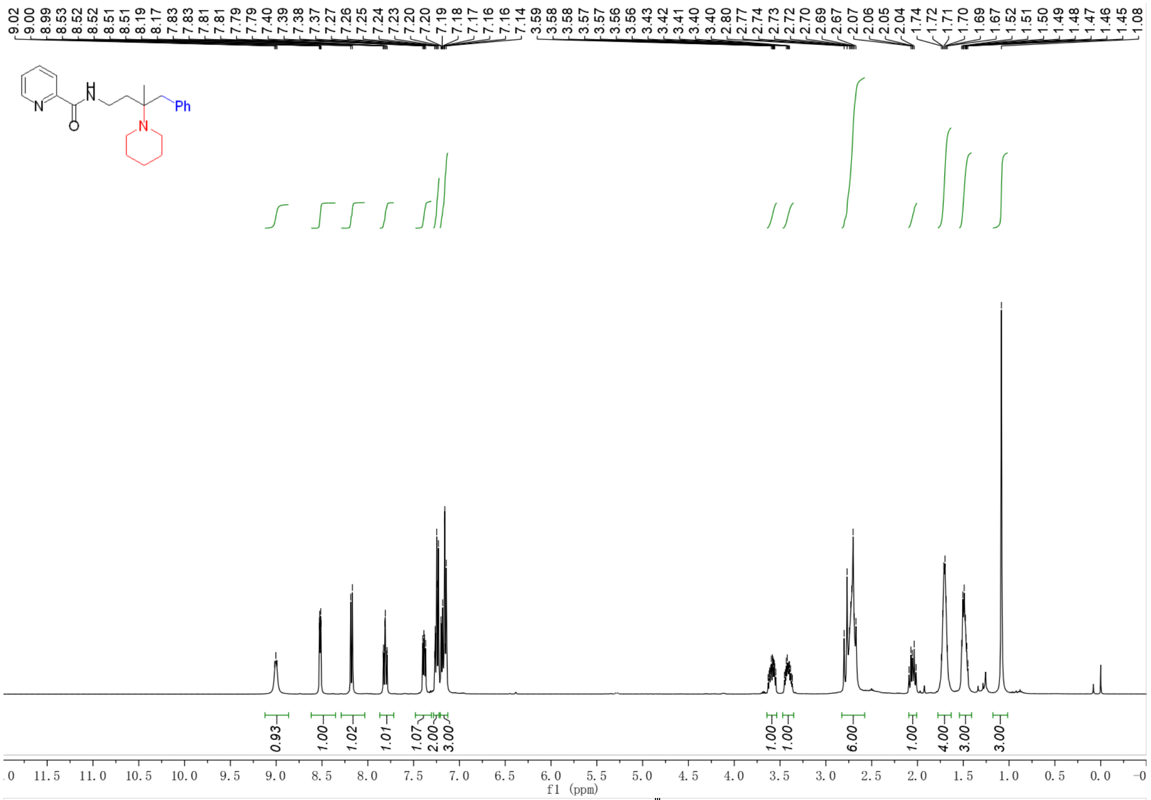

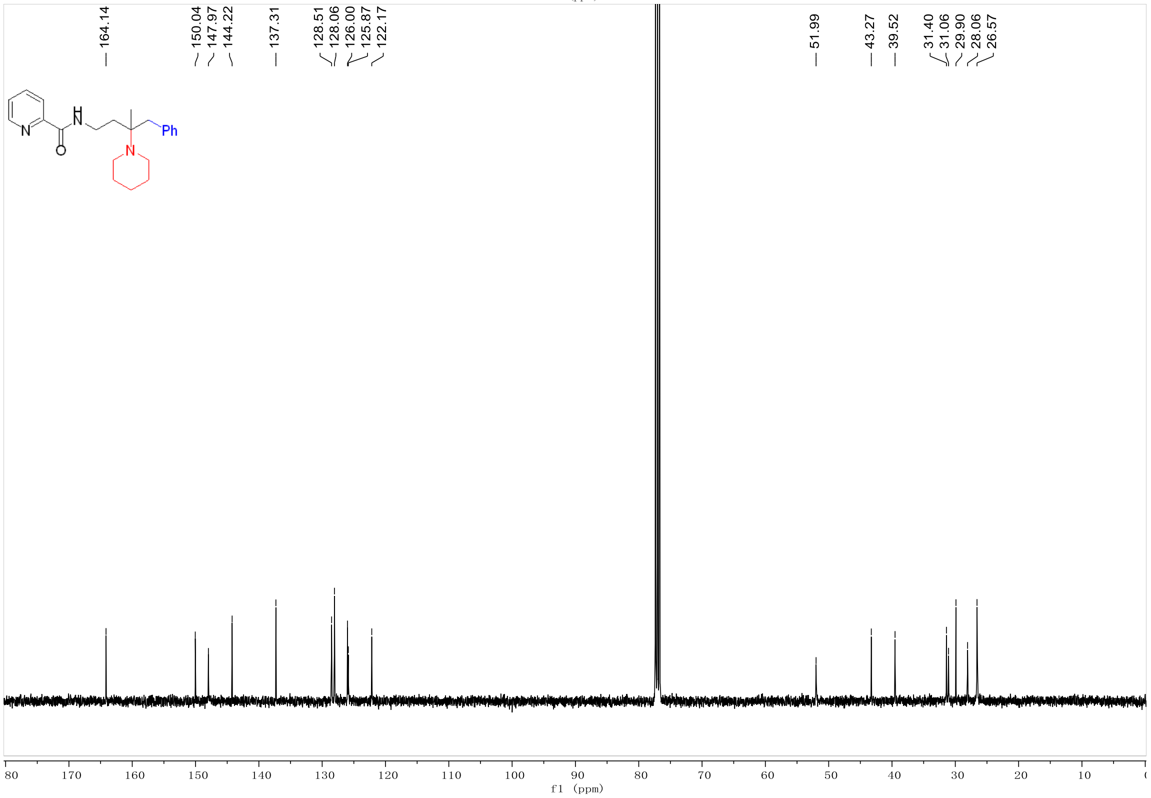

Supplementary Figure 54. ^{1}H NMR and ^{13}C NMR spectra of **4e**.

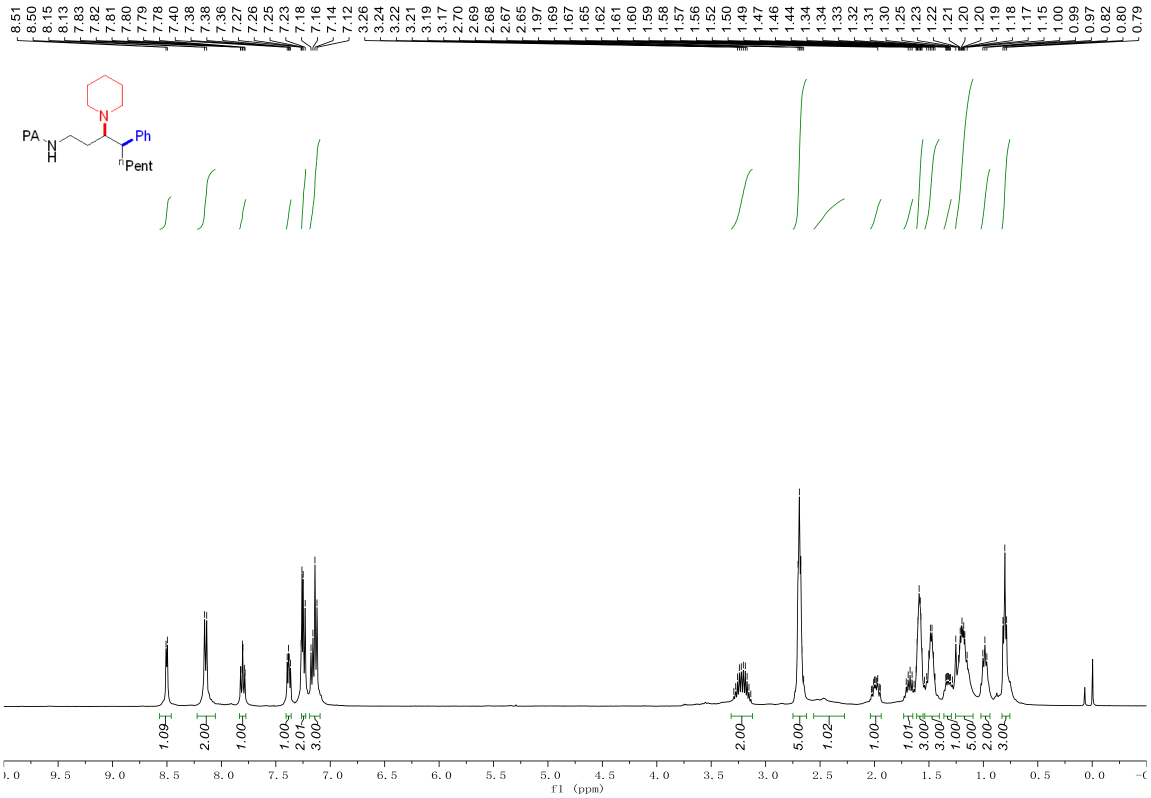

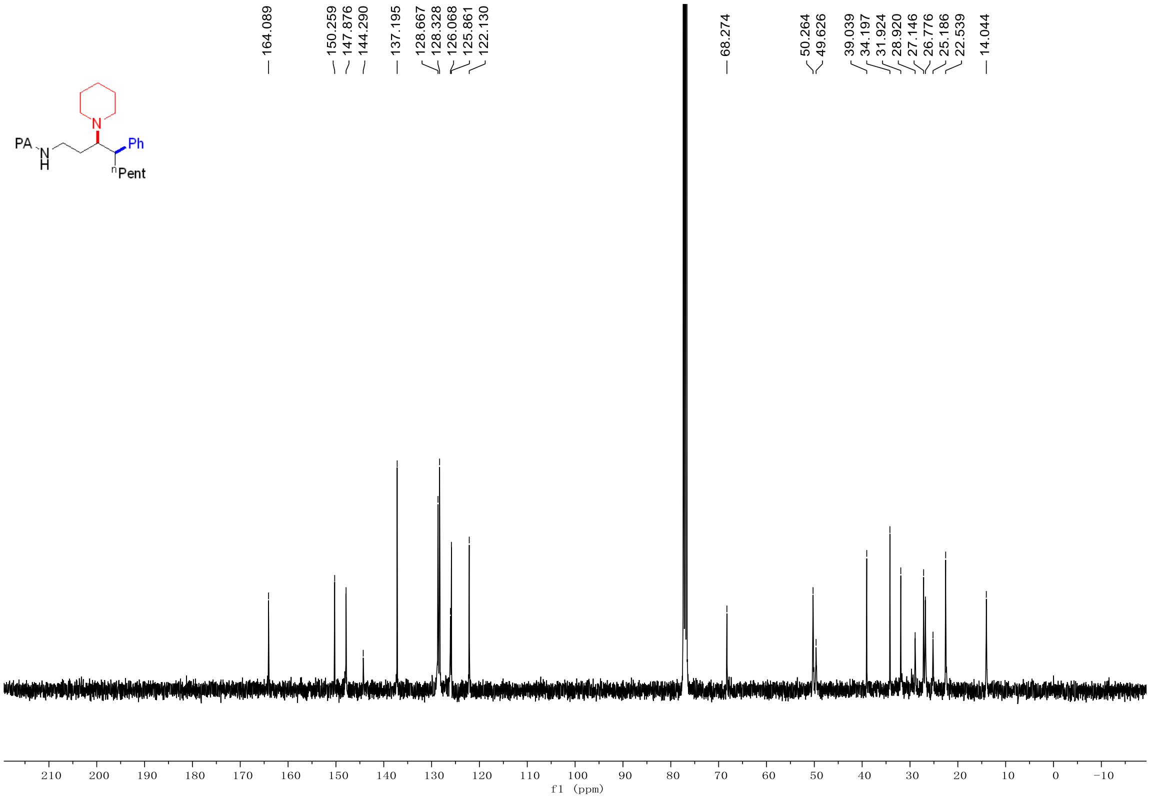

Supplementary Figure 55. ^{1}H NMR and ^{13}C NMR spectra of **4f**.

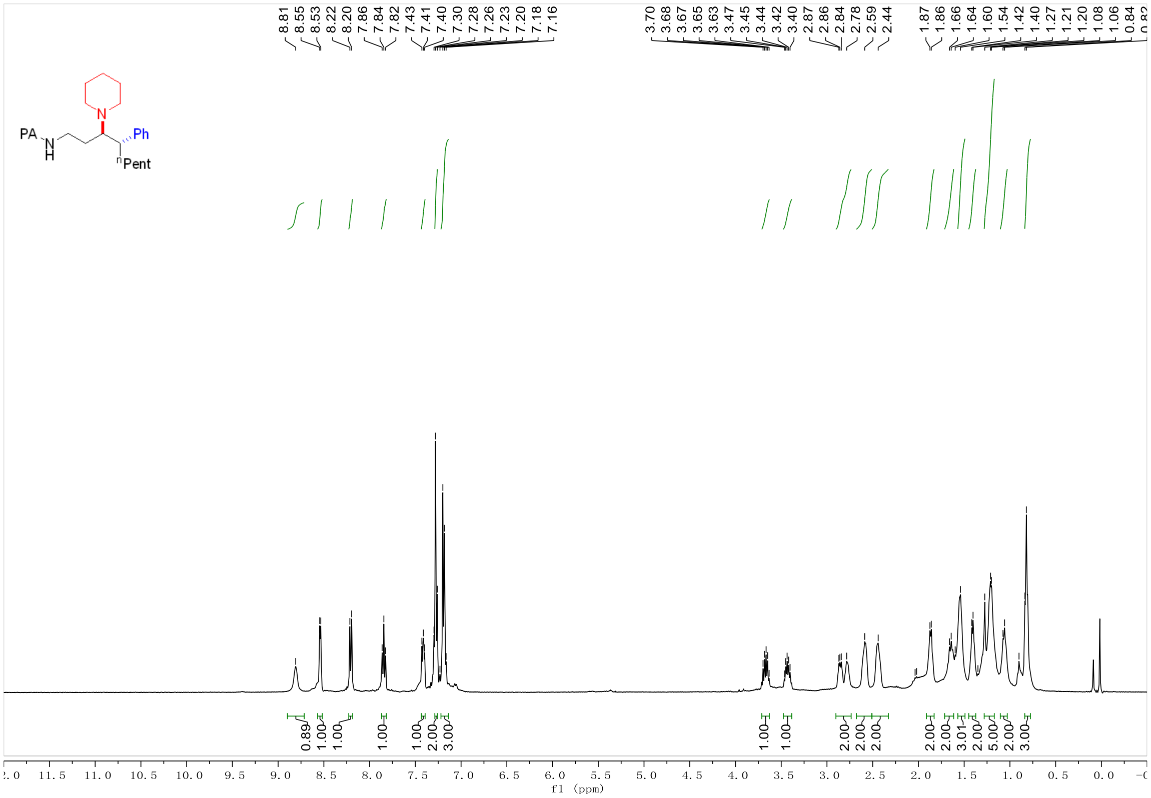

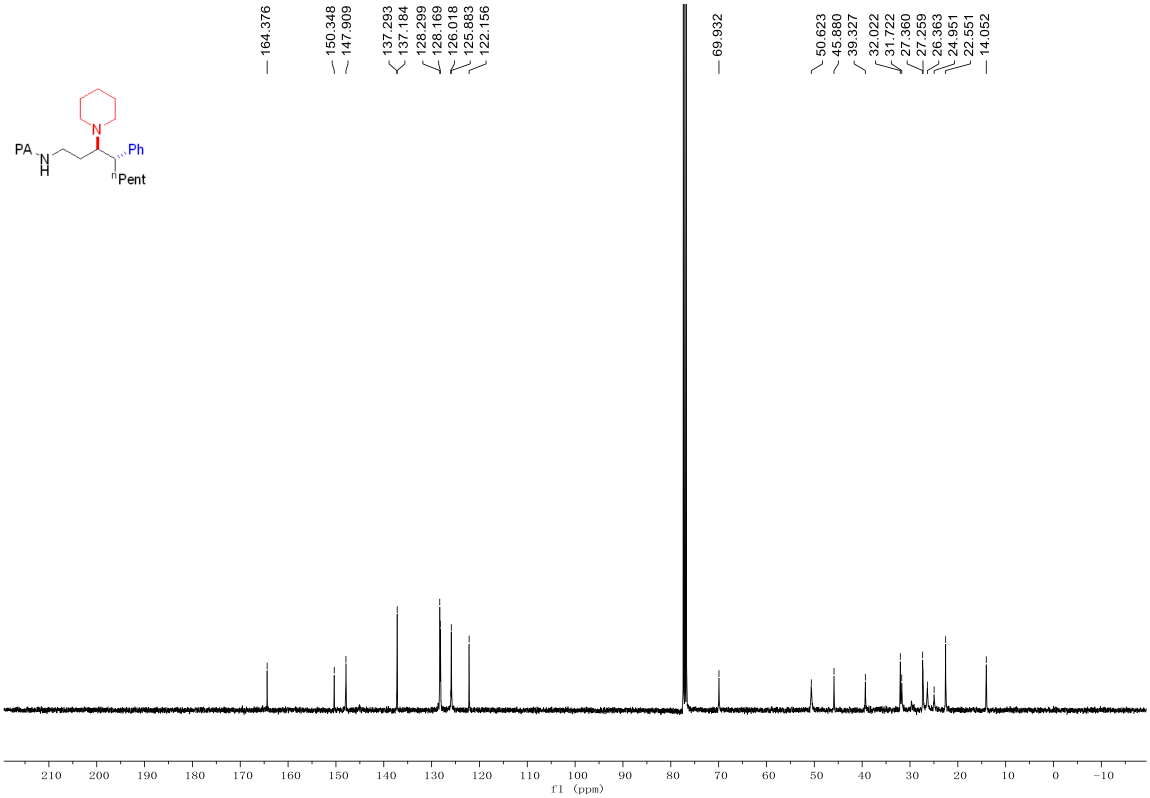

Supplementary Figure 56. ^{1}H NMR and ^{13}C NMR spectra of **4g**.

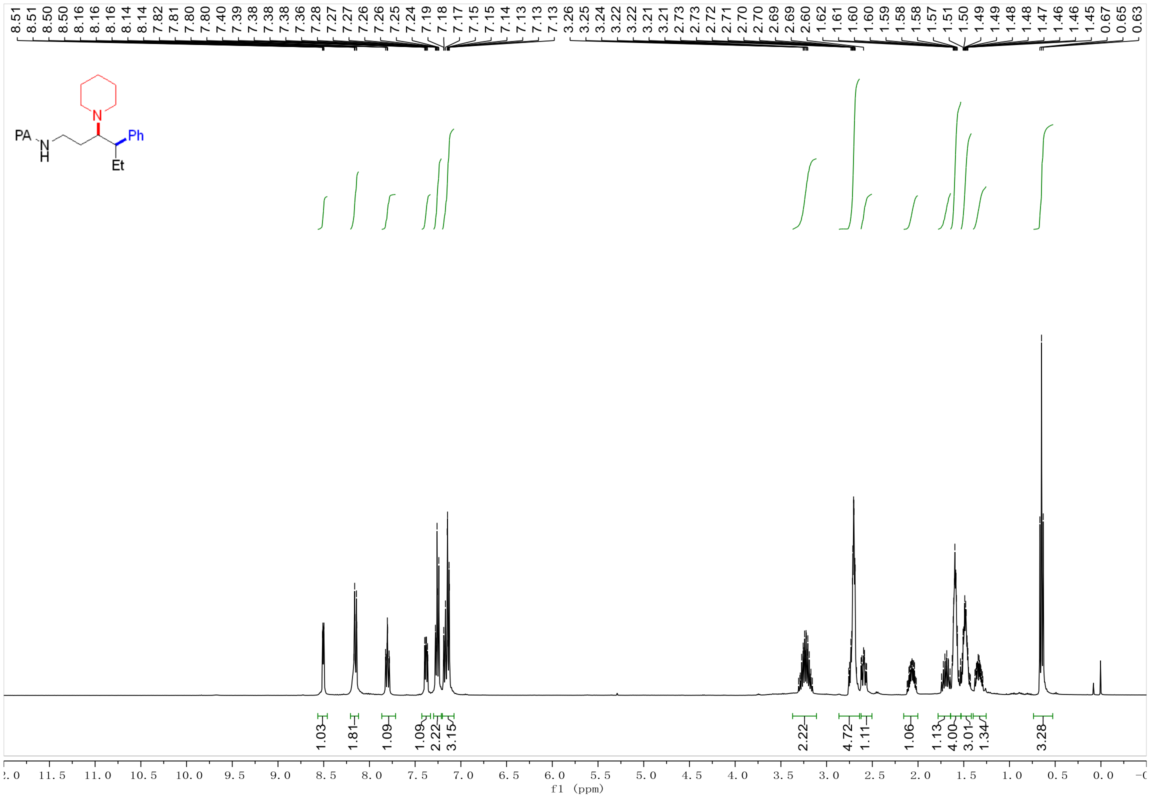

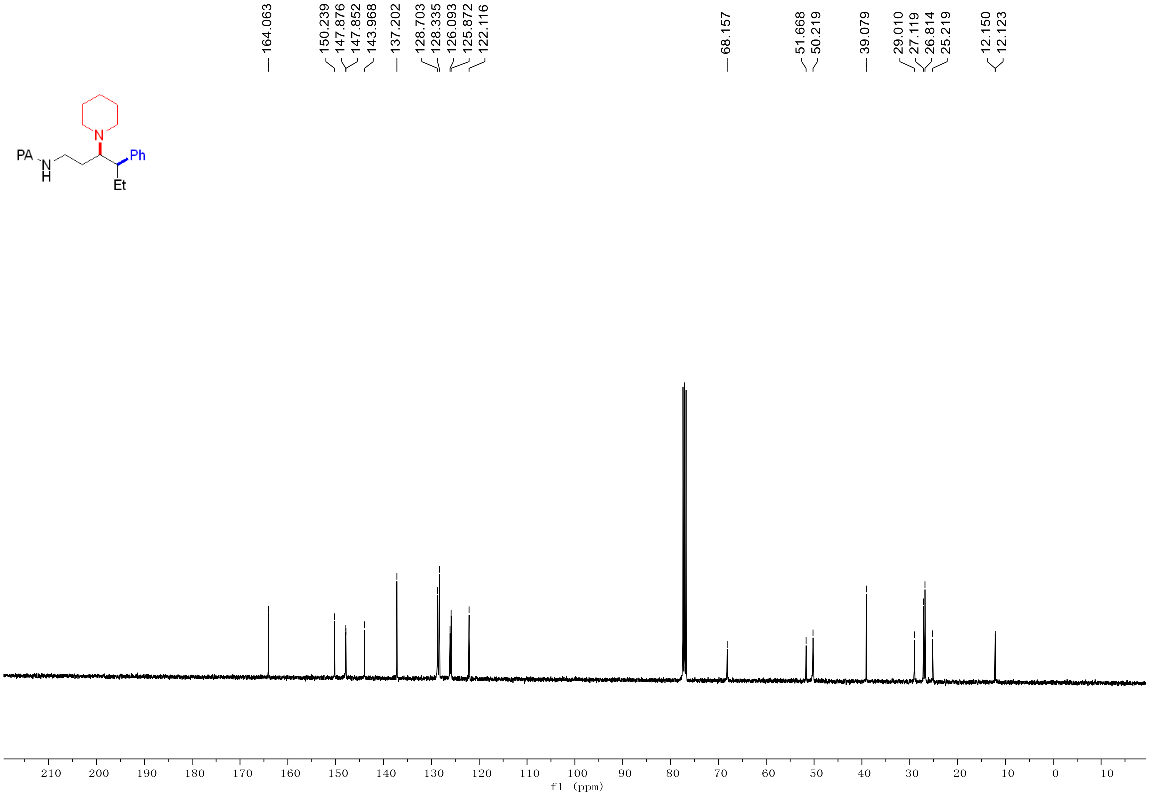

Supplementary Figure 57. ^{1}H NMR and ^{13}C NMR spectra of **4h**.

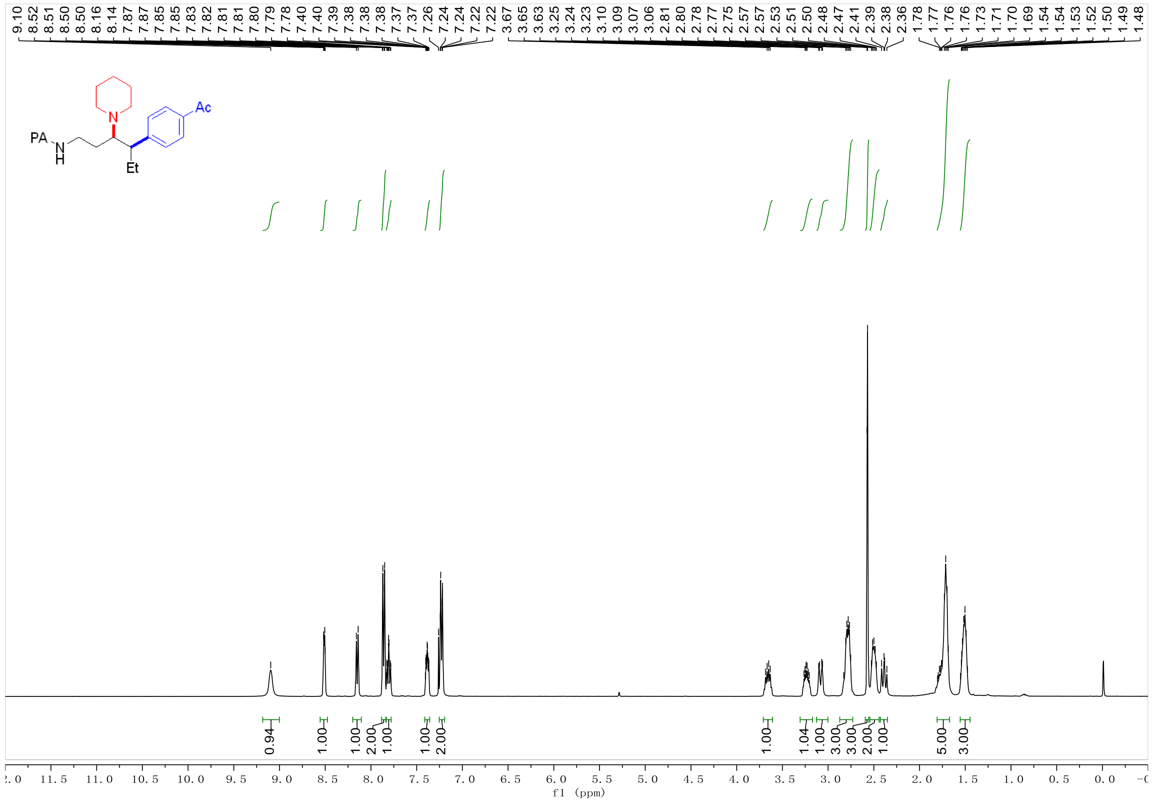

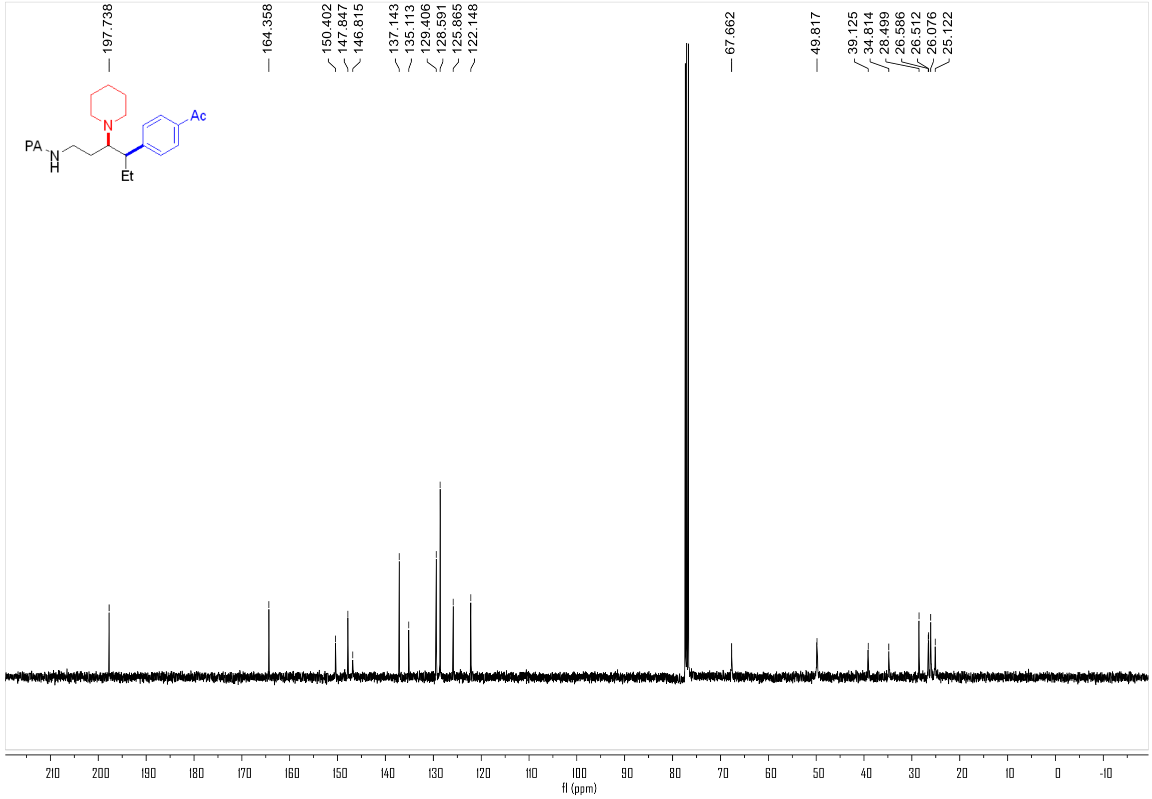

Supplementary Figure 58. ^{1}H NMR and ^{13}C NMR spectra of **4i**.

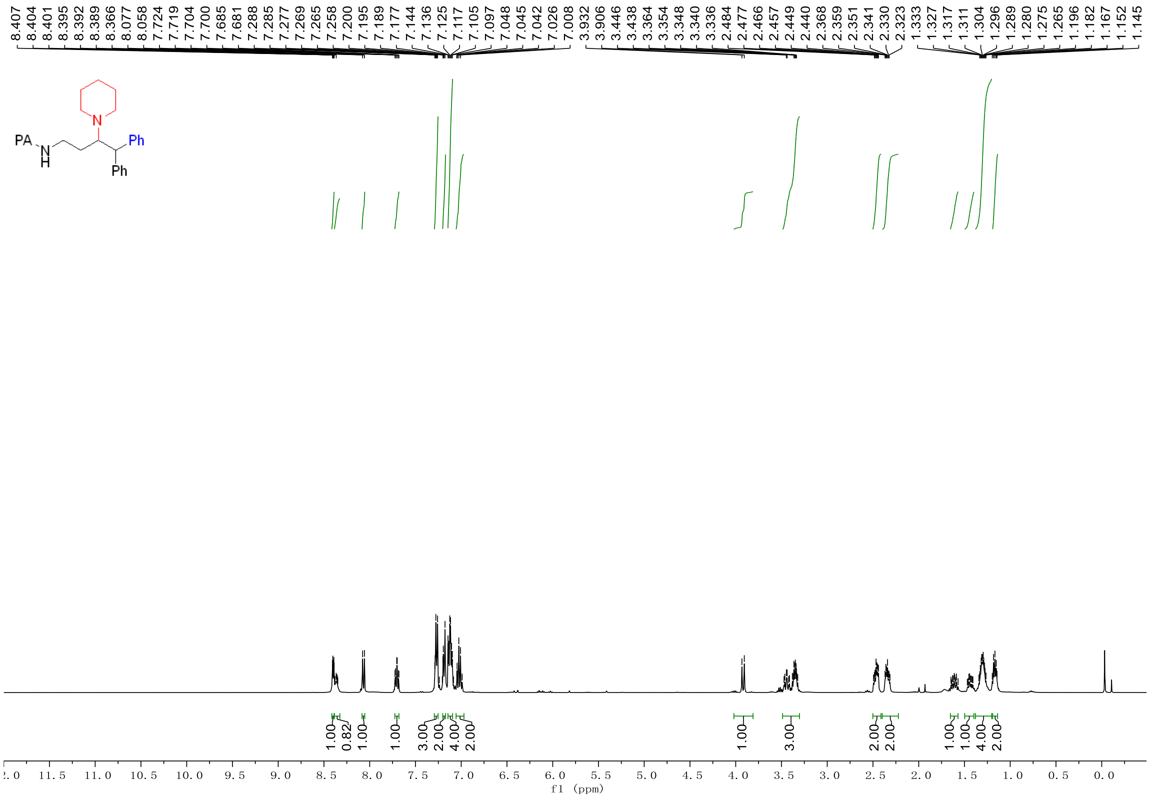

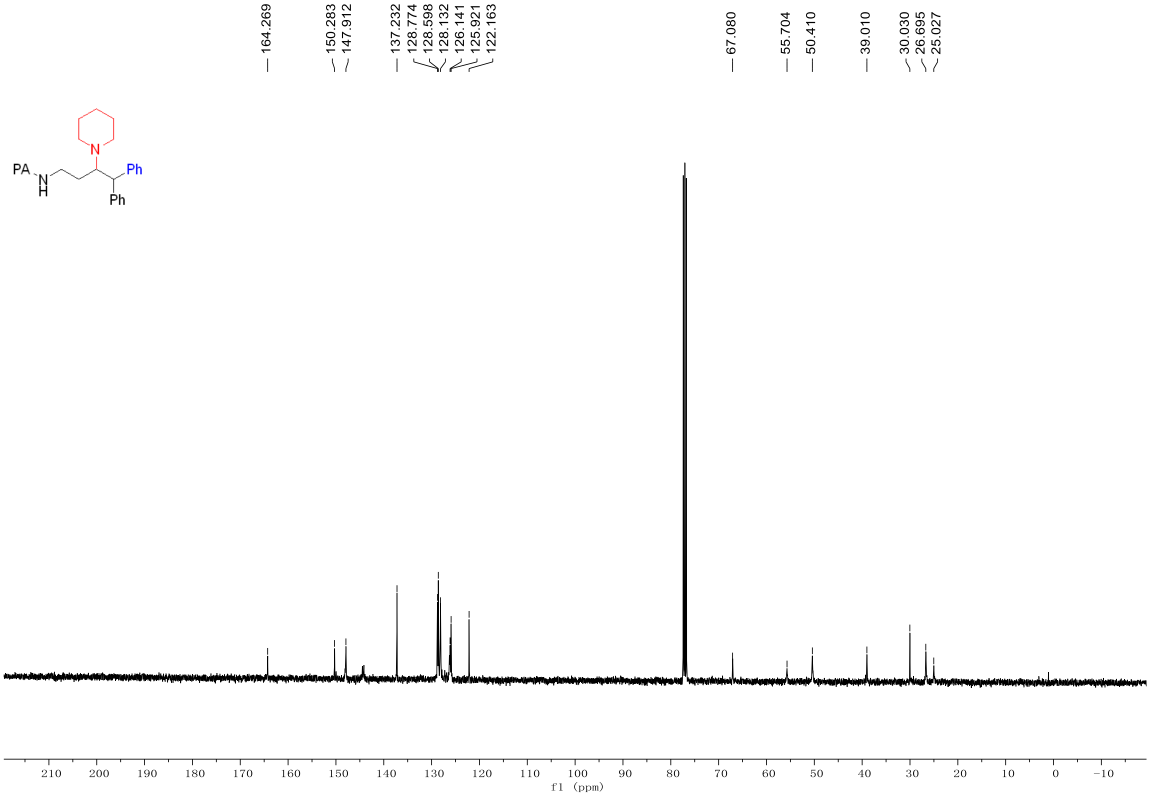

Supplementary Figure 59. ^{1}H NMR and ^{13}C NMR spectra of **4j**.

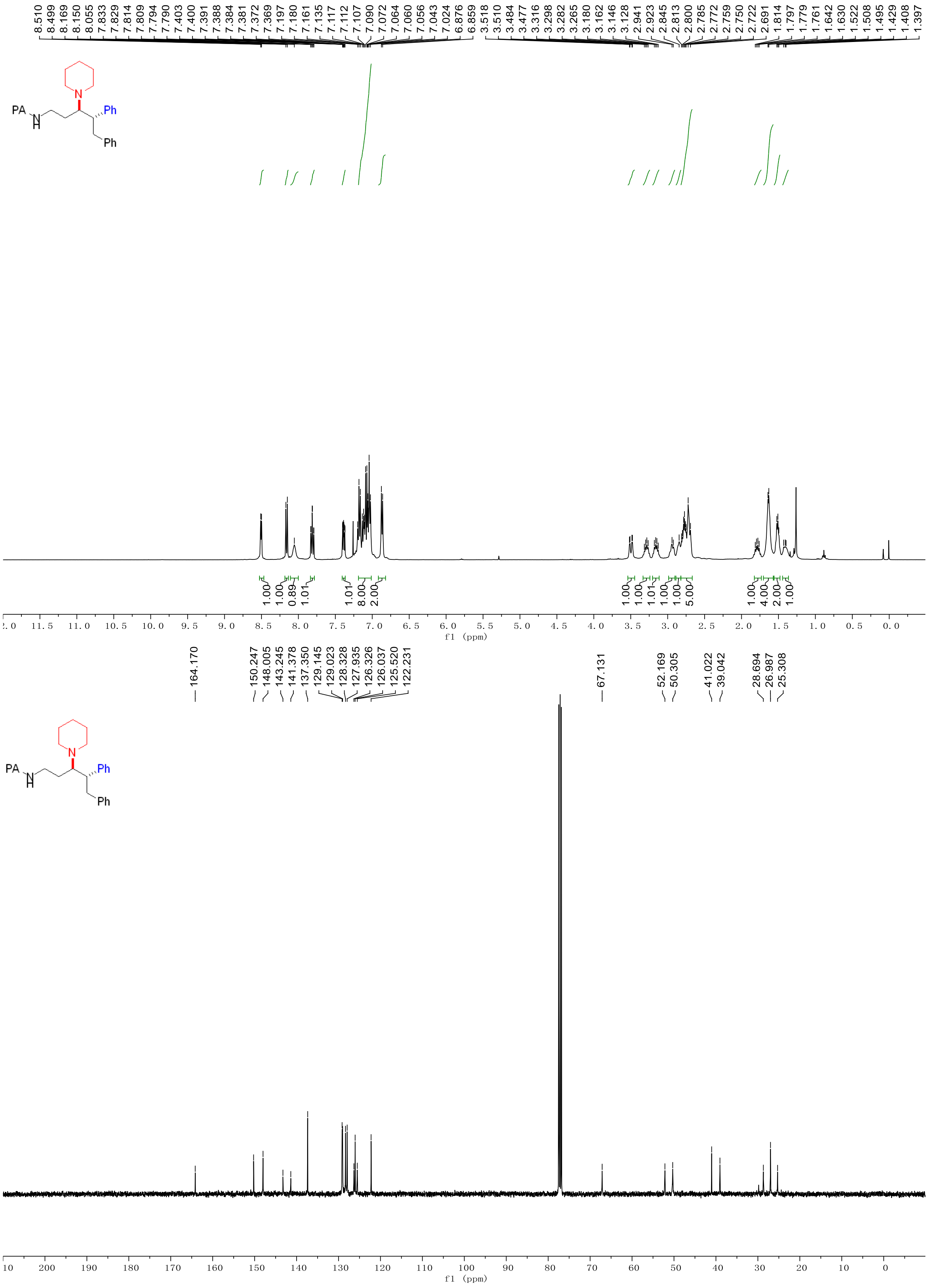

Supplementary Figure 60. ^{1}H NMR and ^{13}C NMR spectra of **4k**.

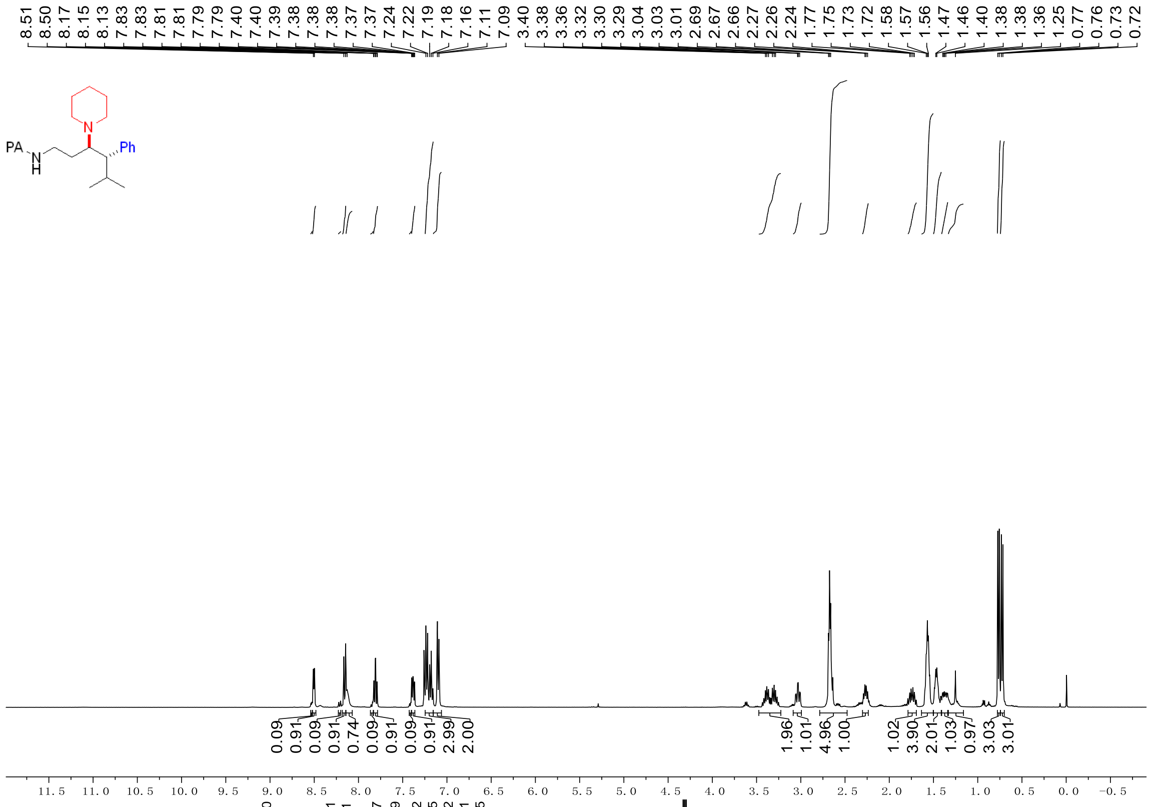

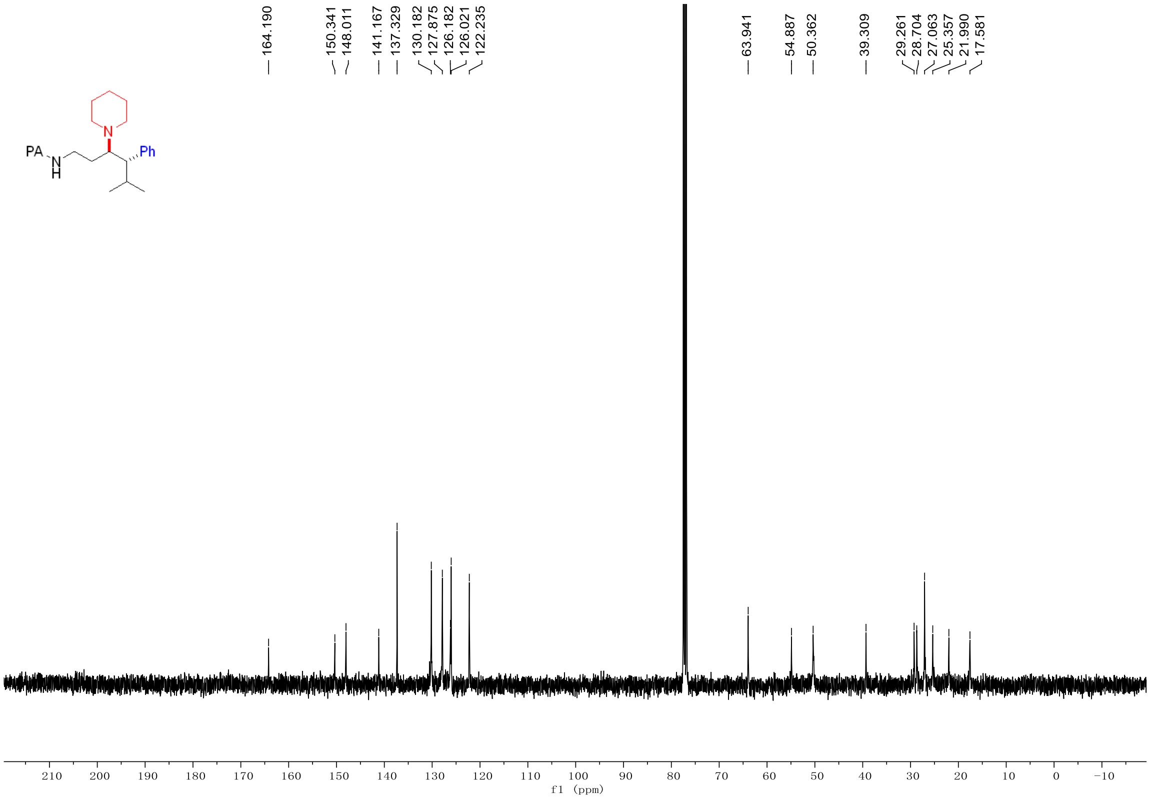

Supplementary Figure 61. ^{1}H NMR and ^{13}C NMR spectra of **4l**.

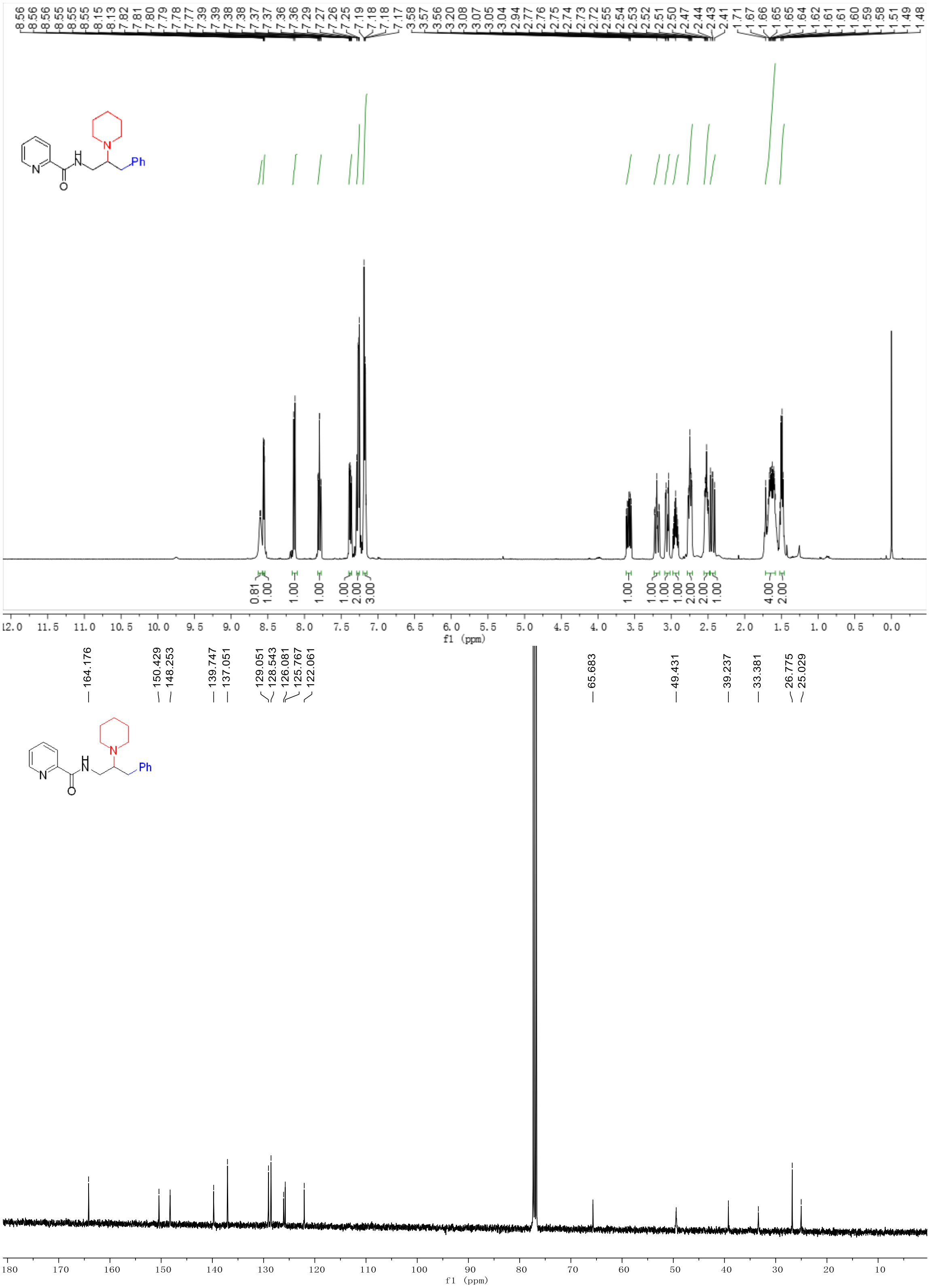

Supplementary Figure 62. ^{1}H NMR and ^{13}C NMR spectra of **4m**.

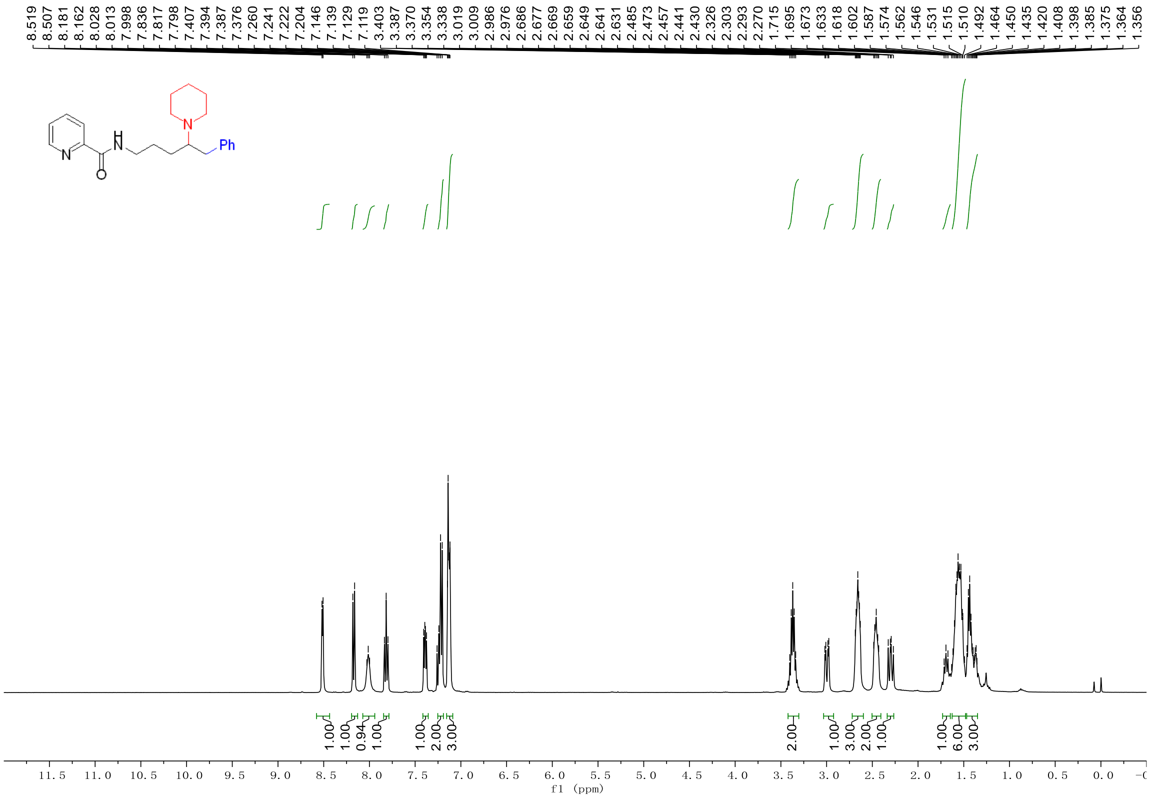

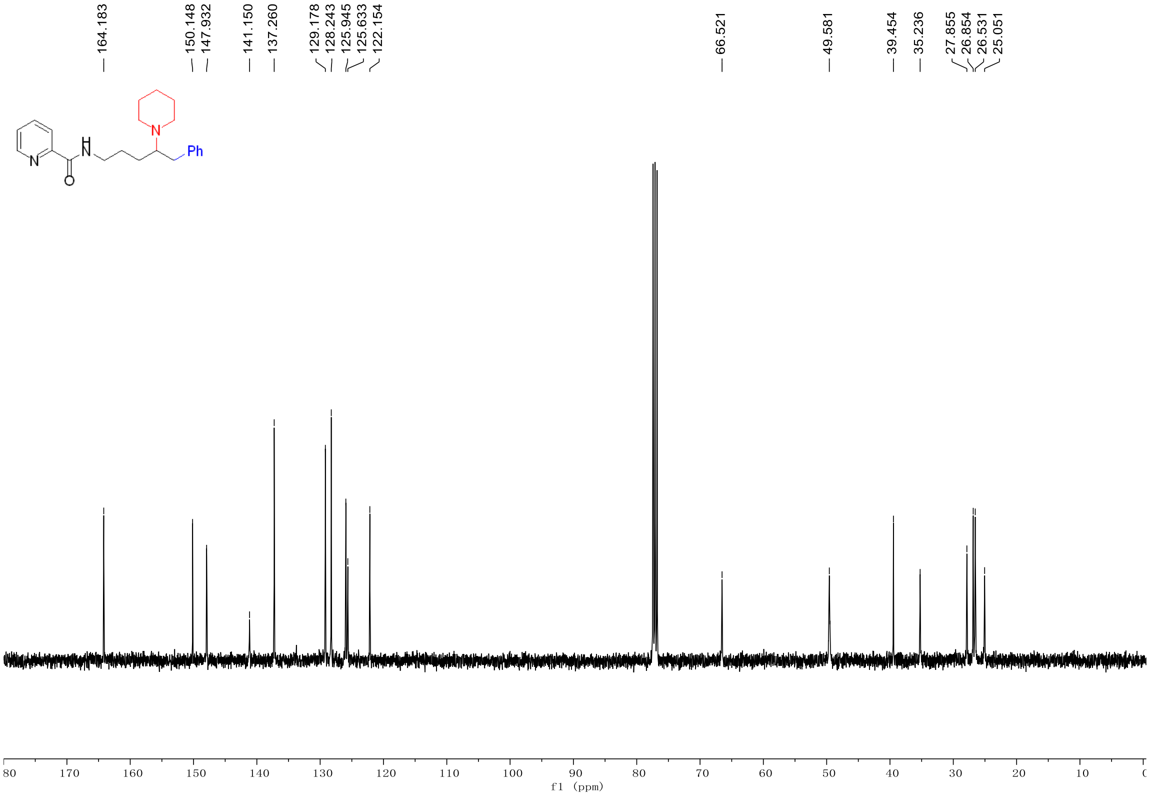

Supplementary Figure 63. ^{1}H NMR and ^{13}C NMR spectra of **4n**.

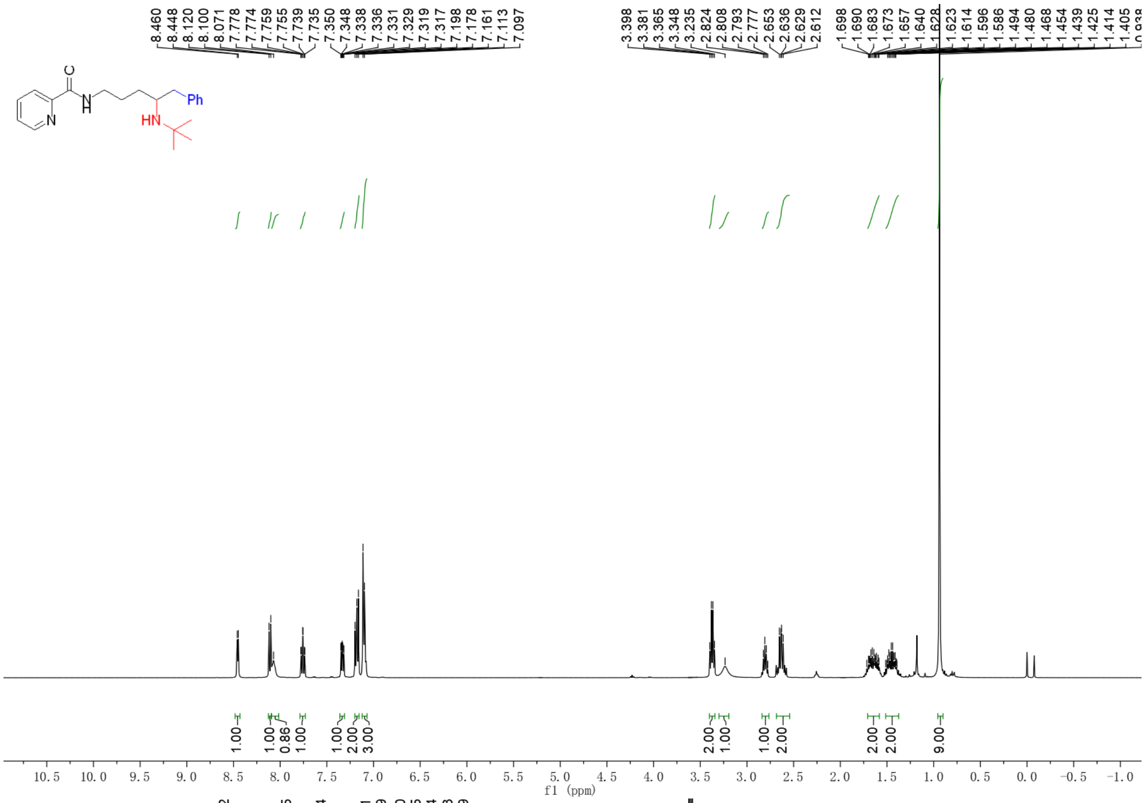

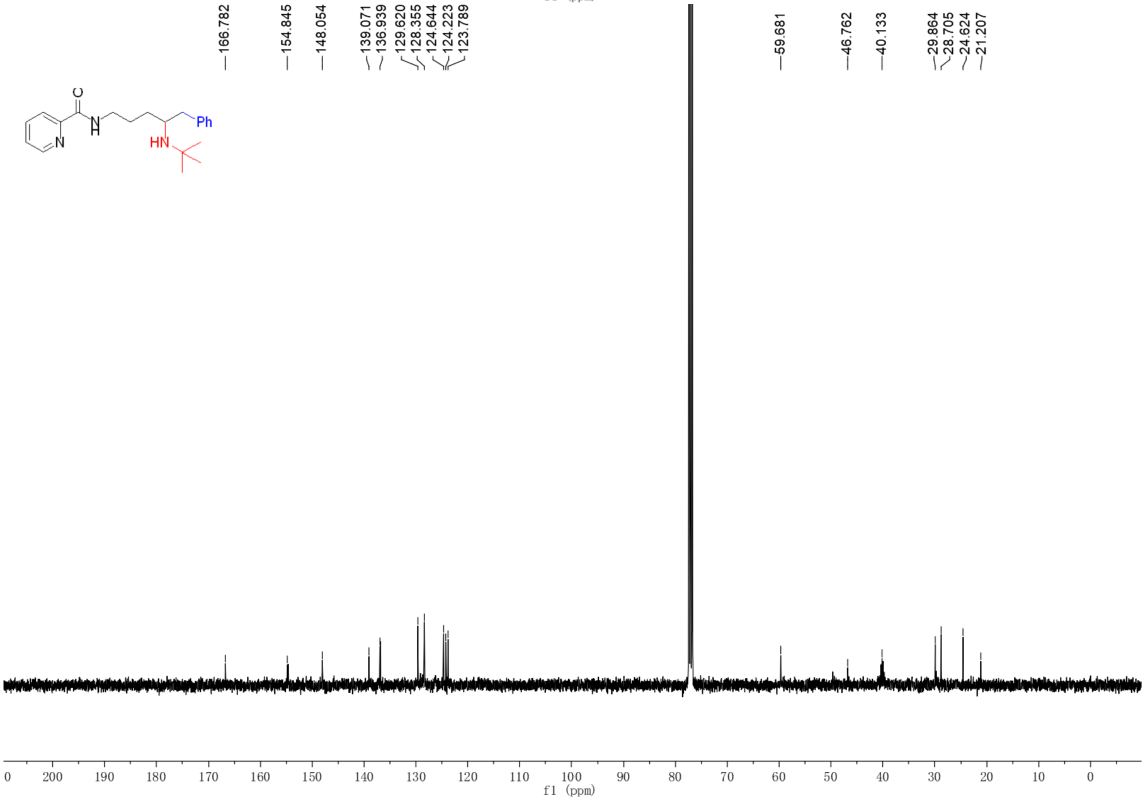

Supplementary Figure 64. ^{1}H NMR and ^{13}C NMR spectra of **4o**.

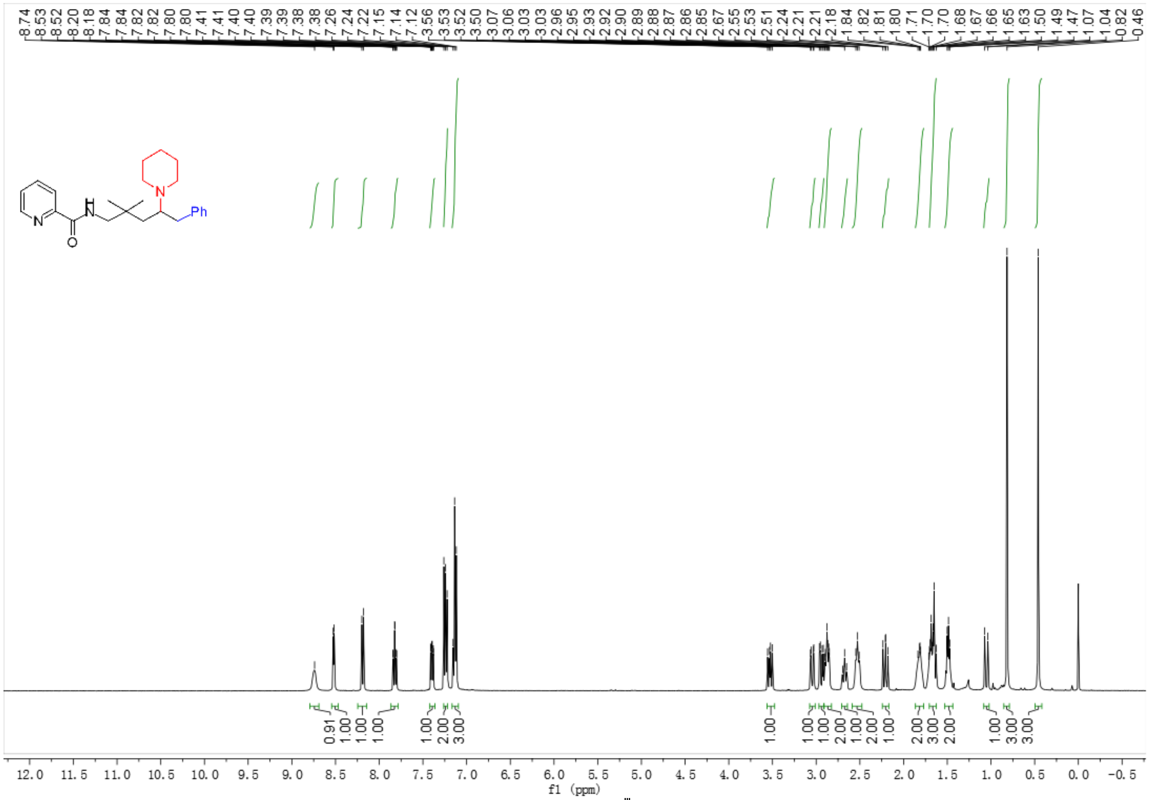

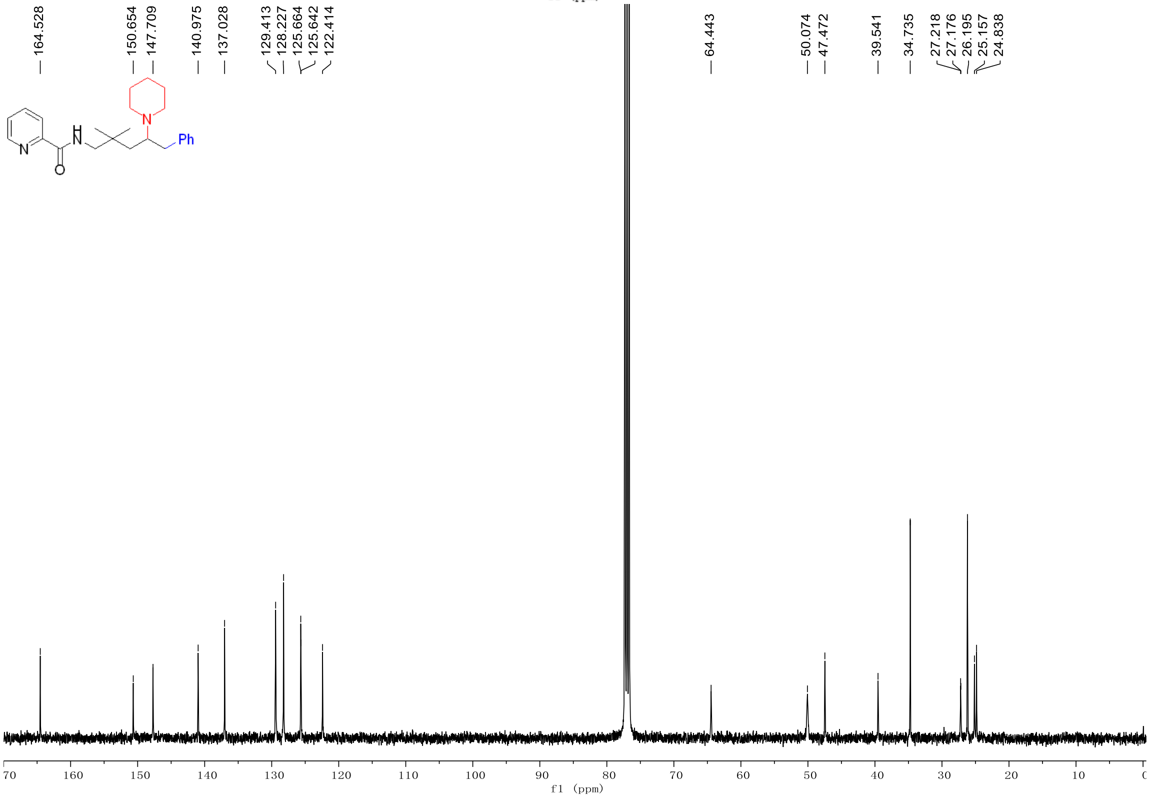

Supplementary Figure 65. ^{1}H NMR and ^{13}C NMR spectra of **4p**.

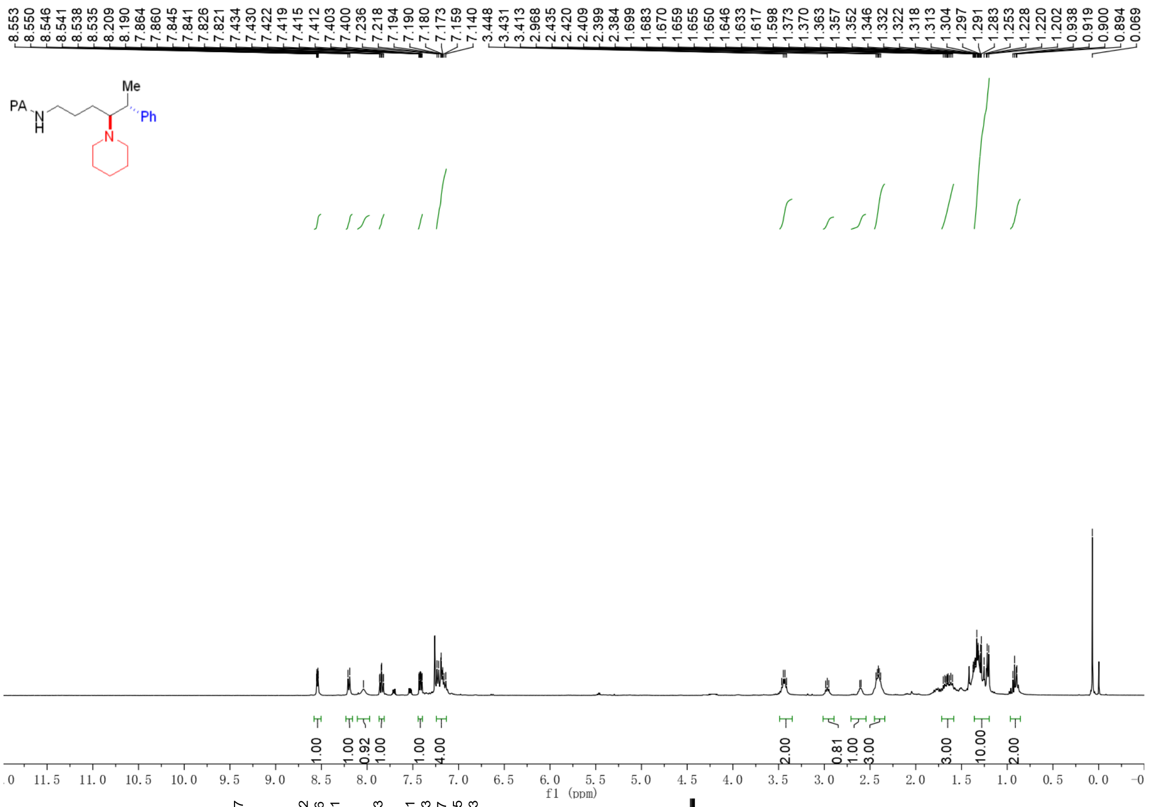

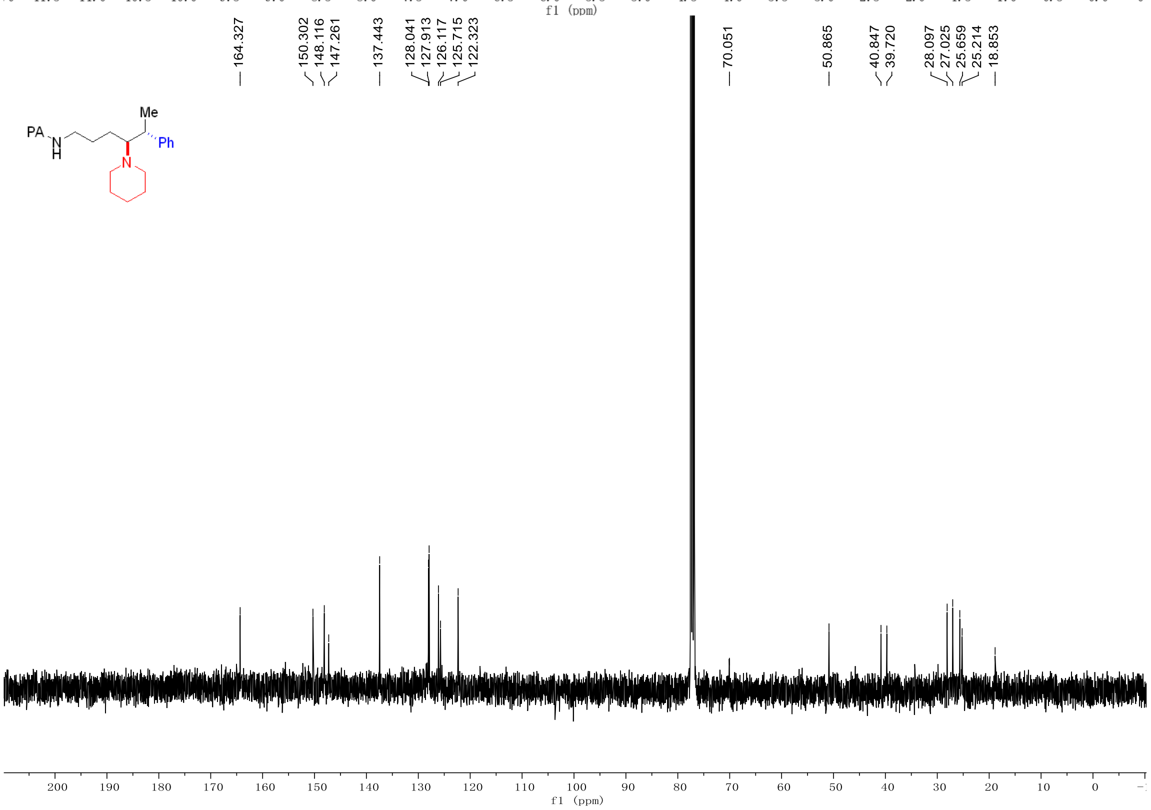

Supplementary Figure 66. ^{1}H NMR and ^{13}C NMR spectra of **4q**.

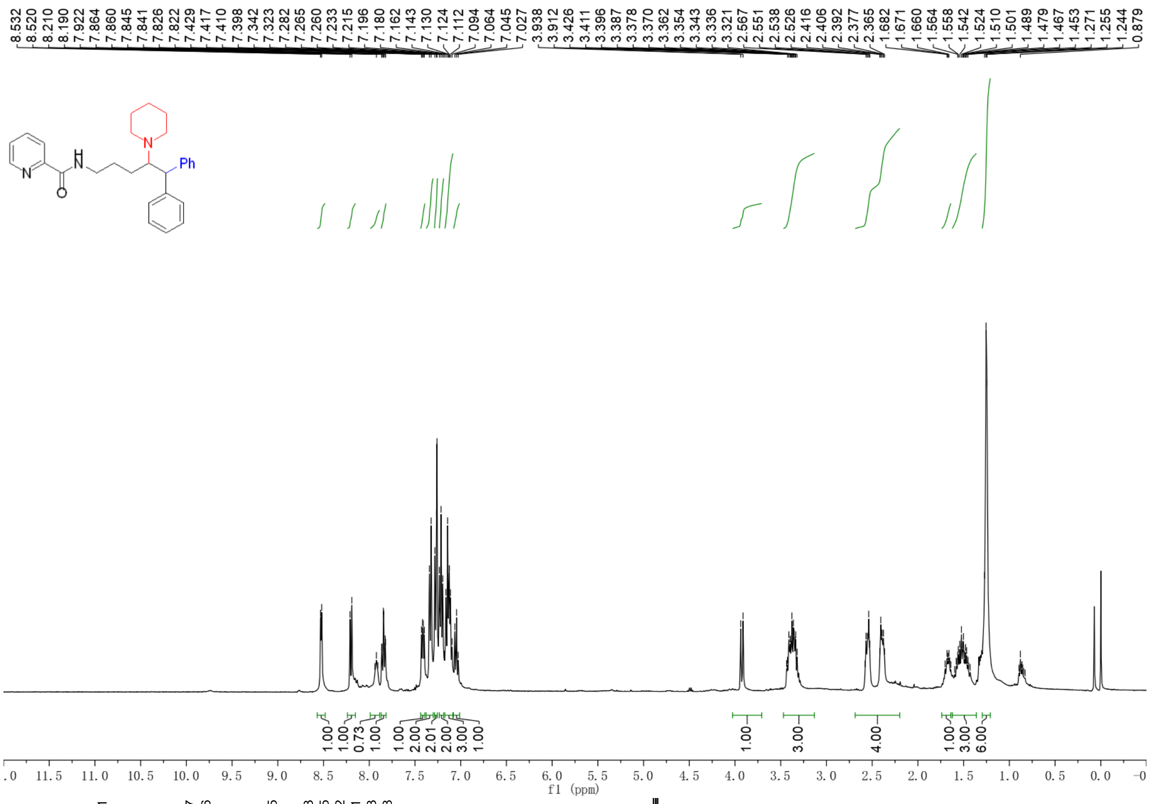

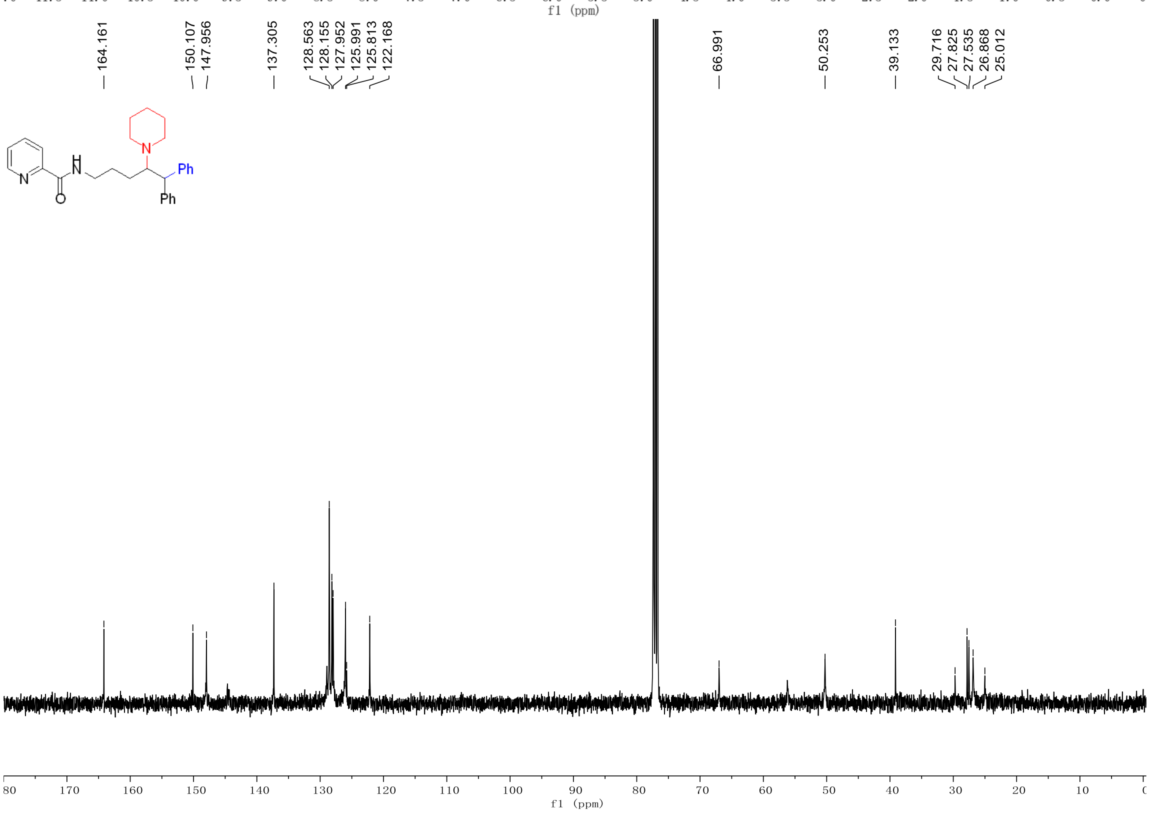

Supplementary Figure 67. ^{1}H NMR and ^{13}C NMR spectra of **4r**.

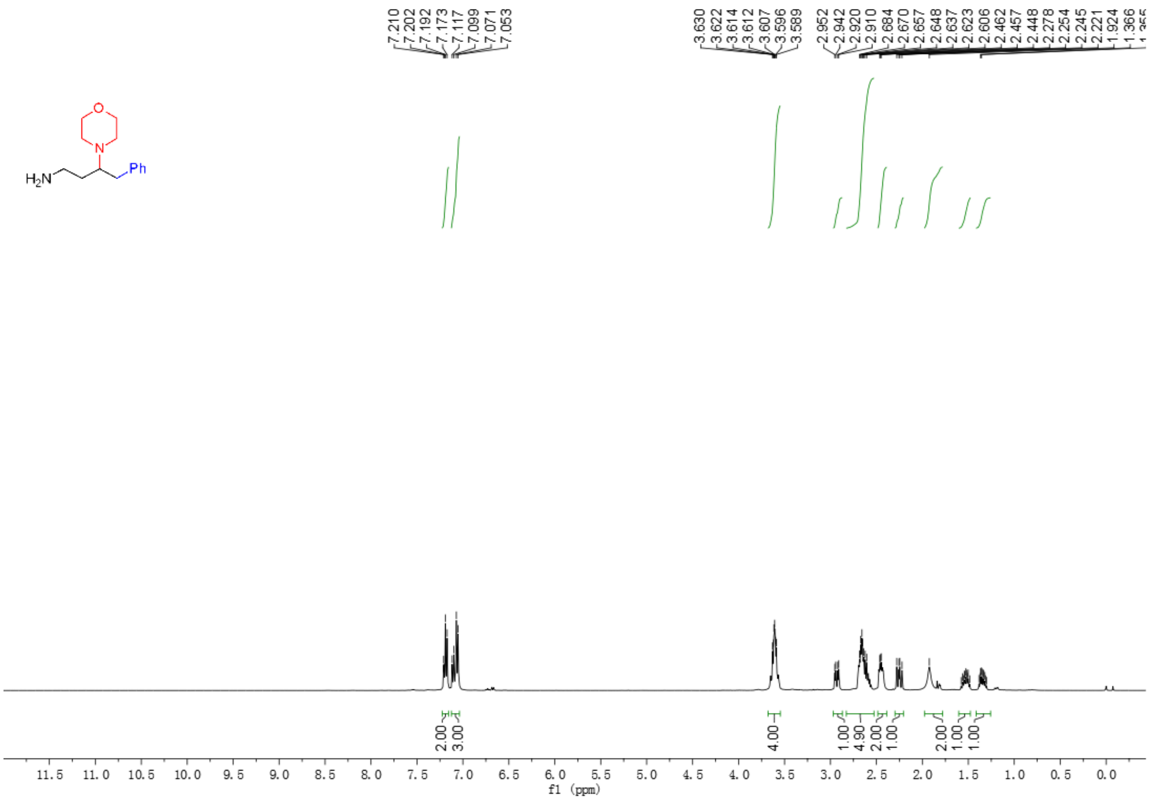

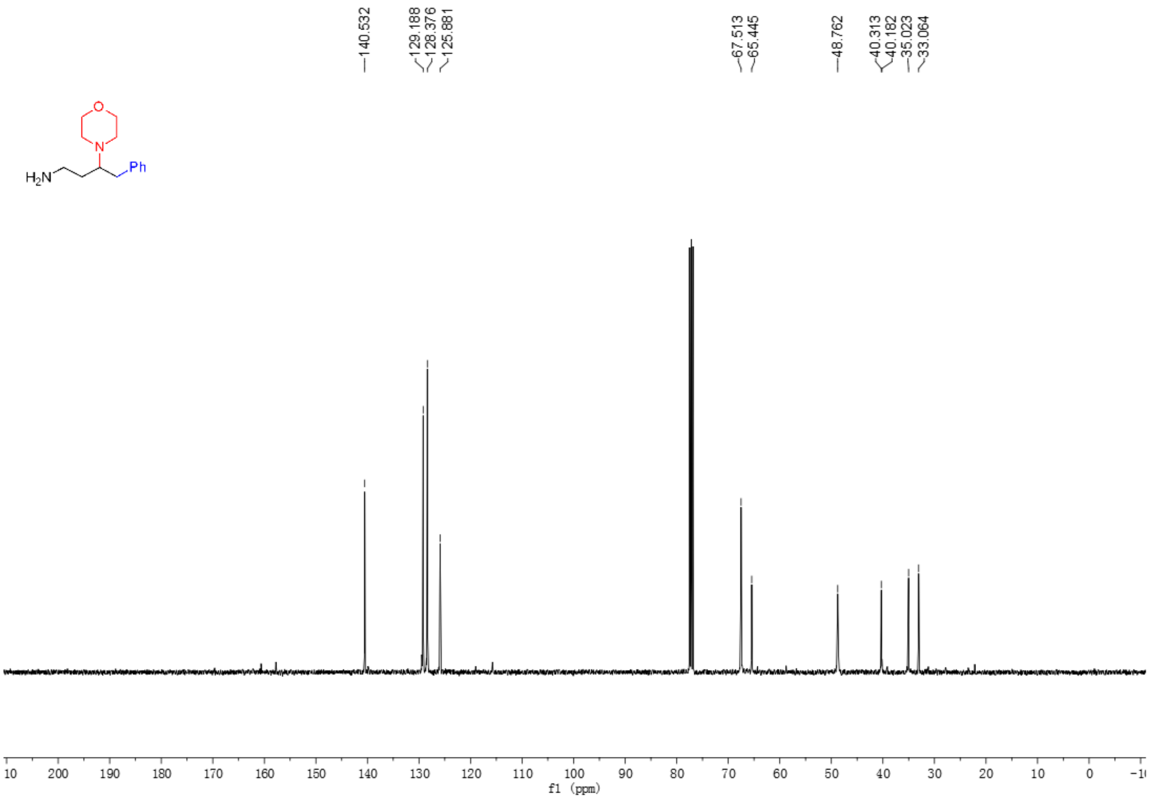

Supplementary Figure 68. ^{1}H NMR and ^{13}C NMR spectra of **5**.

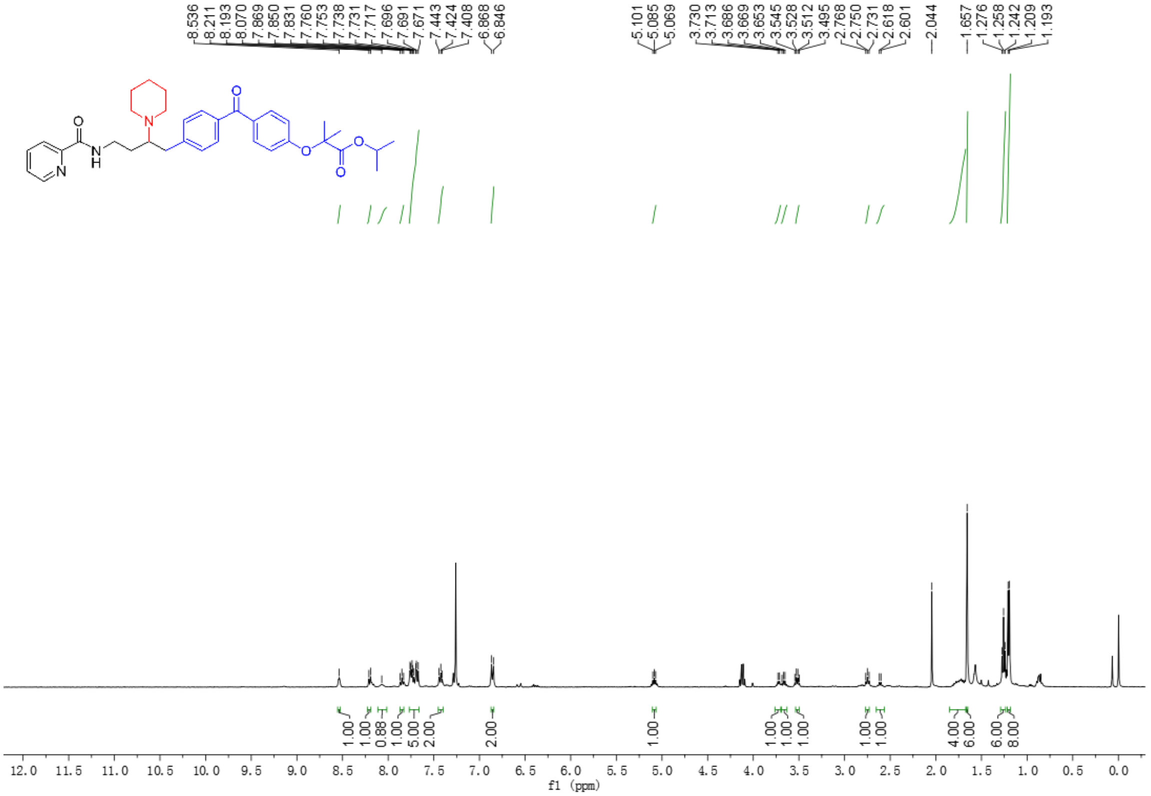

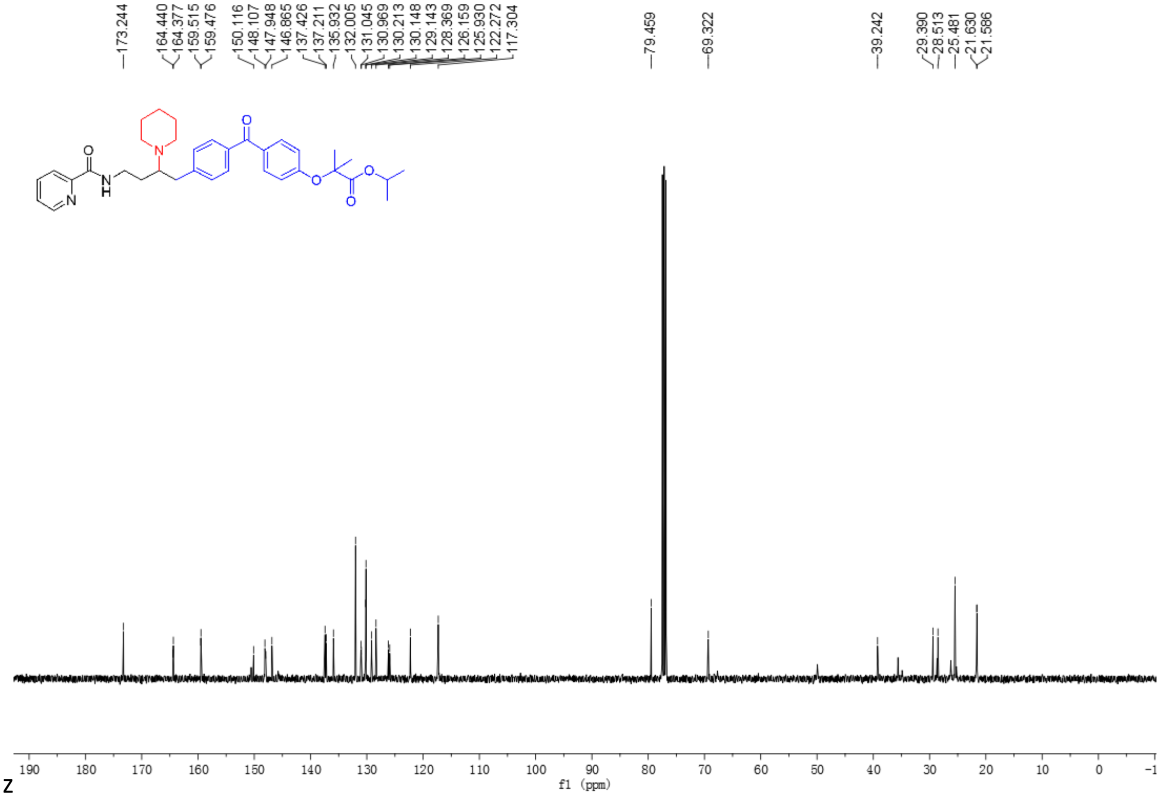

Supplementary Figure 69. ^{1}H NMR and ^{13}C NMR spectra of **6a**.

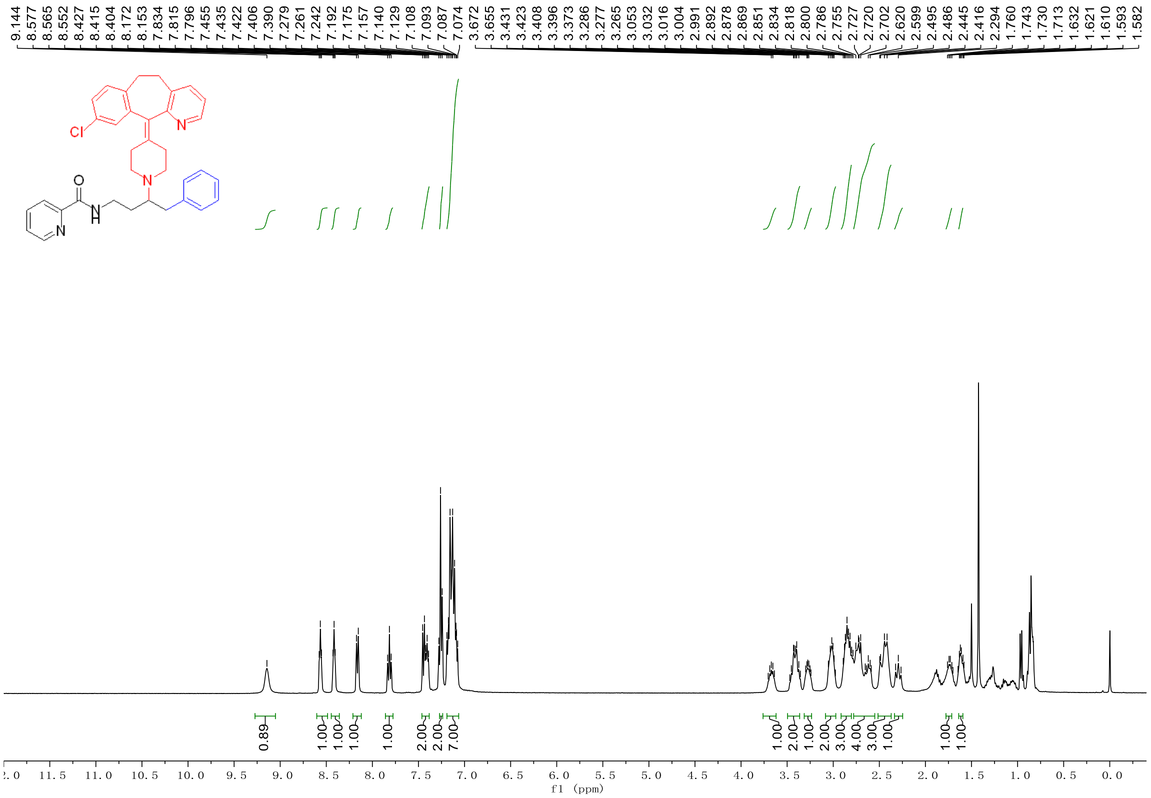

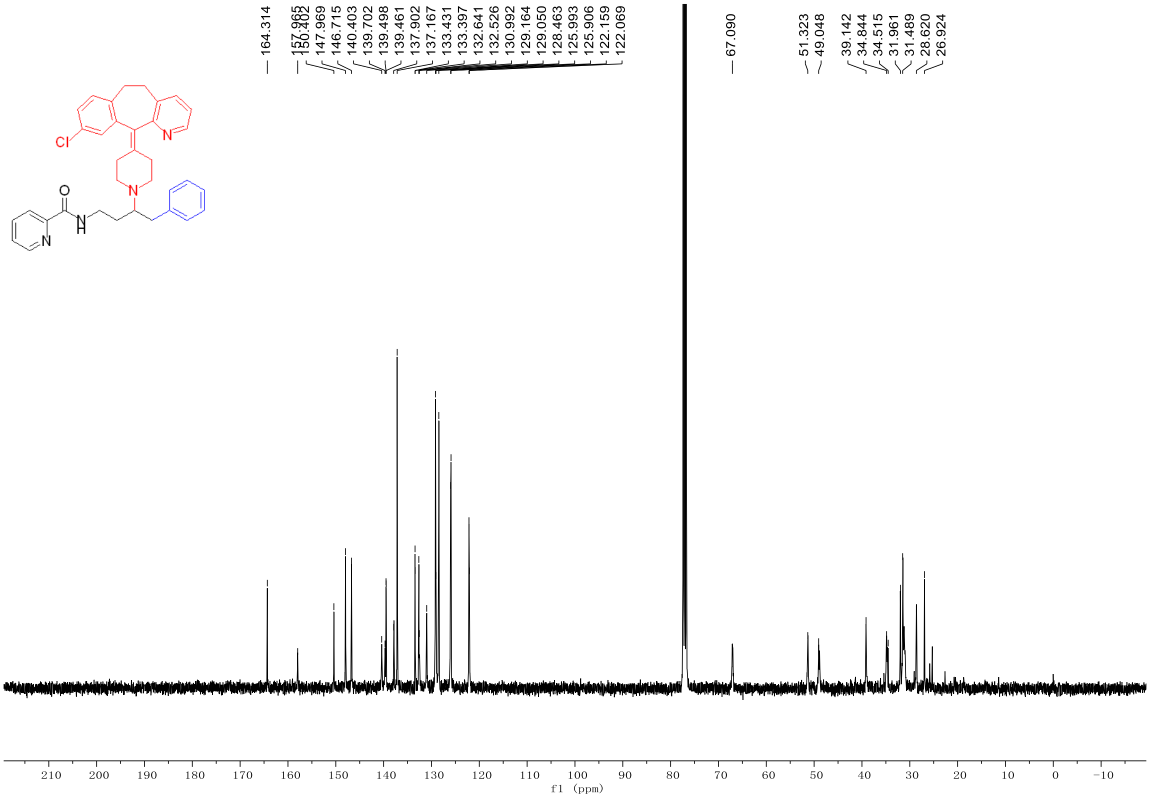

Supplementary Figure 70. ^{1}H NMR and ^{13}C NMR spectra of **6b**.

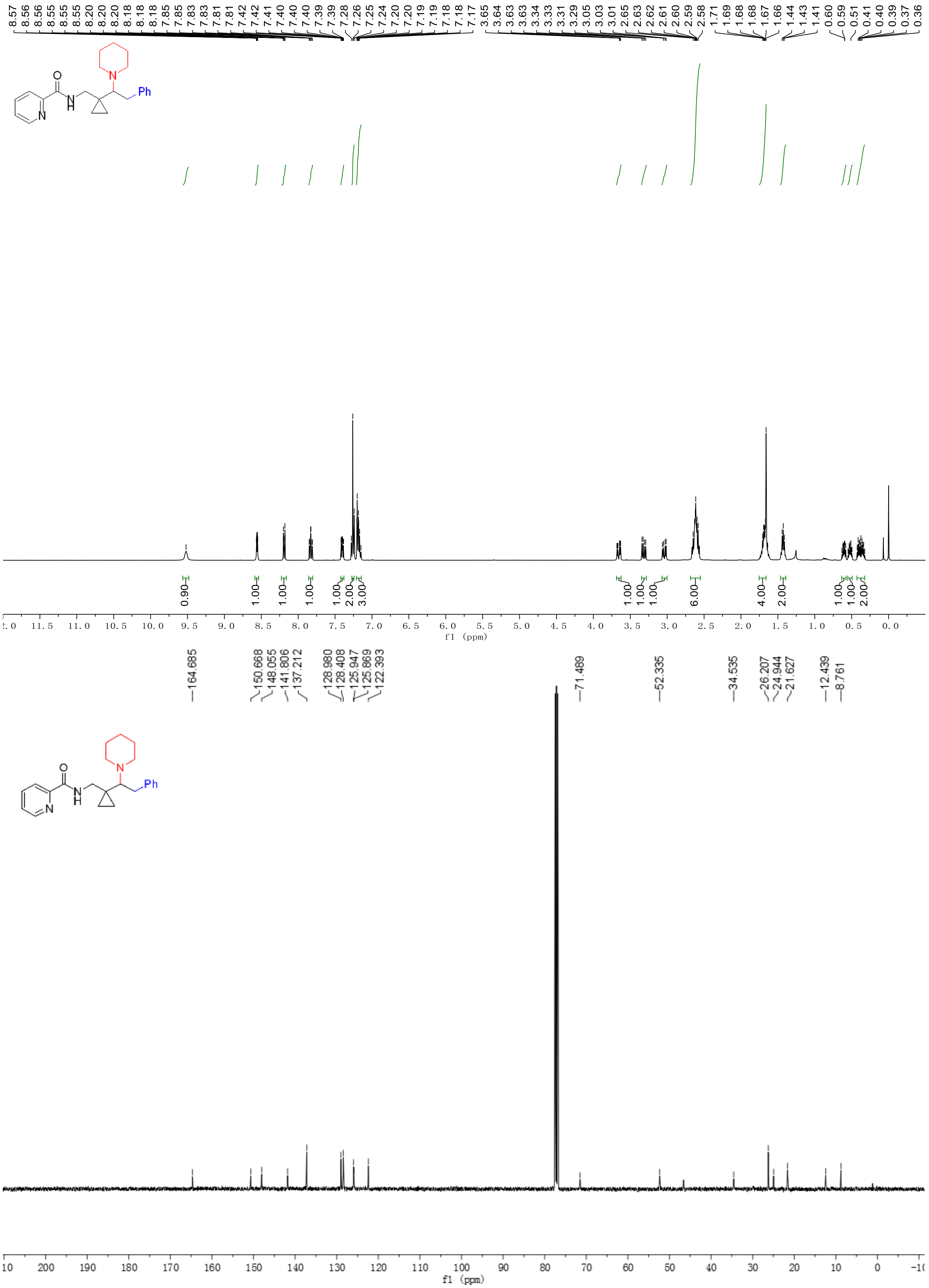

Supplementary Figure 71. ^{1}H NMR and ^{13}C NMR spectra of **4s**.

II. Supplementary References

[1] J. A. Gurak, K. S. Yang, Z. Liu, K. M. Engle, *J. Am. Chem. Soc.* **2016**, *138*, 5805.

[2] Y.-C. Luo, C. Yang, S.-Q, Qiu, Q.-J. Liang, Y.-H. X, T.-P. Loh, *ACS Catal*. **2019**, *9*, 4271.

[3] Z. Liu, T. Zeng, K. S. Yang, K. M. Engle, *J. Am. Chem. Soc.* **2016**, *138,* 15122.

[4] M. Feuerstein, H. Doucet, M. Santelli, *Tetrahedron letters*, **2001**, *42*, 5659.

[5] B. S. Schreib, M. Fadel , E. M. Carreira, *Angew. Chem. Int. Ed.* **2020**, *59*, 7818.

[6] J. Derosa, V. T. Tran, M. N. Boulous, J. S. Chen, K. M. Engle, *J. Am. Chem. Soc.* **2017**, *139*, 10657.
