# Supplementary material for: Directed nickel-catalyzed regio- and diastereoselective arylamination of unactivated alkenes
Source: Nat Commun. 2021 Nov 1;12:6280. doi: 10.1038/s41467-021-26527-x (PMC8560905; doi:10.1038/s41467-021-26527-x)

checkCIF/PLATON report

Structure factors have been supplied for datablock(s) a201210b

THIS REPORT IS FOR GUIDANCE ONLY. IF USED AS PART OF A REVIEW PROCEDURE FOR PUBLICATION, IT SHOULD NOT REPLACE THE EXPERTISE OF AN EXPERIENCED CRYSTALLOGRAPHIC REFEREE.

No syntax errors found. CIF dictionary Interpreting this report

Datablock: a201210b

```
Bond precision:  C-C = 0.0019 A          Wavelength=1.54184

Cell:          a=11.5615(3)      b=12.3247(3)     c=18.0802(4)
               alpha=76.633(2)   beta=74.160(2)   gamma=68.705(2)
Temperature:   150 K

                Calculated                 Reported
Volume          2284.04(10)                2284.03(10)
Space group     P -1                       P -1
Hall group      -P 1                       -P 1
Moiety formula  C27 H31 N3 O               C27 H31 N3 O
Sum formula     C27 H31 N3 O               C27 H31 N3 O
Mr              413.55                     413.55
Dx,g cm-3       1.203                      1.203
Z               4                          4
Mu (mm-1)       0.573                      0.573
F000            888.0                      888.0
F000'           890.37
h,k,lmax        13,14,21                   13,14,21
Nref            8171                       8156
Tmin,Tmax       0.872,0.892                0.215,1.000
Tmin'           0.872

Correction method= # Reported T Limits: Tmin=0.215 Tmax=1.000
AbsCorr = MULTI-SCAN

Data completeness= 0.998          Theta(max)= 67.066

R(reflections)= 0.0411( 7402)     wR2(reflections)= 0.1155( 8156)

S = 1.038                  Npar= 560
```

The following ALERTS were generated. Each ALERT has the format
test-name_ALERT_alert-type_alert-level.
Click on the hyperlinks for more details of the test.

Alert level G

```
PLAT005_ALERT_5_G No Embedded Refinement Details Found  in the CIF     Please Do !
PLAT007_ALERT_5_G Number of Unrefined Donor-H Atoms ..............          2 Report
PLAT154_ALERT_1_G The s.u.'s on the Cell Angles are Equal ..(Note)      0.002 Degree
PLAT793_ALERT_4_G Model has Chirality at C7          (Centro SPGR)          R Verify
PLAT793_ALERT_4_G Model has Chirality at C15         (Centro SPGR)          S Verify
PLAT793_ALERT_4_G Model has Chirality at C34         (Centro SPGR)          S Verify
PLAT793_ALERT_4_G Model has Chirality at C42         (Centro SPGR)          R Verify
PLAT883_ALERT_1_G No Info/Value for _atom_sites_solution_primary .     Please Do !
```

```
   0 ALERT level A = Most likely a serious problem - resolve or explain
   0 ALERT level B = A potentially serious problem, consider carefully
   0 ALERT level C = Check. Ensure it is not caused by an omission or oversight
   8 ALERT level G = General information/check it is not something unexpected

   2 ALERT type 1 CIF construction/syntax error, inconsistent or missing data
   0 ALERT type 2 Indicator that the structure model may be wrong or deficient
   0 ALERT type 3 Indicator that the structure quality may be low
   4 ALERT type 4 Improvement, methodology, query or suggestion
   2 ALERT type 5 Informative message, check
```

It is advisable to attempt to resolve as many as possible of the alerts in all categories. Often the minor alerts point to easily fixed oversights, errors and omissions in your CIF or refinement strategy, so attention to these fine details can be worthwhile. In order to resolve some of the more serious problems it may be necessary to carry out additional measurements or structure refinements. However, the purpose of your study may justify the reported deviations and the more serious of these should normally be commented upon in the discussion or experimental section of a paper or in the "special_details" fields of the CIF. checkCIF was carefully designed to identify outliers and unusual parameters, but every test has its limitations and alerts that are not important in a particular case may appear. Conversely, the absence of alerts does not guarantee there are no aspects of the results needing attention. It is up to the individual to critically assess their own results and, if necessary, seek expert advice.

Publication of your CIF in IUCr journals

A basic structural check has been run on your CIF. These basic checks will be run on all CIFs submitted for publication in IUCr journals (*Acta Crystallographica*, *Journal of Applied Crystallography*, *Journal of Synchrotron Radiation*); however, if you intend to submit to *Acta Crystallographica Section C* or *E* or *IUCrData*, you should make sure that full publication checks are run on the final version of your CIF prior to submission.

Publication of your CIF in other journals

Please refer to the *Notes for Authors* of the relevant journal for any special instructions relating to CIF submission.

PLATON version of 05/12/2020; check.def file version of 05/12/2020

Datablock a201210b - ellipsoid plot

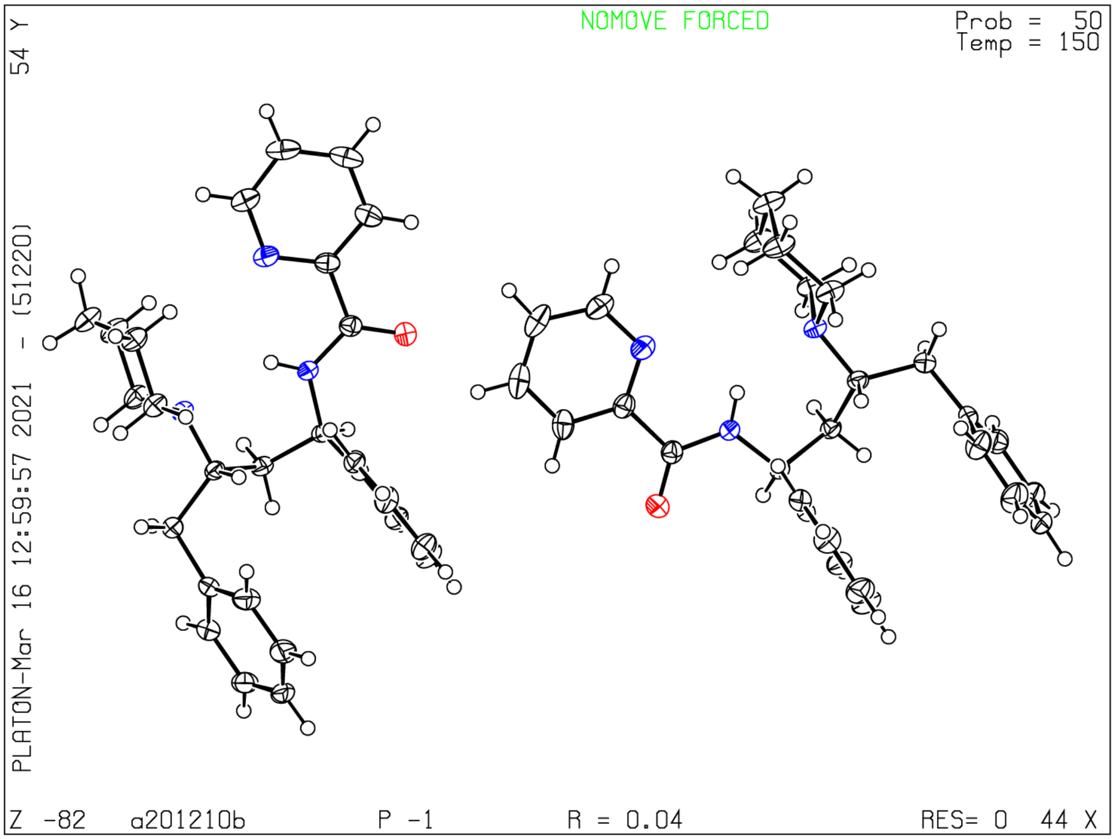

Supplement: Supplementary file 2 — Source Data [file 41467_2021_26527_MOESM2_ESM.zip › 4b.checkcif.pdf]
