# Supplementary material for: Directed nickel-catalyzed regio- and diastereoselective arylamination of unactivated alkenes
Source: Nat Commun. 2021 Nov 1;12:6280. doi: 10.1038/s41467-021-26527-x (PMC8560905; doi:10.1038/s41467-021-26527-x)

checkCIF/PLATON report

Structure factors have been supplied for datablock(s) a210105b

THIS REPORT IS FOR GUIDANCE ONLY. IF USED AS PART OF A REVIEW PROCEDURE FOR PUBLICATION, IT SHOULD NOT REPLACE THE EXPERTISE OF AN EXPERIENCED CRYSTALLOGRAPHIC REFEREE.

No syntax errors found. CIF dictionary Interpreting this report

Datablock: a210105b

```
Bond precision:  C-C = 0.0062 A          Wavelength=1.54184

Cell:           a=21.748(3)    b=5.4836(6)         c=19.4942(13)
                alpha=90       beta=99.738(10)     gamma=90
Temperature:    293 K

                Calculated                 Reported
Volume          2291.3(4)                  2291.3(4)
Space group     P 21/c                     P 1 21/c 1
Hall group      -P 2ybc                    -P 2ybc
Moiety formula  C25 H33 N3 O2              C25 H33 N3 O2
Sum formula     C25 H33 N3 O2              C25 H33 N3 O2
Mr              407.54                     407.54
Dx,g cm-3       1.181                      1.181
Z               4                          4
Mu (mm-1)       0.593                      0.593
F000            880.0                      880.0
F000'           882.42
h,k,lmax        25,6,23                    25,6,23
Nref            4105                       4044
Tmin,Tmax       0.899,0.920                0.692,1.000
Tmin'           0.888

Correction method= # Reported T Limits: Tmin=0.692 Tmax=1.000
AbsCorr = MULTI-SCAN

Data completeness= 0.985          Theta(max)= 67.061

R(reflections)= 0.0787( 2299)     wR2(reflections)= 0.2353( 4044)

S = 1.072                   Npar= 273
```

The following ALERTS were generated. Each ALERT has the format
test-name_ALERT_alert-type_alert-level.
Click on the hyperlinks for more details of the test.

Alert level C

```
PLAT241_ALERT_2_C High   'MainMol' Ueq as Compared to Neighbors of         C5 Check
PLAT241_ALERT_2_C High   'MainMol' Ueq as Compared to Neighbors of         C7 Check
PLAT242_ALERT_2_C Low    'MainMol' Ueq as Compared to Neighbors of         C1 Check
PLAT340_ALERT_3_C Low Bond Precision on  C-C Bonds ...............    0.00622 Ang.
```

Alert level G

```
PLAT007_ALERT_5_G Number of Unrefined Donor-H Atoms ..............          1 Report
PLAT012_ALERT_1_G No       _shelx_res_checksum Found in CIF ......     Please Check
PLAT199_ALERT_1_G Reported _cell_measurement_temperature ..... (K)        293 Check
PLAT200_ALERT_1_G Reported   _diffrn_ambient_temperature ..... (K)        293 Check
PLAT793_ALERT_4_G Model has Chirality at C9          (Centro SPGR)          S Verify
PLAT793_ALERT_4_G Model has Chirality at C15         (Centro SPGR)          S Verify
PLAT883_ALERT_1_G No Info/Value for _atom_sites_solution_primary .     Please Do !
PLAT933_ALERT_2_G Number of OMIT Records in Embedded .res File ...          5 Note
PLAT941_ALERT_3_G Average HKL Measurement Multiplicity ...........        1.9 Low
```

```
   0 ALERT level A = Most likely a serious problem - resolve or explain
   0 ALERT level B = A potentially serious problem, consider carefully
   4 ALERT level C = Check. Ensure it is not caused by an omission or oversight
   9 ALERT level G = General information/check it is not something unexpected

   4 ALERT type 1 CIF construction/syntax error, inconsistent or missing data
   4 ALERT type 2 Indicator that the structure model may be wrong or deficient
   2 ALERT type 3 Indicator that the structure quality may be low
   2 ALERT type 4 Improvement, methodology, query or suggestion
   1 ALERT type 5 Informative message, check
```

It is advisable to attempt to resolve as many as possible of the alerts in all categories. Often the minor alerts point to easily fixed oversights, errors and omissions in your CIF or refinement strategy, so attention to these fine details can be worthwhile. In order to resolve some of the more serious problems it may be necessary to carry out additional measurements or structure refinements. However, the purpose of your study may justify the reported deviations and the more serious of these should normally be commented upon in the discussion or experimental section of a paper or in the "special_details" fields of the CIF. checkCIF was carefully designed to identify outliers and unusual parameters, but every test has its limitations and alerts that are not important in a particular case may appear. Conversely, the absence of alerts does not guarantee there are no aspects of the results needing attention. It is up to the individual to critically assess their own results and, if necessary, seek expert advice.

Publication of your CIF in IUCr journals

A basic structural check has been run on your CIF. These basic checks will be run on all CIFs submitted for publication in IUCr journals (*Acta Crystallographica*, *Journal of Applied Crystallography*, *Journal of Synchrotron Radiation*); however, if you intend to submit to *Acta Crystallographica Section C* or *E* or *IUCrData*, you should make sure that full publication checks are run on the final version of your CIF prior to submission.

Publication of your CIF in other journals

Please refer to the *Notes for Authors* of the relevant journal for any special instructions relating to CIF submission.

PLATON version of 05/12/2020; check.def file version of 05/12/2020

Datablock a210105b - ellipsoid plot

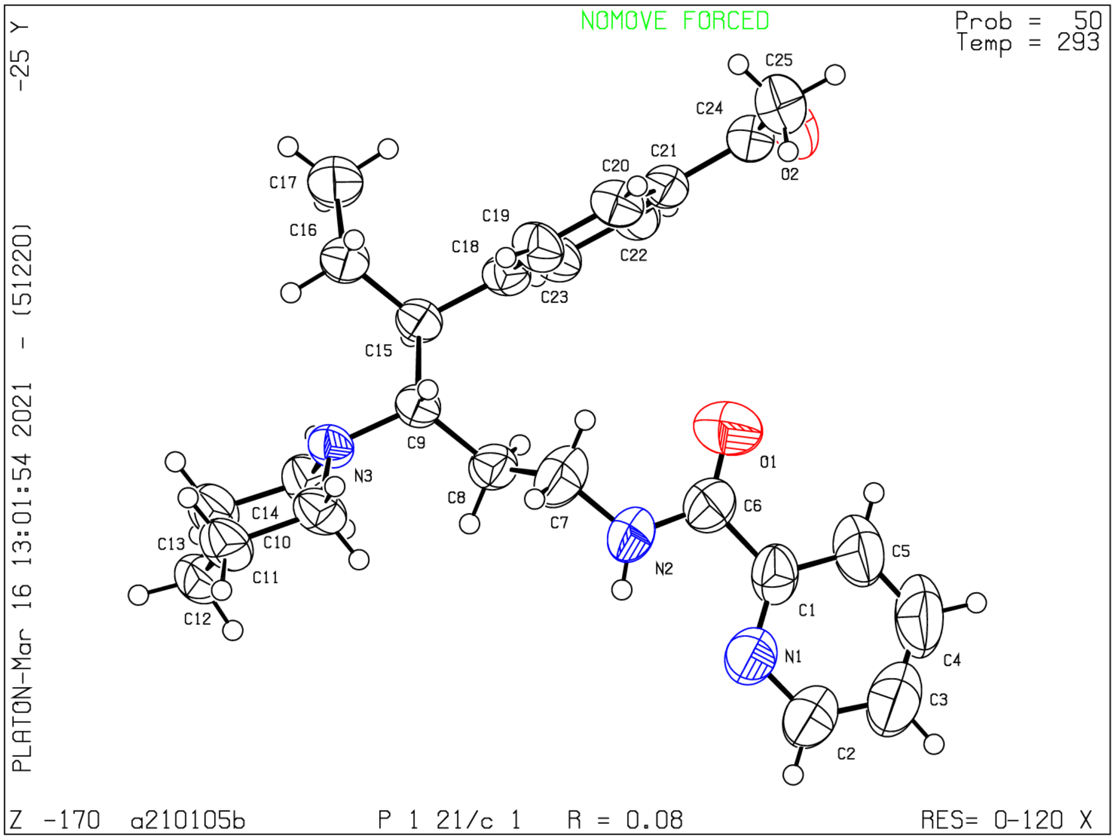

Supplement: Supplementary file 2 — Source Data [file 41467_2021_26527_MOESM2_ESM.zip › 4i.checkcif.pdf]
