# Supplementary material for: Directed nickel-catalyzed regio- and diastereoselective arylamination of unactivated alkenes
Source: Nat Commun. 2021 Nov 1;12:6280. doi: 10.1038/s41467-021-26527-x (PMC8560905; doi:10.1038/s41467-021-26527-x)
Supplement: Supplementary file 2 — Source Data [file 41467_2021_26527_MOESM2_ESM.zip › X-ray Crystallographic Data.pdf]

Supplementary Table 1. Crystal data and structure refinement for **4b**.

Identification code	4b
Empirical formula	$C_{27}H_{31}N_3O$
Formula weight	413.55
Temperature/K	150.00(10)
Crystal system	triclinic
Space group	P-1
a/Å	11.5615(3)
b/Å	12.3247(3)
c/Å	18.0802(4)
α/°	76.633(2)
β/°	74.160(2)
γ/°	68.705(2)
Volume/Å^3	2284.03(10)
Z	4
ρ_{calc}g/cm^3	1.203
μ/mm^{-1}	0.573
F(000)	888.0
Crystal size/mm^3	0.24 × 0.23 × 0.2
Radiation	CuKα (λ = 1.54184)
2Θ range for data collection/°	5.136 to 134.132
Index ranges	-13 ≤ h ≤ 13, -14 ≤ k ≤ 14, -21 ≤ l ≤ 21
Reflections collected	34752
Independent reflections	8156 [R_{int} = 0.0478, Rsigma = 0.0303]
Data/restraints/parameters	8156/0/560
Goodness-of-fit on F^2	1.038
Final R indexes [I>=2σ (I)]	R_1 = 0.0411, wR_2 = 0.1105
Final R indexes [all data]	R_1 = 0.0449, wR_2 = 0.1155

Largest diff. peak/hole / e Å^{-3}	0.25/-0.23

Supplementary Table 2. Fractional Atomic Coordinates (×10^4) and Equivalent Isotropic Displacement Parameters (Å^2×10^3) for **4b**. U_{eq} is defined as 1/3 of the trace of the orthogonalised U_{IJ} tensor.

Atom	x	y	z	U(eq)
O(1)	3140.6(8)	7328.6(8)	3523.3(5)	29.8(2)
N(1)	3070.6(10)	9420.1(9)	4604.0(6)	30.2(2)
N(2)	2128.6(9)	7661.4(8)	4754.2(6)	22.3(2)
N(3)	990.2(9)	8118.0(8)	6332.7(6)	20.4(2)
C(1)	3499.7(11)	8783.8(10)	4020.8(7)	23.7(3)
C(2)	3573.6(14)	10264.7(12)	4545.1(9)	37.6(3)
C(3)	4495.5(14)	10505.4(13)	3924.3(9)	40.3(3)
C(4)	4946.7(13)	9830.5(13)	3334.8(8)	37.7(3)
C(5)	4442.5(12)	8946.5(12)	3384.4(7)	29.8(3)
C(6)	2907.0(11)	7854.8(10)	4075.2(7)	22.4(2)
C(7)	1417.8(11)	6847.1(10)	4877.9(7)	20.7(2)
C(8)	2223(1)	5556(1)	4994.3(6)	20.4(2)
C(9)	1838.3(11)	4729.3(11)	4801.4(7)	25.2(3)
C(10)	2504.3(13)	3538.7(11)	4925.9(8)	31.7(3)
C(11)	3563.7(13)	3150.4(11)	5252.3(8)	31.6(3)
C(12)	3954.3(12)	3963.1(11)	5449.8(7)	28.8(3)
C(13)	3292.1(11)	5160.1(11)	5316.1(7)	24.4(3)
C(14)	291.1(10)	7132.1(10)	5568.7(7)	21.2(2)
C(15)	673.3(10)	7064.7(10)	6332.1(7)	19.8(2)
C(16)	-110.0(11)	9186.9(10)	6454.5(8)	26.3(3)
C(17)	335.2(13)	10252.2(11)	6299.7(8)	32.3(3)
C(18)	1231.8(13)	10087.8(11)	6830.5(8)	33.4(3)

C(19)	2310.7(12)	8928.7(11)	6754.3(8)	29.1(3)
C(20)	1791.6(11)	7913.5(10)	6888.0(7)	25.2(3)
C(21)	-300.5(11)	6747.8(10)	7048.4(7)	25.0(3)
C(22)	-364.9(11)	5537.1(10)	7076.1(7)	23.6(3)
C(23)	-1500.0(12)	5377.6(11)	7088.5(7)	26.7(3)
C(24)	-1544.6(13)	4275.3(12)	7063.9(7)	31.0(3)
C(25)	-450.8(14)	3315.9(11)	7022.6(7)	31.2(3)
C(26)	683.0(13)	3455.8(11)	7021.7(8)	33.9(3)
C(27)	724.4(12)	4554.3(11)	7053.7(8)	30.8(3)
O(2)	4502.9(9)	2304.6(8)	2227.4(5)	31.7(2)
N(4)	6300.7(12)	4271.9(10)	1628.4(7)	35.5(3)
N(5)	5890.1(9)	2597.8(8)	1102.1(6)	22.5(2)
N(6)	7553.9(9)	3157.3(8)	-333.1(6)	20.6(2)
C(28)	5501.3(11)	3735.9(10)	2113.2(7)	25.0(3)
C(29)	6478.9(16)	5121.9(13)	1884.4(9)	44.2(4)
C(30)	5882.4(15)	5471.8(13)	2602.8(10)	43.2(4)
C(31)	5076.7(13)	4902.1(13)	3097.7(9)	38.1(3)
C(32)	4890.0(12)	4008.2(12)	2852.8(8)	30.7(3)
C(33)	5256.7(11)	2807.6(10)	1821.1(7)	23.2(3)
C(34)	5680.9(11)	1775.4(10)	730.6(7)	20.9(2)
C(35)	6394.9(11)	494.1(10)	990.6(6)	21.1(2)
C(36)	7461.8(11)	147.9(11)	1309.6(7)	25.1(3)
C(37)	8099.9(12)	-1038.7(12)	1509.1(8)	32.0(3)
C(38)	7683.0(13)	-1890.5(12)	1394.0(8)	35.8(3)
C(39)	6619.5(14)	-1555.4(12)	1077.1(9)	35.9(3)
C(40)	5983.4(12)	-374.9(11)	879.2(8)	28.4(3)
C(41)	6023.7(11)	2091.7(10)	-158.6(7)	21.0(2)
C(42)	7395.2(10)	2095.3(10)	-479.3(6)	20.5(2)

C(43)	6966.6(12)	4253.7(10)	-816.8(7)	25.6(3)
C(44)	7016.2(12)	5307.3(11)	-537.4(8)	31.8(3)
C(45)	8376.3(13)	5201.9(11)	-558.1(9)	34.9(3)
C(46)	9016.2(13)	4022.2(11)	-108.4(9)	33.7(3)
C(47)	8892.4(11)	3019.5(11)	-403.8(8)	28.6(3)
C(48)	7862.5(12)	1857.9(10)	-1333.4(7)	24.8(3)
C(49)	7932.5(11)	637.8(10)	-1399.2(7)	23.3(2)
C(50)	8734.6(13)	-355.0(12)	-1029.3(8)	33.8(3)
C(51)	8718.9(15)	-1471.3(12)	-1038.1(8)	39.3(3)
C(52)	7916.3(13)	-1618.3(11)	-1422.8(7)	32.0(3)
C(53)	7148.5(12)	-648.0(11)	-1811.7(8)	30.4(3)
C(54)	7161.4(12)	470.7(11)	-1800.5(7)	26.9(3)

Supplementary Table 3. Anisotropic Displacement Parameters (Å^2×10^3) for **4b**. The Anisotropic displacement factor exponent takes the form: -2π^2[h^{2}a*^{2}U$_{11}$+2hka*b*U$_{12}$+…].

Atom	U_{11}	U_{22}	U_{33}	U_{23}	U_{13}	U_{12}
O(1)	34.7(5)	28.8(5)	25.8(4)	-7.0(4)	-0.1(4)	-12.9(4)
N(1)	33.0(6)	25.1(5)	34.9(6)	-5.0(4)	-2.8(5)	-14.9(5)
N(2)	27.1(5)	20.9(5)	22.1(5)	-4.7(4)	-2.7(4)	-12.2(4)
N(3)	19.7(5)	16.7(5)	25.9(5)	-4.0(4)	-5.3(4)	-6.1(4)
C(1)	23.2(6)	20.7(6)	26.0(6)	2.6(5)	-8.0(5)	-7.4(5)
C(2)	44.8(8)	30.1(7)	44.6(8)	-6.9(6)	-6.8(6)	-20.6(6)
C(3)	44.5(8)	35.0(7)	49.1(9)	5.7(6)	-13.1(7)	-26.6(7)
C(4)	36.5(7)	43.3(8)	35.2(7)	10.4(6)	-7.7(6)	-24.5(6)
C(5)	28.8(6)	33.4(7)	26.4(6)	3.1(5)	-6.4(5)	-13.0(5)
C(6)	22.3(6)	19.1(6)	24.1(6)	-0.8(5)	-5.8(5)	-5.4(4)
C(7)	24.2(6)	19.4(6)	21.7(6)	-2.1(4)	-6.0(4)	-10.6(5)
C(8)	22.4(6)	21.3(6)	17.6(5)	-4.6(4)	-0.1(4)	-9.1(5)

C(9)	26.5(6)	24.9(6)	27.4(6)	-4.7(5)	-8.0(5)	-9.8(5)
C(10)	38.4(7)	23.0(6)	39.7(7)	-9.0(5)	-12.2(6)	-11.2(5)
C(11)	33.4(7)	20.1(6)	39.1(7)	-6.6(5)	-10.3(6)	-2.7(5)
C(12)	25.3(6)	28.5(7)	32.5(7)	-7.8(5)	-8.7(5)	-4.5(5)
C(13)	24.3(6)	24.5(6)	26.4(6)	-7.4(5)	-3.6(5)	-9.3(5)
C(14)	20.3(5)	18.4(5)	27.3(6)	-3.7(4)	-5.9(5)	-7.9(4)
C(15)	19.2(5)	15.9(5)	24.1(6)	-4.9(4)	-2.0(4)	-5.9(4)
C(16)	23.4(6)	19.5(6)	36.6(7)	-8.0(5)	-7.6(5)	-4.1(5)
C(17)	36.0(7)	17.7(6)	44.3(8)	-4.8(5)	-13.4(6)	-6.1(5)
C(18)	41.0(7)	23.8(6)	43.0(8)	-9.8(6)	-11.4(6)	-14.2(6)
C(19)	30.8(6)	28.4(7)	34.9(7)	-6.9(5)	-11.3(5)	-12.9(5)
C(20)	26.8(6)	21.0(6)	29.2(6)	-4.2(5)	-9.5(5)	-6.3(5)
C(21)	26.5(6)	22.3(6)	25.8(6)	-6.6(5)	0.7(5)	-10.2(5)
C(22)	29.0(6)	23.4(6)	18.0(5)	-1.5(4)	-0.2(4)	-12.1(5)
C(23)	28.5(6)	25.8(6)	25.1(6)	-2.4(5)	-1.9(5)	-11.3(5)
C(24)	38.9(7)	32.2(7)	27.4(6)	-0.4(5)	-5.8(5)	-21.2(6)
C(25)	50.6(8)	22.0(6)	23.4(6)	-0.5(5)	-4.6(5)	-18.2(6)
C(26)	40.2(7)	20.5(6)	32.5(7)	1.7(5)	-3.0(6)	-6.9(5)
C(27)	29.9(6)	25.8(6)	35.0(7)	1.2(5)	-5.8(5)	-11.1(5)
O(1)	37.9(5)	33.2(5)	25.2(4)	-5.9(4)	0.8(4)	-17.1(4)
N(4)	50.3(7)	29.5(6)	31.2(6)	-7.5(5)	-4.3(5)	-19.3(5)
N(5)	25.2(5)	21.2(5)	22.9(5)	-6.0(4)	-1.7(4)	-10.4(4)
N(6)	21.4(5)	16.9(5)	24.9(5)	-1.4(4)	-7.0(4)	-7.2(4)
C(28)	26.8(6)	20.1(6)	26.8(6)	-4.7(5)	-8.8(5)	-3.0(5)
C(29)	63.3(10)	34.2(8)	44.8(9)	-10.5(6)	-7.4(7)	-26.7(7)
C(30)	52.7(9)	32.0(8)	52.8(9)	-20.9(7)	-17.3(7)	-9.2(7)
C(31)	32.5(7)	41.7(8)	40.3(8)	-23.9(6)	-10.8(6)	1.3(6)
C(32)	24.6(6)	34.8(7)	31.5(7)	-13.6(5)	-6.7(5)	-2.1(5)

C(33)	24.2(6)	19.9(6)	23.2(6)	-3.1(5)	-6.3(5)	-3.6(5)
C(34)	20.9(5)	20.7(6)	23.0(6)	-5.9(4)	-2.8(4)	-8.7(4)
C(35)	22.6(5)	21.8(6)	18.2(5)	-4.9(4)	1.2(4)	-9.2(5)
C(36)	26.2(6)	25.9(6)	24.3(6)	-4.4(5)	-3.1(5)	-10.8(5)
C(37)	28.5(6)	31.4(7)	33.5(7)	-2.4(5)	-9.7(5)	-5.7(5)
C(38)	38.0(7)	21.5(6)	42.5(8)	-1.1(6)	-9.0(6)	-5.0(5)
C(39)	41.3(8)	24.1(7)	46.8(8)	-4.8(6)	-10.4(6)	-14.9(6)
C(40)	28.5(6)	25.8(6)	34.0(7)	-4.8(5)	-7.4(5)	-11.3(5)
C(41)	23.3(6)	19.8(6)	22.4(6)	-4.2(4)	-5.6(4)	-8.5(4)
C(42)	23.0(6)	17.3(5)	21.5(6)	-1.1(4)	-5.1(4)	-7.4(4)
C(43)	27.6(6)	20.3(6)	30.8(6)	1.4(5)	-12.2(5)	-8.5(5)
C(44)	34.4(7)	18.6(6)	43.1(8)	-0.8(5)	-15.4(6)	-6.3(5)
C(45)	41.2(7)	23.0(6)	47.8(8)	1.2(6)	-18.4(6)	-16.1(6)
C(46)	33.0(7)	26.5(7)	49.8(8)	-0.1(6)	-20.5(6)	-13.7(5)
C(47)	23.4(6)	21.6(6)	42.2(7)	-2.2(5)	-11.6(5)	-7.0(5)
C(48)	29.9(6)	21.8(6)	22.5(6)	-2.1(5)	-2.7(5)	-10.7(5)
C(49)	25.5(6)	22.4(6)	19.2(5)	-4.8(4)	1.6(4)	-7.9(5)
C(50)	37.5(7)	28.6(7)	36.3(7)	-7.7(6)	-14.6(6)	-4.9(6)
C(51)	50.8(8)	22.6(7)	38.2(8)	-2.6(6)	-15.7(7)	-1.0(6)
C(52)	44.6(8)	21.8(6)	27.5(6)	-7.5(5)	2.8(6)	-13.2(6)
C(53)	32.3(7)	31.3(7)	30.4(7)	-10.3(5)	-2.0(5)	-13.3(5)
C(54)	29.6(6)	24.7(6)	25.2(6)	-4.4(5)	-5.5(5)	-6.9(5)

Supplementary Table 4. Bond Lengths for **4b**.

Atom	Atom	Length/Å	Atom	Atom	Length/Å
O(1)	C(6)	1.2305(15)	O(2)	C(33)	1.2313(15)
N(1)	C(1)	1.3369(17)	N(4)	C(28)	1.3388(17)
N(1)	C(2)	1.3370(17)	N(4)	C(29)	1.3368(18)
N(2)	C(6)	1.3384(15)	N(5)	C(33)	1.3383(15)

N(2)	C(7)	1.4587(14)	N(5)	C(34)	1.4556(15)
N(3)	C(15)	1.4716(14)	N(6)	C(42)	1.4756(14)
N(3)	C(16)	1.4717(15)	N(6)	C(43)	1.4708(14)
N(3)	C(20)	1.4667(15)	N(6)	C(47)	1.4667(14)
C(1)	C(5)	1.3855(17)	C(28)	C(32)	1.3854(18)
C(1)	C(6)	1.5070(16)	C(28)	C(33)	1.5045(17)
C(2)	C(3)	1.381(2)	C(29)	C(30)	1.379(2)
C(3)	C(4)	1.375(2)	C(30)	C(31)	1.375(2)
C(4)	C(5)	1.3864(19)	C(31)	C(32)	1.3809(19)
C(7)	C(8)	1.5228(16)	C(34)	C(35)	1.5224(16)
C(7)	C(14)	1.5370(16)	C(34)	C(41)	1.5389(15)
C(8)	C(9)	1.3914(16)	C(35)	C(36)	1.3888(17)
C(8)	C(13)	1.3884(16)	C(35)	C(40)	1.3915(17)
C(9)	C(10)	1.3849(18)	C(36)	C(37)	1.3895(18)
C(10)	C(11)	1.3842(19)	C(37)	C(38)	1.3788(19)
C(11)	C(12)	1.3844(18)	C(38)	C(39)	1.385(2)
C(12)	C(13)	1.3916(17)	C(39)	C(40)	1.3825(19)
C(14)	C(15)	1.5365(16)	C(41)	C(42)	1.5338(15)
C(15)	C(21)	1.5451(15)	C(42)	C(48)	1.5494(16)
C(16)	C(17)	1.5196(16)	C(43)	C(44)	1.5223(17)
C(17)	C(18)	1.5289(18)	C(44)	C(45)	1.5209(18)
C(18)	C(19)	1.5227(18)	C(45)	C(46)	1.5241(18)
C(19)	C(20)	1.5201(16)	C(46)	C(47)	1.5193(17)
C(21)	C(22)	1.5095(16)	C(48)	C(49)	1.5071(16)
C(22)	C(23)	1.3896(17)	C(49)	C(50)	1.3930(18)
C(22)	C(27)	1.3935(18)	C(49)	C(54)	1.3852(18)
C(23)	C(24)	1.3893(18)	C(50)	C(51)	1.3863(19)
C(24)	C(25)	1.381(2)	C(51)	C(52)	1.382(2)

C(25)	C(26)	1.382(2)	C(52)	C(53)	1.3760(19)
C(26)	C(27)	1.3869(19)	C(53)	C(54)	1.3892(18)

Supplementary Table 5. Bond Angles for **4b**.

Atom	Atom	Atom	**Angle/°**	Atom	Atom	Atom	**Angle/°**
C(1)	N(1)	C(2)	116.94(11)	C(29)	N(4)	C(28)	116.69(12)
C(6)	N(2)	C(7)	121.67(10)	C(33)	N(5)	C(34)	121.16(10)
C(15)	N(3)	C(16)	114.79(9)	C(43)	N(6)	C(42)	114.66(9)
C(20)	N(3)	C(15)	112.28(9)	C(47)	N(6)	C(42)	111.93(9)
C(20)	N(3)	C(16)	109.76(9)	C(47)	N(6)	C(43)	109.46(9)
N(1)	C(1)	C(5)	123.14(11)	N(4)	C(28)	C(32)	123.28(12)
N(1)	C(1)	C(6)	117.01(10)	N(4)	C(28)	C(33)	117.19(11)
C(5)	C(1)	C(6)	119.85(11)	C(32)	C(28)	C(33)	119.52(11)
N(1)	C(2)	C(3)	123.81(14)	N(4)	C(29)	C(30)	123.86(14)
C(4)	C(3)	C(2)	118.72(13)	C(31)	C(30)	C(29)	118.72(13)
C(3)	C(4)	C(5)	118.50(13)	C(30)	C(31)	C(32)	118.64(13)
C(1)	C(5)	C(4)	118.86(13)	C(31)	C(32)	C(28)	118.78(13)
O(1)	C(6)	N(2)	123.89(11)	O(2)	C(33)	N(5)	123.83(11)
O(1)	C(6)	C(1)	120.88(10)	O(2)	C(33)	C(28)	120.63(11)
N(2)	C(6)	C(1)	115.23(10)	N(5)	C(33)	C(28)	115.53(10)
N(2)	C(7)	C(8)	113.71(9)	N(5)	C(34)	C(35)	113.19(9)
N(2)	C(7)	C(14)	109.16(9)	N(5)	C(34)	C(41)	109.80(9)
C(8)	C(7)	C(14)	110.96(9)	C(35)	C(34)	C(41)	110.93(9)
C(9)	C(8)	C(7)	118.50(10)	C(36)	C(35)	C(34)	123.25(10)
C(13)	C(8)	C(7)	122.99(10)	C(36)	C(35)	C(40)	118.35(11)
C(13)	C(8)	C(9)	118.45(11)	C(40)	C(35)	C(34)	118.36(10)
C(10)	C(9)	C(8)	120.94(11)	C(35)	C(36)	C(37)	120.50(11)
C(11)	C(10)	C(9)	120.26(12)	C(38)	C(37)	C(36)	120.53(12)

C(10)	C(11)	C(12)	119.40(12)	C(37)	C(38)	C(39)	119.46(12)
C(11)	C(12)	C(13)	120.25(11)	C(40)	C(39)	C(38)	120.05(12)
C(8)	C(13)	C(12)	120.69(11)	C(39)	C(40)	C(35)	121.11(12)
C(15)	C(14)	C(7)	114.05(9)	C(42)	C(41)	C(34)	114.40(9)
N(3)	C(15)	C(14)	110.87(9)	N(6)	C(42)	C(41)	110.91(9)
N(3)	C(15)	C(21)	115.62(9)	N(6)	C(42)	C(48)	115.33(9)
C(14)	C(15)	C(21)	111.66(9)	C(41)	C(42)	C(48)	111.39(9)
N(3)	C(16)	C(17)	109.94(10)	N(6)	C(43)	C(44)	110.11(10)
C(16)	C(17)	C(18)	110.54(10)	C(45)	C(44)	C(43)	111.05(11)
C(19)	C(18)	C(17)	110.04(11)	C(44)	C(45)	C(46)	109.82(10)
C(20)	C(19)	C(18)	110.61(10)	C(47)	C(46)	C(45)	110.42(11)
N(3)	C(20)	C(19)	110.25(9)	N(6)	C(47)	C(46)	110.83(10)
C(22)	C(21)	C(15)	111.62(10)	C(49)	C(48)	C(42)	111.53(9)
C(23)	C(22)	C(21)	121.00(11)	C(50)	C(49)	C(48)	120.92(11)
C(23)	C(22)	C(27)	118.04(11)	C(54)	C(49)	C(48)	121.01(11)
C(27)	C(22)	C(21)	120.89(11)	C(54)	C(49)	C(50)	118.01(11)
C(24)	C(23)	C(22)	121.05(12)	C(51)	C(50)	C(49)	120.67(12)
C(25)	C(24)	C(23)	120.17(12)	C(52)	C(51)	C(50)	120.46(12)
C(24)	C(25)	C(26)	119.52(12)	C(53)	C(52)	C(51)	119.45(12)
C(25)	C(26)	C(27)	120.26(12)	C(52)	C(53)	C(54)	120.05(12)
C(26)	C(27)	C(22)	120.93(12)	C(49)	C(54)	C(53)	121.29(12)

Supplementary Table 6. Torsion Angles for **4b.**

A	B	C	D	Angle/°	A	B	C	D	Angle/°
N(1)	C(1)	C(5)	C(4)	-1.80(18)	N(4)	C(28)	C(32)	C(31)	2.18(19)
N(1)	C(1)	C(6)	O(1)	171.41(11)	N(4)	C(28)	C(33)	O(2)	-177.80(11)
N(1))	C(1)	C(6)	N(2)	-8.49(15)	N(4)	C(28)	C(33)	N(5)	1.14(15)
N(1)	C(2)	C(3)	C(4)	-1.2(2)	N(4)	C(29)	C(30)	C(31)	1.5(2)

N(2)	C(7)	C(8)	C(9)	-153.89(10)	N(5)	C(34)	C(35)	C(36)	-22.14(15)
N(2)	C(7)	C(8)	C(13)	29.06(15)	N(5)	C(34)	C(35)	C(40)	160.09(10)
N(2)	C(7)	C(14)	C(15)	-58.34(12)	N(5)	C(34)	C(41)	C(42)	58.06(12)
N(3)	C(15)	C(21)	C(22)	-168.43(9)	N(6)	C(42)	C(48)	C(49)	167.50(9)
N(3)	C(16)	C(17)	C(18)	-58.24(14)	N(6)	C(43)	C(44)	C(45)	58.07(14)
C(1)	N(1)	C(2)	C(3)	0.0(2)	C(28)	N(4)	C(29)	C(30)	-0.7(2)
C(2)	N(1)	C(1)	C(5)	1.54(18)	C(29)	N(4)	C(28)	C(32)	-1.19(19)
C(2)	N(1)	C(1)	C(6)	-178.29(11)	C(29)	N(4)	C(28)	C(33)	177.87(12)
C(2	C(3)	C(4)	C(5)	0.9(2)	C(29)	C(30)	C(31)	C(32)	-0.5(2)
C(3)	C(4)	C(5)	C(1)	0.50(19)	C(30)	C(31)	C(32)	C(28)	-1.25(19)
C(5)	C(1)	C(6)	O(1)	-8.42(17)	C(32)	C(28)	C(33)	O(2)	1.30(17)
C(5)	C(1)	C(6)	N(2)	171.68(10)	C(32)	C(28)	C(33)	N(5)	-179.77(10)
C(6)	N(2)	C(7)	C(8)	75.64(13)	C(33)	N(5)	C(34)	C(35)	-80.54(13)
C(6)	N(2)	C(7)	C(14)	-159.88(10)	C(33)	N(5)	C(34)	C(41)	154.91(10)
C(6)	C(1)	C(5)	C(4)	178.03(11)	C(33)	C2(8)	C(32)	C(31)	-176.86(11)
C(7)	N(2)	C(6)	O(1)	-4.08(17)	C(34)	N(5v	C(33)	O(2)	2.81(17)
C(7)	N(2)	C(6)	C(1)	175.82(9)	C(34)	N(5)	C(33)	C(28)	-176.09(9)
C(7)	C(8)	C(9)	C(10)	-177.25(11)	C(34)	C(35)	C(36)	C(37)	-177.59(11)
C(7)	C(8)	C(13)	C(12)	176.39(11)	C(34)	C(35)	C(40)	C(39)	177.63(12)
C(7)	C(14)	C(15)	N(3)	77.55(11)	C(34)	C(41)	C(42)	N(6)	-75.71(11)
C(7)	C(14)	C(15)	C(21)	-151.96(9)	C(34)	C(41)	C(42)	C(48)	154.39(9)
C(8)	C(7)	C(14)	C(15)	67.74(12)	C(35)	C(34)	C(41)	C(42)	-67.79(12)
C(8)	C(9)	C(10)	C(11)	0.5(2)	C(35)	C(36)	C(37)	C(38)	-0.07(19)
C(9)	C(8)	C(13)	C(12)	-0.66(17)	C(36)	C(35)	C(40)	C(39)	-0.25(19)
C(9)	C(10)	C(11)	C(12)	-0.3(2)	C(36)	C(37)	C(38)	C(39)	0.0(2)
C(10)	C(11)	C(12)	C(13)	-0.4(2)	C(37)	C(38)	C(39)	C(40)	-0.1(2)
C(11)	C(12)	C(13)	C(8)	0.92(19)	C(38)	C(39)	C(40)	C(35)	0.2(2)
C(13)	C(8)	C(9)	C(10)	-0.07(18)	C(40)	C(35)	C(36)	C(37)	0.19(18)

C(14)	C(7)	C(8)	C(9)	82.60(13)	C(41)	C(34)	C(35)	C(36)	101.80(12)
C(14)	C(7)	C(8)	C(13)	-94.45(12)	C(41)	C(34)	C(35)	C(40)	-75.98(13)
C(14)	C(15)	C(21)	C(22)	63.58(12)	C(41)	C(42)	C(48)	C(49)	-64.95(12)
C(15)	N(3)	C(16)	C(17)	-169.84(10)	C(42)	N(6)	C(43)	C(44)	171.62(10)
C(15)	N(3)	C(20)	C(19)	168.57(10)	C(42)	N(6)	C(47)	C(46)	-169.58(10)
C(15)	C(21)	C(22)	C(23)	-122.17(12)	C(42)	C(48)	C(49)	C(50)	-61.56(15)
C(15)	C(21)	C(22)	C(27)	54.84(15)	C(42)	C(48)	C(49)	C(54)	115.58(12)
C(16)	N(3)	C(15)	C(14)	75.14(12)	C(43)	N(6)	C(42)	C(41)	-72.51(12)
C(16)	N(3)	C(15)	C(21)	-53.24(13)	C(43)	N(6)	C(42)	C(48)	55.28(13)
C(16)	N(3)	C(20)	C(19)	-62.48(13)	C(43)	N(6)	C(47)	C(46)	62.16(13)
C(16)	C(17)	C(18)	C(19)	53.55(15)	C(43)	C(44)	C(45)	C(46)	-53.57(16)
C(17)	C(18)	C(19)	C(20)	-53.29(15)	C(44)	C(45)	C(46)	C(47)	53.27(16)
C(18)	C(19)	C(20)	N(3)	58.04(14)	C(45)	C(46)	C(47)	N(6)	-58.23(15)
C(20)	N(3)	C(15)	C(14)	-158.59(9)	C(47)	N(6)	C(42)	C(41)	162.04(10)
C(20)	N(3)	C(15)	C(21)	73.03(12)	C(47)	N(6)	C(42)	C(48)	-70.16(12)
C(20)	N(3)	C(16)	C(17)	62.59(13)	C(47)	N(6)	C(43)	C(44)	-61.65(13)
C(21)	C(22)	C(23)	C(24)	175.85(11)	C(48)	C(49)	C(50)	C(51)	174.64(12)
C(21)	C(22)	C(27)	C(26)	-175.24(11)	C(48)	C(49)	C(54)	C(53)	-174.83(11)
C(22)	C(23)	C(24)	C(25)	-0.32(19)	C((49)	C(50)	C(51)	C(52)	0.8(2)
C(23)	C(22)	C(27)	C(26)	1.85(19)	C(50)	C(49)	C(54)	C(53)	2.38(18)
C(23)	C(24)	C(25)	C(26)	1.29(19)	C(50)	C(51)	C(52)	C(53)	1.3(2)
C(24)	C(25)	C(26)	C(27)	-0.68(19)	C(51)	C(52)	C(53)	C(54)	-1.5(2)
C(25)	C(26)	C(27)	C(22)	-0.9(2)	C(52)	C(53)	C(54)	C(49)	-0.36(19)
C(27)	C(22)	C(23)	C(24)	-1.24(18)	C(54)	C(49)	C(50)	C(51)	-2.6(2)

Supplementary Table 7. Hydrogen Atom Coordinates (Å×10^4) and Isotropic Displacement Parameters (Å^2×10^3) for **4b.**

Atom	x	y	z	U(eq)
H(2)	2045	8027	5127	27

H(2A)	3286	10716	4946	45
H(3)	4805	11112	3905	48
H(4)	5576	9964	2912	45
H(5)	4732	8471	2997	36
H(7)	1072	6986	4414	25
H(9)	1124	4980	4586	30
H(10)	2239	2998	4790	38
H(11)	4009	2351	5338	38
H(12)	4662	3708	5673	35
H(13)	3569	5700	5444	29
H(14A)	-275	7919	5439	25
H(14B)	-176	6587	5648	25
H(15)	1464	6403	6339	24
H(16A)	-587	9103	6985	32
H(16B)	-664	9296	6107	32
H(17A)	-393	10951	6389	39
H(17B)	770	10361	5761	39
H(18A)	1576	10735	6690	40
H(18B)	767	10089	7366	40
H(19A)	2848	8972	6239	35
H(19B)	2825	8792	7131	35
H(20A)	2490	7185	6826	30
H(20B	1297	7838	7414	30
H(21A	-65	6776	7518	30
H(21B)	-1132	7325	7031	30
H(23)	-2242	6019	7114	32
H(24)	-2313	4184	7075	37
H(25)	-477	2581	6996	37

H(26)	1421	2811	7000	41
H(27)	1490	4636	7060	37
H(5A)	6430	2955	855	27
H(29)	7038	5500	1559	53
H(30)	6022	6081	2750	52
H(31)	4666	5114	3587	46
H(32)	4364	3597	3178	37
H(34)	4772	1871	876	25
H(36)	7751	715	1390	30
H(37)	8814	-1260	1722	38
H(38)	8113	-2684	1528	43
H(39)	6333	-2125	997	43
H(40)	5268	-158	668	34
H(41A)	5884	1533	-396	25
H(41B)	5454	2865	-311	25
H(42)	7920	1426	-175	25
H(43A)	6091	4331	-788	31
H(43B)	7413	4230	-1355	31
H(44A)	6641	6024	-866	38
H(44B)	6524	5357	-11	38
H(45A)	8381	5838	-329	42
H(45B)	8841	5263	-1093	42
H(46A)	9907	3923	-168	40
H(46B)	8626	4008	440	40
H(47A)	9327	3006	-944	34
H(47B)	9291	2276	-107	34
H(48A)	7288	2433	-1646	30
H(48B)	8696	1948	-1536	30

H(50)	9286	-268	-774	41
H(51)	9252	-2126	-783	47
H(52)	7895	-2367	-1419	38
H(53)	6621	-741	-2082	36
H(54)	6642	1120	-2068	32

Supplementary Table 8. Crystal data and structure refinement for **4i**.

Identification code	4i
Empirical formula	$C_{25}H_{33}N_3O_2$
Formula weight	407.54
Temperature/K	293(2)
Crystal system	monoclinic
Space group	$P2_1/c$
a/Å	21.748(3)
b/Å	5.4836(6)
c/Å	19.4942(13)
α/°	90
β/°	99.738(10)
γ/°	90
Volume/Å^3	2291.3(4)
Z	4
ρ_{calc}g/cm^3	1.181
μ/mm^{-1}	0.593
F(000)	880.0
Crystal size/mm^3	0.2 × 0.15 × 0.4
Radiation	CuKα (λ = 1.54184)
2Θ range for data collection/°	8.25 to 134.122
Index ranges	$-24 \leq h \leq 25, -4 \leq k \leq 6, -21 \leq l \leq 23$

Reflections collected	7506
Independent reflections	4044 [R_{int} = 0.0533, Rsigma = 0.0617]
Data/restraints/parameters	4044/0/273
Goodness-of-fit on F^2	1.072
Final R indexes [I>=2σ (I)]	R_1 = 0.0787, wR_2 = 0.1919
Final R indexes [all data]	R_1 = 0.1287, wR_2 = 0.2353
Largest diff. peak/hole / e Å^{-3}	0.22/-0.30

Supplementary Table 9. Fractional Atomic Coordinates (×10^4) and Equivalent Isotropic Displacement Parameters (Å^2×10^3) for **4i**. U_{eq} is defined as 1/3 of the trace of the orthogonalised U_{IJ} tensor.

Atom	x	y	z	U(eq)
O(1)	6302.2(19)	7341(9)	4638.9(18)	116.3(14)
O(2)	6847.6(16)	9790(7)	7094.0(15)	93.3(11)
N(1)	5720.6(18)	3411(9)	3245.0(19)	89.4(13)
N(2)	6548.4(15)	7067(8)	3558.6(19)	81.9(11)
N(3	8573.8(13)	10317(6)	3350.3(12)	49.6(7)
C(1)	5769.7(18)	4386(9)	3870(2)	72.5(12)
C(2)	5293(3)	1684(14)	3070(3)	118(2)
C(3)	4897(3)	933(14)	3489(4)	124(2)
C(4)	4947(3)	1900(16)	4128(4)	137(3)
C(5)	5398(3)	3662(15)	4338(3)	123(2)
C(6)	6230.1(19)	6408(10)	4061(2)	77.8(13)
C(7)	6992(2)	9091(10)	3642(3)	88.0(14)
C(8)	7650.0(17)	8269(7)	3758.6(17)	57.9(9)
C(9)	8131.4(16)	10365(7)	3848.4(14)	48.6(8)
C(10)	8264.7(18)	10872(8)	2644.6(15)	60.3(10)
C(11)	8739(2)	11116(9)	2152.8(17)	69.7(12)

C(12)	9133.4(19)	8867(9)	2167.6(17)	68.8(11)
C(13)	9435.0(18)	8309(9)	2907.1(18)	71.1(12)
C(14)	8941.4(18)	8085(8)	3369.7(17)	61.4(10)
C(15)	8491.2(16)	10517(7)	4605.5(15)	53.3(9)
C(16)	8969.6(19)	12585(9)	4697.9(17)	69.2(12)
C(17)	9397(2)	12568(12)	5399(2)	100.6(19)
C(18)	8044.5(16)	10732(7)	5126.8(15)	50.5(8)
C(19)	7637.5(19)	12682(7)	5108.8(16)	61.2(10)
C(20)	7238.0(19)	12905(8)	5594.7(17)	62.2(10)
C(21)	7241.3(17)	11196(7)	6110.9(15)	54.4(9)
C(22)	7639.8(18)	9224(8)	6124.2(16)	59.5(10)
C(23)	8039.4(18)	9022(7)	5640.3(15)	56.9(9)
C(24)	6832.9(18)	11364(9)	6656.7(19)	64.8(11)
C(25)	6427(2)	13553(10)	6656(3)	89.9(15)

Supplementary Table 10. Anisotropic Displacement Parameters (Å^2×10^3) for **4i**. The Anisotropic displacement factor exponent takes the form: -2π^2[$h^2a^{*2}U_{11}$+2hka*b*U_{12}+…].

Atom	U_{11}	U_{22}	U_{33}	U_{23}	U_{13}	U_{12}
O(1)	118(3)	148(4)	81(2)	-32(2)	13(2)	-14(3)
O(2)	108(2)	111(3)	69.9(17)	17.9(19)	39.2(17)	-3(2)
N(1)	80(3)	117(4)	70(2)	3(2)	9.6(18)	-30(3)
N(2)	57(2)	107(3)	81(2)	-9(2)	11.8(17)	-22(2)
N(3)	52.0(16)	61.6(19)	34.0(12)	4.4(12)	3.6(11)	0.5(14)
C(1)	50(2)	95(4)	72(2)	16(2)	9.4(18)	0(2)
C(2)	96(4)	155(6)	102(4)	-13(4)	19(3)	-53(4)
C(3)	91(4)	143(6)	142(5)	4(5)	29(4)	-38(4)
C(4)	113(5)	173(7)	140(6)	24(5)	63(4)	-40(5)
C(5)	104(4)	177(7)	98(4)	4(4)	47(3)	-30(5)

C(6)	56(2)	101(4)	73(2)	-4(3)	2.7(19)	-5(2)
C(7)	60(3)	89(4)	113(4)	-4(3)	8(2)	-13(3)
C(8)	62(2)	63(2)	47.6(16)	2.0(16)	4.7(15)	-8.8(19)
C(9)	52.5(19)	55(2)	37.7(14)	3.7(14)	5.2(13)	-0.5(17)
C(10)	65(2)	76(3)	37.7(15)	3.2(16)	2.9(15)	11(2)
C(11)	83(3)	85(3)	41.7(16)	11.9(18)	13.5(17)	3(2)
C(12)	65(2)	95(3)	47.6(17)	-3(2)	13.3(16)	-1(2)
C(13)	58(2)	100(3)	54.8(19)	1(2)	9.7(16)	10(2)
C(14)	61(2)	74(3)	47.6(17)	11.3(18)	6.9(15)	11(2)
C(15)	59(2)	63(2)	36.5(14)	3.3(15)	6.1(14)	-4.7(18)
C(16)	68(2)	93(3)	45.6(17)	-5.5(19)	8.1(16)	-18(2)
C(17)	80(3)	166(6)	54(2)	-20(3)	6(2)	-33(4)
C(18)	61(2)	51(2)	37.3(14)	1.9(14)	3.5(14)	-2.9(18)
C(19)	81(3)	61(3)	42.5(16)	13.4(17)	13.4(16)	0(2)
C(20)	71(2)	64(3)	52.0(18)	-1.8(18)	9.7(16)	3(2)
C(21)	58(2)	64(2)	40.6(15)	-0.1(16)	6.8(14)	-10.6(19)
C(22)	73(2)	63(3)	42.5(16)	11.6(16)	9.1(16)	-6(2)
C(23)	67(2)	63(2)	40.4(15)	7.2(16)	6.4(15)	3(2)
C(24)	61(2)	80(3)	54.3(19)	-7(2)	11.6(17)	-12(2)
C(25)	80(3)	105(4)	92(3)	-12(3)	35(3)	-7(3)

Supplementary Table 11. Bond Lengths for **4i**.

Atom	Atom	Length/Å	Atom	Atom	Length/Å
O(1)	C(6)	1.222(5)	C9	C15	1.551(4)
O(2)	C(24)	1.210(5)	C10	C11	1.528(5)
N(1)	C(1)	1.318(6)	C11	C12	1.499(6)
N(1)	C(2)	1.331(7)	C12	C13	1.511(5)
N(2)	C(6)	1.342(5)	C13	C14	1.519(5)
N(2)	C(7)	1.461(6)	C15	C16	1.529(5)

N(3)	C(9)	1.478(4)	C15	C18	1.524(4)
N(3)	C(10)	1.458(4)	C16	C17	1.518(5)
N(3	C(14)	1.459(5)	C18	C19	1.385(5)
C(1)	C(5)	1.375(6)	C18	C23	1.373(5)
C(1)	C(6)	1.498(7)	C19	C20	1.395(5)
C(2)	C(3)	1.347(8)	C20	C21	1.374(5)
C(3)	C(4)	1.341(9)	C21	C22	1.383(5)
C(4)	C(5)	1.389(9)	C21	C24	1.500(5)
C(7)	C(8)	1.481(6)	C22	C23	1.391(5)
C(8)	C(9)	1.545(5)	C24	C25	1.490(7)

Supplementary Table 12. Bond Angles for **4i**.

Atom	Atom	Atom	Angle/˚	Atom	Atom	Atom	Angle/˚
C(1)	N(1)	C(2)	118.0(4)	C(12)	C(11)	C(10)	111.1(3)
C(6)	N(2)	C(7)	122.4(4)	C(11)	C(12)	C(13)	109.9(3)
C(10)	N(3)	C(9)	111.6(3)	C(12)	C(13)	C(14)	110.3(3)
C(10)	N(3)	C(14)	111.3(3)	N(3)	C(14)	C(13)	110.9(3)
C(14)	N(3)	C(9)	114.4(3)	C(16)	C(15)	C(9)	112.1(3)
N(1)	C(1)	C(5)	122.1(5)	C(18)	C(15)	C(9)	111.3(3)
N(1)	C(1)	C(6)	118.5(4)	C(18)	C(15)	C(16)	111.2(3)
C(5)	C(1)	C(6)	119.4(5)	C(17)	C(16)	C(15)	113.7(4)
N(1)	C(2)	C(3)	123.6(6)	C(19)	C(18)	C(15)	121.3(3)
C(4)	C(3)	C(2)	118.8(6)	C(23)	C(18)	C(15)	121.2(3)
C(3)	C(4)	C(5)	119.5(5)	C(23)	C(18)	C(19)	117.5(3)
C(1)	C(5)	C(4)	118.0(6)	C(18)	C(19)	C(20)	121.3(3)
O(1)	C(6)	N(2)	124.0(5)	C(21)	C(20)	C(19)	120.7(4)
O(1)	C(6)	C(1)	121.1(4)	C(20)	C(21)	C(22)	118.2(3)
N(2)	C(6)	C(1)	115.0(4)	C(20)	C(21)	C(24)	123.2(4)

N(2)	C(7)	C(8)	112.9(4)	C(22)	C(21)	C(24)	118.6(3)
C(7)	C(8)	C(9)	114.2(4)	C(21)	C(22)	C(23)	120.7(3)
N(3)	C(9)	C(8)	114.6(3)	C(18)	C(23)	C(22)	121.5(4)
N(3)	C(9)	C(15)	110.3(3)	O(2)	C(24)	C(21)	120.5(4)
C(8)	C(9)	C(15)	111.8(3)	O(2)	C(24)	C(25)	121.3(4)
N(3)	C(10)	C(11)	111.0(3)	C(25)	C(24)	C(21)	118.1(4)

Supplementary Table 13. Torsion Angles for **4i.**

A	B	C	D	Angle/˚	A	B	C	D	Angle/˚
N(1)	C(1)	C(5)	C(4)	-2.0(10)	C(9)	C(15)	C(18)	C(23)	120.4(4)
N(1)	C(1)	C(6)	O(1)	-178.6(5)	C(10)	N(3)	C(9)	C(8)	69.2(4)
N(1)	C(1)	C(6)	N(2)	1.0(7)	C(10)	N(3)	C(9)	C(15)	-163.7(3)
N(1)	C(2)	C(3)	C(4)	-2.6(12)	C(10)	N(3)	C(14)	C(13)	59.1(4)
N(2)	C(7)	C(8)	C(9)	180.0(3)	C(10)	C(11)	C(12)	C(13)	-54.6(5)
N(3)	C(9)	C(15)	C(16)	51.2(4)	C(11)	C(12)	C(13)	C(14)	55.6(5)
N(3)	C(9)	C(15)	C(18)	176.4(3)	C(12)	C(13)	C(14)	N(3)	-57.9(5)
N(3)	C(10)	C(11)	C(12)	55.8(5)	C(14)	N(3)	C(9)	C(8)	-58.3(4)
C(1)	N(1)	C(2)	C(3)	2.0(10)	C(14)	N(3)	C(9)	C(15)	68.8(4)
C(2)	N(1)	C(1)	C(5)	0.4(8)	C(14)	N(3)	C(10)	C(11)	-57.7(4)
C(2)	N(1)	C(1)	C(6)	-177.4(5)	C(15)	C(18)	C(19)	C(20)	-178.6(3)
C(2)	C(3)	C(4)	C(5)	0.8(13)	C(15)	C(18)	C(23)	C(22)	179.0(3)
C(3)	C(4)	C(5)	C(1)	1.4(12)	C(16)	C(15)	C(18)	C(19)	64.9(4)
C(5)	C(1)	C(6)	O(1)	3.6(8)	C(16)	C(15)	C(18)	C(23)	-113.8(4)
C(5)	C(1)	C(6)	N(2)	-176.8(5)	C(18)	C(15)	C(16)	C(17)	63.2(5)
C(6)	N(2)	C(7)	C(8)	105.1(5)	C(18)	C(19)	C(20)	C(21)	0.5(6)
C(6)	C(1)	C(5)	C(4)	175.7(6)	C(19)	C(18)	C(23)	C(22)	0.3(5)
C(7)	N(2)	C(6)	O(1)	-3.8(8)	C(19)	C(20)	C(21)	C(22)	-1.6(6)
C(7)	N(2)	C(6)	C(1)	176.7(4)	C(19)	C(20)	C(21)	C(24)	178.7(4)

C(7)	C(8)	C(9)	N(3)	-123.1(4)	C(20)	C(21)	C(22)	C(23)	2.0(5)
C(7)	C(8)	C(9)	C(15)	110.5(4)	C(20)	C(21)	C(24)	O(2)	178.9(4)
C(8)	C(9)	C(15)	C(16)	179.9(3)	C(20)	C(21)	C(24)	C(25)	-3.0(6)
C(8)	C(9)	C(15)	C(18)	-54.9(4)	C(21)	C(22)	C(23)	C(18)	-1.3(6)
C(9)	N(3)	C(10)	C(11)	173.2(3)	C(22)	C(21)	C(24)	O(2)	-0.8(6)
C(9)	N(3)	C(14)	C(13)	-173.2(3)	C(22)	C(21)	C(24)	C(25)	177.2(4)
C(9)	C(15)	C(16)	C(17)	-171.5(4)	C(23)	C(18)	C(19)	C(20)	0.1(5)
C(9)	C(15)	C(18)	C(19)	-60.8(5)	C(24)	C(21)	C(22)	C(23)	-178.3(3)

Supplementary Table 14. Hydrogen Atom Coordinates (Å×10^4) and Isotropic Displacement Parameters (Å^2×10^3) for **4i.**

Atom	x	y	z	U(eq)
H(2)	6489	6269	3173	98
H(2A)	5266	955	2635	141
H(3)	4594	-235	3337	149
H(4)	4682	1395	4427	165
H(5)	5448	4333	4782	148
H(7A)	6922	10085	4033	106
H(7B)	6916	10102	3228	106
H(8A)	7717	7269	3368	69
H(8B)	7723	7253	4172	69
H(9)	7894	11884	3762	58
H(10A)	8033	12384	2646	72
H(10B)	7971	9584	2480	72
H(11A)	8521	11390	1682	84
H(11B)	9005	12514	2289	84
H(12A)	9453	9115	1883	83
H(12B)	8876	7501	1978	83
H(13A)	9725	9601	3080	85

H(13B)	9668	6796	2918	85
H(14A)	8668	6724	3214	74
H(14B)	9141	7762	3844	74
H(15)	8721	8985	4703	64
H(16A)	9222	12466	4334	83
H(16B)	8750	14131	4641	83
H(17A)	9151	12715	5763	151
H(17B)	9682	13912	5425	151
H(17C)	9626	11066	5454	151
H(19)	7630	13866	4766	73
H(20)	6966	14225	5569	75
H(22)	7641	8020	6460	71
H(23)	8309	7698	5665	68
H(25A)	6195	13434	7032	135
H(25B)	6144	13643	6222	135
H(25C)	6682	14993	6715	135
